# Revision of the Afrotropical species of the hover fly genus *Mesembrius* Rondani (Diptera, Syrphidae) using morphological and molecular data

**DOI:** 10.3897/zookeys.1046.57052

**Published:** 2021-06-21

**Authors:** Kurt Jordaens, Georg Goergen, Jeffrey H. Skevington, Scott Kelso, Marc De Meyer

**Affiliations:** 1 Royal Museum for Central Africa, Invertebrates Section and JEMU, Leuvensesteenweg 13, B-3080 Tervuren, Belgium Royal Museum for Central Africa Tervuren Belgium; 2 International Institute for Tropical Agriculture (IITA), Biodiversity Centre, 08 BP 0932 Tri Postal, Cotonou, Benin International Institute for Tropical Agriculture Cotonou Benin; 3 Canadian National Collection of Insects, Arachnids and Nematodes, Agriculture and Agri-Food Canada, K.W. Neatby Building, 960 Carling Avenue, Ottawa,ON K1A0C6, Canada Canadian National Collection of Insects Ottawa Canada

**Keywords:** Africa, Afrotropical Region, DNA barcoding, Eristalinae, flower fly, new species, taxonomy

## Abstract

The Afrotropical representatives of the hover fly genus *Mesembrius* Rondani, 1857 (Diptera) are divided into two subgenera, namely *Mesembrius* s.s. and *Vadonimyia* Séguy, 1951 and, in this present work, the subgenus Mesembrius s.s. is revised. A total of 23 *Mesembrius* s.s. species are recognised for the Afrotropics. Known species are re-described and six species new to science are described: *Mesembrius arcuatus***sp. nov.**, *M. copelandi***sp. nov.**, *M. longipilosus***sp. nov.**, *M. sulcus***sp. nov.**, *M. tibialis***sp. nov.** and *M. vockerothi***sp. nov**. *Mesembrius africanus* (Verrall, 1898) is considered a junior synonym of *M. senegalensis* (Macquart, 1842), *M. ctenifer* Hull, 1941 a junior synonym of *M. caffer* (Loew, 1858), *M. lagopus* (Loew, 1869) a junior synonym of *M. capensis* (Macquart, 1842) and *M. platytarsis* Curran, 1929 a junior synonym of *M. simplicipes* Curran, 1929. The females of *Mesembrius chapini* Curran, 1939, *M. rex* Curran, 1927 and *M. regulus* (Hull, 1937) are described for the first time. Lectotypes are designated for *Mesembrius caffer*, *M. capensis*, *M. cyanipennis* (Bezzi, 1915), *M. minor* (Bezzi, 1915), *M. senegalensis*, *M. strigilatus* (Bezzi, 1912) and *M. tarsatus* (Bigot, 1883). Separate identification keys for males and females are presented. We obtained 236 DNA barcodes for 18 species. The relationships amongst the different *Mesembrius* species are briefly discussed, based on morphological and DNA barcode data.

## Introduction

Over the last two decades, there has been an increased activity on the taxonomy and systematics of Afrotropical hover flies (also called flower flies) (Diptera, Syrphidae). Indeed, Whittington (2003) in his assessment of the Afrotropical syrphid fauna points out that the taxonomy of most of the Afrotropical hover fly genera is poorly known and that generic identification keys are largely incomplete. However, in recent years, the number of taxonomic studies on the group is increasing with new identification keys for a number of genera, including *Afrosyrphus* Curran, 1927 (Mengual et al. 2020), *Ceriana* Rafinesque, 1815 (Thompson 2013), *Chasmomma* Bezzi, 1915 (Kassebeer 2000), Eristalinus Rondani, 1845 (subgenus Merodonoides) (Thompson 2019), *Megatrigon* Johnson, 1898 (Doczkal et al. 2016), (part of) *Merodon* Meigen, 1803 (Radenković et al. 2018), *Phytomia* Guérin-Méneville, 1834 (De Meyer et al. 2020a), *Senaspis* Macquart, 1850 (De Meyer et al. 2020b), *Syritta* Le Peletier & Serville, 1828 (Lyneborg and Barkemeyer 2005) and *Spheginobaccha* de Meijere, 1908 (Thompson and Hauser 2015). Nevertheless, gaps in our taxonomic knowledge of several other genera remain (Ssymank et al. in press), the genus *Mesembrius* Rondani, 1857 being one of them.

**Figures 1, 2.**
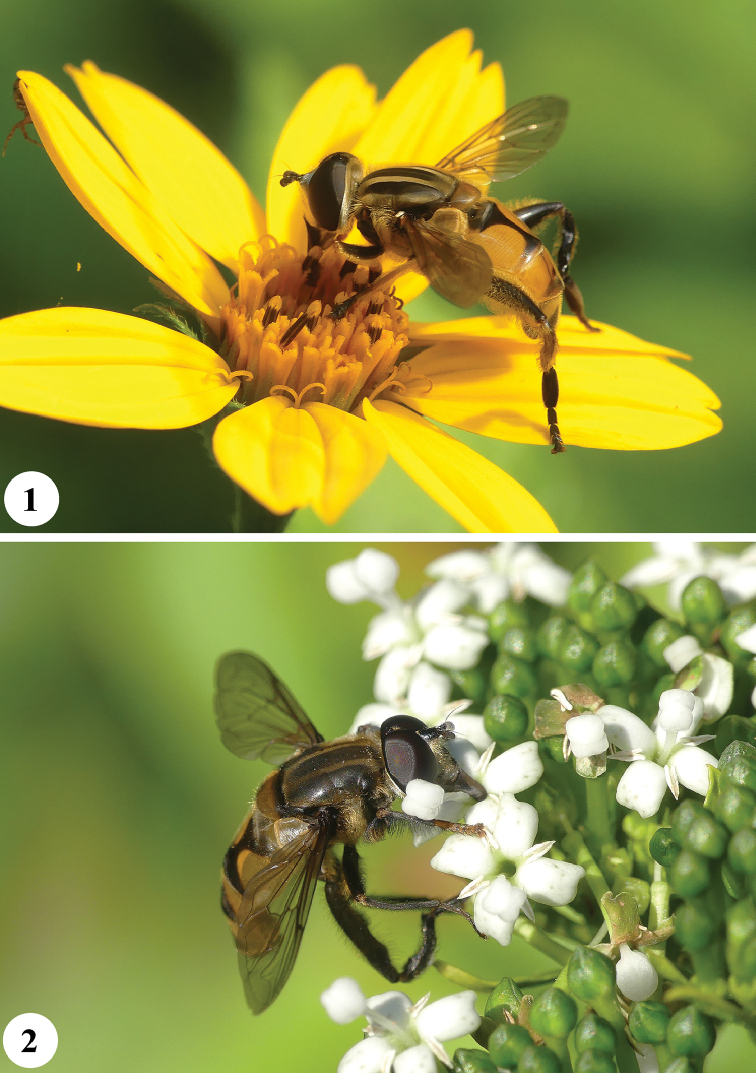
*Mesembrius* spp., live pictures **1***Mesembrius caffer* (Loew) (♂), Uganda. Photo: Menno Reemer **2***Mesembrius tarsatus* (Bigot) (♂), Uganda. Photo: Menno Reemer.

The genus *Mesembrius* (Figs 1, 2) is an Old World genus with some 58 described species, occurring in the Afrotropics, Australasia, Oriental Region and the Mediterranean Basin of the Palaearctic Region. Twenty-five species occur in the Afrotropical Region and are widely distributed on the African mainland and Madagascar. The genus comprises two subgenera, namely *Mesembrius* sensu stricto (hereafter as *Mesembrius* s.s.) with 21 species and *Vadonimyia* Séguy, 1951 with four species, of which the males have extremely enlarged terminalia (Hippa 1985). Whittington (2003), citing Thompson (1988), lists *Mesembrius strenuus* (Walker, 1857) from the Afrotropical Region, but the original description does not specify the origin of the species and Thompson (1988) stated “Palaeotropics” as its distribution. Dirickx (1998) considered *Vadonimyia* as a separate genus, following Hippa (1985). *Vadonimyia* and *Mesembrius* are considered monophyletic (Hippa 1985), but it remains subjective as to whether *Vadonimyia* should receive generic or sub-generic status. We, therefore, treat *Vadonimyia* as a subgenus until the phylogenetic affinities between both taxa are resolved (see also Hippa 1985). Here, we focus on the taxonomy of the subgenus Mesembrius s.s.

The taxonomy of the Afrotropical species of *Mesembrius* s.s. is puzzling and in need of revision. Six of the species are only known from their type(s) and many of the original descriptions lack sufficient detail to allow unambiguous identification. Detailed re-descriptions are mandatory to allow an unambiguous recognition of the different taxa. Females are particularly difficult to identify and several appear incorrectly identified (Curran 1939; see also Dirickx 1998: p. 83). For instance, Hervé-Bazin (1914a), Bezzi (1915) and Curran (1927) report females of *M. ingratus* (Loew, 1858) from the Democratic Republic of the Congo, Uganda and South Africa, respectively, but according to Curran (1939), all may belong to *M. tarsatus* (Bigot, 1883). Moreover, there is no identification key to all Afrotropical *Mesembrius* s.s. species. Bezzi (1915) and Curran (1927, 1939) provide incomplete keys, of which the key in Curran (1939) is the most complete with ten of the 21 species keyed out.

Several species show strong sexual dimorphism with males exhibiting long pile on the legs and modified metafemora with grooves and excavations (e.g. Figs 151–159, 183–187), while females have less conspicuous leg pile and unmodified metafemora (e.g. Figs 200–204; see also Discussion). Males, therefore, show more accessible and diagnostic morphological taxonomic characters than females. In addition to morphological examination, we use DNA barcoding (Hebert et al. 2003) to unambiguously associate females to males of the different species. Consequently, we provide separate identification keys for males and females. Finally, we briefly discuss relationships amongst the species and propose some future ideas on the study of the breeding biology of this genus.

## Materials and methods

### Examined collections

Specimens from the following institutional and private collections were studied:

**AMGS**Albany Museum of South Africa, Grahamstown, South Africa;

**AMNH**American Museum of Natural History, New York, USA;

**ANSP**Academy of Natural Sciences of Philadelphia, Philadelphia, USA;

**ASPC** Axel Ssymank Personal Collection, Wachtberg, Germany;

**BMSA**National Museum Bloemfontein, Bloemfontein, South Africa;

**CAS**California Academy of Sciences, San Francisco, USA;

**CNC**Canadian National Collections of Insects, Arachnids and Nematodes, Ottawa, Canada;

**CSCA**California State Collection of Arthropods, Sacramento, USA;

**DMSA**Durban Museum of South Africa, Durban, South Africa;

**ICIPE** International Centre of Insect Physiology and Ecology, Nairobi, Kenya;

**IITA**International Institute of Tropical Agriculture, Cotonou, Benin;

**KBIN** Koninklijk Belgisch Instituut voor Natuurwetenschappen, Brussel, Belgium;

**KMMA**Koninklijk Museum voor Midden Afrika, Tervuren, Belgium;

**MAPC** Michelson Azo’o Ela Personal Collection, Maroua, Cameroon;

**MNB**Museum für Naturkunde, Berlin, Germany;

**MNHN**Muséum national d’Histoire naturelle, Paris, France;

**MSNG**Museo Civico di Storia Naturale “Giacomo Doria”, Genoa, Italy;

**MZH**Finnish Museum of Natural History, Zoological Museum, Helsinki, Finland;

**NHMUK**The Natural History Museum, London, UK;

**NMB**Naturhistorisches Museum Basel, Basel, Switzerland;

**NMK**National Museums of Kenya, Nairobi, Kenya;

**NMSA**KwaZulu-Natal Museum, Pietermaritzburg, South Africa;

**NRMS**Naturhistoriska Riksmuseet, Stockholm, Sweden;

**OBPE** Office Burundais pour la Protection de l’Environnement, Bujumbura, Burundi;

**OXUM** Oxford University Museum, Oxford, UK;

**RMNH**Naturalis Biodiversity Center, Leiden, The Netherlands;

**SCPC** Simon Cavaillès Personal Collection, Kergoc, France;

**UNS**University of Novi Sad, Novi Sad, Serbia;

**ZFMK**Zoologisches Forschungsmuseum Alexander Koenig, Bonn, Germany.

Part of the material has been collected by the authors between 1994 and 2018. Mostly, hover flies were collected from agricultural land and its adjacent environment. Private grounds were never accessed without prior consent by the owners and were visited with national recruited staff and as part of the ongoing projects on pest control and biodiversity of the institutions. IITA is a non-profit international organisation and a member of the Consultative Group on International Agricultural Research (CGIAR) Consortium. Research work in Ghana, Benin and Nigeria is based on bilateral agreements in the form of memorandums of understanding (MoU), signed by the Ministries of Agriculture of all respective governments (more information can be found on http://www.iita.org), in which research work in the field is an integral part of IITA’s contracted mandate. In Togo, IITA has a close partnership with the National Plant Protection Service and the University of Lomé through which material was obtained. Therefore, no specific permissions were required for the collected hover flies. In Uganda, collecting permits were obtained from the Uganda Wildlife Authority (UWA/COD/96/05) and the Uganda National Council for Science and Technology (UNCST) (NS642). Permits for collecting in Malawi were obtained from the Forestry Research Institute of Malawi (FRIM). None of the collected species occurs on Red Lists or are considered to be endangered/threatened, neither is any ranked in IUCN lists or protected by CITES.

### Morphology

Morphological terminology follows Cumming and Wood (2017), except that we use the suffixes pro-, meso- and meta- to refer to the first, second and third pair of legs or leg parts, respectively. Morphological observations were made with a Leica MZ8 stereomicroscope. Since original descriptions were often very brief, written in different languages and using various terminologies, all species recognised as valid were re-described in order to allow comparison for all character states. Body length and wing length ranges given are minimum and maximum values observed in the studied material. Body measurements were taken between the frons and the posterior end of tergite IV, while wing measurements were taken between the tegula and the apex of the wing. Stacking pictures were made using the set-up as outlined in Brecko et al. (2014) and stacking was done with the Zerene Stacker software (https://zerenesystems.com/cms/home). Male genitalia were macerated for 24–48 hours in a 10% potassium hydroxide (KOH) solution at room temperature. Afterwards, genitalia were transferred to acetic acid for 24 hours and thereafter stored in glycerine. Digital images of genitalia were made with a Leica MZ16 microscope and mounted Leica DFC500 digital camera, using LEICA APPLICATION SUITE (LAS) automontage software (version 3.8). Terminology for the male genitalia follows Cumming and Wood (2017). Literature references are given for original taxon descriptions under each species. For type material, text on identification and location labels is given *ad verbatim*. Text is indicated in quotation marks (“ ”) and each line on the label is separated by a double forward slash (//). Text not present on labels (i.e. collection depository) is given in square brackets ([]). The abbreviation spp. in the figure legends refers to different species.

### DNA barcoding

Procedures for DNA barcoding followed Jordaens et al. (2015). Briefly, genomic DNA was extracted from a single leg using the NucleoSpin Tissue Kit (Macherey-Nagel, Düren), following the manufacturer’s instructions. PCR reactions were undertaken in 25 µl reaction volumes, that contained 1.5 mM MgCl_2_ in 1× PCR buffer (Invitrogen), 0.2 mM of each dNTP, 0.2 µM of each primer and 0.5 units of Taq polymerase (Invitrogen). The DNA barcode fragment of the mitochondrial cytochrome *c* oxidase subunit I (COI) gene was amplified using primer pair LCO1490 and HCO2198 (Folmer et al. 1994). The PCR profile was an initial denaturation step of 5 min at 95 °C, followed by 35 cycles of 45 s at 95 °C, 45 s at an annealing temperature of 45 °C and 1.5 min at 72 °C and ending with a final extension step of 5 min at 72 °C. PCR products were purified using the GFX PCR DNA Purification Kit (GE Healthcare) and diluted in 15 µl of sterile water or using the ExoSap protocol (Invitrogen) following the manufacturer’s instructions. PCR-products were bidirectionally sequenced using the ABI PRISM BigDye Terminator v.3.1 Cycle Sequencing Kit and run on an ABI3130xl Genetic Analyzer. Sequences were assembled in SEQSCAPE v.2.5 (Life Technologies) and inconsistencies were checked by eye on the chromatogram.

For the molecular analysis, we obtained 159 DNA barcodes which were submitted to GenBank under accession numbers MW186259–MW186437 (Suppl. material 1: Table S1). This dataset was complemented with 57 DNA barcodes from Jordaens et al. (2015) (GenBank accession numbers: KR831045–KR831101), 16 unpublished barcodes obtained from CNC and four unpublished barcodes from MZH (see Suppl. material 1: Table S1). Hence, the total *Mesembrius* DNA barcode dataset comprised 236 sequences of 18 species (Fig. 229). For five species (*M. ingratus*, *M. longipilosus* sp. nov., *M. maculifer*, *M. rex* and *M. vockerothi* sp. nov.), no DNA barcodes could be obtained.

A Neighbour-Joining (NJ) tree (Saitou and Nei 1987) was constructed using the K2P model in MEGA v.7 (Kumar et al. 2016) (see Fig. 229) and pairwise *p*-distances (i.e. the proportion of sites at which two sequences differ) within and amongst species were calculated (Suppl. material 2: Table S2). In addition, a Maximum Likelihood (ML) analysis was performed using GARLI v.2.01 (Zwickl 2006), after removing identical sequences with DAMBE v.7 (Xia 2018). Branch support in the NJ-analysis was evaluated using 1,000 bootstrap replicates. For the ML analysis, the dataset was partitioned according to the codon position and the most appropriate model of evolution for each partition was selected using the Akaike Information Criterion in jModelTest v.2 (Guindon and Gascuel 2003; Darriba et al. 2012). These models were the F81+I+G (first position), GTR+I+G (second position) and GTR+G (third position), respectively. Then, GARLI v.2.01 was used to perform the ML analysis (two replicates; 500 bootstrap pseudoreplicates) taking into account the most appropriate models of evolution for each of the three codon positions. In each analysis, *Eristalis tenax* (Linnaeus, 1758) was constrained as the root (GenBank accession number MW186258). Bootstrap values were considered to be meaningful if ≥ 70% (Hillis and Bull 1993).

## Results

### Taxonomy and systematics

#### Mesembrius

Taxon classificationAnimaliaDipteraSyrphidae

Rondani, 1857

4E9E15B3-2A91-5056-B629-586447A8C6FB

Mesembrius Rondani, 1857: 50. Type-species: Helophilus peregrinus Loew, 1846, (by monotypy).
Prionotomyia Bigot, 1883: cxxi. Type-species: Prionotomyia tarsata Bigot, 1883 (by monotypy).
Vadonimyia Séguy, 1951: 16. Type-species: Vadonimyia discophora Séguy, 1951 (by original designation).
Tityusia Hull, 1937: 118. Type-species: Tityusia regulus Hull, 1937 (by original designation).

##### Generic diagnosis.

Afrotropical species of *Mesembrius* s.s. (i.e. excluding representatives of the subgenus Vadonimyia, cf. Introduction) have the following combination of diagnostic characters: postpronotum pilose; compound eye bare (Figs 46–82); wing vein R_4+5_ strongly sinuate; wing vein M_1_ processive distally (Figs 127–150); wing cell r_1_ open (rarely, cell r_1_ is narrowly open as in Fig. 142); thorax with katepimeron conspicuously pilose; and metabasitarsus with basoventral globuliferous setae (e.g. Figs 185, 196).

### Key to the Afrotropical species of *Mesembrius* s.s.

#### Key to males

Note: the males of *M. maculifer* Hull, 1941 and *M. morio* (Bezzi, 1915) are unknown; if the male of *M. maculifer* is similar to the female, then the male would be very different from any other *Mesembrius* male.

**Table:** 

1	Profemur with long, downwardly curved pile in distal half which is at least 1.4× as long as femur width (referred to hereafter as “apical pile brush”) (Figs 151–159)	**2**
–	Profemur lacking apical pile brush, pile less than 1.4× as long as femur width (Figs 6, 9)	**10**
2	Probasitarsus with tuft of black pile (Figs 164, 165: arrow); apical pile brush either dense (individual pili are difficult to discern; Figs 151, 155, 156: arrow) or loose (individual pili easy to discern: Figs 157–159: arrow)	**5**
–	Probasitarsus either without tuft of pile (Fig. 153) or with tuft of orange pile on posterior side (Fig. 163: arrow); apical pile brush dense, i.e. the pile is so dense that individual pili are difficult to discern (Figs 152–154: arrow)	**3**
3	Profemur with apical pile brush dark brown; protibia strongly flattened and with long black pile in proximal half and long yellow-orange pile in distal half; probasitarsus without tuft of orange pile (Fig. 153); metabasitarsus with very long, thick pile on posterior half (Fig. 183: arrow); wing cell r_1_ narrowly open (Fig. 142); eyes holoptic (Fig. 61)	***regulus* (Hull)**
–	Profemur with apical pile brush black (Figs 152, 154: arrow); protibia not strongly flattened and with long black pile on proximal 1/3 (Fig. 163) or with long golden pile over entire length (Fig. 154); probasitarsus with tuft of orange pile (Fig. 163: arrow); metabasitarsus without very long, thick pile on posterior half (Fig. 184); wing cell r_1_ distinctly open (Figs 140, 143); eyes holoptic or dichoptic (Figs 59–62)	**4**
4	Profemur with apical pile brush entirely black, no yellow setae interspersed (Fig. 154: arrow); metafemur ventrally with row of > 10 short, widely spaced black spines in the proximal 2/3 and with denser, short black pile on distal 1/3 (Fig. 184); metatibia with one deep depression on posterior side in proximal 1/4, with a collar of black pile ventrally; eyes holoptic, eye contiguity approximately as long as ocellar triangle (Fig. 62)	***rex* Curran**
–	Profemur with apical pile brush black with some short yellow pile interspersed (Fig. 152: arrow); metafemur without row of short spines, but entirely covered in very short, thick black pile and with longer black pile on distal end (Fig. 185); metatibia with three depressions on posterior side in middle third (Fig. 185: arrows), surrounded with black pile, especially dorsally (Fig. 185); eyes slightly dichoptic, distance between eyes approx. the width of anterior ocellus (Fig. 59)	***perforatus* (Speiser)**
5	Profemur with apical pile brush very dense (individual pili difficult to discern) (Figs 151, 155, 156: arrow); metafemur with black pile on ventroproximal section either few or absent; posterior groove on metatibia, if present, bordered with long black pile (Figs 186, 187)	**6**
–	Profemur, apical pile brush loose (individual pili easy to discern) (Figs 157–159: arrow); metafemur with yellow and black pile on 1/10 to 2/3 of ventroproximal section (e.g. Fig. 189: red arrow); posterior groove on metatibia, if present, not markedly bordered with long black pile (Fig. 190)	**8**
6	Profemur with apical pile brush golden yellow to orange (Fig. 151: arrow); lateral sides of abdomen with long yellow and shorter black pile; protarsus chocolate-brown (Fig. 151); metafemur with longer, golden pile at the ventroproximal end, with series of minute, black spines in the ventroproximal section (Fig. 186) and a few long, black setulae in the middle section (Fig. 186: arrow); metatibia with a shallow groove in the posterior proximal half that is bordered with long black pile (Fig. 186)	***chapini* Curran**
–	Profemur with apical pile brush black dorsally (Figs 155, 156: black arrow), golden-yellow ventrally (Fig. 156: white arrow); lateral sides of abdomen with long yellow pile only; protarsus at least partly orange (Figs 155, 164); metafemur without long golden pile at ventroproximal end; metatibia either without groove (Fig. 188) or with a very deep groove that is bordered by long black pile (Fig. 187)	**7**
7	Protarsus orange (Fig. 164); metafemur with long yellow and shorter black pile ventrally; metatibia with a deep groove in the posterior proximal half which is bordered by long black pile (Fig. 187); mesotibia unmodified (Fig. 176)	***sulcus* Jordaens, Goergen & De Meyer, sp. nov.**
–	Probasitarsus black in anterior half, orange in posterior half, protarsi 2–4 black, protarsus 5 lighter with darkened tips (Fig. 165); metafemur long yellow pilose, with few, long black pile interspersed ventrally; metatibia without a deep groove in the posterior proximal half (Fig. 188); mesotibia with proximal half strongly compressed (Fig. 177: arrow)	***tibialis* Jordaens, Goergen & De Meyer, sp. nov.**
8	Profemur with apical pile brush entirely black (Fig. 159: arrow); metafemur with long yellow and shorter black pile on proximal 1/3 (Fig. 189: red arrow); metatibia ventrally with a low, rounded swelling (Fig. 189: black arrow)	***tarsatus* (Bigot)**
–	Profemur with apical pile brush either yellowish with some black pile interspersed (Fig. 158: arrow) or black dorsally and yellow ventrally (Fig. 157: arrow); metafemur either with black pile restricted to proximal 1/10 or black pile more extensive on ventroproximal 2/3 (Fig. 191); metatibia either strongly dorsoventrally compressed in middle 1/3 (Fig. 191) or with one deep groove (Fig. 190: arrow)	**9**
9	Profemur with apical pile brush yellowish with some long black pile interspersed (Fig. 158: arrow); metafemur with long black pile in proximal 1/10; metatibia with deep groove in proximal half of posterior side (Fig. 190: arrow) which is, especially dorsally, bordered by short, curved black pile	***ingratus* (Loew)**
–	Profemur with apical pile brush black dorsally, yellow ventrally (Fig. 157: arrow); metafemur with long, black pile in posteroventral proximal 1/2 and thick, black pile at distal end (Fig. 191); metatibia curved, strongly dorsoventrally compressed in middle 1/3 (Fig. 191)	***arcuatus* Jordaens, Goergen & De Meyer, sp. nov.**
10	Face with ground colour black (Figs 10, 15), but often strongly white pilose and white pollinose in frontal view (Figs 53, 58)	**11**
–	Face with ground colour white to yellow, with black medial vitta (e.g. Figs 47–50)	**12**
11	Metafemur with dense, thick black pile on proximal 1/5 (Fig. 192: arrow); scutum with one pair of vittae; tergite II with a pair of more or less triangular yellow-orange maculae; tergite III with a pair of semi-circular yellow-orange maculae (Fig. 95)	***nigriceps* Curran**
–	Metafemur without dense, thick black pile on proximal 1/5; scutum not vittate, sometimes with a pair of very faint vittae; tergite II with a pair of large, more or less rectangular orange maculae; tergite III either similar as tergite II or entirely orange (Fig. 90)	***cyanipennis* (Bezzi)**
	(Note: we suspect that the male of *M. morio* will key out here.)
12	Probasitarsus whitish to orange, with a lateral lobe bearing an orange pile tuft (Fig. 167: arrow); profemur dorsally flattened (Fig. 166); metabasitarsus with (Fig. 193: arrow) or without (Fig. 194) long lobe (Madagascar only)	***simplicipes* Curran**
–	Probasitarsus orange, brown or black, but never with a lateral lobe and never with an orange pile tuft; profemur either dorsally flattened (Fig. 13) or of a normal shape (Fig. 160); metabasitarsus without long lobe (entire Afrotropical Region)	**13**
13	Metafemur with anteroventral proximal 1/4 bare and posteroventral proximal 1/4 with thick yellow (Fig. 195: red arrow) or black (Fig. 196: red arrow) pile; mesotibia entirely yellow pilose, only with some short black pile at distal end ventrally; only mesobasitarsus with yellow (and black) conspicuous pile (Figs 178, 182: arrow)	**14**
–	Metafemur with anteroventral proximal 1/4 pilose; mesotibia either entirely black pilose or yellow and black pilose (Figs 179, 180), in the latter the yellow pile is either very conspicuous on all mesotarsomeres (Fig. 179) or inconspicuous to absent on all mesotarsomeres (Fig. 181)	**15**
14	Metatibia ventrally with a tooth-like projection on the distal end (Fig. 196: black arrow) and metafemur predominantly black pilose in the posteroventral proximal part (Fig. 196: red arrow)	***caffer* (Loew) (spined morph)**
–	Metatibia ventrally without a tooth-like projection on the distal end and metafemur predominantly yellow pilose in the posteroventral proximal part (Fig. 195: red arrow)	***caffer* (Loew) (nominal morph)**
15	Mesotibia proximal 2/3 dorsally with long, curved yellow pile; distal 1/3 with short black pile on ventrolateral side (Fig. 180) (Madagascar only)	***madagascariensis* Keiser**
–	Mesotibia with pile otherwise, not markedly different between proximal and distal part (Figs 179, 181) (entire Afrotropical Region)	**16**
16	All mesotarsomeres, except the most distal, with conspicuous equally long yellow pile along the posterior edge (Fig. 179: arrow); profemur with yellow pile ventrally, except for a small patch of black pile at extreme proximal end (Fig. 160: arrow)	***capensis* (Macquart)**
–	Mesotarsomeres with either pale yellow pile absent or with pale yellow pile inconspicuous (Fig. 181); profemur with either at most 3–4 black pile at ventral proximal end (Fig. 162) or predominantly black pilose on ventral 1/4; otherwise yellow pilose	**17**
17	Face conical in profile, extending forward ventrally (Fig. 26); scutellum with long yellow and equally dense, very short black pile on entire surface, metafemur and metatibia nearly straight, not markedly curved	***vockerothi* Jordaens, Goergen & De Meyer, sp. nov.**
–	Face not conical (e.g. Figs 14, 22); scutellum yellow pilose, if black pilose in posterior half, then metafemur and metatibia strongly curved	**18**
18	Metafemur and metatibia strongly curved (Fig. 197) (especially visible in posterior view); fourth abdominal segment entirely yellowish pilose with a large patch of very light appressed pile on either side (Fig. 102); scutellum rarely with some very short black pile amongst the longer yellow pile in the posterior half	***strigilatus* (Bezzi)**
–	Metafemur and metatibia not markedly curved (posterior view) (Fig. 198); fourth abdominal segment without patch of very light appressed pile on either side (Figs 89, 92, 94, 100); scutellum yellow pilose only	**19**
19	Metafemur with two areas of dense, conspicuous black pile in the posteroventral section (Fig. 198): a brush-like tuft of black pile over the entire width on the ventral side near the proximal end and, perpendicular to this band, a band of mostly black pile on the posteroventral side	***minor* (Bezzi)**
–	Metafemur with pile distribution otherwise	**20**
20	Profemur with conspicuous thick black pile amongst the yellow pile on ventral proximal 1/4; metafemur with conspicuous black pile amongst the yellow pile on ventral proximal 1/5; metatibia with a tuft of longer, black pile on posteroventral proximal end (Fig. 199: arrow); metabasitarsus almost as long as metatibia (Fig. 199)	***copelandi* Jordaens, Goergen & De Meyer, sp. nov.**
–	Profemur with, at most, some thin black pile amongst the yellow pile on ventral proximal 1/4 (Fig. 162); metafemur with black pile not concentrated in proximal 1/5; metatibia without a tuft of longer, black pile on posteroventral proximal end; metabasitarsus at most 1/2 length of metatibia (Fig. 200)	**21**
21	Profemur without long, black pile on basoventral section (Fig. 161); metafemur with a band of short, thick black pile on the posteroventral side. Maculae on tergite II rectangular; tergite II yellow and black pilose (Fig. 100); male eyes, distance between eyes approx. 1/2 width of anterior ocellus (Fig. 63)	***senegalensis* (Macquart)**
–	Profemur with a few long, black pile on basoventral section (Fig. 162: arrows); metafemur with some shorter, thicker black pile on the ventral side, except on the extreme proximal end (the black and yellow pile are equally long). Maculae on tergite II rounded (Fig. 92); tergite II yellow pilose; male eyes, distance between eyes approx. the width of anterior ocellus (Fig. 55)	***longipilosus* Jordaens, Goergen & De Meyer, sp. nov.**

#### Key to females

Note: the females of *M. arcuatus* sp. nov., *M. ingratus*, *M. longipilosus* sp. nov., *M. nigriceps*, *M. perforatus* and *M. tibialis* sp. nov. are unknown.

**Table:** 

1	Thorax and abdomen reddish-brown (Fig. 33); second abdominal tergite with one pair of cream-coloured slender maculae (Fig. 114); frons, dark brown pilose (Madagascar only)	***maculifer* Hull**
–	Thorax and abdomen dark brown to black; second abdominal tergite either with one pair of yellow-orange maculae (e.g. Fig. 109) or fascia (e.g. Fig. 108) or entirely black (e.g. Fig. 117); frons either black pilose (e.g. Fig. 72) or black and white pilose (e.g. Fig. 78) (entire Afrotropical Region)	**2**
2	Face with ground colour black (but often strong white pilose and pollinose) (Figs 71, 74); wing markedly darker in anterior half (Figs 132, 138)	3
–	Face with ground colour white to yellow (e.g. Figs 70, 72, 75), with black medial vitta; wing not markedly darker in anterior half (e.g. Figs 127–129)	**4**
3	Abdomen entirely black (Fig. 117)	***morio* (Bezzi)**
–	Abdominal tergite II with pair of large orange maculae (Figs 112, 113), other tergites to a various extent orange	***cyanipennis* (Bezzi)**
	(Note: we suspect that the female of *M. nigriceps* will key out here).	
4	Abdomen (almost) black, but with tergites II and III with a pair of vague, lateral maculae (Figs 115, 118) (Madagascar only)	**5**
–	Abdomen yellow or orange and black, with a pair of lateral maculae or vittae on tergites II and III well visible (e.g. Figs 100–111) (entire Afrotropical Region)	**6**
5	Abdomen nearly black (Fig. 115); pro- and mesolegs extensively brown and black (Fig. 34)	***madagascariensis* Keiser**
–	Abdomen very dark but with a pair of vague maculae on tergites II and III (Figs 118, 123); pro- and mesolegs reddish-brown (Figs 37, 41)	***simplicipes* Curran**
6	Frons black pilose on its entire length, except laterally (Figs 72, 76, 77, 80, 81)	**7**
–	Frons pale pilose on ventral half (Figs 70, 78, 82)	**11**
	(Note: we suspect that the female of *M. arcuatus* sp. nov., *M. ingratus*, *M. longipilosus* sp. nov., *M. perforatus* and *M. tibialis* sp. nov. will key out here).	
7	All legs black, except for protarsus which is reddish-brown (Fig. 168); pro- and mesotibia without black pile; mesofemur without black pile on posterior side; metafemur without small ventral swelling in the middle	**8**
–	Legs, inclusive protarsus, very dark (Fig. 169), but especially the tibiae yellow-brown to chocolate-brown; pro- and mesotibia with black pile; mesofemur either with or without black pile on posterior side; metafemur either with or without small ventral swelling in the middle	**9**
8	Tergite II with pair of small yellow-orange maculae, laterally only reaching to halfway tergal length, medially very narrow, pointed; tergite III, pair of anterolateral yellow-orange maculae diffuse (Fig. 124)	***sulcus* Jordaens, Goergen & De Meyer, sp. nov.**
–	Tergite II with pair of large yellow-orange maculae, laterally almost reaching tergal posterior end, medially rounded; tergite III, pair of anterolateral yellow-orange maculae clear (Fig. 125)	***tarsatus* (Bigot)**
9	Protibia with very conspicuous black pile over its entire length; pile on posterior side of mesotibia black on distal half, yellow on proximal half; metafemur without a ventral swelling in the middle (Fig. 201); protarsus dark brown to black (Fig. 169); sternite I and II entirely white, rarely with darkened medial area; wing cell r_1_ distinctly open (as in Fig. 130)	***chapini* Curran**
–	Protibia either with inconspicuous black pile or black pile restricted to distal half; pile on posterior side of mesotibia black at most in 1/4 of distal end, otherwise yellow; metafemur with a small ventral swelling in the middle (Fig. 202: arrow); protarsus yellow-brown to chocolate-brown (Figs 170, 171); sternite I either white or black, sternite II white with a medial darker area; cell r_1_ open (as in Fig. 143) or nearly closed (as in Fig. 142)	**10**
10	Pro- and mesotibia without black pile ventrally (Fig. 170); protarsus chocolate-brown, concolourous with protibia; wing cell r_1_ distinctly open (Fig. 143)	***rex* Curran**
–	Pro- and mesotibia with black pile ventrally (Fig. 171: arrow); protarsus orange-brown, lighter than distal part of protibia; wing cell r_1_ nearly closed (Fig. 142)	***regulus* (Hull)**
11	Tergite II with yellow fascia (Figs 108, 110); if with a vague medial black marking, then posterior black marking never well-developed	**12**
–	Tergite II with a pair of yellow maculae (Figs 107, 116, 121, 123, 126); if medial black marking vague, then posterior black marking well-developed	**13**
12	Mesotibia with black pile either absent or very inconspicuous, but with a few thick, black spines at distal ventral end	***capensis* (Macquart)**
–	Mesotibia with black pile on mesotibia conspicuous in ventral distal half, without thick, black spines at distal end	***caffer* (Loew) (spined morph)**
13	Pro- and metafemur, as well as pro- and metatibia yellow-brown with distal half somewhat darkened, dorsally for a large part covered with strongly contrasting setae-like black pile (Fig. 174); abdomen with central and posterior black markings strongly reduced because of strong white pollinosity which is covered by uniform black, setae-like pilosity (Fig. 121); metafemur light brown without a marked ventral swelling (Fig. 200)	***senegalensis* (Macquart)**
–	Pro- and metafemur dark brown to black with distal end yellow-orange to orange-brown; pro- and metatibia yellow-orange to orange-brown in proximal half, dark brown to black in distal half (Fig. 175); abdomen with clear central and posterior black marking (Figs 107, 109, 116, 126); metafemur dark brown to black, in the middle either without (Fig. 203) or with a marked ventral swelling (Fig. 204: arrow)	**14**
14	Metafemur with clear ventral swelling on middle (Fig. 204: arrow); second abdominal tergite with black posterior marking that does not extend to the lateral margins and approx. equal in size to the anterior black marking (Fig. 116); pro- and mesotarsi brown with a darkened medial part, except in basitarsus (Fig. 173)	***minor* (Bezzi)**
–	Metafemur without ventral swelling on middle (Fig. 203); second abdominal tergite with black posterior marking that extends to the lateral margins and larger than anterior black marking (Figs 107, 109, 126); pro- and mesotarsi uniformly dark brown (as in Fig. 172)	**15**
15	Face markedly produced downward (Figs 45, 82); posteroventral side of metafemur with short black setae on distal 1/2 to 1/3	***vockerothi* Jordaens, Goergen & De Meyer, sp. nov.**
–	Face not markedly produced downwards (Figs 27, 42); posteroventral side of metafemur with short black setae only at distal 1/6	**16**
16	Mesofemur with very few, short black pile on distal end ventrally	***caffer* (Loew) (nominal morph)**
–	Mesofemur with long black pile ventrally, especially on distal half	***strigilatus* (Bezzi)**

### Species account

#### Mesembrius arcuatus

Taxon classificationAnimaliaDipteraSyrphidae

Jordaens, Goergen & De Meyer sp. nov.

E0399246-B030-5848-9FF0-1EE8B740D122

http://zoobank.org/BBA3D30D-32BC-463D-A4E3-0F515798965E

**Figures 3, 4.**
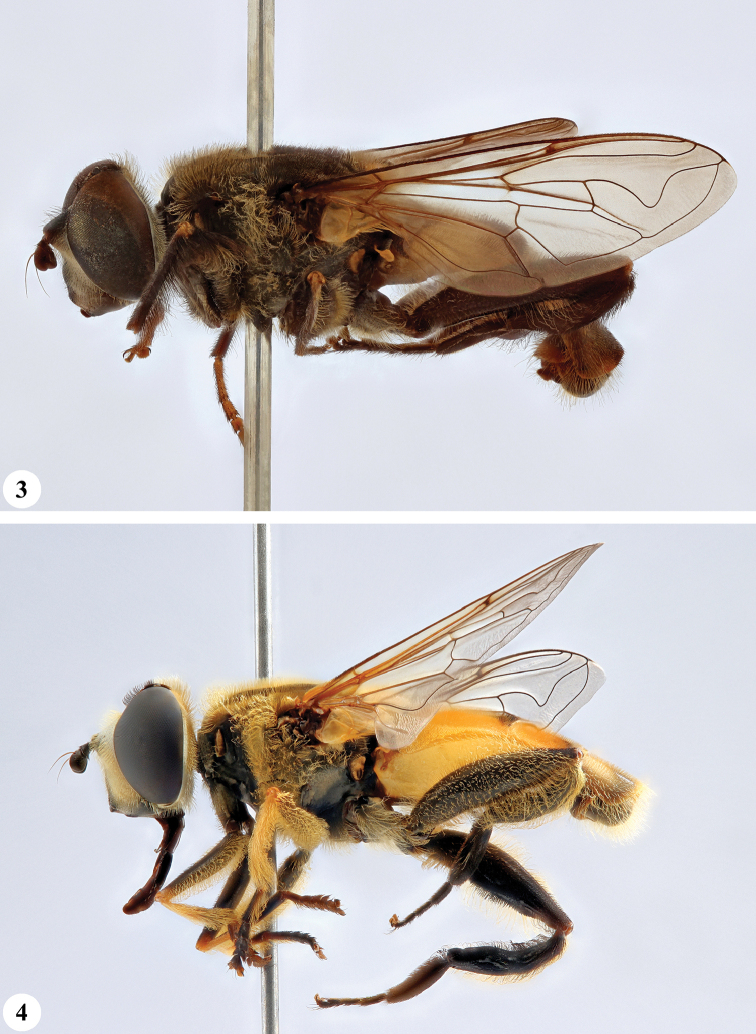
*Mesembrius* spp., habitus, lateral view **3***M. arcuatus* sp. nov. (♂) **4***M. caffer* (Loew) (nominal morph) (♂).

Figs 3 , 46 , 83 , 127 , 157 , 191 , 205

##### Differential diagnosis.

The male of *Mesembrius arcuatus* sp. nov. is holoptic, has a profemur with a loose, black apical pile brush and a strongly curved metatibia which is dorsoventrally compressed in the middle third. It can be distinguished from any other species by the apical pile brush of the profemur which is loose and black dorsally and yellow ventrally (yellowish with some black pile interspersed in *M. ingratus*; black in *M. tarsatus*) and by the strongly compressed metatibia (with deep groove in *M. ingratus*; with a rounded swelling in *M. tarsatus*). The female is unknown.

##### Examined material.

*Mesembrius arcuatus* Jordaens, Goergen & De Meyer: Holotype, male, “HOLOTYPUS” “Entebbe, // Uganda.//21.8.11. // C.C. Gowdey.//1912-100.” “Mesembrius arcuatus // Det. K. Jordaens, 2019” “NHMUK 010369965” [NHMUK].

***Paratypes*:** Uganda • 1♂; Entebbe; 11 Aug 1911; C.C. Gowdey leg.; NHMUK • 1♂; Entebbe; 14 Aug 1911; C.C. Gowdey leg.; NHMUK • 2♂♂; Entebbe; 14 Aug 1911; C.C. Gowdey leg.; NHMUK • 2♂♂, Entebbe; 16 Aug 1911; C.C. Gowdey leg.; NHMUK • 1♂; Entebbe; 17 Aug 1911; C.C. Gowdey leg.; NHMUK • 3♂♂; Entebbe; 21 Aug 1911; C.C. Gowdey leg.; NHMUK • 1♂; Entebbe; 3–4 Dec 1912; C.C. Gowdey leg.; NHMUK • 1♂; Entebbe; 13 Nov 1912; C.C. Gowdey leg.; NHMUK • 2♂♂; Entebbe; 16 Oct 1912; C.C. Gowdey leg.; NHMUK • 5♂♂; Entebbe; 1–11 Sep 1911; S.A. Neave leg.; NHMUK • 1♂; N.E. Side of Lake Albert; 1906; A. Hodges leg.; NHMUK • 1♂; near Entebbe; 5 Mar 1972; H. Falke leg.; CNC • 1♂; near Entebbe; 1–14 Feb 1973; H. Falke leg.; CNC • 1♂; ?Kanue; 3 May 1911; collector unknown; NHMUK • 1♂; Mbarara; 29 May 1911; collector unknown; NHMUK; 1♂; Central Region, Wakisa District, Mabamba Swamps; 16 Dec 2018; X. Mengual; ZFMK • 2♂♂, Central Region, Wakisa District, Mabamba Swamps; 16 Dec 2018; M. Reemer leg.; RMNH • 1♂; Central Region, Wakisa District, Mabamba Swamps 1♂; 16 Dec 2018; K. Jordaens leg.; KMMA.

##### Description male

**(Fig. 3).** Body length: 13.7–14.2 mm. Wing length: 9.8–10.4 mm.

***Head*** (Fig. 46). Eyes bare; holoptic, length of eye contiguity equal to approx. the length of ocellar triangle. Face dark with dark medial vitta; white pilose; white pollinose. Vertical triangle black pilose; white pollinose in dorsal half. Ocellar triangle dark; black pilose; white pollinose; distance between lateral ocellus and eye margin 1/2 width of ocellus. Occiput yellow; white pilose; yellow and white pollinose. Frontal triangle orange-brown; white pilose. Frontal prominence shiny black; black pilose. Antenna dark brown to black; postpedicel white pollinose; antennal arista reddish-brown.

***Thorax*.** Scutum black with a pair of dorsal, well-demarcated grey pollinose vittae; yellow and black pilose. Scutellum uniformly light yellow-brown; yellow pilose throughout, black pilose on posterior 2/3.

***Legs*.** All femora and tibiae with long, loose, yellow pile and, especially at distal end, loose, black pile. Proleg (Fig. 157): Femur dark brown to black; with a loose, apical pile brush which is black pilose dorsally, yellow pilose ventrally; with long, yellow pile posterodorsally and with shorter, black pile anterodorsally and anteroventrally. Tibia with long, black pile, except dorsally. Basitarsus orange-brown; with long, black pile posteriorly. Other tarsi orange-brown; yellow and black pilose dorsally; orange pilose ventrally. Mesoleg: Dark brown to black, except for tarsi which are reddish-brown; black pilose dorsally, orange pilose ventrally. Metaleg (Fig. 191): Femur dark brown to black; very slender; covered with long, thin yellow pile, except on ventral side which is almost bare; with shorter, black pile on posteroventral proximal 2/3; with a series of thick, black setulae at distal 1/3; with thick, black pile at distal end. Tibia curved; strongly dorsoventrally compressed in middle 1/3; with long, yellow and black pile, except in the flattened posterior section. Tarsi yellow and black pilose, except ventrally where orange pilose.

***Wing*** (Fig. 127). Entire wing uniformly microtrichose.

***Abdomen*** (Fig. 83). Tergite II with a pair of very large, yellow triangular to rounded maculae; yellow pilose; black markings hourglass-shaped; posterior black marking equal in size or somewhat narrower than anterior black marking, with a medial white pollinose area; posterior black marking with black pile that posterolaterally extends into the yellow maculae. Tergite III with yellow-orange fascia and a large, black and strongly white pollinose triangular marking; pile short, stiff and black, except on the lateral sides where it is longer, thinner and yellow. Tergite IV strongly white pollinose anteriorly, with a large posterior, rounded, white pollinose black marking; pile short, thick and black, except on the lateral sides where it is longer, thinner and yellow.

***Genitalia*** (Fig. 205). Epandrium: Dorsal lobe of surstylus short and stout, with a very long, sharp expansion on distal end; short black spinose on apex and long brown pilose on dorsal surface. Ventral lobe of surstylus strongly convex; bare.

##### Female.

Unknown.

##### Distribution.

Uganda.

##### Comments.

This is a new species to the Afrotropical Region and only collected from Uganda. The female remains unknown, despite the fact that 28 males were collected or encountered in various collections.

##### Etymology.

The specific epithet *arcuatus* (Latin) means bent like a bow and was chosen with reference to the strongly curved metatibia. It is to be treated as an adjective (nominative singular masculine).

#### Mesembrius caffer

Taxon classificationAnimaliaDipteraSyrphidae

(Loew, 1858)

243A5D3A-DB20-5ECF-9714-BB61D130610A

Figs 1 , 4 , 5–6 , 27–28 , 29 , 47–49 , 84–86 , 107–109 , 128 , 175 , 178 , 182 , 195 , 196 , 206–208

Helophilus caffer Loew, 1858: 380.
Helophilus caffer – Loew (1860): 384 – Karsch (1888): 381 – Hervé-Bazin (1914a): 297.
Helophilus (Tubifera) caffer – Hervé-Bazin (1914b): 103.
Tubifera caffra – Kertész (1910): 250.
Mesembrius caffer – Smith and Vockeroth (1980): 504.
Mesembrius mediopectinatus Szilády, 1942: 97. Syn. by Smith and Vockeroth (1980): 504.
Mesembrius ctenifera Hull, 1941: 333. syn. nov.
Mesembrius ctenifera – Keiser (1971): 261.
Mesembrius ctenifer – Smith and Vockeroth (1980): 504.

##### Differential diagnosis.

*Mesembrius caffer* males lack an apical pile brush on the profemur and have an unmodified metatibia. The metafemur is entirely covered with long, thin yellow pile, but lacks pile in the anteroventral and ventral proximal 1/4. The posteroventral proximal 1/4 has a comb of long yellow or black pile and the remainder of the posteroventral side has a comb of shorter, black pile. The maculae on tergite II are very large and rounded so that black markings have an hourglass shape; the posterior black marking is narrower than the anterior black marking. It can be distinguished from any other male by the bare anteroventral proximal area of the metafemur and the thick comb of yellow or black pile on the proximal end posteroventrally. Females have a frons which is pale pilose on the ventral half. Females of the spined morph (see description below) differ from females of all other species with pale pile on the ventral half of the frons (except from *M. capensis*) in tergite II which has a yellow fascia (a pair of yellow maculae in other species). It differs from the female of *M. capensis* in the mesotibia which has conspicuous black pile in the ventral distal half (inconspicuous in *M. capensis*) and the absence of thick, black spines at the distal ventral end (present in *M. capensis*). Females of the nominal morph have a pair of yellow maculae on tergite II (fascia in the spined morph of *M. caffer* and in *M. capensis*). Pro- and mesofemur are dark brown to black (yellow-brown in *M. senegalensis*), the metafemur lacks a ventral medial swelling (present in *M. minor*), the face is not markedly produced downwards (produced downwards in *M. vockerothi* sp. nov.) and the mesofemur has very sparse and short black pile on the distal end ventrally (long black pile in *M. strigilatus*).

##### Examined material.

*Helophilus caffer* Loew: Lectotype (hereby designated), male, “Helophilus // caffer // ♂” “206” “207” “Loan // 575/99” “NHRS-BYWS // 000002618” [NRMS]. *Helophilus caffer* Loew: Paralectotype, female, “Helophilus // caffer // ♀” “206” “Loan // 575/99” “NHRS-BYWS // 000002617” [NRMS].

*Mesembrius ctenifera* Hull: Holotype, male, “Mesembrius // ctenifera // Hull n. sp.” “TYPE 6596 // Mesembrius // ctenifera // F.M. Hull” “Oriental forest // Fanovana Dist. // Fianarantsoa // Madagascar” “1-V, 1937. // (C. Lamberton)” [ANSP].

*Mesembrius ctenifera* Hull: Allotype, female, “Allotype ♀ // Mesembrius // ctenifera // F.M. Hull” “Oriental forest // Fanovana, Dist. // Fianarantsoa // Madagascar” [ANSP].

##### Other material

**(nominal morph; see below).** Burundi • 1♀; Bujumbura; 20 Feb 2017; G. Goergen leg.; IITA. Cameroon • 1♀; Maroua, Meskina; 23 May 2018; M. Azo’o Ela leg.; MAPC. Democratic Republic of the Congo • 1♂, ?Adu; 8 Apr 1955; collector unknown; KMMA • 1♀; Coquilhatville [= Mbandaka], Bamania, Equateur; 21 Jul 1924; J. Bequaert leg.; KMMA • 1♂; Bambesa, Bas-Uélé; Dec 1933; H.J Brédo leg.; KMMA • 1♀; Barumbu, Léopoldville [= Kinshasa]; 15 Oct 1910; Dr. Bequaert leg.; KMMA • 1♀; Boma; 15 Jul 1920; H. Schouteden leg.; KMMA • 1♀; Bondo, Bas-Uélé; J.J. Rodhain leg.; KMMA • 1♂; Bukama, Haut-Lomami; 28 Mar 1911; J. Bequaert leg.; KMMA • 1♀; Bukama, Haut-Lomami; 24 May 1911; J. Bequaert leg.; KMMA • 1♀; Bunia, Ituri; 1938; P. Lefèvre leg.; KMMA • 3♂♂ 3♀♀; Faradje, Haut-Uélé; Nov 1912; Lang and Chapin leg.; KMMA • 4♂♂; Garamba, Haut-Uélé; Jun–Jul 1912; Lang and Chapin leg.; KMMA • 2♀♀; Kalemie; 1–20 Jan 1919; R. Mayné leg.; KMMA • 1♂; Kasenyi, Lac Albert, Ituri; 15 May 1935; H.J. Brédo leg.; KMMA • 1♀; Kasongo, Maniema; date unknown; Dr. Pons leg.; KMMA • 1♂; Kasonsero, Ituri; 17 Jul 1914, J. Bequaert; KMMA • 1♂ 1♀; Stanleyville [= Kisangani], Tshopo; Mar 1915; Lang and Chapin leg.; KMMA • 1♂; Komi, Sankuru; May 1930; J. Ghesquière leg.; KMMA • 1♂; N’Gwese, Lac Kivu; date unknown; Carlier leg.; KMMA • 1♂; Elisabethville [= Lubumbashi], Haut-Katanga; 3 Jun 1920; M. Bequaert leg.; KMMA • 1♀; Elisabethville [= Lubumbashi], Haut-Katanga; Dec 1925; Van Sackeghem leg.; KMMA; 1♂; Elisabethville [= Lubumbashi], Haut-Katanga; Jan1933; M. Bequaert leg.; RMNH • 1♀; Elisabethville [= Lubumbashi], Haut-Katanga; 16 Nov 1921; M. Bequaert leg.; RMNH • 1♀; Elisabethville [= Lubumbashi], Haut-Katanga; 9 May 1920; M. Bequaert leg.; RMNH • 1♂; Faradje, Ituri; 11 Apr 1930; A. Collart leg.; KMMA • 1♀; Malima; 14 Oct 1910; J. Bequaert leg.; KMMA • 1♀; Mayumbe, Luki; 1924; L. Pieters leg.; KMMA • 1♀; Nyangwe, Maniema; 13 Dec 1910; Dr. Bequaert leg.; KMMA • 1♀; Nyangwe, Maniema; 19 Nov 1910; Dr. Bequaert leg.; KMMA • 1♀; Nyangwe, Maniema; 12 Nov 1910; J. Bequaert leg.; KMMA • 1♂ 1♀; Nyangwe, Maniema; Apr–May 1918; R. Mayné leg.; KMMA • 1♂ 1♀, Apr–May 1918, R. Mayné leg.; RMNH • 1♀; Ubundu, Tshopo; 21 Oct 1910; J. Bequaert leg.; KMMA • 1♂; Wombali, Mai–Ndombe; Jul 1913, P. Vanderijst leg.; KMMA • 1♀; unknown locality; 14 Dec 1951; H. De Saeger leg.; KMMA. • 1♂ 1♀; 4 Jan 1951; H. De Saeger leg.; KMMA • 1♀; 30 Oct 1951; H. De Saeger leg.; KMMA. Ethiopia • 1♀; Koka; 14 Jan 1968; J.W. Boyes leg.; CNC • 1♂, N.W. shore of lake Zwai; 3 Nov 1926; H. Scott leg.; NHMUK • 1♂; unknown locality; Nov 1911; R.J. Stordy leg.; NHMUK. Ghana • 1♂; Tamale; Nov 1916; J.J. Simpson leg.; NHMUK. Kenya • 2♂♂; Kabete; 24 May 1916; T.J. Anderson leg.; NHMUK • 4♂♂; Kanyamkago; 13 Jun 1911; J. Pugh leg.; NHMUK • 1♂; Marsabit District, Rendili Njoro; date unknown; C.A. Neave leg.; NHMUK • 1♂; Mbuyuni, Serengetti Plains; 25 May 1916; T.J. Anderson leg.; NHMUK • 1♂; Voi; 8–10 Feb 1912; S.A. Neave leg.; NHMUK • 1♀; Fisherman’s Camp, Naivasha; 14 Mar 1993; M. De Meyer leg.; KMMA • 1♀; Chawia, near Wundanyi; 25 Jan 2017; A. Ssymank leg.; ASPC • 1♀; Ologassai; Apr 1986; J. Muhangani leg.; NMK • 1♀; Rift Valley; 27–29 May 2013; R. Copeland leg.; ICIPE • 1♂ 1♀; Taita Hills; 2017; A. Ssymank leg.; ASPC • 1♂; Turkana, Lothagam; Jul–Aug 1994; A.M. George leg.; NMK. Liberia • Bong 1♂; County, Suakoko; 6 Feb 1988; G.G.M. Schulten leg.; RMNH. Madagascar • 1♀; Andasibe, Périnet; 29 Oct 2010; A. Ssymank leg.; ASPC • 3♂♂; Antananarivo; 28 Feb 2016; G. Goergen leg.; IITA • 2♂♂ 1♀; Antananarivo; 28 Feb 2016; G. Goergen leg.; KMMA • 1♂; Antananarivo; 8 Mar 2016; G. Goergen leg.; KMMA • 1♂; Antananarivo, Tsimbazaza; 7 Feb 1968; J.W. Boyes leg.; CNC • 1♀; Antananarivo, Antananarivo, Tsimbazaza; 6 Nov 1993; M. Hauser leg.; CAS • 1♂; Antananarivo, Antananarivo, Tsimbazaza; 9 Oct 1993; M. Hauser leg.; CAS • 1♂ 1♀; Antananarivo, Tsimbazaza; 16–22 Oct 1993; C. Kassebeer leg.; CNC • 1♂ 1♀; Antananarivo, Tsimbazaza; 16–22 Oct 1993; C. Kassebeer leg.; NMK • 1♀; Fianarantsoa, Mahabo Mananivo, Ampitavananima forest; 17–24 Mar 2007; M. Irwin, F. Parker and R. Harin’Hala leg.; CAS • 1♀; Tananarive; 28–30 Apr 1968; K.M. Guichard leg.; NHMUK • 1♂; Tananarive; 15 Oct 1957; F. Keiser leg.; NMB • 1♀; Tananarive; 20 Oct 1957; F. Keiser leg.; NMB • 1♂ 1♀; Fianarantsoa; Ambodimanga; 8 Aug 1958; F. Keiser leg.; NMB • 1♂; Ambongamaranitra; 20 Jun 1958; F. Keiser leg.; NMB • 2♂♂; Tananarive; 28–30 Apr 1968; K.M. Guichard leg.; NHMUK • 1♀; Tananarive; 9 Sep 1980; J. Stelleman leg.; RMNH. Malawi • 1♂; Blantyre; 25 Apr 1910; J.E.S. Old. leg.; NHMUK • 2♂♂; Chiromo; J.E.S. Old. leg.; NHMUK • 1♂; Cholo; R.C. Wood leg.; NHMUK • 1♂ 2♀♀; Mulanje Mountain Forest Reserve; 12–15 Nov 2016; K. Jordaens leg.; KMMA • 1♂ 6♀♀; Mulanje Mountain; Likhubula; 12–14 Nov 2016; K. Jordaens leg.; KMMA • 3♂♂; N. Malawi; 1916; N.M. Leys leg.; NHMUK • 3♂♂; Ruo; R.C. Wood leg.; NHMUK • 1♂; Chinteche; H.S. Stannus leg.; NHMUK. Mozambique • 3♂♂; Sofala, Gorongosa Park; 20–30 Apr 2015; M. Hauser and A. Runig leg.; CAS • 1♀; Luaba, lower Zambesi; Jun–Jul 1957; P.J. Usher and B. Stuckenberg leg.; CNC • 1♂; Luabo, lower Zambesi; Apr 1958; P.J. Usher leg.; CNC • 1♂; E of Mount Mulanje; 3–7 Oct 1913; S.A. Neave leg.; NHMUK. Nigeria • 1♀; Samaru; 15–22 Jun 1972; P.H. Ward leg.; NHMUK • 1♀; Ibadan; 28 May 1987; G.G.M. Schulten leg.; RMNH. Senegal • 1♂; Dakar; 14 Jan 1945; collector unknown; RMNH. South Africa • 1♀; Mariepskop, Mpumulanga; 20–22 Jan 2017; K. Jordaens leg.; KMMA • 1♂; St. Lucia Bay, Natal; 3 Nov 1959; D.J. Greathead leg.; NHMUK • 2♂♂; KwaZulu-Natal, Durban; 1 Jul 1903; G. Burn leg.; NMSA • 1♂; Rinkarla; date unkown; H. Junod leg.; NMSA. Tanzania • 2♂♂; Bondei; Jan 1986; C.W. Schmidt leg.; MNB • 1♂; Zanzibar; Jan–Feb 1925; H.J. Snell leg.; NHMUK • 5♂♂; Zanzibar, Kizimbani; 15 Jul 1985; G.G.M. Schulten leg.; RMNH • 5♂♂ 3♀♀; Kilanbeo; 1 May 1971; W.S. Bos leg.; RMNH. Togo • 1♂; Kloto Forest; Feb 2017; G. Goergen leg.; KMMA. Uganda • 2♂♂; Budongo, forest near Lake Albert; Apr 1972; E.B. Babyetagara leg.; CNC • 1♂; Entebbe; 3–4 Dec 1912; C.C. Gowdey leg.; NHMUK • 1♂; Entebbe; 12–20 Jan 1912; S.A. Neave leg.; NHMUK • 2♂♂; Ibanda; 23–28 Dec 1972; H. Falke leg.; CNC • 1♂; Kigezi, Kayonza Forest; Sep 1972; H. Falke leg.; CNC • 1♂; Kigezi, Kayonza Forest; Dec 1972; H. Falke leg.; CNC • 1♀; Kitende; 11 Sep 1927; J. Bequaert leg.; KMMA • 1♂; Masindi; 15–19 Dec 1911; S.A. Neave leg.; NHMUK • 1♂; locality unknown; Nov 1904; E.D.W. Greig leg.; NHMUK • 2♂♂ 4♀♀; Western Region, Kamwege District, Kibale Forest; 8 Dec 2018; K. Jordaens; leg. KMMA • 3♂♂ 1♀; Mabamba swamps, Nkima lodge and surroundings; 16 Dec 2018; K. Jordaens leg.; KMMA. Zambia • 1♂ 1♀; Lusaka Province 8.5 km NW Katondwe; 20 Apr 2016; M. Hauser leg.; CSCA. Zimbabwe • 1♀; Chishi Island, Bangweolo; 26 Jun 1908; S.A. Neave leg.; OXUM • 1♂; Chinsali District, mid-Chambezi Valley; 17 Apr 1908; S.A. Neave leg.; OXUM • 1♂; Mirongo; 3 Apr 1908; S.A. Neave leg.; OXUM • 1♂; upper Luangwa Valley; 29 Feb 1908; S.A. Neave leg.; OXUM • 1♂; upper Luangwa Valley; 17 Mar 1908; S.A. Neave leg.; OXUM • 1♂; upper Luangwa Valley; 20 Mar 1908; S.A. Neave leg.; OXUM • 1♂; upper Luangwa Valley; 5–7 Mar 1908; S.A. Neave leg.; OXUM • 1♂; upper Luangwa Valley; 23–24 Mar 1908; S.A. Neave leg.; OXUM.

(Morphotype with conspicuous spine on metatibia; see Variation, comments and discussion). “ARABIA” • 1♂; Abu; date unknown; C.G. Nurse leg.; NHMUK. Benin • 3♂♂ 3♀♀; Calavi; 8 Dec 2013; K. Jordaens and G. Goergen leg.; KMMA • 2♂♂ 3♀♀; 9 Dec 2013; K. Jordaens and G. Goergen leg; KMMA • 1♀; Calavi; Jan 2014; G. Goergen leg.; KMMA • 1 M; Calavi; Mar 2000; G. Goergen leg.; IITA • 1♂; Calavi; 17 Jul 2015; G. Goergen leg.; IITA • 1♂; Calavi; Oct 2015; G. Goergen leg.; IITA • 1♂; Cotonou; Dec 2003; G. Goergen leg.; IITA • 1♂ 2♀♀; Cotonou; 14 Dec 2013; K. Jordaens and G. Goergen leg.; KMMA • 4♀♀; Cotonou; 28 Jan 2016; K. Jordaens and G. Goergen leg.; KMMA • 1♂; Cotonou; 1 May 1989; G.G.M. Schulten leg.; RMNH • 1♂; Lama Forest; 23 Jan 1995; G. Goergen leg.; IITA • 1♂; Lama Forest; 26 Jul 1995; G. Goergen leg.; IITA • 2♀♀; Lama Forest; 23 Jun 1995; G. Goergen leg.; IITA • 1♀; Niaouli; 2 Feb 2014; G. Goergen leg.; IITA • 1♂ 1♀; Pobè; 27 Jan 2016; G. Goergen leg.; IITA • 1♂ 1♀; Porto Novo; 27 Jan 2016; K. Jordaens and G. Goergen leg.; KMMA • 1♂; Sémé; 7 Feb 2016; G. Goergen leg.; KMMA • 1♂; Tanougou Waterfalls; Nov 2016; G. Goergen leg.; KMMA. Cameroon • 20♂ 20♀; Maroua, Meskina; 23 May 2018; M. Azo’o Ela leg.; MAPC • 1♂; Kribi, Lobe Falls; 22 May 2006; A. Ssymank leg.; ASPC • 1♂; Victoria; 5–18 Nov 1975; W. Schacht leg.; RMNH. Ghana • 1♂; Japi; Nov 1915; J.J. Simpson leg.; NHMUK • 1♀; Tamale; Nov 1915; J.J. Simpson leg.; NHMUK • 9♂♂; Tamale; Nov 1916; J.J. Simpson leg.; NHMUK. Malawi • 1♀; Mulanje Mountain; 12–14 Dec 2016; K. Jordaens leg.; KMMA. Mali • 1♂ 2♀♀; Kogoni; Aug 1983; B. Sidibe leg.; NHMUK. Nigeria • 1♂; Samaru; 7–14 Jul 1970; P.H. Ward leg.; NHMUK. South Africa • 1♂; Dukuduku Forest Reserve; 16–17 Jul 1981; J.G.H. Londt and K. Craddock leg.; NMSA. Senegal • 3♀♀; Dassilamé Sérère; 30 Nov 2011; S. Cavaillès leg.; SCPC • 1♀; Dindefelo; 5 Nov 2016; S. Cavaillès and R. Bou leg.; SCPC. Togo • 1♂; Marais d’Asrama; 8 Apr 2008; A. Ssymank leg.; MZH • 1♀; Kloto Forest; Nov 2016; G. Goergen leg.; KMMA. Zambia • 1♂; Chilanga; 8 Sep 1913; R.C. Wood leg.; NHMUK • 1♂; Chilanga; 2 Jan 1914; R.C. Wood leg.; NHMUK.

(Morphotype with low spine on metatibia; see Variation, comments and discussion). Ghana • 9♂♂; Tamale; Nov 1916; J.J. Simpson leg.; NHMUK. Nigeria • 1♂; Samaru; 7–14 Jul 1970; P.H. Ward leg.; NHMUK. Zambia • 1♂; Chilanga; 2 Jan 1914; R.C. Wood leg.; NHMUK.

##### Re-description of male

**Figures 5, 6.**
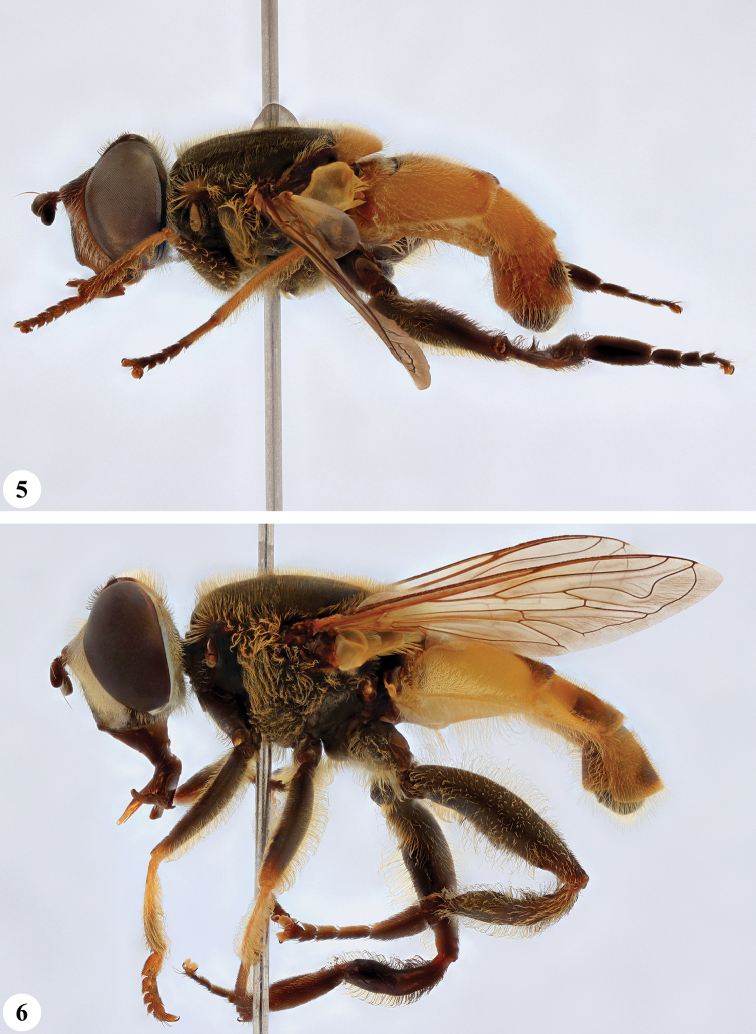
*Mesembrius* spp., habitus, lateral view **5***M. caffer* (Loew) (spined morph) (♂) **6***M. ctenifer* Hull syn. nov. (♀).

**(Figs 4–6).** Body length: 12.1–13.8 mm. Wing length: 8.2–10.0 mm.

***Head*** (Figs 47–49). Eyes bare; slightly dichoptic, distance between eyes approx. the width of ocellus. Face white with dark medial vitta; white pollinose; white pilose. Frontal triangle white; white pilose; white pollinose. Vertical triangle black pilose in ventral half and at ocellar triangle, yellow pilose on vertex; yellow pollinose until just before anterior ocellus; distance between lateral ocellus and eye margin less than 1/2 width of ocellus. Frontal prominence shiny black. Occiput yellow; yellow and white pollinose; yellow pilose. Antenna black, antennal arista reddish-brown.

***Thorax.*** Scutum black with only vague grey pollinose pair of vittae; yellow to rufous pilose. Scutellum uniformly yellow-brown; yellow pilose.

***Legs.*** Proleg: Femur without apical pile brush; black; pile long, yellow, ventrally of equal size over entire length; without black pile. Tibia yellow in proximal half, black in distal half; with long, yellow pile. Tarsi dark brown; black pilose dorsally; orange pilose ventrally with some thick black pile, especially posteroventrally. Mesoleg (Figs 178, 182): Femur dark brown to black; long yellow pilose, but short black pilose on dorsal distal 1/3. Tibia yellow, distally darkened; long yellow pilose, but with black pile interspersed in distal half. Tarsi dark brown; black pilose, with some yellow pile interspersed on basitarsus. Metaleg (Figs 195, 196): Femur dark brown to black; bare in anteroventral and ventral proximal 1/4, posteroventral proximal 1/4 with thicker pile varying from entirely yellow (as in Fig. 195: red arrow) to entirely black (as in Fig. 196: red arrow); otherwise with long and thin yellow pile interspersed with shorter black pile ventrally. Tibia black; long yellow and black pilose; distal end variable: either with (as in Fig. 196; referred to as spined morph) or without (as in Fig. 195; referred to as nominal morph) a tooth-like projection (“spine”) at the ventral distal end; unmodified, but with two shallow depressions on posterior side; long yellow pilose on anterior and dorsal side, black pilose on posterior and ventral side. Basitarsus long, as long as tarsomeres 2+3; dark brown; black pilose. Other tarsi dark brown; black pilose.

**Figures 7, 8.**
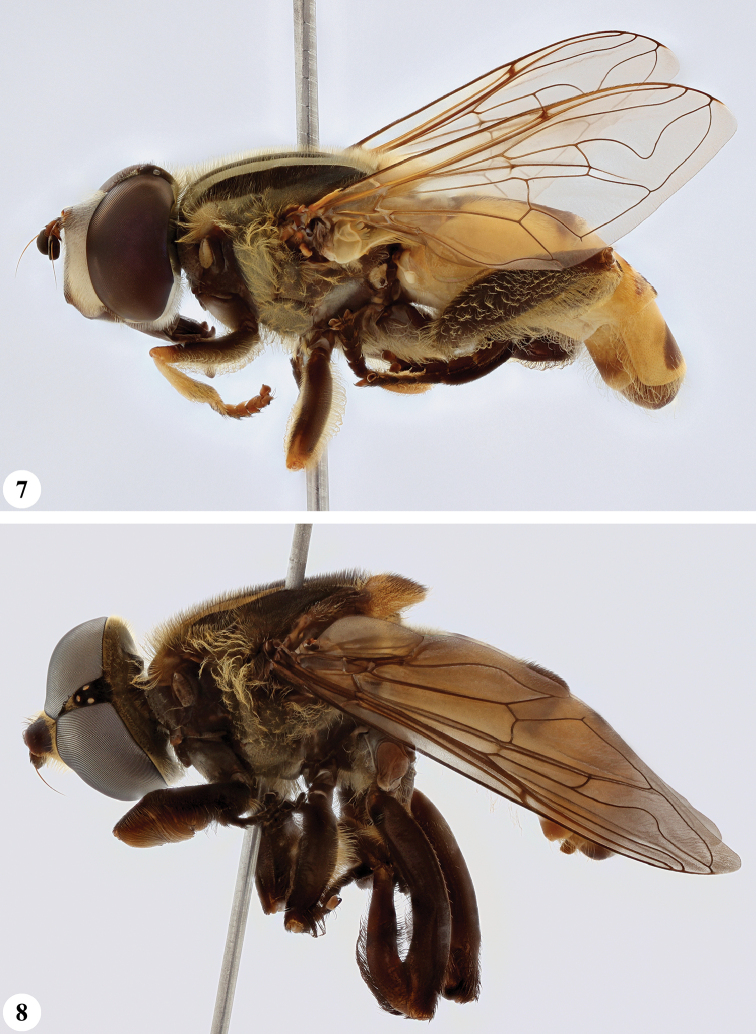
*Mesembrius* spp., habitus, lateral view **7***M. capensis* (Macquart) (♂) **8***M. chapini* Curran (♂).

***Wing*** (Fig. 128). Entire wing uniformly dense microtrichose.

**Figures 9, 10.**
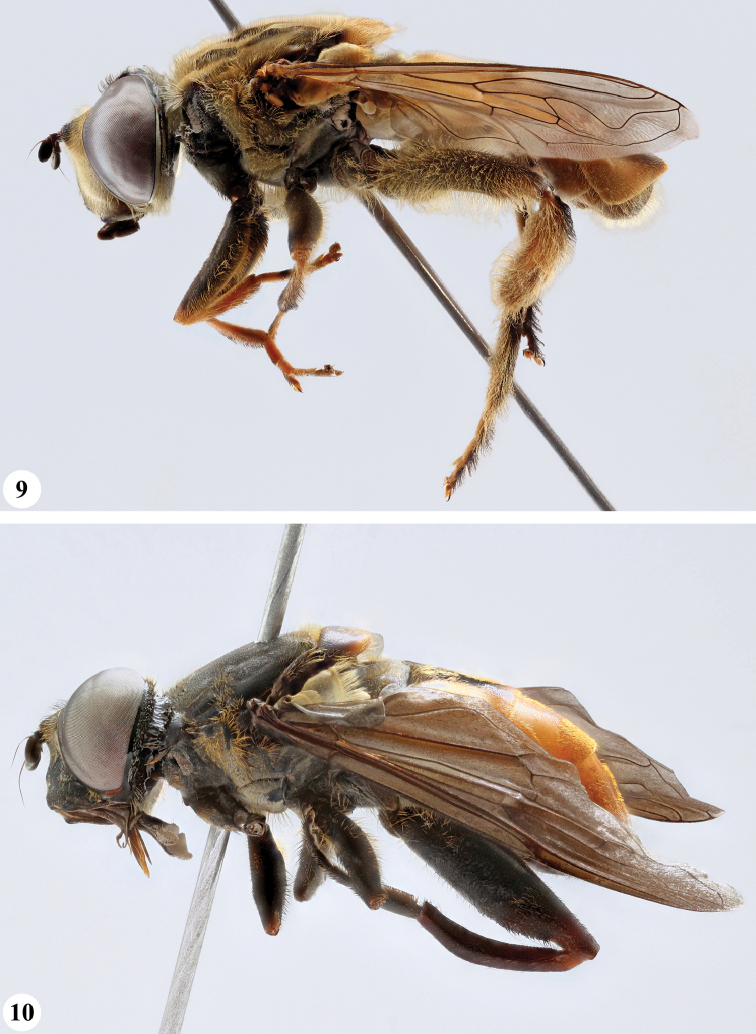
*Mesembrius* spp., habitus, lateral view **9***M. copelandi* sp. nov. (♂). 10. *M. cyanipennis* (Bezzi) (♂).

***Abdomen*** (Figs 84–86). Tergite II with a pair of very large, yellow rounded maculae; black marking hourglass-shaped; posterior black marking sometimes nearly absent (as in Fig. 85), if present, then narrower than anterior black marking; with short, stiff black setulae which do not extend to the lateral margins. Tergite III and IV with yellow fascia of variable size, often occupying almost the entire tergite, but sometimes strongly reduced; Tergite V strongly white pollinose, except for a black medial zone; with short, black stiff pile posteriorly which does not reach the lateral tergal sides.

**Figures 11, 12.**
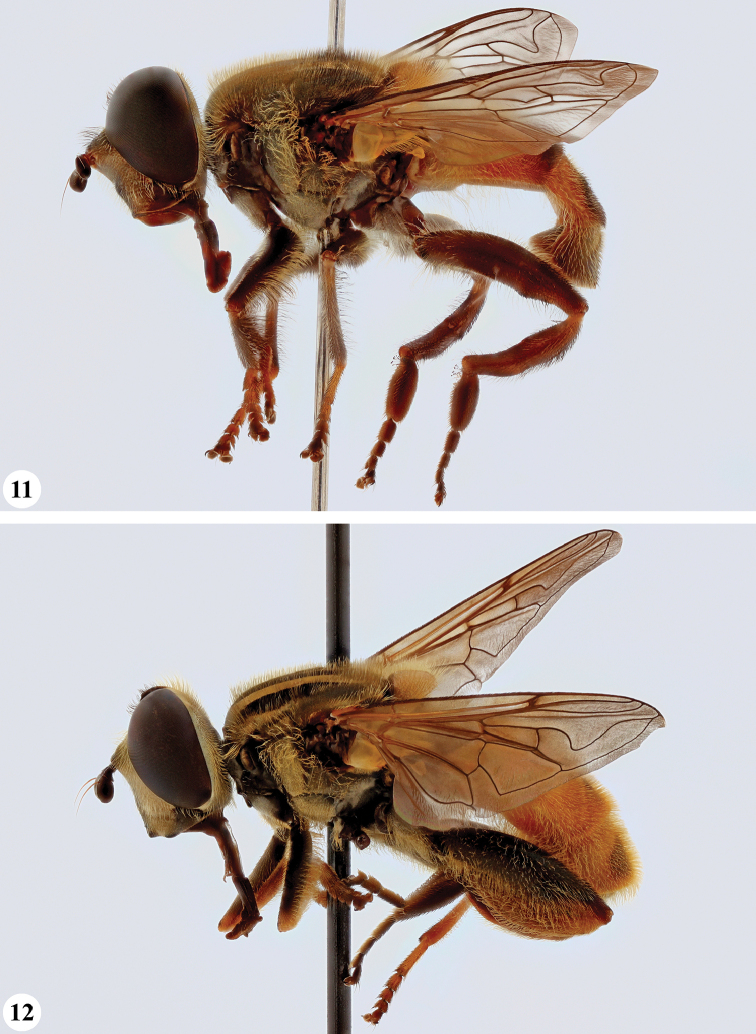
*Mesembrius* spp., habitus, lateral view **11***M. ingratus* (Loew) (♂) **12***M. longipilosus* sp. nov. (♂).

***Genitalia*** (Figs 206–208). Epandrium: Dorsal lobe of surstylus somewhat elongated, broadly rounded; with short, black spines on almost entire surface; dorsally long yellow pilose. Ventral lobe of surstylus straight without conspicuous pilosity.

**Figures 13, 14.**
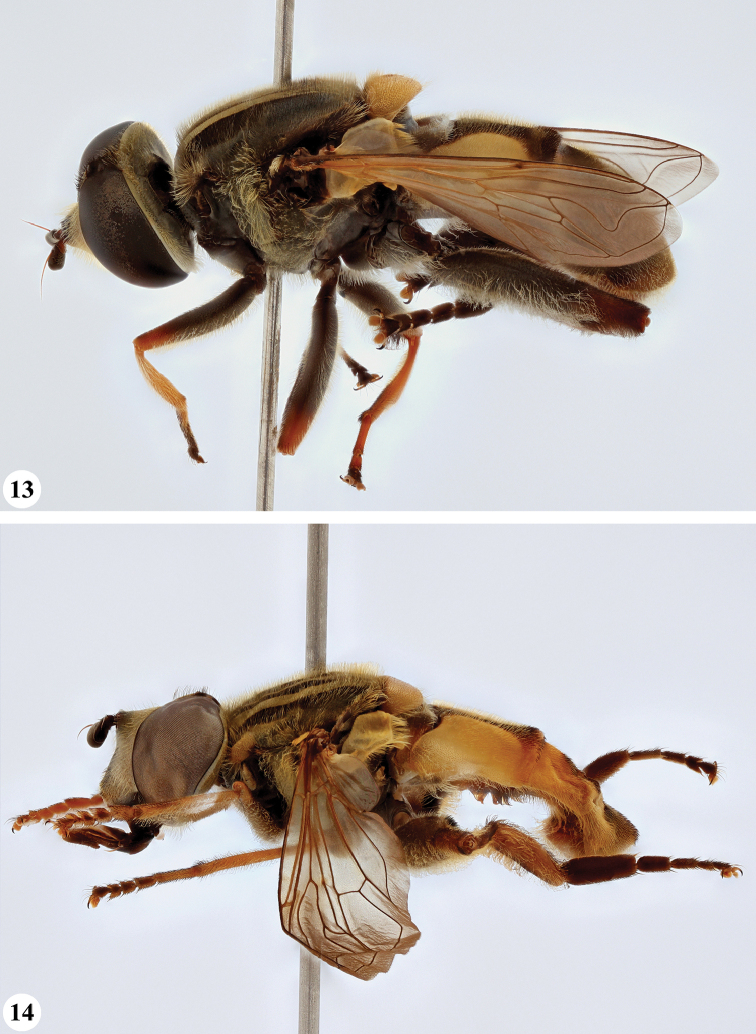
*Mesembrius* spp., habitus, lateral view **13***M. madagascariensis* Keiser (♂) **14***M. minor* (Bezzi) (♂).

##### Variation.

Males of this species are highly variable in their morphology. Some males (spined morph) have a tooth-like projection (spine) on the distal ventral end of the metatibia and the pile on the posterventral distal end of the metafemur is predominantly black. The nominal morph does not have a tooth-like projection (spine) on the distal ventral end of the metatibia and the pile on the posterventral distal end of the metafemur is predominantly yellow. We found 11 males from Zambia, Nigeria and Ghana with a very low spine on the distal ventral end of the metatibia; some of these had a broad, yellow fascia on tergite II, while others had a pair of large, yellow maculae on tergite II.

**Figures 15, 16.**
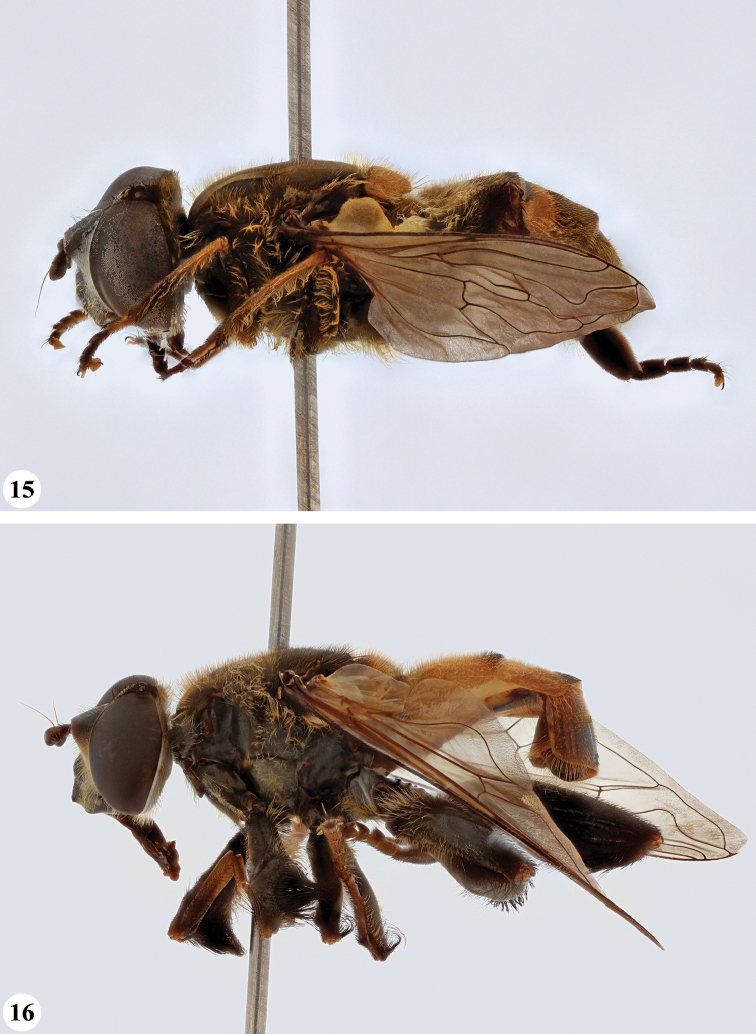
*Mesembrius* spp., habitus, lateral view **15***M. nigriceps* Curran (♂) **16***M. perforatus* (Speiser) (♂).

**Figures 17, 18.**
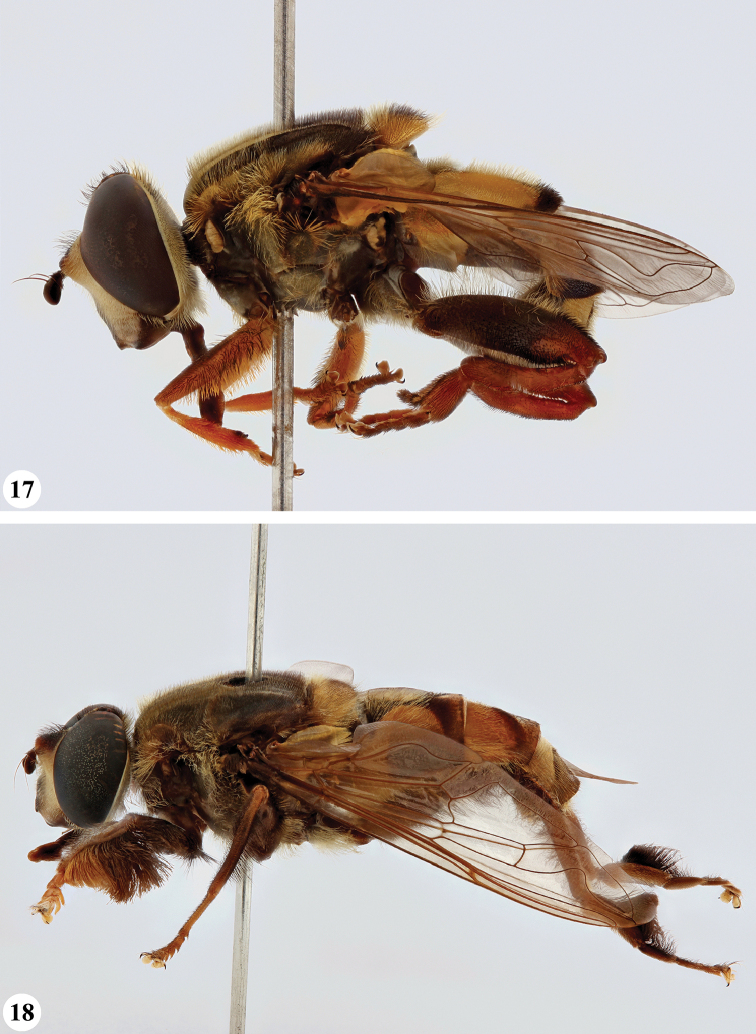
*Mesembrius* spp., habitus, lateral view **17***M. platytarsis* Curran syn. nov. (♂) **18***M. regulus* (Hull) (♂).

**Figures 19, 20.**
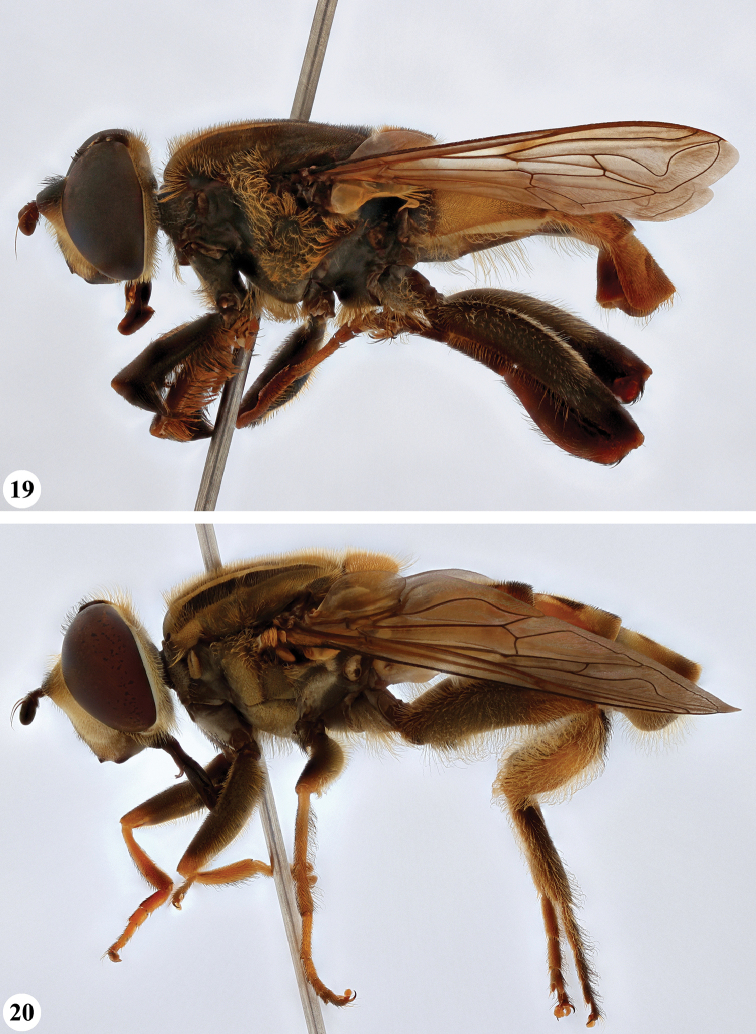
**19***Mesembrius* spp., habitus, lateral view. *M. rex* Curran (♂) **20***M. senegalensis* (Macquart) (♂).

##### Re-description of female

**(Figs 27–29).** Body length: 12.5–15.0 mm. Wing length: 10.1–10.3 mm.

***Head*.** Eyes bare; dichoptic. Face white with dark medial vitta; white pilose, white pollinose. Frons black on dorsal 2/5, yellow-white on ventral 3/5; black pilose on ocellar triangle and just ventrally of ocellar triangle, otherwise white pilose; pollinosity variable, but mostly strongly white pollinose on ventral 3/5. Distance between lateral ocellus and eye margin approx. width of ocellus. Occiput yellow-white; yellow-white pilose; yellow-white pollinose. Frontal prominence shiny black; antenna dark brown to black; antennal arista reddish-brown.

***Thorax.*** Scutum dark brown with one pair of dorsolateral yellow pollinose vittae which are connected posteriorly and with lateral, yellow pollinose vitta; yellow pilose. Scutellum yellow-orange; yellow pilose.

***Legs.*** Proleg: Femur black, distal end orange-brown; yellow pilose, short, black pilose on dorsal distal end. Tibia orange-brown in proximal 2/3, dark brown to black in distal 1/3; yellow pilose with thicker black pile interspersed on ventral side. Tarsi orange-brown; black pilose dorsally, yellow pilose ventrally. Mesoleg: Femur black; yellow-white pilose. Tibia orange-brown in proximal half, darkened in distal half; yellow-white pilose, with some shorter and thicker black pile ventrally, especially in distal half. Tarsi black; black pilose ventrally and dorsally, yellow pilose on posterior and anterior side. Metaleg: Femur black; yellow-white pilose with shorter and thicker black pile on ventral distal half. Tibia black; yellow-white pilose with some black pile interspersed ventrally. Tarsi black; short black pilose dorsally; densely yellow-orange pilose ventrally. In some specimens from Benin, the metafemur and metatibia is brown (but not as light as in *M. senegalensis*) and without the interspersed black pile ventrally.

***Wing.*** Entire wing uniformly microtrichose.

***Abdomen*** (Figs 107–109). Tergite II with a pair of very large, rounded yellow-orange maculae (Figs 107, 109; nominal morph) or with a yellow-orange fascia (Fig. 108; spined morph); yellow pilose on maculae, yellow and black pilose on hourglass-shaped black marking. Tergite III with broad (approx. 3/5 of tergal length in medial section), yellow-orange fascia, with posterior black marking; yellow pilose on fascia, predominantly black pilose on black marking and just anterior of black marking. Tergite IV with narrower yellow-orange fascia (approx. 1/3 of tergal length in medial section); yellow pilose on fascia, yellow and black pilose on black marking. Tergite V black; yellow pilose.

##### Variation.

The females also show substantial variation in their morphology. Females of the spined morph have a broad yellow-orange fascia on tergite II, whereas females of the nominal morph have one pair of large, yellow-orange maculae. Females are variable in the colour of the legs (varying from brown to black) and the abdominal pattern, with some females almost entirely lacking black abdominal markings. Especially the extent of the black markings is variable. In some specimens from Benin, the black markings were very vague so that specimens had an almost yellow-orange abdomen.

##### Distribution.

‘Arabia’, Benin, Burundi, Cameroon, Democratic Republic of the Congo, Ethiopia, Ghana, Kenya, Liberia, Madagascar, Malawi, Mali, Mozambique, Nigeria, Senegal, South Africa, Tanzania, Togo, Uganda, Zambia and Zimbabwe.

##### Comments.

The male syntype of *M. caffer* (Loew, 1858) (designated here as lectotype) and the male holotype of *M. ctenifer* Hull, 1941 are similar. According to Hull (1941), *M. ctenifer* is differentiated from any other *Mesembrius* species by the broad oval metabasitarsus and the black comb-like patch of black setae on the metatibia. Yet, these characters are shared with *M. caffer*, a species which was not mentioned in Hull (1941). DNA barcoding does not differentiate between *M. caffer* and presumed specimens of *M. ctenifer* (i.e. from Madagascar and identified as such by others). Male genital morphology neither differentiates between *M. caffer* and presumed *M. ctenifer* males (compare Fig. 206 with Fig. 207). Thus, we conclude that both are conspecific and that *M. ctenifer* Hull, 1941 is a junior synonym of *M. caffer* (Loew, 1858). According to Hull (1941), a female allotype is deposited at the ANSP, but the specimen could not be found.

Apart from the differences outlined above, males and females of both morphs are similar in morphology and male genitalia of both morphotypes are similar as well (compare Fig. 206 with Fig. 207). DNA barcoding does not differentiate both morphs (both morphotypes even share haplotypes). For the time being, we consider the morphological difference between the nominal and spined morph as intraspecific variation.

The species is widespread in the Afrotropical Region and has also been reported from “Arabia” (a male from Abu collected by C.G. Nurse; see examined material above). Arabia is the peninsular region, together with offshore islands, located in the extreme south-western corner of Asia. It is bounded by the Red Sea on the west and southwest, the Gulf of Aden on the south, the Arabian Sea on the south and southeast and the Gulf of Oman and the Persian Gulf on the east. It includes the modern coastal Arabian states of Yemen, Oman and the United Arab Emirates which, in a zoogeographical context, are part of the Afrotropical Region. However, we could not trace any reference of the collector of the specimen (C.G. Nurse) for the Afrotropical Region. Rather, C.G. Nurse has collected insects on Mount Abu, which is in Rajasthan (India) (see Rosa et al. 2020, for example) and it is thus likely that “Abu” refers to Mount Abu and not a place in Arabia (which are usually also given as binomens, e.g. Abu-Dhabi). The occurrence of the species on the Arabian Peninsula is thus doubtful. In case the specimen is not mislabelled, then it means that *M. caffer* also occurs in India.

#### Mesembrius capensis

Taxon classificationAnimaliaDipteraSyrphidae

(Macquart, 1842)

0A6DC797-C790-5549-9B7E-D02757AC502D

Figs 7 , 30 , 50 , 70 , 87 , 110 , 129 , 160 , 179 , 209

Helophilus capensis Macquart, 1842: 122 (South Africa).
Helophilus capensis – Séguy (1931): 118.
Helophilus (Mesembrius) capensis – Bezzi (1915): 95.
Tubifera capensis – Kertész (1910): 250.
Mesembrius capensis – Curran (1927): 64 – Curran (1939): 10 – Smith and Vockeroth (1980): 504.
Helophilus lagopus Loew, 1860: 386 (South Africa). syn. nov.
Helophilus lagopus – Bezzi (1901): 16.
Helophilus (Mesembrius) lagopus – Bezzi (1915): 95.
Tubifera lagopus – Kertész (1910): 255.
Mesembrius lagopus – Curran (1927): 62 – Curran (1939): 10 – van Doesburg (1955): 355 – Smith and Vockeroth (1980): 504 – De Meyer and Maragia (1993): 3.

##### Differential diagnosis.

*Mesembrius capensis* males lack an apical pile brush on the profemur and have an unmodified metatibia. The basoventral section of the profemur has a patch of black pile. The metafemur is entirely covered with long, thin yellow pile, but with long black pile interspersed in the anteroventral proximal 1/5. The fascia on tergite II is very large with a large anterior and smaller posterior triangular black marking. Males are easily distinguished from any other male by the patch of long black pile on the ventroproximal end of the profemur. Females have a frons which is pale pilose on the ventral half. It differs from females of other species with a pale pilose frons in the ventral half (except from the spined morph of *M. caffer*) in tergite II which has a yellow fascia (a pair of yellow maculae in other species). It differs from the female of the spined morph of *M. caffer* in the mesotibia which lacks or has very inconspicuous black pile, except for a few thick, black spines at the distal ventral end (black pile conspicuous in ventral distal half and without thick black spines at distal ventral end in *M. caffer*).

##### Examined material.

*Helophilus capensis* Macquart: Lectotype (hereby designated), male, “SYNTYPE” “MNHN, Paris // ED6791” “1♂ Helophilus // capensis Macq // C.F. Kassebeer 1999” [MNHN]. *Helophilus capensis* Macquart: Paralectotype, female, “SYNTYPE” “MNHN, Paris // ED6790” “SYNTYPES // Vockeroth ‘69” “afrique // Delalande” [MNHN].

*Helophilus lagopus* Loew: Holotype, female, “Helophilus // lagopus” “208” “Cap. B. // Spei.” “Victo- // rin.” “LECTOTYPUS // Helophilus lagopus // Loew, 1858” “design. Kassebeer 1993” “NHRS-BYWS // 000002620” [NRMS].

##### Other material.

 Angola • 1♂ 1♀; 30 km NE of Duque de Braganza; Nov–Dec. 1957; G.H. Heinrich leg.; NHMUK • 1♀; Duque de Braganza; Nov–Dec 1957; G.H. Heinrich leg.; NHMUK. Botswana • 1♂; Gabarone; 12 Nov 1988; W.H.O. Ernst leg.; RMNH. Democratic Republic of the Congo • 1♀; Sakania; Sep 1931; N.P. Cockerell leg.; NHMUK • 1♂ 1♀; Lualaba; Bunkeya; Oct 1907; S.A. Neave leg.; NHMUK • 1♂; Katanga; Kafubu Mission; Nov 1931; J. Ogilvie leg.; CNC • 1♀; South Kivu; Kalambelembe-Baraka; Jul 1918; R. Mayné leg.; KMMA • 1♀; Kapanga; Lulua; Nov 1928; Walker leg.; KMMA • 1♂ 1♀ Léopoldville [= Kinshasa]; 27 May 1915; Lang and Chapin leg.; KMMA • 1♂; Elisabethville [= Lubumbashi]; 11–17 Sep 1931; A. Mackie leg.; NHMUK • 1♀; Elisabethville [= Lubumbashi]; 23 May 1920; M. Bequaert leg.; KMMA • 1♂ 1♀; Elisabethville [= Lubumbashi]; 1927; M. Bequaert leg.; KMMA • 1♀; Elisabethville [= Lubumbashi]; 12 Nov 1928; M. Bequaert leg.; KMMA • 1♂ 1♀; Elisabethville [= Lubumbashi]; 1 Dec 1929; M. Bequaert leg.; KMMA • 1♂; Elisabethville [= Lubumbashi]; 11–17 Nov 1931; T.D.A. Cockerell leg.; CNC • 1♂ 1♀; Elisabethville [= Lubumbashi]; 9 Sep 1932; De Loose leg. • KMMA • 2♀♀; Elisabethville [= Lubumbashi]; 1 Dec 1929; M. Bequaert leg.; RMNH • 1♂; Elisabethville [= Lubumbashi]; 15 Mar 1928; M. Bequaert leg.; RMNH • 1♂; Elisabethville [= Lubumbashi]; 23 May 1920; M. Bequaert leg.; RMNH • 1♂; Sakania; Nov 1931; V.P. Cockerell leg.; CNC • 1♀; Tumbwe; 16 Nov 1921; M. Bequaert leg.; KMMA • 1♀; locality unknown; 8 Nov 1951; H. De Saeger leg.; KMMA. Eswatini; 1♀; Manzini; 7 May 1991; J.A.W. Lucas leg.; RMNH. Ethiopia • 1♂ 1♀; Errer River; date unknown; G. Kristensen leg.; NHMUK. Ghana • 1♂; Kumasi; 28 Oct 1946; J. Bowden leg.; NMSA. Kenya • 2♂♂ 3♀♀; Nairobi Prov., Kasarani; 10–14 Dec 2016; K. Jordaens and R. Copeland leg.; NMK • 1♀; Nairobi Prov., Kasarani; IV.2014; R. Copeland leg.; ICIPE • 1♀; Central Prov., Kinnyaga, Mucagara Farm; 16–18 Dec 2016; K. Jordaens and R. Copeland leg.; NMK • 2♂♂; Nyeri; X.1948; van Someren leg.; NHMUK • 2♂♂ 2♀♀; Sola District, Lonje Valley, Laikipia escarpment; 12 Sep 1919; T.J. Anderson leg.; NHMUK. Madagascar • 1♀; Antananarivo; 28 Feb 2016; G. Goergen leg.; IITA. Malawi • 1♀; Ruo; date unknown; R.C. Wood leg.; NHMUK. Mozambique • 1♂; Luabo; Lower Zambezi River, Port East Africa; 1 Apr 1958; P. Usher leg.; NMSA • 1♂; Rikatla; date unknown; H. Junod leg.; NMSA. Rwanda • 1♀; Nduga; Mar 1953; P. Basilewsky leg.; KMMA. South Africa • 1♀; Barberton, Aloe Bridge Farm; 22 Oct 2016; A. Vujić et al.; leg.; UNS • 2♂♂; KwaZulu-Natal, Bisley Valley Nature Reserve; 22 Dec 1993; J.G.H. Londt and Craddock leg.; NMSA • 1♂ 1♀; Bontebok National Park; 4 Dec 2016; A. Vujić; S. Radenković; N. Veličković and Z. Petanidou leg.; UNS • 1♂ 1♀; Capland; date unknown; S. Krebs leg.; MNB; 1♂; Western Cape, Cederberg; 17–21 Oct 2017; K. Jordaens leg.; KMMA • 1♂; KwaZulu-Natal, Doonybrook; 10 Oct 2015; Vujić et al. leg.; UNS • 1♀; Eastern Cape, East London; 9 Apr 1922; H.K. Munro leg.; RMNH • 1♂; Eastern Cape, East London; 9 Apr 1922; H.K. Munro leg.; NMSA • 1♂; Hottentot, Holland; 5 Dec 2016; A. Vujić; S.Radenković; N. Veličković and Z. Petanidou leg.; UNS • 1♂; KwaZulu-Natal, Ashburton; 8 Nov 1982; D.A. Barraclough leg.; NMSA • 1♂; KwaZulu-Natal, Barlett Estate, Cato Ridge; 9 Oct 2018; G. Theron leg.; NMSA • 1♂; KwaZulu-Natal, Bishopstown, near Pietermaritzburg; 11 Dec 1982; A. Seymour leg.; NMSA • 2♂♂; KwaZulu-Natal, Bisley Valley Reserve; 22 Dec 1993; J.G.H. Londt leg.; NMSA • 1♂; KwaZulu-Natal, Congelia; 26 Oct 1906; G.F. Leigh leg.; NMSA • 2♂♂; KwaZulu-Natal; Ferncliff; 16 Nov 2018; K. Jordaens leg.; NMSA • 1♂; KwaZulu-Natal, Hudley; date unknown; E. Pinhey leg.; NMSA • 1♂; KwaZulu-Natal, Illovo; 14 Jun 1919; collector unknown; NMSA • 1♂; KwaZulu-Natal, Ingwavuma; 21 Feb 1979; J.G.H. Londt leg.; NMSA • 1♂; KwaZulu-Natal, Kosi Bay Nature Reserve; 30 Nov 1982; B.R. Stuckenberg leg.; NMSA • 1♀; KwaZulu-Natal, Kosi Lake; 22–27 Jan 1967; D. Gilissen leg.; RMNH • 1♂; KwaZulu-Natal, Mkuzi Game Reserve, Nsumu Pan Area; 12 Jan 1994; Natal Museum Staff leg.; NMSA •1♂; KwaZulu-Natal, Mtunzini; 7 Feb 1965; T. Schofield leg.; NMSA • 1♂; KwaZulu-Natal, Port Shepstone, Uvongo; 23 Sep 2005; A. Wilson leg.; NMSA • 1♂; KwaZulu-Natal, Salt Rock; 5 Oct 1991; J.G.H. Londt leg.; NMSA • 2♂♂; KwaZulu-Natal; Zululand, Ndumu Game Reserve; 26 Oct 1972; M.E. Irwin leg.; NMSA • 3♂♂ 4♀♀; KwaZulu-Natal, Howick; 18 Oct 2015; A. Vujić et al. leg.; UNS • 1♀; KwaZulu-Natal, Howick, near Curry’s Post; 14 Feb 2016; A. Vujić; S. Radenković leg.; UNS • 1♂; Western Cape, Keniworth Racecourse Cons. Area; 5 Nov 2014; A. Vujić et al. leg.; UNS • 1♀; Mpumulanga, Mariepskop National Park; 23–24 Jan 2017; K. Jordaens leg.; KMMA • 1♂ 1♀; Mpumulanga, Molele Farm; 28 Jan 2017; K. Jordaens leg.; KMMA • 1♂; Muizenberg, False Bay; 3 Jan 1972; Southern African Expedition leg.; NHMUK • 1♀; KwaZulu-Natal, N. from Pietermaritzburg along Otto’s Bluff; 19 Oct 2015; X. Mengual leg.; ZFMK • 4♂♂ 8♀♀; KwaZulu-Natal, near Howick; 18 Oct 2015; X. Mengual leg.; ZFMK • 1♂; Cape Province, Port Elizabeth; 22–27 Dec 1985; J.G.H. Londt leg.; NMSA • 1♂; Stellenbosch, Delheim winery; 10 Feb 2009; E.M. & L. Laasonen leg.; MZH • 1♂; Western Cape, Cederberg NP; 20 Nov 2011; A. Vujić leg.; MZH • 1♂ 1♀; KwaZulu-Natal, Winterton; 1 Oct 2015; X. Mengual leg.; ZFMK • 1♂; KwaZulu-Natal, Winterton; 27 Sep 2015; A. Vujić et al. leg.; UNS • 1♂ 2♀♀; Zastron; 16 Dec 2016; A. Vujić; S. Radenković; N. Veličković and Z. Petanidou leg.; UNS • 1♂ 1♀; locality and date unknown; S. Krebs leg.; MNB • 1♂; Limpopo Province, Moorddrift; date and collector unknown; NMSA • 1♂; Limpopo Province, Plat River; 1 Jan 1903; V. Judzncka leg.; NMSA • 1♂; Mongosi; May 1916; W.E. Jones leg.; RMNH • 2♂♂; Mpumalanga, Barberton, De Kaap; 18 and 29 Apr 1929; H.K. Munro leg.; NMSA • 2♂♂; Mpumalanga, Lomati River; 7 Nov 1970; B.R. Stuckenberg leg.; NMSA • 1♀; Piet Retief; 15 Mar 1918; Dr. Brauns leg.; RMNH • 1♀; Port Elizabeth; 24 Feb 1922; H.K. Munro leg.; RMNH • 1♂; Western Cape, Knysna; 1 Jan 1910; H. Brauns leg.; NMSA. Togo • 1♀; Kloto Forest; Feb 2005; G. Goergen leg.; IITA. Uganda • 1♂; Entebbe; 23–31 Jan 1973; H. Falke leg.; CNC • 1♀; Entebbe; 1–15 Apr 1983; G.G.M. Schulten leg.; RMNH • 1♂; Ibanda; 23–28 Dec 1972; H. Falke leg.; CNC. Zambia • 1♂; Lake Bangweulu, Mbawala Island; Nov–Dec 1946; collector unknown; NHMUK • 1♂; N. of lake Bangweulu, near Milambo; 20 Oct 1946; collector unknown; NHMUK • 1♂; N. of Lake Bangweulu; N’Sombo; 11 Dec 1946; collector unknown; NHMUK • 1♂; Chilanga; 2 Jan 1914; R.C. Wood leg.; NHMK. Zimbabwe • 1♂; upper Kalungwsisi valley; 10 Sep 1908; S.A. Neave leg.; OXUM • 1♂; Salisbury [= Harare]; date unknown; G.A.K. Marshall leg.; NHMUK • 1♀; Umtali District; 2 Jan 1931; P.A. Sheppard leg.; RMNH • 1♂; N. Vumba; 6 Oct 1963; D. Cookson leg.; NMSA.

##### Re-description male

**(Fig. 7).** Body length: 10.6–14.5 mm. Wing length: 8.1–10.4 mm.

***Head*** (Fig. 50). Eyes bare; slightly dichoptic, distance between eyes approx. the width of ocellus. Face white with dark medial vitta; white pollinose; white pilose. Vertical triangle with black pile in lower half and at ocellar triangle, yellow pile on vertex; yellow pollinose until just before anterior ocellus; distance between lateral ocellus and eye margin 1/2 width of ocellus. Frontal triangle white; white pilose; white pollinose. Frontal prominence shiny black. Occiput yellow; yellow pilose; yellow and white pollinose. Antenna black, antennal arista reddish-brown.

***Thorax.*** Scutum black with, dorsally, pair of well-demarcated yellow vittae and a faint yellow medial line, which are both connected at anterior and posterior parts of scutum; with lateral, yellow vitta; pile rufous. Scutellum uniformly yellow-brown; yellow pilose.

***Legs.*** Proleg (Fig. 160): femur dark brown to black, without apical pile brush; yellow pilose, pile shorter on dorsal side; with a small patch of black pile at posteroventral proximal end. Tibia yellow to orange-brown, long yellow pilose with a few thick black pile at distal end; tarsi orange-brown, black pilose dorsally, yellow-orange pilose ventrally. Mesoleg (Fig. 179): femur as in proleg, but without the posteroventral black pile at proximal end; tibia yellow-orange; with black pile on anterodorsal side; otherwise long yellow pilose. Basitarsus and second tarsal segment orange-brown; black pilose dorsally, long yellow pilose posteriorly. Other tarsi brown; black pilose, with long dark brown pile posteriorly. Metaleg: femur dark brown to black; yellow pilose, with long black pile interspersed at posteroventral proximal half and with some short and thicker black pile at distal end. Tibia dark brown; unmodified; long yellow pilose with sorter, black pile on ventrally and posteriorly, ventral side also with some long, black pile. Tarsi dark brown to black; black pilose dorsally, dark brown and orange pilose ventrally.

***Wing*** (Fig. 129). Entire wing uniformly dense microtrichose.

***Abdomen*** (Fig. 87). Tergite II with a pair of large, yellow triangular maculae; black marking hourglass-shaped; posterior black marking, though sometimes very vague (as in Fig. 87), equal in size or somewhat narrower than anterior black marking. Posterior black part with short, stiff black setulae which to not extend to the lateral margins. Tergite III and IV with yellow fascia of variable size, often occupying almost the entire tergite, but always with black triangular marking posteriorly. Tergite V strongly white pollinose, except for a black medial zone; with short, black stiff pile posteriorly which do not reach the lateral tergal sides, this pile being absent in specimens where the posterior black marking is strongly reduced.

***Genitalia*** (Fig. 209). Epandrium: Dorsal lobe of surstylus elongated, more or less rectangular with upwardly curved apex; with short, black spines in distal half; dorsolaterally with a few longer, black setulae; dorsally and laterally with long, yellow pile. Ventral lobe of surstylus with large expansion ventrally with, on ventral side of the expansion, very long and dense black setulae.

##### Re-description female

**(Fig. 30).** Body length: 9.6–14.4 mm. Wing length: 8.1–10.2 mm.

***Head*** (Fig. 70). Eyes bare; dichoptic. Face yellow-white with dark medial vitta; white pilose, white pollinose. Frons yellow-white; predominantly black pilose on ocellar triangle and just ventrally of ocellar triangle, otherwise white pilose; strongly white pollinose on ventral 3/5. Distance between lateral ocellus and eye margin approx. 1½x width of ocellus. Occiput yellow-white; yellow-white pilose; yellow-white pollinose. Frontal prominence shiny black, orange-brown at distal end; antenna black; antennal arista reddish-brown.

***Thorax.*** Scutum dark brown with one pair of dorsolateral lighter, yellow pollinose vittae which are connected posteriorly; with lateral, yellow pollinose vitta; yellow pilose. Scutellum yellow-orange; yellow pilose.

***Legs.*** Proleg: Femur black, distal end yellow-orange; yellow pilose; white pollinose. Tibia orange, darkened on dorsal distal 1/3; yellow pilose. Tarsi orange-brown to black; black pilose dorsally, yellow pilose ventrally with some thick, black pile ventrally. Mesoleg: Femur black; yellow-white pilose, with some short black spines at distal end ventrally. Tibia orange; yellow pilose, with some long, thick black pile at ventral distal end. Basitarsus orange; yellow pilose, with some long, thick black pile on dorsal distal end and ventrally. Other tarsi black; dorsally short black pilose, ventrally yellow pilose with some large, thick black pile. Metaleg: Femur black; yellow-white pilose with shorter and thicker black pile on ventral distal half; white pollinose. Tibia chocolate-brown; yellow-white pilose with some black pile interspersed ventrally, pile longer on distal ventral end. Tarsi black; short black and yellow pilose dorsally; densely yellow-orange pilose ventrally.

***Wing.*** Entire wing uniformly microtrichose.

***Abdomen*** (Fig. 110). Tergite II with a broad orange fascia, with a narrow black anterior marking and a larger, posterior black marking which, in the medial part, is approx. 1/2 the tergal length; yellow pilose, with some very short and thicker black pile posteriorly; especially the posterior part of the posterior black marking white pollinose. Tergite III with yellow-orange fascia which in the medial part is approx. 2/5 of tergal length; yellow pilose, with some very short and thicker black pile posteriorly; especially in the medial part strongly white pollinose. Tergite IV similar, but entire orange fascia strongly white pollinose and black pile in black marking almost absent. Tergite V black; yellow-white pilose; strongly white pollinose on anterior half, especially on the lateral sides.

##### Distribution.

Angola, Botswana, Democratic Republic of the Congo, Eswatini, Ethiopia, Ghana, Kenya, Madagascar, Malawi, Mozambique, Rwanda, South Africa, Togo, Uganda, Zambia and Zimbabwe.

##### Comments.

The study of the type material shows that *M. lagopus* (Loew, 1860) is morphologically similar to *M. capensis* (Macquart, 1842) and hence, we consider both conspecific, with *M. lagopus* (Loew, 1860) being a junior synonym of *M. capensis* (Macquart, 1842).

#### Mesembrius chapini

Taxon classificationAnimaliaDipteraSyrphidae

Curran, 1939

7220A207-FAF4-5ACB-A7C8-3B7B13D2F214

Figs 8 , 31 , 51 , 72 , 88 , 111 , 130 , 151 , 169 , 186 , 201 , 210

Mesembrius chapini Curran, 1939: 10.
Mesembrius chapini – Smith and Vockeroth (1980): 504.

##### Differential diagnosis.

*Mesembrius chapini* males have a profemur with a thick and dense, golden apical pile brush. The metafemur is long and slender with some long, black thick pile in the ventral middle. The metatibia has a shallow anterior depression in the middle and a deeper depression on the ventral side of the distal end; the ventral side has a carina. The male is easily distinguished by the thick golden yellow to orange apical pile brush of the profemur and the series of minute, black spines in the ventroproximal section of the metafemur. Females have a frons which is black pilose on its entire length, except laterally. It can be distinguished from the female of *M. sulcus* sp. nov. and *M. tarsatus* by the yellow-brown to chocolate-brown tibiae (black in *M. sulcus* sp. nov. and *M. tarsatus*). It differs from the female of *M. rex* and *M. regulus* by the very conspicuous black pile over the entire length of the protibia (absent in *M. rex*; restricted to distal half in *M. regulus*) and by the dark brown to black protarsus (yellow-brown to chocolate-brown in *M. rex* and *M. regulus*). The legs are very dark, but especially the tibiae are yellow-brown to chocolate-brown. The protibia has conspicuous black pile over its entire length. Wing cell r_1_ is distinctly open.

**Figures 21, 22.**
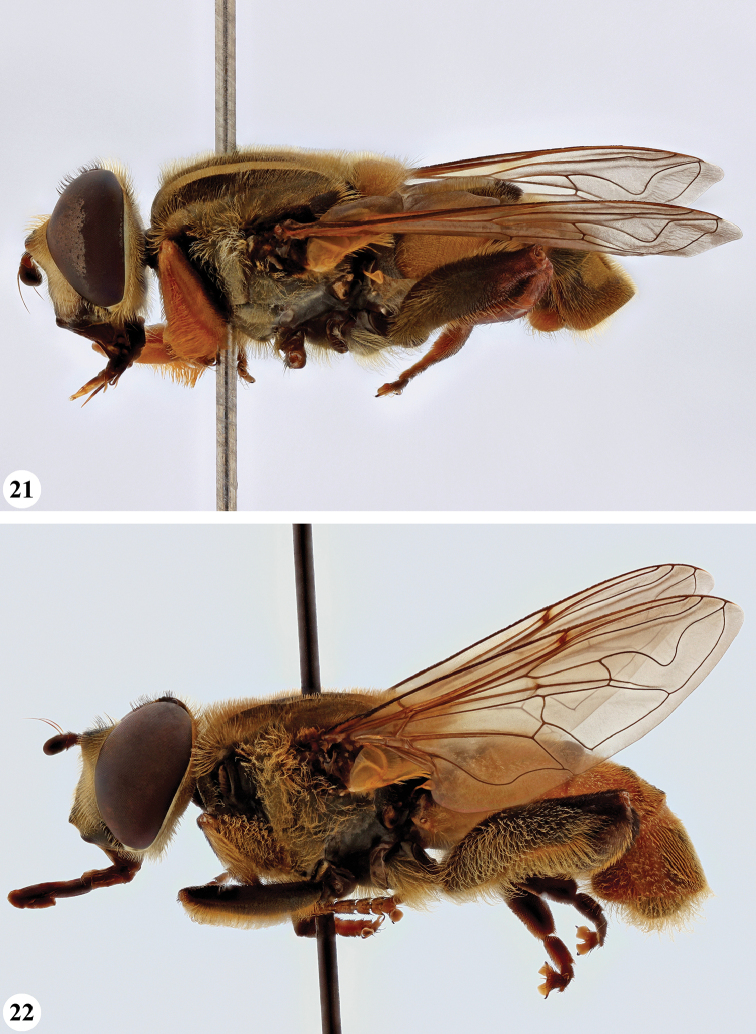
**21***Mesembrius* spp., habitus, lateral view. *M. simplicipes* Curran (♂) **22***M. strigilatus* (Bezzi) (♂).

##### Examined material.

*Mesembrius chapini* Curran: Holotype, male, “Mesembrius // chapini // ♂ // Holotype // Curran” “Lukolela // left bank // Congo R. 1°5’S/7.I.1931” “J.P. Chapin // Ac. 31300” [AMNH]. Type studied from picture on the website.

**Figures 23, 24.**
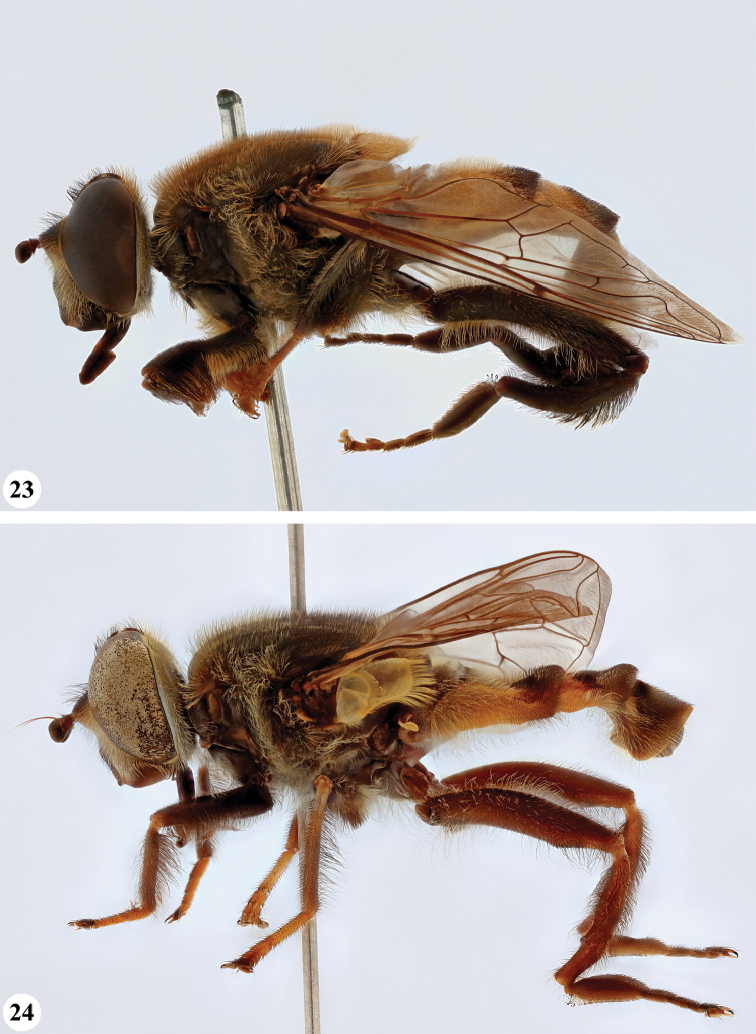
*Mesembrius* spp., habitus, lateral view **23***M. sulcus* sp. nov. (♂) **24***M. tarsatus* (Bigot) (♂).

##### Other material

 Benin • 2♂♂; Ahozon; date unknown; G. Goergen leg.; IITA • 3♂♂ 2♀♀; Calavi; Apr 2014; G. Goergen leg.; IITA • 1♀; Cotonou; 16–18 Jan 2016; G. Goergen leg.; IITA • 1♂ 1♀; Cotonou; 1 Nov 2013; G. Goergen and K. Jordaens leg.; KMMA • 1♂; Cotonou; 28 Jan 2016; K. Jordaens leg.; KMMA • 1♂; Dangbo; 13 Jun 2015; G. Goergen leg.; KMMA • 2♂♂; Lokossa; Nov 2005; G. Goergen leg.; IITA • 1♂; Niaouli; 15 Jan 1998; G. Goergen leg.; IITA • 2♂♂ 1♀; Pahou; 11 Jan 2014; G. Goergen leg.; IITA • 2♀♀; Porto Novo; 28 Nov 2002; G. Goergen leg.; IITA • 1♂; Porto Novo; Oct 2004; G. Goergen leg.; IITA • 1♂; Porto Novo; 27 Jan 2016; G. Goergen leg.; IITA • 1♂; Porto Novo; Jan 2016; K. Jordaens and G. Goergen leg.; KMMA • 2♂♂; 5♀♀; Porto Novo; 20 Jan 2018; G. Goergen leg.; IITA • 1♂ 12♀♀; Porto Novo; 27 Jan 2016; K. Jordaens and G. Goergen leg.; KMMA • 1♂ 1♀; Porto Novo; 27 Jan 2016; K. Jordaens and G. Goergen leg.; IITA • 5♂♂ 8♀♀; Porto Novo; 7 Mar 2018; G. Goergen & K. Jordaens leg.; KMMA • 4♂♂; Porto Novo; date unknown; K. Jordaens leg.; KMMA. Democratic Republic of the Congo • 1♀; Tshopo, Basoko; Oct 1948; P.L.G. Benoit leg.; KMMA • 1♂ 1♀; Equateur, Eala; Jul 1936; J. Ghesquière leg.; KMMA; 1♂; Equateur, Eala; Mar 1936; J. Ghesquière leg.; RMNH • 1♂; Equateur, Eala; 5 Apr 1935; J. Ghesquière leg.; RMNH • 1♂; Equateur, Eala; Sep 1935; J. Ghesquière leg.; RMNH • 1♀; Equateur, Eala; Jan 1936; J. Ghesquière leg.; RMNH • 2♂♂ 2♀♀; Equateur, Eala; Feb 1936; J. Ghesquière leg.; RMNH • 5♂♂ 1♀; Equateur, Eala; Mar 1936; J. Ghesquière leg.; RMNH • 1♀; Equateur, Eala; Sep 1936; J. Ghesquière leg.; RMNH • 1♀; Equateur, Eala; 28 Sep 1936; J. Ghesquière leg.; RMNH • 1♂ 2♀♀; Equateur, Eala; Dec 1936; J. Ghesquière leg.; RMNH • 1♂; Equateur, Eala; 25 Jul 1935; J. Ghesquière leg.; KBIN • 2♂♂ 1♀; Equateur, Eala; Aug 1935; J. Ghesquière leg.; KBIN • 1♂; Equateur, Eala; Sep 1935; J. Ghesquière leg.; KBIN • 1♀; Equateur, Eala; Dec 1935; J. Ghesquière leg.; KBIN • 1♀; Léopoldville [= Kinshasa]; 1 Jun 1915; Lang and Chapin leg.; KMMA. Nigeria • 1♂; Ibadan, IITA Station; 18 Nov 2004; G. Goergen leg.; IITA.

**Figures 25, 26.**
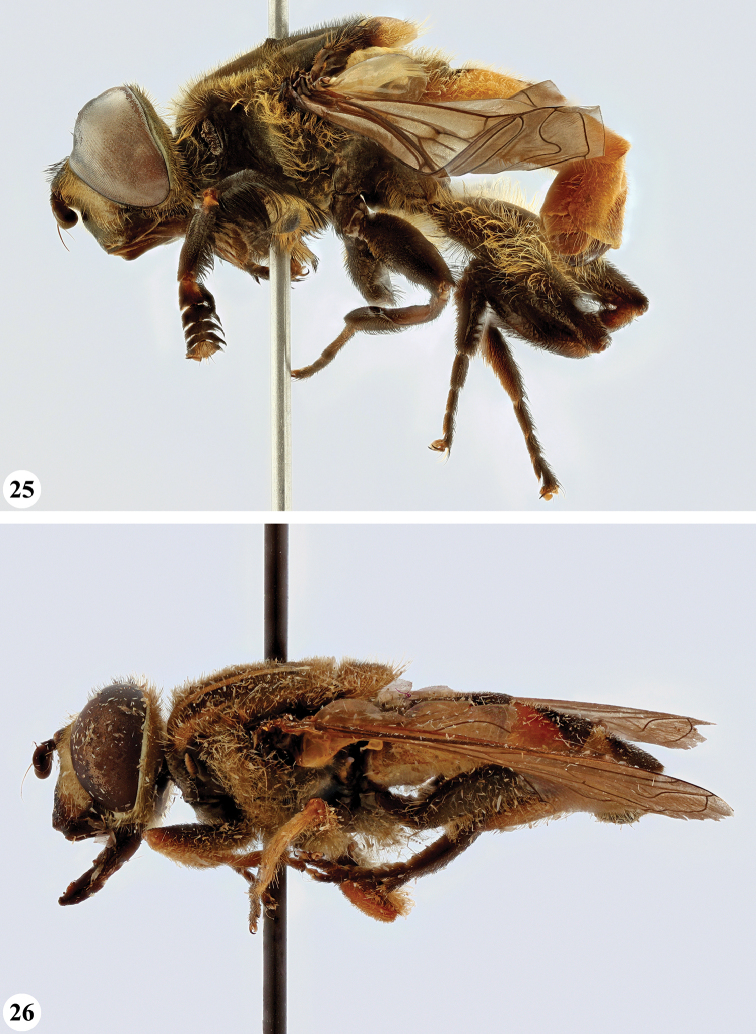
*Mesembrius* spp., habitus, lateral view **25***M. tibialis* sp. nov. (♂) **26***M. vockerothi* sp. nov. (♂).

##### Re-description male

**(Fig. 8).** Body length: 14.0–17.2 mm. Wing length: 10.2–11.3 mm.

**Figures 27, 28.**
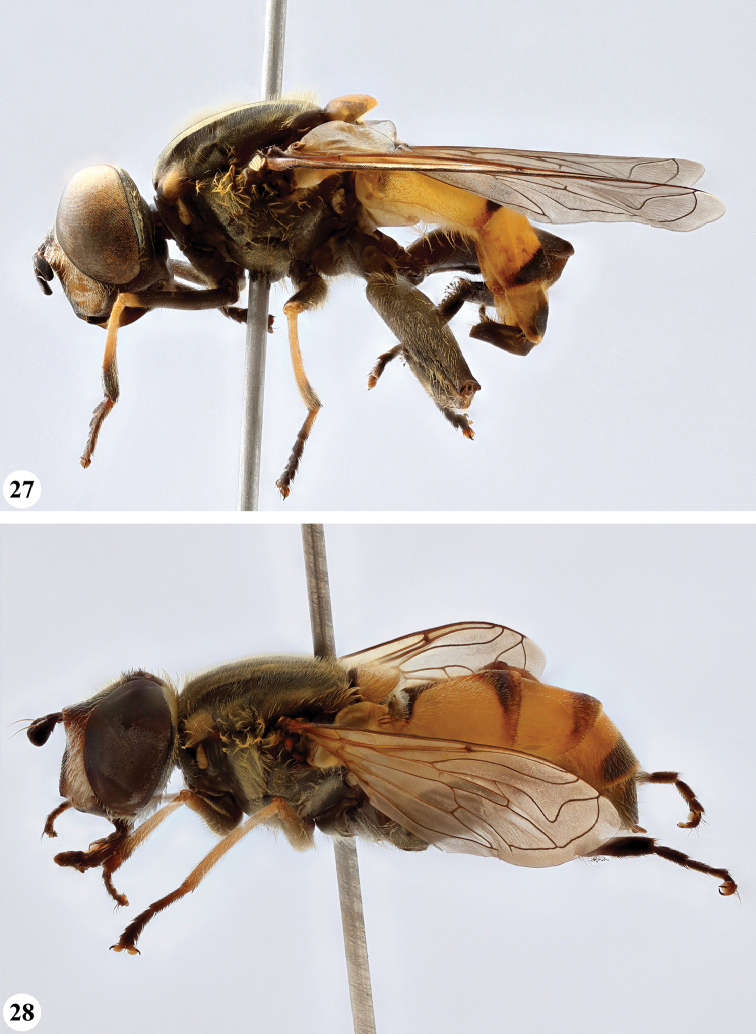
*Mesembrius* spp., habitus, lateral view **27***M. caffer* (Loew) (nominal morph) (♀) **28***M. caffer* (Loew) (spined morph) (♀).

***Head*** (Fig. 51). Eyes bare; holoptic, eye contiguity almost as long as length of ocellar triangle. Face white with dark medial vitta; white pilose; white pollinose. Vertical triangle black; black pilose; yellow pollinose on ventral half. Distance between lateral ocellus and eye margin 1/2 width of ocellus. Frontal triangle short; yellow-white; with some long, black pile; yellow pollinose. Frontal prominence shiny black with orange-brown apex. Occiput yellow; yellow pilose; with some shorter and thicker black pile near eye margin; yellow and white pollinose. Antenna, scape and pedicel reddish-brown; postpedicel black; antennal arista reddish-brown.

**Figures 29, 30.**
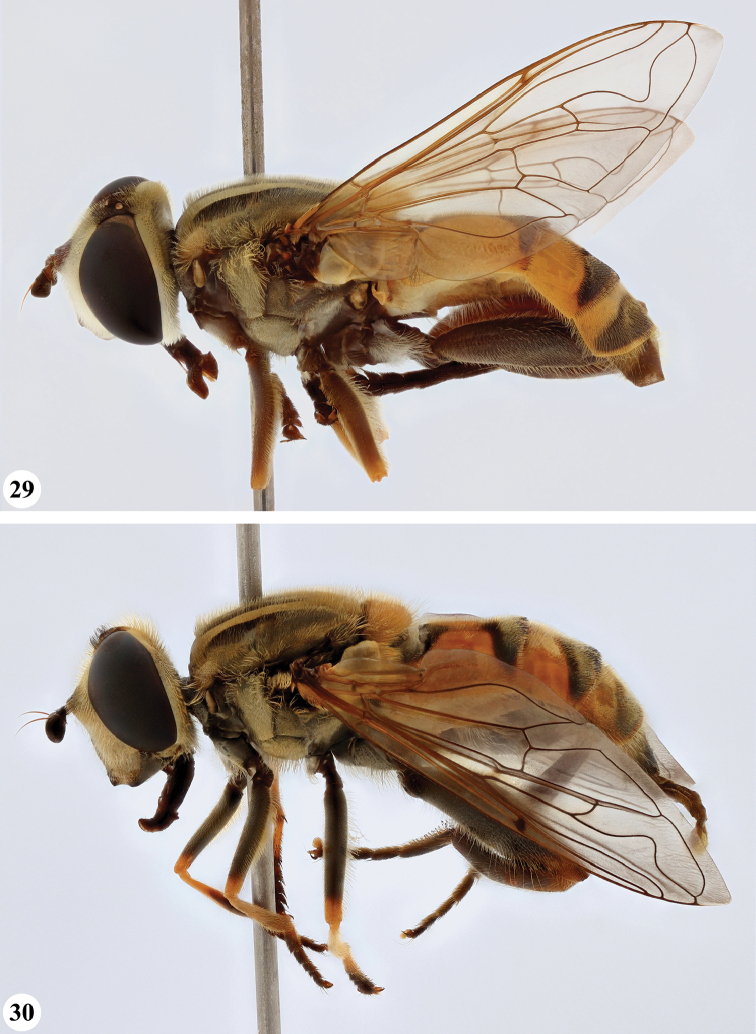
*Mesembrius* spp., habitus, lateral view **29***M. ctenifer* syn. nov. Hull (♀) **30***M. capensis* (Macquart) (♀).

***Thorax.*** Scutum black with dorsally a pair of well-demarcated yellow vittae which are largely connected posteriorly. Scutum with faint lateral vitta; yellow and black pilose. Scutellum uniformly yellow-brown; with long yellow and shorter black pile.

**Figures 31, 32.**
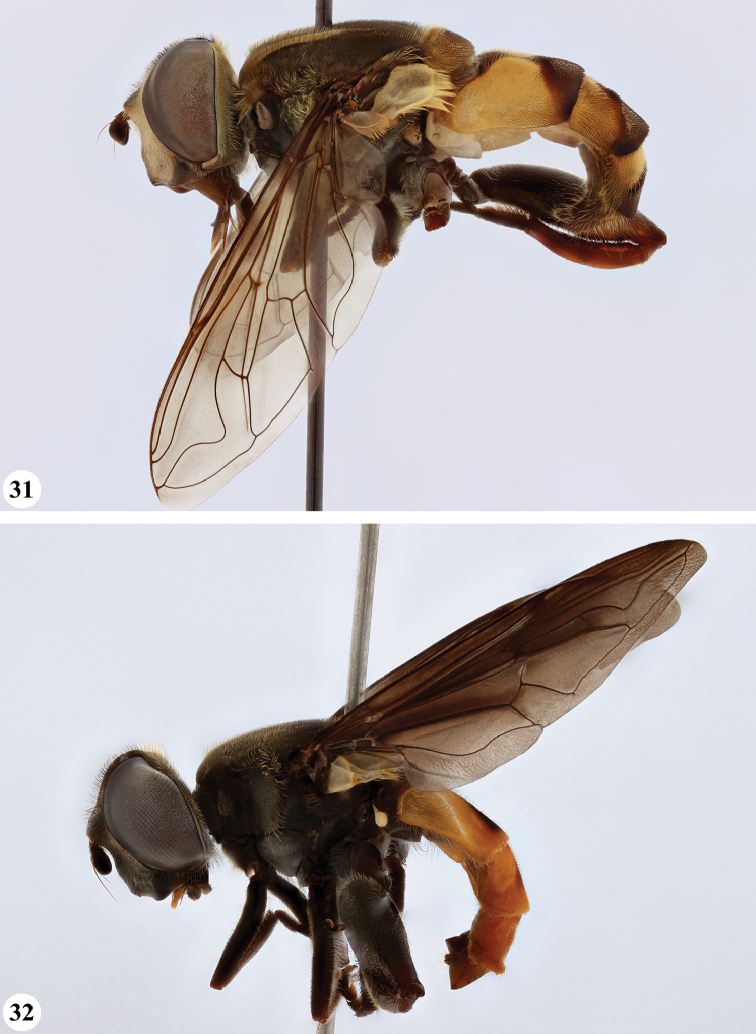
*Mesembrius* spp., habitus, lateral view **31***M. chapini* Curran (♀) **32***M. cyanipennis* (Bezzi) (♀).

***Legs.*** All legs chocolate-brown; tarsi chocolate-brown. Proleg (Fig. 151): Femur dorsoventrally flattened; posterodorsal side with sparse, long black and yellow pile in proximal half; with apical pile brush of long, dense and curved yellow thick pile, this yellow pile is longer in the distal 1/3 and is interspersed with equally long black pile; ventrally with long, black pile. Basitarsus without tuft of orange or black pile posteriorly. Mesoleg: Femur with long, yellow pile on proximal 2/3 and long black pile on distal 1/3. Tibia and tarsi black pilose. Metaleg (Fig. 186): Femur long and thin, slightly curved pile; pile on dorsal and anterior side inconspicuous, except for some long, black pile at anterior distal end; patch of long yellow pile on proximal part; with row of very short and thick black spines on ventral distal half; with 2–5 very long, thick black pile in ventral middle; posteriorly with loose, long yellow and black pile which becomes denser at distal end. Tibia with long, black pile, especially in distal 2/3; anteriorly with shallow excavation in the middle; posteriorly with deep and broad bare excavation in proximal 1/3. Tarsi black pilose dorsally, orange pilose ventrally.

**Figures 33, 34.**
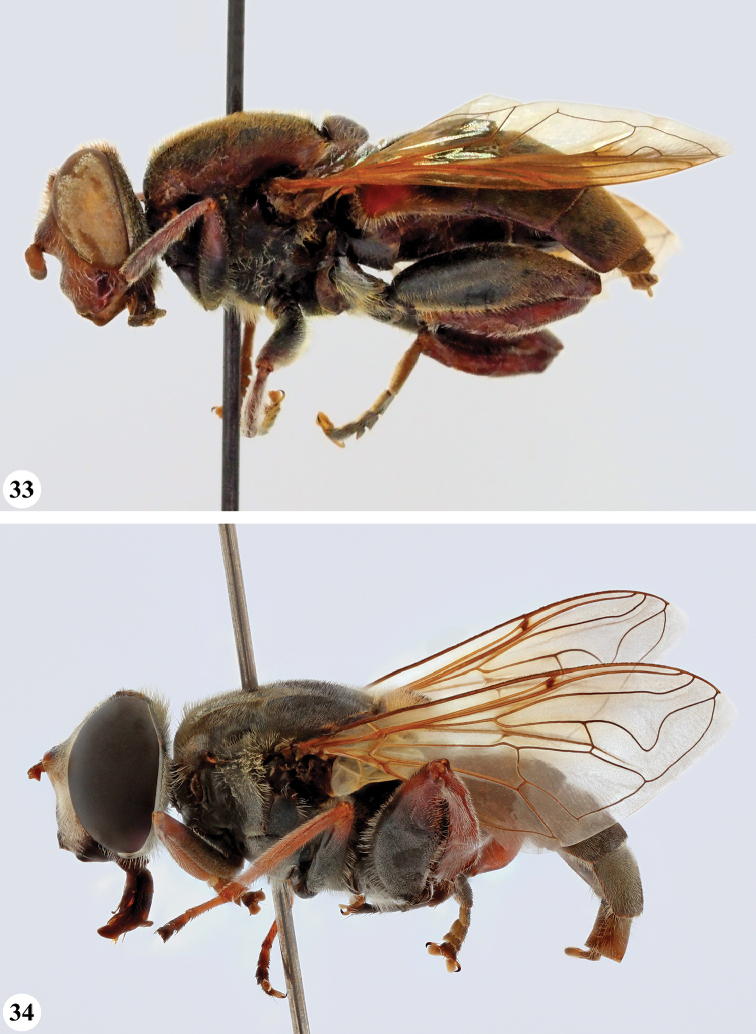
*Mesembrius* spp., habitus, lateral view **33***M. maculifer* Hull (♀) **34***M. madagascariensis* Keiser (♀).

***Wing*** (Fig. 130). Entire wing dark, uniformly dense microtrichose.

**Figures 35, 36.**
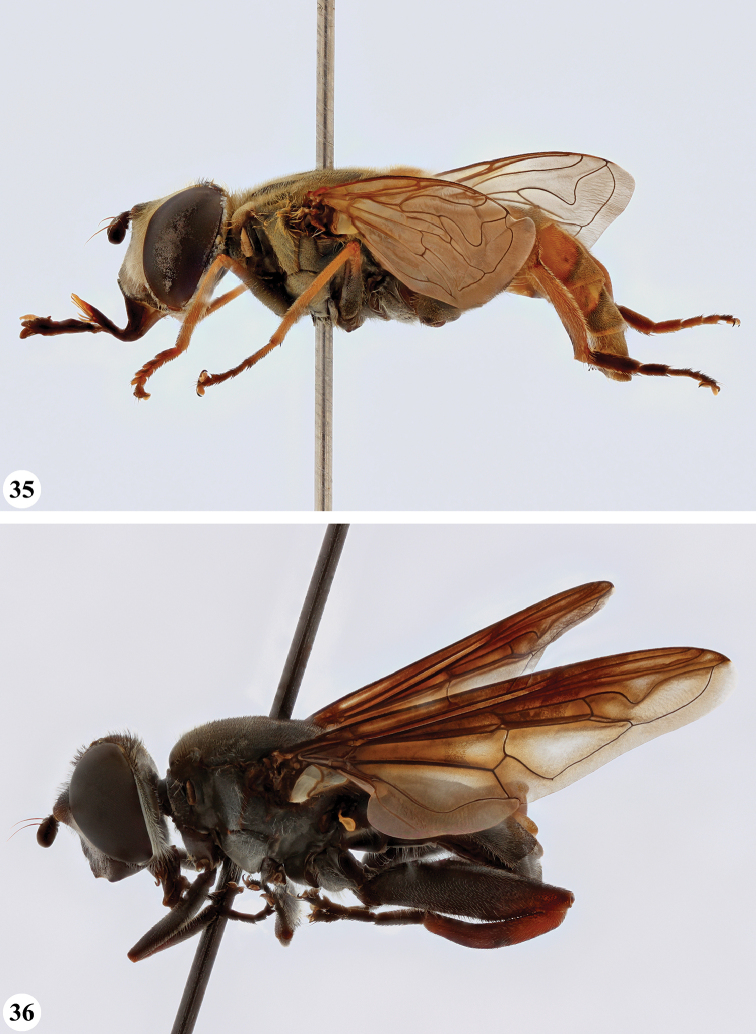
*Mesembrius* spp., habitus, lateral view **35***M. minor* (Bezzi) (♀) **36***M. morio* (Bezzi) (♀).

***Abdomen*** (Fig. 88). Tergite II with pair of very large, yellow rounded maculae; black markings hourglass-shaped; anterior and posterior black markings equal in size marking, but posterior black marking with stronger white pollinosity; yellow and black pilose, but black pile more conspicuous in posterior half of tergite and somewhat denser in posterolateral corners. Tergite III and IV with broad yellow fascia; black markings on posterior half vague because of white pollinosity, but more pronounced in medial part; black and yellow pilose, but black pile rare in anterior 1/4.

**Figures 37, 38.**
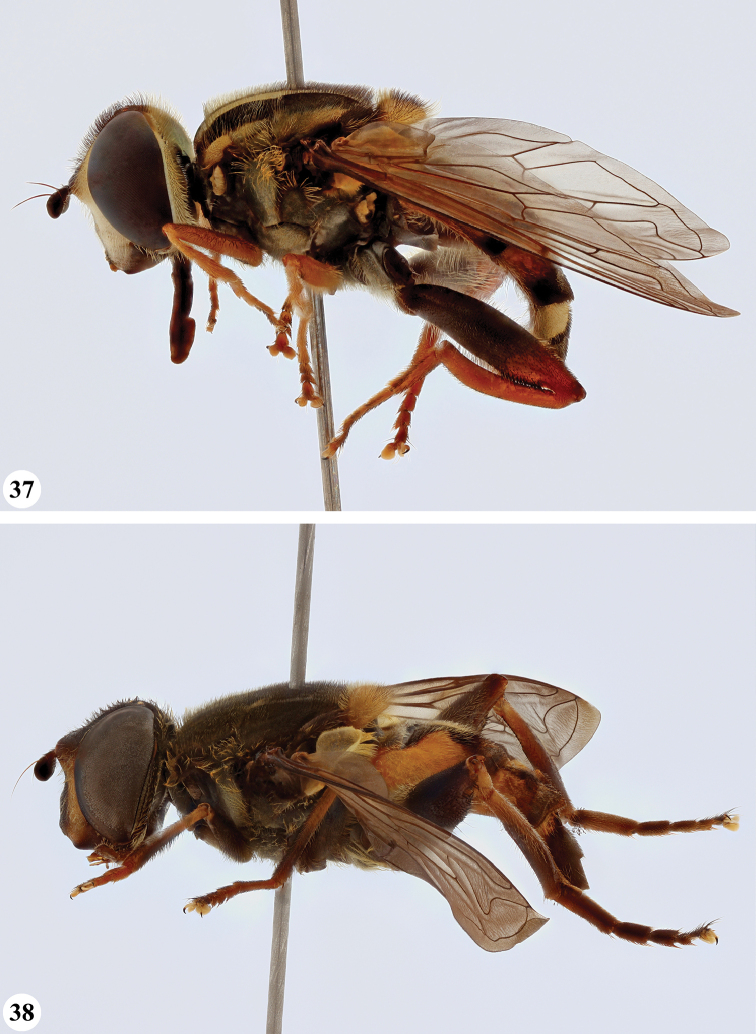
*Mesembrius* spp., habitus, lateral view **37***M. platytarsis* syn. nov. Curran (♀) **38***M. regulus* (Hull) (♀).

***Genitalia*** (Fig. 210). Epandrium: Dorsal lobe of surstylus short, broadly rounded; with short black spines on almost entire surface. Ventral lobe of surstylus straight; bare.

**Figures 39, 40.**
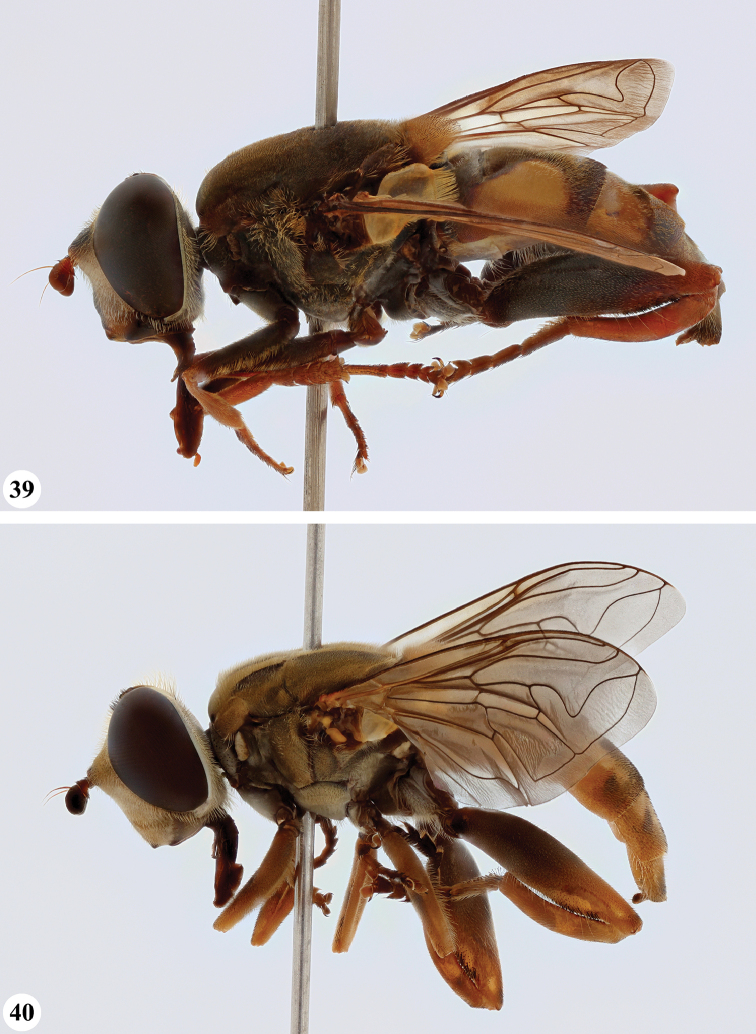
*Mesembrius* spp., habitus, lateral view **39***M. rex* Curran (♀) **40***M. senegalensis* (Macquart) (♀).

##### Description female

**(Fig. 31).** Body length: 13.3–16.3 mm. Wing length: 10.2–11.0 mm.

**Figures 41, 42.**
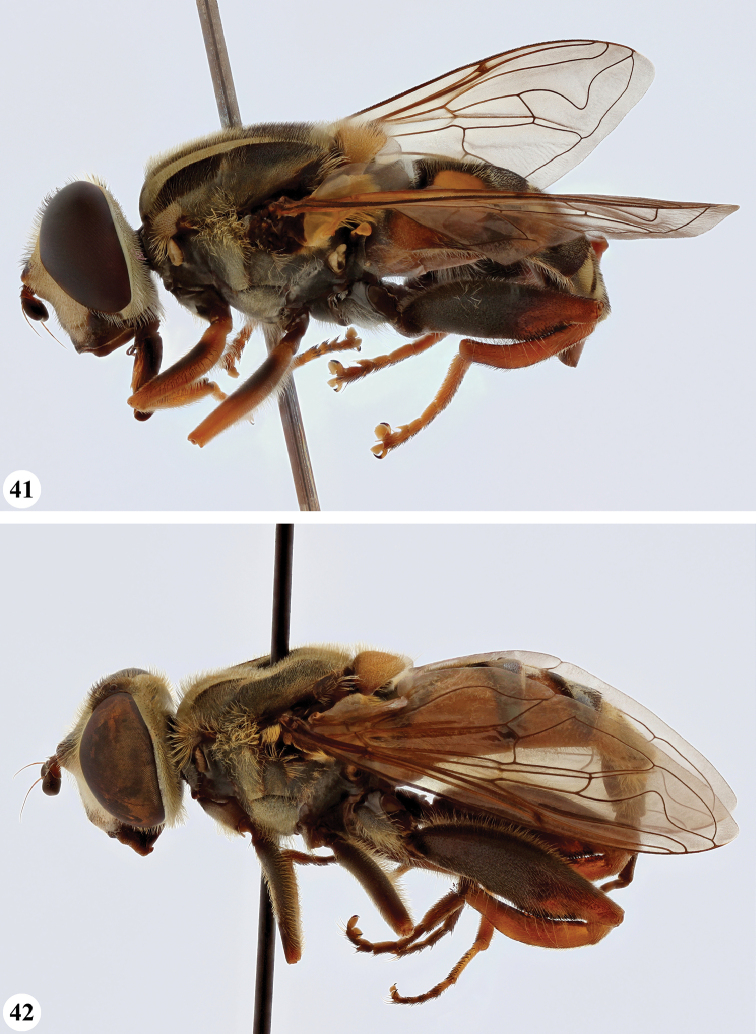
*Mesembrius* spp., habitus, lateral view **41***M. simplicipes* Curran (♀) **42***M. strigilatus* (Bezzi) (♀).

***Head*** (Fig. 72). Eye bare; dichoptic. Face yellow-orange with dark medial vitta; white pilose; white pollinose. Frons black; black pilose in dorsal half, black and yellow pilose on ventral half; weakly white pollinose. Distance between lateral ocellus and eye margin approx. width of ocellus. Occiput black; yellow pilose, with some black pile near eye margin; yellow-white pollinose. Frontal prominence shiny black, distal end orange-brown; scape and pedicel orange-brown; postpedicel black; antennal arista reddish-brown.

**Figures 43, 44.**
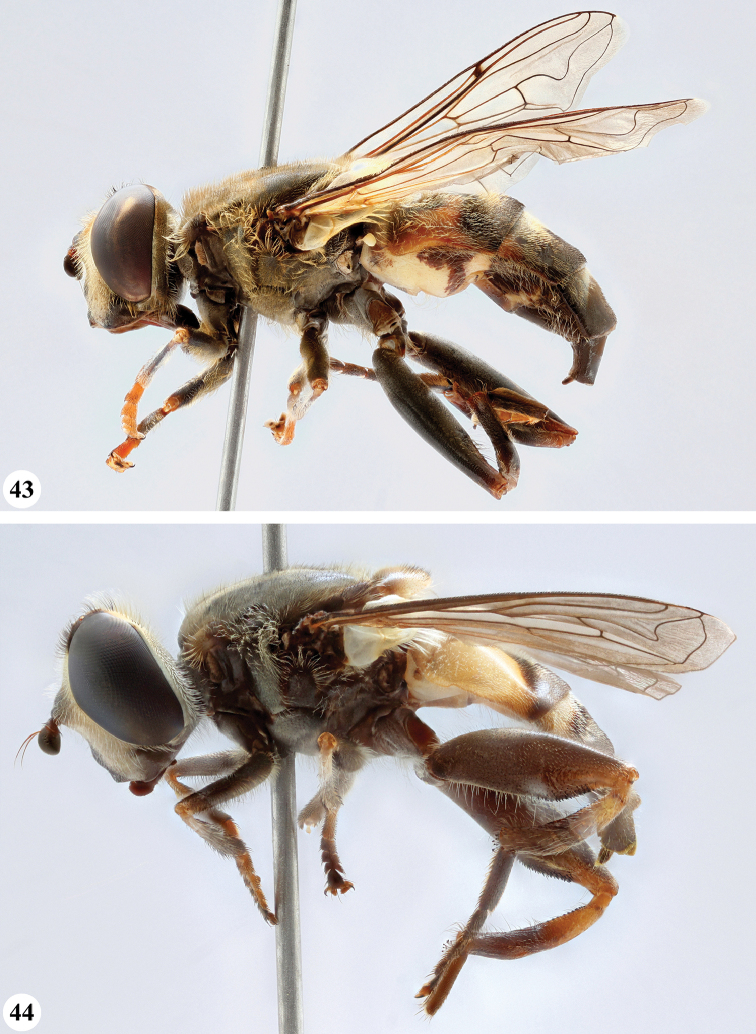
*Mesembrius* spp., habitus, lateral view **43***M. sulcus* sp. nov. (♀) **44***M. tarsatus* (Bigot) (♀).

***Thorax.*** Scutum dark brown to black with dorsolateral a pair of vague, grey pollinose vittae which are connected posteriorly; grey pollinose lateral vitta very vague; yellow pilose with some black pile interspersed. Scutellum yellow-orange; yellow pilose with very sparse, shorter black pile interspersed.

**Figure 45.**
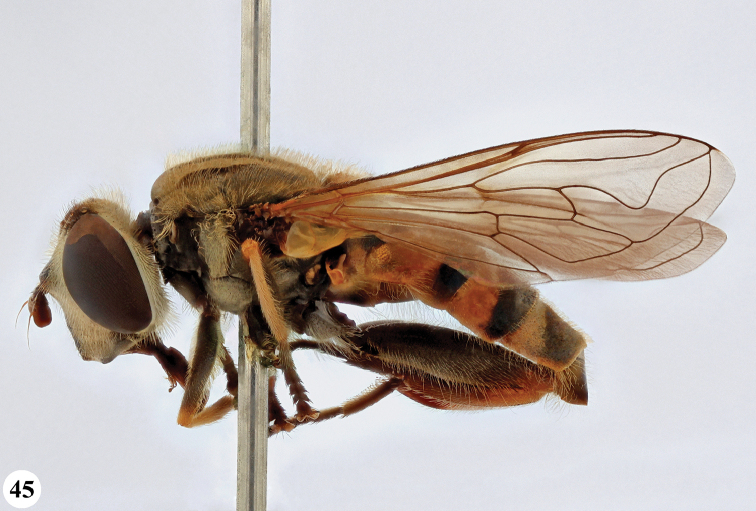
*Mesembrius* spp., habitus, lateral view. *M. vockerothi* sp. nov. (♀).

***Legs*** (Figs 169, 201). All femora black, except for extreme distal ends which are orange-brown; yellow pilose. All tibia orange-brown, most distal tarsomere darkened; yellow and black pilose dorsally, yellow pilose ventrally. All tarsi orange-brown; black pilose, except for protarsus which is yellow-orange pilose ventrally.

***Wing.*** Entire wing uniformly dense microtrichose.

***Abdomen*** (Fig. 111). Tergite II with a pair of large, orange, rounded maculae; yellow pilose, with dense short thick black pile on posterior black marking. Tergite III with orange fascia in anterior half and black marking in posterior half; yellow pilose with dense short thick black pile on posterior black marking. Tergite IV with narrow orange fascia; yellow-orange pillose. Tergite V black; yellow pilose.

##### Distribution.

Benin, Democratic Republic of the Congo and Nigeria.

##### Comments.

The female of the species was hitherto unknown. The male cannot be confused with any other species.

#### Mesembrius copelandi

Taxon classificationAnimaliaDipteraSyrphidae

Jordaens, Goergen & De Meyer sp. nov.

7445843A-2C37-57C4-903A-CFFDF002A473

http://zoobank.org/9383F227-89B2-4F37-889F-080B3327C04D

Figs 9 , 52 , 89 , 131 , 199 , 211

##### Differential diagnosis.

Males of *Mesembrius copelandi* sp. nov. lack an apical pile brush on the profemur and have an unmodified metatibia. The ventral 1/4 of the profemur is black pilose with some longer yellow pile antero- and posteroventrally. The metaleg has long, yellow pile which becomes darker on the tarsi; the pile is much shorter on the posterior side. The metafemur has a patch of conspicuous black pile on the proximal 1/5 ventrally. The metabasitarsus is very long and almost as long as the metatibia; in all other species, the metabasitarsus is much shorter than the metatibia. The female is unknown.

##### Examined material.

*Mesembrius copelandi* Jordaens, Goergen & De Meyer: Holotype, male, “HOLOTYPUS” “Kenya // Nairobi Prov. // ICIPE campus // Kasarani, 1.22296°S, // 36.89704°E, 1600 m” “6 m Malaise trap, near // stream, woodland // remnant, 17-25 JAN // 2017, R. Copeland” “Mesembrius copelandi//Det. K. Jordaens, 2019” “DNA 1301F04 // K. Jordaens // RMCA 2020” “ICIPE 1180” [KMMA]

Paratype: Kenya • 1♂; Sosoma area; 30 Jun–6 Jul 2018; R. Copeland leg.; ICIPE 9544; ICIPE.

##### Description male

**(Fig. 9).** Body length: 13.7–14.7 mm. Wing length: 9–10.5 mm.

***Head*** (Fig. 52). Eyes bare; slightly dichoptic, distance between eyes approx. 1/2 width of ocellus. Face white-yellow with dark medial vitta; white pollinose; white pilose. Vertical triangle black pilose, with some yellow pile near vertex; yellow pollinose until just before anterior ocellus. Distance between lateral ocellus and eye margin 1/2 width of ocellus. Occiput yellow; yellow pilose; yellow and white pollinose. Frontal triangle brownish; yellow pilose with a few black pile near antenna; yellow pollinose. Frontal prominence shiny black, dark brown at apex. Antenna, scape and pedicel reddish-brown; postpedicel black; antennal arista reddish-brown.

***Thorax.*** Scutum black; with a pair of well-demarcated yellow vittae and a yellow medial line dorsally, vitta and medial line are broadly connected posteriorly; with lateral, yellow vitta; yellow pilose with some black pile in the posterolateral corners. Scutellum uniformly yellow; yellow pilose.

***Legs.*** Tibia and tarsi entirely rufous, but metatarsi black dorsally. Proleg: Femur black, distal end rufous; without apical pile brush; pile ventrally black on distal 1/4, otherwise rufous. Tibia rufous; black and yellow pilose which is longer on posterior side. Basitarsus black and rufous pilose. Other tarsi black pilose. Mesoleg: Femur black, distal end rufous; yellow and black pilose. Tibia similar as in proleg, but black and yellow pile in proximal 1/4 of markedly longer than on remainder of tibia. Tarsi similar as in proleg. Metaleg (Fig. 199): Femur black, distal end rufous; with long, thin yellow pile, but shorter and less dense ventrally and posteroventrally; with a band of black pile on extreme proximal end ventrally. Tibia rufous; unmodified; long yellowish pilose, except for the posterior side where pile shorter and black; with a patch of posteroventral longer black pile. Basitarsus black dorsally, rufous ventrally; very long, almost as long as tibia; very long yellow pilose with some long black pile at distal end. Other tarsi black dorsally, rufous ventrally; very long yellow pilose with some long black pile at distal end.

***Wing*** (Fig. 131). Entire wing uniformly dense microtrichose.

***Abdomen*** (Fig. 89). Tergite II with a pair of large, yellow rectangular maculae; black marking hourglass-shaped; posterior black marking equal in size or somewhat narrower than anterior black marking. Posterior black part with short, stiff black setulae which to not extend to the lateral tergal sides. Tergite III and IV with yellow fascia of variable size, often occupying almost the entire tergite with mostly posterior, triangular black marking. Tergite V strongly white pollinose, except for a black medial zone; with short, black stiff setulae at posteriorly which do not reach the lateral tergal sides, these setulae are absent in specimens where the posterior black marking is strongly reduced.

***Genitalia*** (Fig. 211). Epandrium: Dorsal lobe of surstylus elongated, distally rounded, with characteristic small tooth-like projection; dorsally long yellow pilose, short black pilose at distal end. Ventral lobe of surstylus with one large black setula in middle section and a row of approximately ten long black setulae.

##### Female.

unknown.

##### Distribution.

Kenya.

##### Comments.

This is a new species to the Afrotropical Region. The species is only known from two males from Kenya. Two DNA barcodes are available (Fig. 229) and the species is strongly differentiated from others.

##### Etymology.

Named in honour of Robert Copeland (ICIPE) who collected both males. The specific epithet should be treated as a noun in the genitive case.

#### Mesembrius cyanipennis

Taxon classificationAnimaliaDipteraSyrphidae

(Bezzi, 1915)

B32BAEF0-179C-51C6-9292-EAA6D1C2F139

Figs 10 , 32 , 53 , 71 , 90 , 112 , 113 , 132 , 212

Helophilus cyanipennis Hervé-Bazin, 1914a: 297. Nomen nudum.
Helophilus (Mesembrius) cyanipennis Bezzi, 1915: 97.
Helophilus cyanipennis – Curran (1927): 60 – Brunetti (1926): 166 – Curran (1939): 9 – Smith and Vockeroth (1980): 504.

##### Differential diagnosis.

*Mesembrius cyanipennis* males are dichoptic, have a black face, no apical pile brush on the profemur, an unmodified metatibia, have short, but conspicuously dense dark pile on the anterior proximal half of all femora, a dark scutum without vittae and a largely yellow-orange abdomen. *Mesembrius cyanipennis* is one of the two species with a black face and differs from *M. nigriceps* in the absence of conspicuous thick black pile on the ventral side of the metafemur. Females have a black face (as in *M. morio*), but tergite II has a pair of large orange maculae and the other tergites are to a various extent orange (tergites entirely black in *M. morio*). All other species have a white to yellow face with a medial dark vitta.

##### Examined material.

*Helophilus cyanipennis* Bezzi: Lectotype (hereby designated), male, “SYNTYPUS” “Syn-//type” “Hel. (Mes.)//♂ Type// cyanipennis//Bezzi” “Pres. By//Impl. Bureau Ent.//1915-165.” “Uganda Prot.,//Entebbe.//1–11 Sep1911.//S.A. Neave.” “Mesembrius//cyanipennis//n.sp//Type ♂” “NHMUK 013428968” [NHMUK]. Paralectotype, female, “Syn-//type” “Hel. (Mes.)//♀ Type// cyanipennis//Bezzi” “Ashanti.//Obuasi.//7.xiii.06//W.M. Graham.//1907-74.” “Mesembrius//cyanipennis//Type ♀ n.sp.” “NHMUK 013428969” [NHMUK]. Paralectotype, female, “Syn-//type”, “H.(M.) cyanipennis, Bezzi//Bezzi det.//1915.)” “caught on flower” “Obuasi,//Ashanti,//20.vii.1907,//Dr. W.M. Graham.//1908-245.” “NHMUK 013428970” [NHMUK]. Paralectoype, female, “Syn-//type”, “H.(M.) cyanipennis, Bezzi//Bezzi det.//1915.)” “caught on flower” “Obuasi,//Ashanti,//21.vi.1907,//Dr. W.M. Graham//1908-245.” “NHMUK 013428971” [NHMUK]. Paralectotype, female, “Syn-//type”, “H.(M.) cyanipennis, Bezzi//Bezzi det.//1915.)” “Caught on umbelli-//ferus flowers in//swamp.” “Obuasi,//Ashanti,//28.vi.1907,//Dr. W.M. Graham//1908-245.” “NHMUK 013428972” [NHMUK].

##### Other material

 Benin • 1♂ 1♀; Ifangni-range; 6 Jun 2015; G. Goergen leg.; IITA • 1♀; Ifangni-range; 6 May 2016; G. Goergen leg.; IITA • 1♂ 1♀; Ifangni-range; 19 Mar 2017; G. Goergen leg.; IITA • 1♂; Lokossa; Oct 2005; G. Goergen leg.; IITA • 1♀; Niaouli; 10 Dec 2013; G. Goergen and K. Jordaens leg.; KMMA • 1♂ 3♀♀; Niaouli; 2 Feb 2014; G. Goergen leg.; IITA • 1♂; Pobé; 13 Jun 2015; G. Goergen leg.; IITA • 1♀; Pobé; 28 Jan 2016; G. Goergen leg.; IITA • 1♀; Pobé; 27 Jan 2016; G. Goergen leg.; KMMA. Cameroon • 1♀; Abong M’Bang District; 1–30 Apr 1936; F.G. Merfield leg.; NHMUK. Democratic Republic of the Congo • 1♀; Haut-Uélé, Arebi; date unknown; J. Bequaert leg.; KMMA • 1♀; Bas-Uélé, Bambesi; 15 Sep 1933; H.J. Brédo leg.; KMMA • 1♀; Bas-Uélé, Bambili; date unknown; J. Rodhain leg.; KMMA • 1♀; Lulua, Kapanga; Nov 1932; G.F. Overlaet leg.; KMMA • 1♀; Katanga; Mar 1933; G.F. Overlaet leg.; KMMA • 1♀; Ituri, Kilo, Kere-Kere; Mar 1948; Turco leg.; KMMA • 1♀; Tshopo, Stanleyville [= Kisangani]; 1914; J. Bequaert leg.; KMMA • 1♀; Tshopo, Stanleyville [= Kisangani]; 4 Apr 1915; Lang and Chapin leg.; KMMA • 1♀; Tshopo, Stanleyville [= Kisangani]; 7 Apr 1915; Lang and Chapin leg.; KMMA • 1♀; Tshopo, Stanleyville [= Kisangani]; 8 Apr 1915; Lang and Chapin leg.; KMMA • 1♀; Tshopo, Stanleyville [= Kisangani]; 9 Apr 1915; Lang and Chapin leg.; KMMA • 1♂ 1♀; Tshopo, Stanleyville [= Kisangani]; Mar 1915; Lang and Chapin leg.; KMMA • 1♂ 1♀; Elisabethville [= Lubumbashi]; 11 Apr 1921; M. Bequaert leg.; KMMA • 1♀; Lomami, Mutombo; Mar 1931; P. Quarré leg.; KMMA • 1♀; Haut-Lomami, Sankisia; 4 Apr 1911; Dr. Bequaert leg.; KMMA • 1♂; Uelé; date unknown; J. Rodhain leg.; KMMA • 1♂; Eala; Apr 1935; J. Ghesquière leg.; RMNH • 1♀; Eala; Feb 1936; J. Ghesquière leg.; RMNH • 1♀; Eala; Nov 1935; G.F. Overlaet leg.; RMNH • 1♀; Eala, Bambesa; 30 Oct 1933; J. Leroy leg.; RMNH • 1♀; Lulua, Kapanga; Nov 1932; G.F. Overlaet leg.; RMNH. Ghana • 1♂; Wati Waterfalls; Feb 2003; G. Goergen leg.; IITA. Nigeria • 1♀; Ibadan, IITA station; 3 Feb 2000; G. Goergen leg.; IITA • 1♂; Ikotobo; 10 Nov 1913; J.W.S. ScottMacie leg.; NHMUK. Sierra Leone • 1♂ 1♀; Kamakoni; 22 Apr 1912; J.J. Simpson leg.; NHMUK. South Africa • 1♂; KwaZulu-Natal, Manguzi Forest Reserve; 13–16 Dec 2010; J.G.H. Londt leg.; NMSA. Togo • 1♀; Kloto Forest; Dec 2007; G. Goergen leg.; IITA • 1♀; Kloto Forest; Nov 2007; G. Goergen leg.; IITA • 1♂; Kloto Forest; Feb 2016; G. Goergen leg.; IITA • 2♀♀; Kloto Forest; 21–24 Jun 2015; G. Goergen leg.; KMMA • 1♀; Kloto Forest; Feb 2016; G. Goergen leg.; KMMA • 1♀; Kloto Forest; Nov 2016; G. Goergen leg.; KMMA • 2♂♂ 2♀♀; Kloto Forest; Feb 2017; G. Goergen leg.; KMMA • 1♂; Kloto Forest; Mar 2017; G. Goergen leg.; KMMA • 1♀; Kuma Adamé; 22–24 Jan 2016; G. Goergen leg.; IITA. Uganda • 2♂♂ 1♀; Entebbe; 9 Aug 1911; C.C. Gowdey leg.; NHMUK • 1♂; Entebbe; 11 Aug 1911; C.C. Gowdey leg.; NHMUK • 1♀; Entebbe; 14 Aug 1911; C.C. Gowdey leg.; NHMUK • 1♂ 1♀; Entebbe; 16 Aug 1911; C.C. Gowdey leg.; NHMUK • 2♂♂; Entebbe; 17 Aug 1911; C.C. Gowdey leg.; NHMUK • 4♂♂ 2♀♀; Entebbe; 21 Aug 1911; C.C. Gowdey leg.; NHMUK • 1♀; Entebbe; 31 Aug 1911; C.C. Gowdey leg.; NHMUK • 1♂; Entebbe; 17 Jul 1911; C.C. Gowdey leg.; NHMUK • 1♀ • Entebbe; 28 Jul 1911; C.C. Gowdey leg.; NHMUK • 1♂ 3♀♀; Entebbe; 12–13 Dec 1912; C.C. Gowdey leg.; NHMUK • 3♀♀; Entebbe; 3–4 Dec 1912; C.C. Gowdey leg.; NHMUK • 1♂; Entebbe; 27 May 1912; C.C. Gowdey leg.; NHMUK • 1♀; Entebbe; 3 Nov 1912; C.C. Gowdey leg.; NHMUK • 3♀♀; Entebbe; 14 Nov 1912; C.C. Gowdey leg.; NHMUK • 9♀♀; Entebbe; 18–20 Nov 1912; C.C. Gowdey leg.; NHMUK • 1♀; Entebbe; 16 Oct 1912; C.C. Gowdey leg.; NHMUK • 1♀; Entebbe; 3 Sep 1912; C.C. Gowdey leg.; NHMUK • 6♂♂ 9♀♀; Entebbe; 1–11 Sep 1911; S.A. Neave leg.; NHMUK • 1♂; Entebbe; 7–9 May 1912; S.A. Neave leg.; NHMUK • 1♀; Entebbe; 18 May 1912; collector unknown; NHMUK • 1♂ 1♀; Entebbe; 7 Mar 1973; H. Falke leg.; RMNH • 1♀; Entebbe; 7 Mar 1973; H. Falke leg.; RMNH • 1♀; Entebbe; 7 Oct 1971; H. Falke leg.; RMNH • 1♀; Entebbe, Kisubi Forest; 8–9 Jun 1976; M.K. Paulus leg.; CNC • 1♀; Kenya Coast, Gedi for Malindi; May 1973; H. Falke leg.; CNC • 1♀; W. shores of Vic. Nyanza; 19–25 Sep 1911; S.A. Neave leg.; NHMUK • 2♀♀; locality and date unknown; C.C. Gowdey leg.; NHMUK. Country unknown • 1♂; Ruwengo; 14 May 1911; collector unknown; NHMUK.

##### Re-description male

**(Fig. 10).** Body length: 11.0–13.2 mm. Wing length: 9.6–10.5 mm.

***Head*** (Fig. 53). Eyes bare; dichoptic, distance between eyes approx. 1½x width of ocellus. Face dark brown to black; white pilose; white pollinose. Vertical triangle black; black pilose, yellow pilose on vertex; yellow pollinose until just before anterior ocellus. Distance between lateral ocellus and eye margin 1/2 width of ocellus. Frontal triangle brown, area near eye margin yellow; white pilose; white pollinose. Frontal prominence shiny black, reddish-brown at apex; black pilose. Occiput black; white pilose with some shorter, thicker, black pile near dorsal eye margin. Antenna, scape and pedicel reddish-brown; postpedicel black; antennal arista reddish-brown.

***Thorax.*** Scutum black; without vitta; pile short, yellow and black. Scutellum, anterior half dark brown, posterior half lighter; yellow pilose with some shorter black pile interspersed.

***Legs.*** Legs predominantly dark brown to black. Pile on posterior side of pro- and mesofemur and on proximal distal 1/4 yellow, pile otherwise black; black pile on ventral side of femora gradually becoming longer towards distal end; ventral side of metatibia with carina.

***Wing*** (Fig. 132). Entire wing uniformly, very densely microtrichose; anterior medial part and posterior half of cell bm brownish.

***Abdomen*** (Fig. 90). Tergite II with large, yellow fascia, interrupted in anterior 2/3; with T-shaped black marking; yellow pilose, with short thick black pile interspersed, especially in posteromedial section. Tergite III and IV yellow-orange; yellow pilose.

***Genitalia*** (Fig. 212). Epandrium: Dorsal lobe of surstylus very long and thin, with pointed apex and with distal end bent upwards; with short, black spines at apex and long yellow pilose on dorsal side. Ventral lobe of surstylus convex.

##### Re-description female

**(Fig. 32).** Body length: 12.2–13.1 mm. Wing length: 8.6–11.0 mm.

***Head*** (Fig. 71). Eyes bare; dichoptic. Face black; black and white pilose; white pollinose. Frons black; black pilose; lower half white pollinose. Vertex black; black pilose; grey pollinose. Distance between lateral ocellus and eye margin approx. the width of ocellus. Occiput black; yellow and black pilose dorsally, yellow pilose ventrally; grey pollinose. Frontal prominence shiny brown-black; black pilose. Antenna black; arista reddish-brown.

***Thorax.*** Scutum and scutellum black; without vitta; short white and black pilose.

***Legs.*** Dark reddish-brown to black; short black and white pilose.

***Wing.*** Entire wing uniformly, very densely microtrichose; anterior medial part and posterior half of cell bm brownish.

***Abdomen*** (Figs 112, 113). Second tergite with a pair of large orange maculae and without (Fig. 112) or with (Fig. 113) distinct posterior black marking. Tergite III from largely orange with a vague medial black marking (Fig. 112) to black with a pair of large, orange maculae (Fig. 113). Tergite IV from entirely orange (Fig. 112) to orange with a posterior black fascia (Fig. 113). Tergite V orange. All tergites short yellow-white and black pilose.

##### Distribution.

Benin, Cameroon, Democratic Republic of the Congo, Ghana, Nigeria, Sierra Leone, South Africa, Togo and Uganda.

##### Comments.

Hervé-Bazin (1914a) was the first to use the name *cyanipennis* (as *Helophilus cyanipennis* Bezzi). Since he did not provide a description of the species, but mentions *that the species will be described later by Mr. Prof. Bezzi* [p. 297: “Cette espèce sera prochainement décrite par M. le Prof Bezzi”], the name should be considered as a *nomen nudum*. Bezzi (1915) is the first to provide an adequate description for the species.

#### Mesembrius ingratus

Taxon classificationAnimaliaDipteraSyrphidae

(Loew, 1858)

579D9241-28DE-5AFF-974E-995F3D727CA1

Figs 11 , 54 , 91 , 133 , 158 , 190 , 213

Helophilus ingratus Loew, 1858: 380.
Helophilus ingratus – Loew (1860): 386 – Hervé-Bazin (1914a): 297.
Helophilus (Mesembrius) ingratus – Bezzi (1915): 97.
Tubifera ingrata – Kertész (1910): 254.
Mesembrius ingratus – Curran (1927): 60 – Szilády (1942): 92 – Smith and Vockeroth (1980): 504.

##### Differential diagnosis.

*Mesembrius ingratus* males are holoptic, have a loose black apical pile brush on the profemur, a densely yellow pilose scutum with vague longitudinal vittae, a tuft of black pile on the posterior side of the probasitarsus, a slender metatibia and a posterior deep depression in the proximal half of the metatibia which ventrally extends into a deep groove. It can be distinguished from any other species by the apical pile brush of the profemur which is loose and yellowish with some black pile interspersed (black dorsally, yellow ventrally in *M. arcuatus* sp. nov.; black in *M. tarsatus*) and by the deep groove on the metatibia (strongly compressed in *M. arcuatus* sp. nov.; with a rounded swelling in *M. tarsatus*). The female is unknown.

##### Examined material.

*Helophilus ingratus* Loew: Holotype, male, “Helophilus // ingratus” “209” “209” “HOLOTYPUS // Helophilus ingratus // Loew, 1858” “design. Kassebeer 1993” “NHRS-BYWS // 000002619” [NRMS].

##### Other material

 Malawi • 1♂; Mount Mulanje; 20 Oct 1912; S.A. Neave leg.; NHMUK. Senegal • 2♂♂; Dakar; 14 Jan 1945; collector unknown; RMNH. South Africa • 1♂; KwaZulu-Natal, Manguzi Forest Reserve; 13–16 Dec 2010; J.G.H. Londt leg.; NMSA • 1♂; KwaZulu-Natal, St. Lucia Park Reserve; 2 Feb 1988; J.G.H. Londt leg.; NMSA • 1♂; KwaZulu-Natal, Ngoya Forest Reserve; 26 Apr 1988; J.G.H. Londt leg.; NMSA.

##### Re-description male

**(Fig. 11).** Body length: 10.9–11.1 mm. Wing length: 8.0–8.4 mm.

***Head*** (Fig. 54). Eyes bare; holoptic, length of eye contiguity approx. 1/3 the length of ocellar triangle. Face white with dark medial vitta; white pilose; white pollinose. Vertical triangle with black pile in ventral half and at ocellar triangle; yellow pilose in dorsal half; yellow pollinose. Distance between lateral ocellus and eye margin 1/2 width of ocellus. Frontal triangle and gena white; white pilose; white pollinose. Frontal prominence shiny black; black pilose. Occiput black, but strongly white-grey pollinose; yellow-brown pilose, with a row of almost equally long black pile at dorsal eye margin. Antenna reddish-brown.

***Thorax.*** Scutum dark brown with dorsally a pair of vague yellow vittae; yellow-brown pilose. Scutellum uniformly light yellow-brown; yellow pilose.

***Legs.*** All legs dark brown, except for pro- and mesotarsi which are yellow-brown. All femora and tibiae with long, loose yellow pile. Proleg (Fig. 158): Femur with a loose, yellow apical pile brush interspersed with some long black pile; very short thick pile and longer thin black pile at proximal 1/4 ventrally. Tibia with long, black pile, except dorsally. Basitarsus black pilose dorsally, with tuft of black pile on posterior side, short orange pilose ventrally. Other tarsi black pilose dorsally, short orange pilose ventrally. Mesoleg: Femur long yellow pilose posteriorly and posterodorsally, except at distal end where pile is black; short black pilose anteriorly and anterodorsally; ventrally with longer black pile. Tibia long black and short yellow pilose. Tarsi short black pilose dorsally; short yellow pilose ventrally with a few thick black spines. Metaleg (Fig. 190): Femur very slender; covered with long, thin yellow pile; shorter black pile on ventral 1/4 and at distal end. Tibia with a deep posterior depression in the proximal half which is extended as a groove on the ventral side, demarcated with short, dense black pile; proximal half dorsoventrally flattened; predominantly black pilose. Tarsi black pilose dorsally; orange pilose ventrally.

***Wing*** (Fig. 133). Entire wing uniformly dense microtrichose.

***Abdomen*** (Fig. 91). Tergite II with pair of very large, yellow triangular maculae; yellow pilose; black marking hourglass-shaped; posterior black marking equal in size or somewhat narrower than anterior black marking, but more vague because of medial white pollinosity; posterior black marking with black pile that posterolaterally extends into the yellow maculae. Tergite III with yellow fascia of variable size, occupying approx. the entire tergite; yellow pilose; with posterior, triangular black marking; black pilose; white pollinose. Tergite IV black; white pilose and white pollinose on anterior fascia, black and yellow pilose on remainder of tergite.

***Genitalia*** (Fig. 213). Epandrium: Dorsal lobe of surstylus somewhat elongated, broadly rounded, with short, black spines on almost entire surface; dorsally long yellow pilose. Ventral lobe of surstylus straight; bare.

##### Female.

Unknown.

##### Distribution.

Malawi, Senegal and South Africa.

##### Comments.

Curran (1927) cites several specimens from three localities in the Democratic Republic of the Congo. In a later publication, Curran (1939) suggests that these specimens belong to *M. tarsatus.*Bezzi (1915) also mentions the species from Uganda and Durban in South Africa, but Curran (1939) also considers these specimens as *M. tarsatus*. We have not encountered females that could be associated with the male of *M. ingratus*. No DNA barcodes are available for *M. ingratus*.

#### Mesembrius longipilosus

Taxon classificationAnimaliaDipteraSyrphidae

Jordaens, Goergen & De Meyer sp. nov.

EB0E1ADA-963B-5287-991F-97C8F5258A02

http://zoobank.org/F22BE141-7E79-4C9B-995B-DCA4B9BCC126

Figs 12 , 55 , 92 , 134 , 162 , 214

##### Differential diagnosis.

*Mesembrius longipilosus* sp. nov. males lack an apical pile brush on the profemur and have an unmodified metatibia. The proximal ventral section of the profemur has 3–4 long black pile and the metafemur is covered with long, thin yellow pile and some shorter and thicker black pile on the ventral side, except on the extreme distal end where the black and yellow pile is equally long. The pair of maculae on tergite II are very large and rounded. The species resembles *M. senegalensis*, but differs in the shape of the maculae on tergite II (rounded in *M. longipilosus* sp. nov.; rectangular in *M. senegalensis*) and the presence of some long black pile on the proximal ventral side of the metafemur (absent in *M. senegalensis*). The female is unknown.

##### Examined material.

*Mesembrius longipilosus* Jordaens, Goergen & De Meyer: Holotype, male “HOLOTYPUS” “Entebbe, UGANDA // 2.III.1972 // H. Falke // In forest” “Mesembrius // sp. 7 // Det J.R. Vockeroth” “Mesembrius longipilosus // Det. K. Jordaens, 2019” “Barcode of Life // DNA voucher specimen // Smple | CNCDIPTERA 102305 // BOLD Proc. ID: CNCDB1109-11” “CNCDIPTERA // # 102305” [CNC].

***Paratype*:** Uganda • 1♂; near Entebbe; 23–31 Jan 1972; 1160 m; H. Falke leg.; CNCDiptera 102306 (head and abdomen lost) [CNC].

##### Description male

**(Fig. 12).** Body length: 8.6 mm. Wing length: 7.2–7.7 mm.

***Head*** (Fig. 55). Eyes bare; dichoptic, distance between eyes approx. the width of ocellus. Face yellow with dark medial vitta; white pilose; white pollinose. Vertical triangle black with yellow and black pile; yellow pollinose on lower half. Distance between lateral ocellus and eye margin approx. 1/2 width of ocellus. Frontal triangle black; white pilose; white pollinose. Frontal prominence shiny black. Occiput yellow; yellow pilose with interspersed short, black setulae; yellow and white pollinose. Antenna black; postpedicel white pollinose, antennal arista reddish-brown.

***Thorax.*** Scutum black; white pilose, with three dorsal, well-demarcated yellow-white pollinose vitta which are connected at posterior end; with lateral, yellow-white pollinose vitta. Scutellum yellow-brown; yellow pilose.

***Legs.*** Femora dark chocolate-brown, tibia and tarsi orange-brown. Proleg (Fig. 162): Femur without apical pile brush; yellow pile ventrally long in proximal half, shorter in distal half; ventrally 3–4 black pile at basal 1/3. Tibia yellow and black pilose. Tarsi black pilose dorsally, yellow-orange pilose ventrally. Mesoleg: Similar to proleg; with black and yellow pile on basitarsus. Metaleg: Femur with long yellow pile anteriorly and shorter pile, posteriorly; ventral pile scarce, yellow and black, the black pile is longer at proximal half than at distal half. Tibia unmodified; with long, yellowish pile and scarce black pile on distal half. Tarsi black pilose dorsally, orange pilose ventrally.

***Wing*** (Fig. 134). Entire wing uniformly dense microtrichose.

***Abdomen*** (Fig. 92). Tergite II with pair of very large, yellow to orange rounded maculae; black marking hourglass-shaped, but the posterior part is smaller compared to the anterior part; yellow pilose. Tergite III almost entirely orange with small medial black maculae; yellow pilose. Tergite IV with anterior white pollinose band; black on posterior 1/3; yellow pilose.

***Genitalia*** (Fig. 214). Epandrium: Dorsal lobe of surstylus elongated, more or less rectangular with upwardly curved apex, with short, black spines in distal half; dorsolaterally with a few longer, black setulae; dorsally and laterally with long, yellow pile. Ventral lobe of surstylus with large expansion ventrally, with, on ventral side of the expansion, short black setulae.

##### Female.

Unknown.

##### Distribution.

Uganda.

##### Comments.

*Mesembrius longipilosus* sp. nov. is a new species which is only known from two male specimens from Uganda (Entebbe). The male genitalia look similar to those of *M. capensis*, but the black setulae on the ventral expansion of the ventral lobe of the surstylus are fewer in number and much shorter. No DNA barcodes are available.

##### Etymology.

The specific epithet *longipilosus* (Latin for hairy, covered with long pili) refers to the long, thin yellow pile on the metalegs. It is to be treated as an adjective (nominative singular masculine).

#### Mesembrius maculifer

Taxon classificationAnimaliaDipteraSyrphidae

Hull, 1941

685FFD53-922E-5405-B768-92715A036447

Figs 33 , 73 , 114 , 135

Mesembrius maculifera Hull, 1941: 332.
Mesembrius maculifer – Smith and Vockeroth (1980): 504.

##### Differential diagnosis.

The female of *Mesembrius maculifer* cannot be confused with any other species by the reddish-brown colour of tergite II with a pair of cream-coloured slender maculae. The male is unknown.

##### Examined material.

*Mesembrius maculifera* Hull: Holotype, female, “Oriental forest // Fanovana, Dist. // Fianarantsoa // Madagascar” “I-V, 1937 // C Lamberton” “TYPE 6595 // Mesembrius // maculifera // F.M. Hull” “Mesembrius // maculifera // Hull n.sp.” [ANSP]

***Paratype*:** Madagascar • 1♀; Oriental Forest, Fanovana; date and collector unknown; CNC.

##### Re-description female

**(Fig. 33).** Body length: 11.2 mm. Wing length: 8.7 mm. (Only female paratype measured).

***Head*** (Fig. 73). Eyes bare, dichoptic. Face chocolate-brown to mahogany-red. Frons with ventral 2/3 light yellow-brown pilose. Face very short reddish pubescent; sparsely long pale pilose. Ocellar triangle bare. Frontal triangle with a transverse band of dark pile. Occiput chocolate-brown to mahogany-red; thick dark brown pilose. Antenna dark reddish-brown; arista paler.

***Thorax.*** Dull black; lateral margins and scutellum very dark red; pleurites black; reddish-orange pilose. Scutellum dull black; white pilose.

***Legs.*** Predominantly black, the distal part of the pro- and mesolegs dark red; all tibiae dark red; all tarsi black dorsally, the hind pair rather flattened. Pile pale whitish on tarsi and tibiae.

***Wing*** (Fig. 135). Proximal half of the wing deep brown, especially at the r-m crossvein.

***Abdomen*** (Fig. 114). Dull black. Tergite II with a pair of prominent light-yellow maculae; white pilose anteriorly, light reddish pilose posteriorly. Tergite III and IV dark brown to black; light reddish pilose, but blackish on the middle of the posterior part.

##### Male.

Unknown.

##### Distribution.

Madagascar.

#### Mesembrius madagascariensis

Taxon classificationAnimaliaDipteraSyrphidae

Keiser, 1971

D68C114E-ABBE-5067-8F15-29F49480749B

Figs 13 , 34 , 56 , 93 , 115 , 136 , 180 , 215

Mesembrius madagascariensis Keiser, 1971: 261.
Mesembrius madagascariensis – Smith and Vockeroth (1980): 504.

##### Differential diagnosis.

*Mesembrius madagascariensis* males lack an apical pile brush on the profemur and have an unmodified metatibia. The profemur is dorsally flattened and the metabasitarsus does not have a lobe as in some males of *M. simplicipes*. The male of *M. madagascariensis* cannot be confused with any other species by the strong difference in the pile colour and length between the proximal and distal part of the mesotibia which is long and yellow pile in the proximal 2/3 and short and black in the distal 1/3. The female of *M. madagascariensis* can be distinguished from any other species (except from *M. simplicipes*) by the nearly black abdomen (clearly yellow to orange and black in other species). It can be distinguished from *M. simplicipes* by the pro- and mesolegs which are extensively brown and black (reddish-brown in *M. simplicipes*).

##### Examined material.

*Mesembrius madagascariensis* Keiser: Holotype, male, Madagascar, Antananarivo, M, 13 Dec 1957, F. Keiser (MNHN: type not studied; see comments).

*Mesembrius madagascariensis* Keiser: Allotype, female, “ALLO // TYPUS” “Madagascar. TAN. // Tananarive // 13.XII.1957 // F. KEISER” “Mesembrius // madagascar- // iensis” [NMB].

***Paratypes*:** Madagascar • 7♂♂ 21♀; Tananarive; 13 Dec 1975; F. Keiser leg.; NMB • 1♀; Lac Kavitaha, Ampefy; 20 Mar 1958; F. Keiser; NMB • 4♂♂ 2♀♀; Ambalavao, Fianarantsoa; 28–29 Jan 1958; F. Keiser; NMB • 9♂♂ 2♀♀; Tzimbazaza Park, Antananarivo; 7–8 Feb 1968; F. Keiser leg.; NMB • 1♀; Station Agric., Alaotra, District Anbatondrakzaka; 24 Dec 1957; B.R. Stuckenberg leg.; NMSA • 4♂♂ 2♀♀; Antananarivo; 1 Jan 1958; B.R. Stuckenberg leg.; NMSA.

##### Other material

 Madagascar • 1♀; Antananarivo, Tsimbazaza; 16 Oct 1993; M. Hauser leg.; CAS • 1♂; Antananarivo, Tsimbazaza; 16–22 Oct 1993; C. Kassebeer leg.; CAS • 1♂ 1♀; Antananarivo, Tsimbazaza; 16–22 Oct 1993; C. Kassebeer leg.; CNC • 1♂; Antananarivo, Tsimbazaza; 14 Oct 1993; C. Kassebeer leg.; NHMUK • 1♀; Antananarivo, Tsimbazaza; 26 Oct 1993; C. Kassebeer leg.; NHMUK • 1♀; Atsimo, Andrefana; 6–16 Jul 2012; M. Irwin and R. Harin’Hala leg.; CAS • 1♀; Atsimo, Andrefana; 31 May 2012; M. Irwin and R. Harin’Hala leg.; CAS • 1♀; Tananarive; 13 Dec 1957; F. Keiser leg.; NMB • 1♀; Tananarive; 15 Dec 1957; F. Keiser leg.; NMB • 1♂ 1♀; Tananarive; 29 Dec 1957; F. Keiser leg.; NMB • 1♀; Tananarive; 17 Apr 1958; F. Keiser leg.; NMB • 2♀♀; Tananarive; 26–30 Apr 1968; K.M. Guichard leg.; NHMUK.

##### Re-description male

**(Fig. 13).** Body length: 13.7–14.6 mm. Wing length: 9.6–10.5 mm.

***Head*** (Fig. 56). Eyes bare; holoptic, length of eye contiguity approx. length of ocellar triangle. Face white with dark medial vitta; white pilose; white pollinose. Vertical triangle black pilose on ventral half and at ocellar triangle, yellow pilose on dorsal half; yellow pollinose until just before anterior ocellus. Lateral ocellus touching eye margin. Occiput yellow; yellow pilose; yellow and white pollinose. Frontal prominence shiny black; black pilose. Antenna, scape and pedicel reddish-brown; postpedicel black; antennal arista reddish-brown.

***Thorax.*** Scutum black; dorsally with a pair of well-demarcated yellow vittae and a faint yellow medial line; vittae and medial line become faint posteriorly; yellow vague lateral vitta; yellow pilose. Scutellum uniformly yellow-brown; yellow pilose.

***Legs.*** All legs dark chocolate-brown to black, except for pro- and mesotibiae and basitarsi which are reddish-brown. Proleg: Femur dark chocolate-brown to black; without apical pile brush; pile inconspicuous, except for long yellow pile on the anterodorsal side which, dorsally, is bordered with a row of largely spaced black pile. Tibia reddish-brown; yellow pilose with some short black pile on distal end dorsally. Basitarsus orange-brown; black pilose with some longer yellow pile posteriorly. Other tarsi black; black pilose, with some longer yellow pile posteriorly, except on the two most distal tarsi. Mesoleg (Fig. 180): Femur with predominantly long yellow pile on ventral and posteroventral side, with shorter black pile on anterodorsal side. Tibia reddish-brown; proximal half with a comb of long, curved yellow pile on proximal half; with much shorter, yellow and black pile on distal half. Basitarsus orange-brown; black pilose with some longer yellow pile posteriorly. Other tarsi black; black pilose, with some longer yellow pile posteriorly, except on the two most distal tarsi. Metaleg: Femur dark chocolate-brown to black; with long, thin yellow pile, especially anteriorly; ventrally with less black pile. Tibia dark chocolate-brown to black; unmodified; with short, black pile throughout and short, yellow pile on anteroproximal half. Basitarsus and second tarsomere orange-brown, other tarsi black; black pilose.

***Wing*** (Fig. 136). Entire wing uniformly microtrichose.

***Abdomen*** (Fig. 93). Tergite II with a pair of yellow-orange, rounded maculae; black marking broadly hourglass-shaped; yellow pilose, except for posterior half of posterior black marking where pile is black; with a stretch of yellow pollinosity on anterior part of black marking. Tergite III with a pair of small, anterolateral maculae; yellow-white pilose; white pollinose on posterior half. Tergite IV dark chocolate-brown; yellow-white pilose; white pollinose.

***Genitalia*** (Fig. 215). Epandrium: Dorsal lobe of surstylus club-shaped; distal end densely covered with black spines; proximal half (‘stalk’) long yellow pilose dorsally and with some shorter, thicker black pile laterally. Ventral lobe of surstylus with one very long and thick black setula distally.

##### Re-description female

(Fig. 34). Body length: 12.5–13.5 mm. Wing length: 8.7–9.3 mm.

***Head*.** Eyes bare; dichoptic. Face white with dark medial vitta; white pilose, white pollinose. Frons black, but strongly white pollinose on ventral 4/5; black pilose on ocellar triangle and just ventral of ocellar triangle; white pilose. Distance between lateral ocellus and eye margin approx. 1½x width of ocellus. Occiput yellow-white; yellow-white pilose; yellow-white pollinose. Frontal prominence shiny black, orange-brown at distal end. Antenna, scape and pedicel orange-brown; postpedicel black, white pollinose; antennal arista yellow-orange.

***Thorax.*** Scutum dark brown to black with a pair of dorsolateral white-grey pollinose vittae; with lateral grey pollinose vague vitta; yellow and black pilose. Scutellum yellow-brown; yellow and black pilose.

**Figures 46–51.**
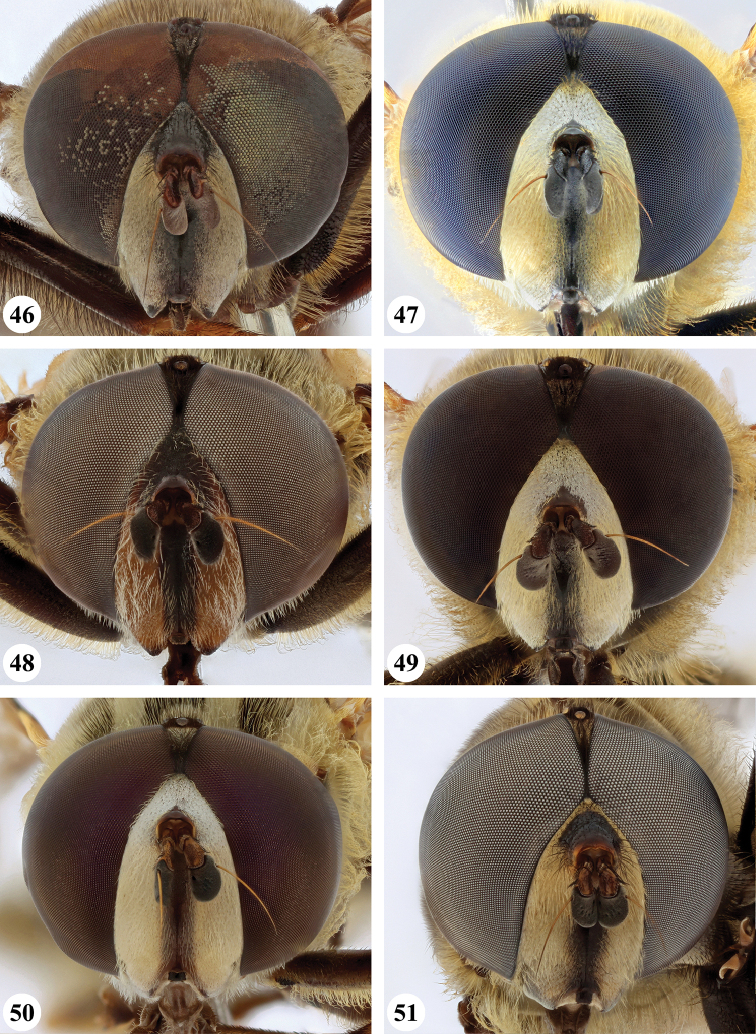
*Mesembrius* spp., head, frontal view **46***M. arcuatus* sp. nov. (♂) **47***M. caffer* (Loew) (nominal morph) (♂) **48***M. caffer* (Loew) (spined morph) (♂) **49***M. ctenifer* Hull syn. nov. (♂) **50***M. capensis* (Macquart) (♂) **51***M. chapini* Curran (♂).

***Legs.*** Pro- and mesoleg: Femur black, distal end reddish-brown; short black and longer yellow pilose. Tibia reddish-brown; yellow and black pilose dorsally; yellow pilose ventrally. Basitarsus reddish-brown; black pilose dorsally; orange-yellow pilose ventrally. Other tarsi black; black pilose dorsally, orange-yellow pilose ventrally. Metaleg: Femur black, distal 1/5 reddish-brown; yellow-white pilose, with scarce very short black pile ventrally. Tibia reddish-brown; white pilose, short black pilose posteriorly. Basitarsus dark reddish-brown; black pilose dorsally, yellow and black pilose ventrally. Other tarsi black; predominantly black pilose dorsally, densely yellow-orange pilose ventrally.

**Figures 52–57.**
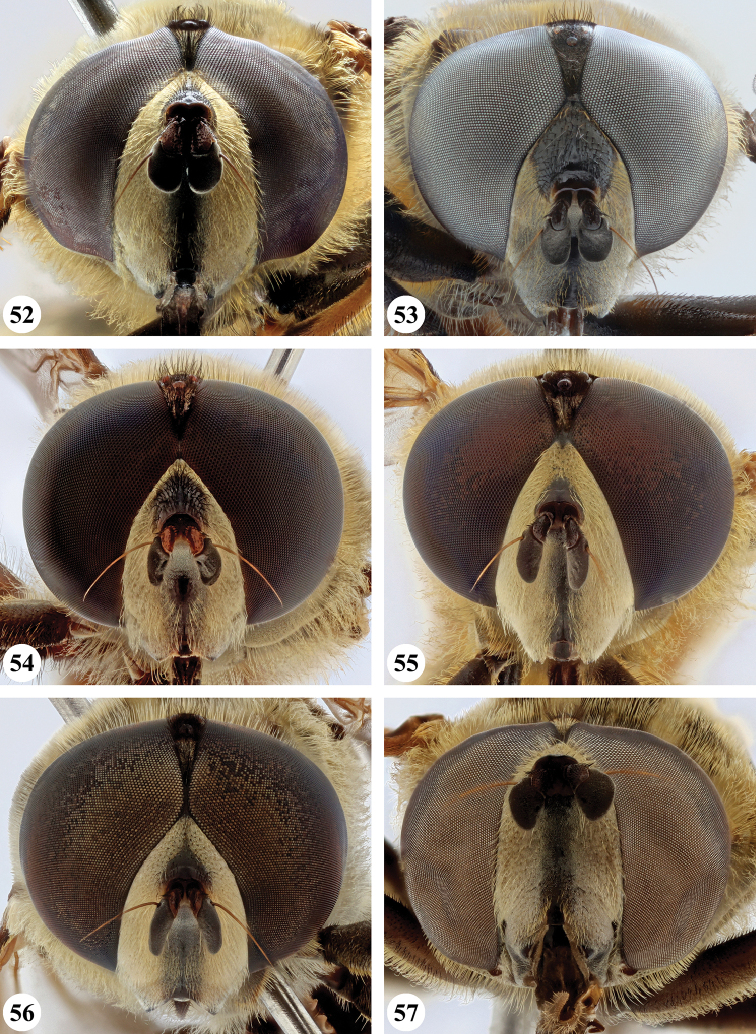
*Mesembrius* spp., head, frontal view **52***M. copelandi* sp. nov. (♂) **53***M. cyanipennis* (Bezzi) (♂) **54***M. ingratus* (Loew) (♂) **55***M. longipilosus* sp. nov. (♂) **56***M. madagascariensis* Keiser (♂) **57***M. minor* (Bezzi) (♂).

***Wing.*** Entire wing uniformly microtrichose.

**Figures 58–63.**
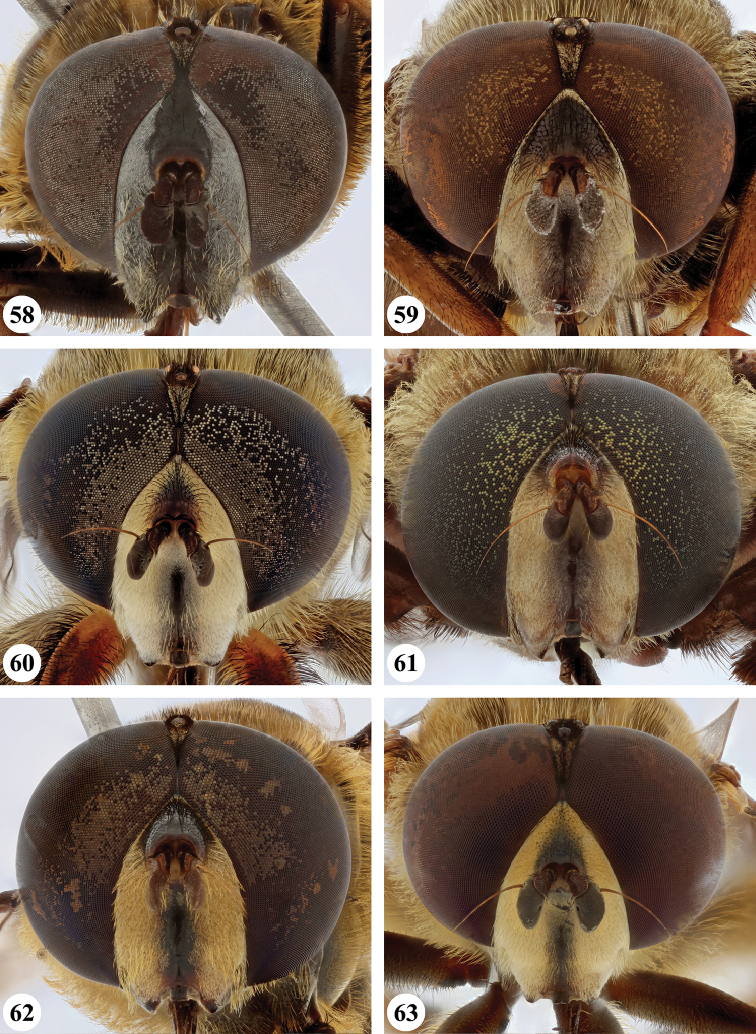
*Mesembrius* spp., head, frontal view **58***M. nigriceps* Curran (♂) **59***M. perforatus* (Speiser) (♂) **60***M. platytarsis* Curran syn. nov. (♂) **61***M. regulus* (Hull) (♂) **62***M. rex* Curran (♂) **63***M. senegalensis* (Macquart) (♂).

***Abdomen*** (Fig. 115). Dark brown to black; largely white pollinose, except for anterior border and medial area of tergite II and posterior half of tergite V; short black and white pilose, except for non-pollinose areas with white pilose only.

**Figures 64–69.**
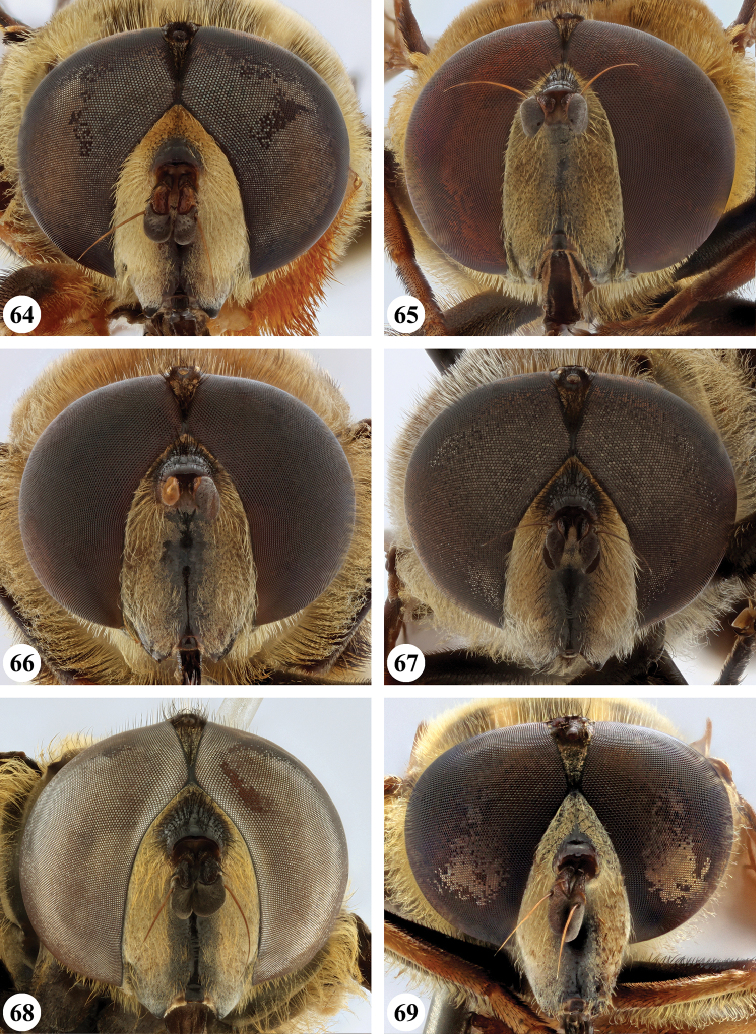
*Mesembrius* spp., head, frontal view **64***M. simplicipes* Curran (♂) **65***M. strigilatus* (Bezzi) (♂) **66***M. sulcus* sp. nov. (♂) **67***M. tarsatus* (Bigot) (♂) **68***M. tibialis* sp. nov. (♂) **69***M. vockerothi* sp. nov. (♂).

##### Distribution.

Madagascar.

**Figures 70–74.**
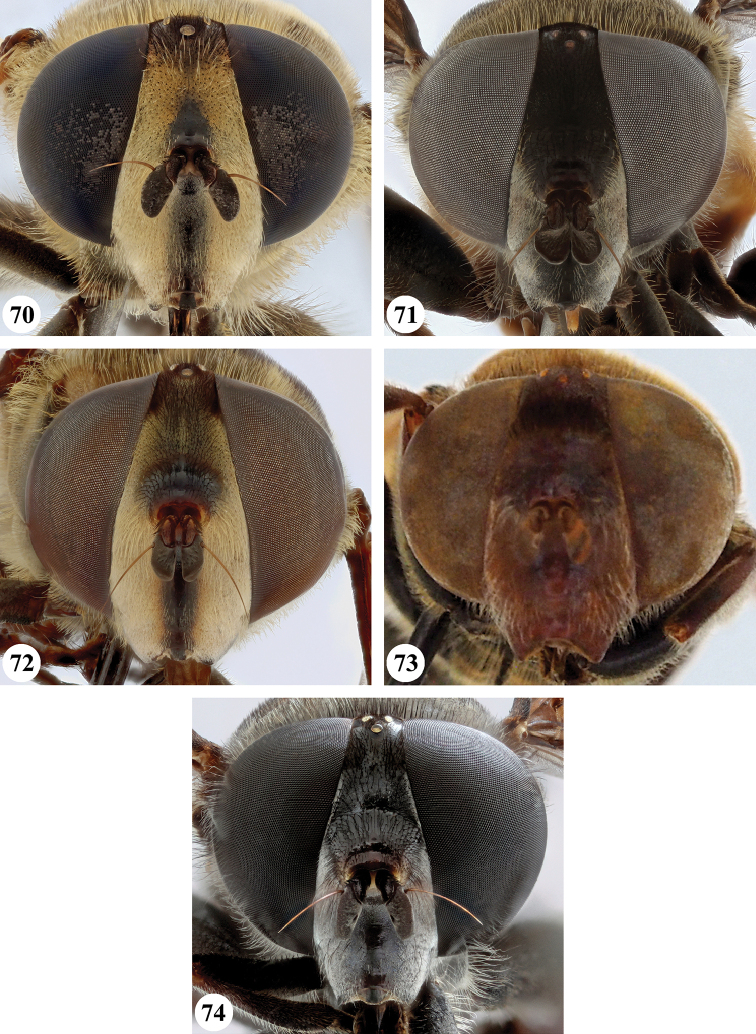
*Mesembrius* spp., head, frontal view **70***M. capensis* (Macquart) (♀) **71***M. cyanipennis* (Bezzi) (♀) **72***M. chapini* Curran (♀) **73***M. maculifer* Hull (♀) **74***M. morio* (Bezzi) (♀).

##### Comments.

The type series comprises more than 50 specimens of both sexes collected from a dozen of sites from the central and eastern domains of Madagascar (Keiser 1971). The holotype of the species should be in the collection at MNHN (Keiser 1971), but neither could we trace the holotype in the MNHN collection, nor is it listed on the MNHN entomology collection webpages.

**Figures 75–78.**
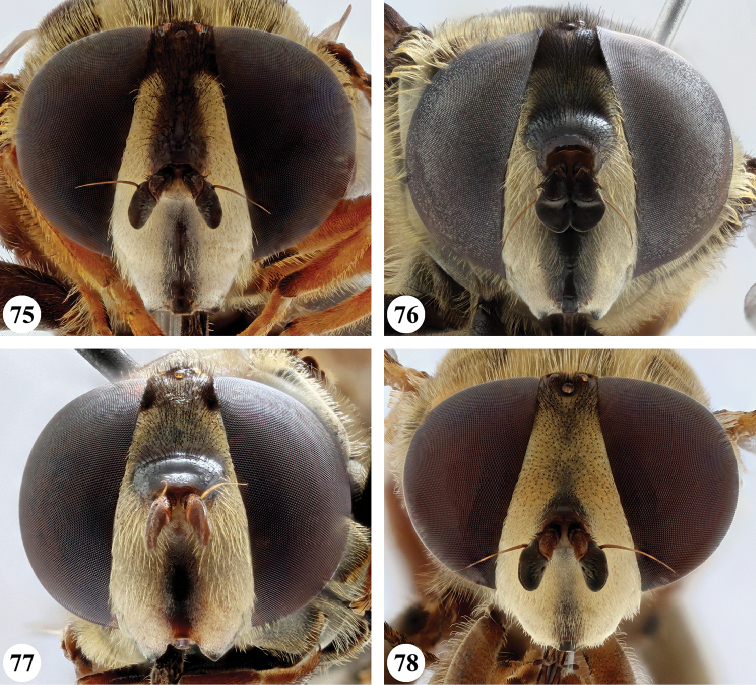
*Mesembrius* spp., head, frontal view **75***M. platytarsis* Curran syn. nov. (♀) **76***M. regulus* (Hull) (♂) **77***M. rex* Curran (♂) **78***M. senegalensis* (Macquart) (♂).

**Figures 79–82.**
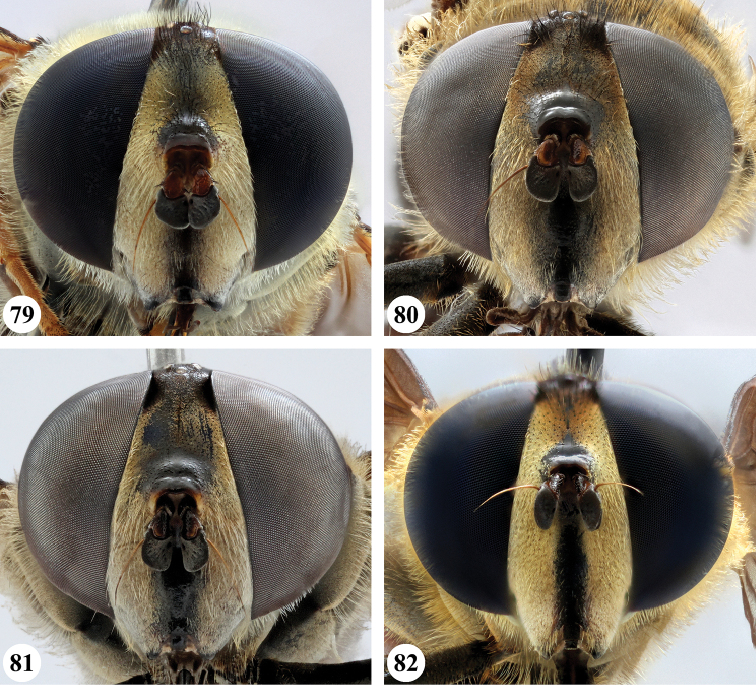
*Mesembrius* spp., head, frontal view **79***M. simplicipes* Curran (♂) **80***M. sulcus* sp. nov. (♀) **81***M. tarsatus* (Bigot) (♀) **82***M. vockerothi* sp. nov. (♀).

#### Mesembrius minor

Taxon classificationAnimaliaDipteraSyrphidae

(Bezzi, 1915)

918A816B-E60C-525D-8099-4D5B7E1A1DDE

Figs 14 , 35 , 57 , 94 , 116 , 137 , 173 , 198 , 204 , 216

Helophilus (Mesembrius) minor Bezzi, 1915: 96.
Mesembrius minor – Bezzi (1921): 7 – Bezzi (1923): 348 – Curran (1927): 64 – Curran (1939): 10 – Smith and Vockeroth (1980): 504.

##### Differential diagnosis.

*Mesembrius minor* males lack an apical pile brush on the profemur and have an unmodified metatibia. The metafemur has a large patch of black pile at the proximal end and, distally, a smaller patch of black pile on the posteroventral side with, anterior to this, a small swelling. The metatibia is unmodified. The maculae on tergite II are very large and rounded and with a narrow black medial vitta. Males differ from males of *M. strigilatus* in the straight metafemur and metatibia (curved in *M. strigilatus*) and from males of other species that lack an apical pile brush in the profemur which has very conspicuous and dense black pile on the ventral side of the metafemur (yellow and less dense in the other species). Females have a frons which is pale pilose on ventral half. Tergite II has a pair of yellow maculae (fascia in *M. capensis* and spined morph of *M. caffer*) and the black posterior marking does not extend to the lateral margins (extends to lateral margins in *M. strigilatus*). The metafemur has a marked ventral swelling in the middle (absent in other species; present in *M. regulus*, but in this species, the pile on the frons is black pilose on the ventral half, except laterally). The pro- and mesotarsi are brown with a darkened medial part, except in the basitarsus.

##### Examined material.

*Helophilus minor* Bezzi: Lectotype (hereby designated), male, “LECTOTYPUS” “Hel. (Mes.)// ♂, Type//minor//Bezzi” “Syn-//type” “Pres. By//Impl. Bureau Ent.//1915-165.” “Chintechi//Nyasaland/; Dr.H.S. Stannus” “Mesembrius//minor n.sp.//Type ♂” “NHMUK 013428949” [NHMUK]. Female, “Syn-//type” “Hel. (Mes.)// ♀, Type//minor//Bezzi”; “Brit. E. Africa//N. of Mt. Kenia,//nr. crater lake,//5700 ft.//T.J. Anderson.// 15.II.1911”; “Pres. By//Impl. Bureau Ent.//1915-165.”; “Mesembrius//minor n.sp.//Type ♀”; “NHMUK 013428950” [NHMUK]. Female, “Syn-//type” “Brit. E. Africa//N. of Mt. Kenia,//nr. crater lake,//5700 ft.//T.J. Anderson.// 15.II.1911” “H.(M.) minor Bezzi.//Bezzi det.//1915.” “Pres. By//Impl. Bureau Ent.//1915-165.” “NHMUK 013428951” [NHMUK].

##### Other material

 Benin • 5♀♀; Azaourissé; 7 Mar 2018; K. Jordaens leg.; KMMA • 1♂; Cotonou; Feb 2003; G. Goergen; IITA • 1♂; Cotonou; 14 Dec 2013; G. Goergen and K. Jordaens leg.; KMMA • 4♂♂ 5♀♀; Cotonou; 28 Jan 2016; K. Jordaens and G. Goergen leg.; KMMA • 1♀; Ouidah; Sep 2004; G. Goergen leg.; IITA • 1♂; Pobé; 16 Mar 2014; G. Goergen leg.; KMMA • 1♂; Sedjé; Sep 2012; G. Goergen leg.; IITA. Cameroon • 1♀; Maroua, Meskina; 23 May 2018; M. Azo’o Ela leg.; MAPC • 1♂; Douala; 9 Jul 1974; J.A.W. Lucas leg.; RMNH. Chad • 1♀; Bebedja; date unknown; F.A. Bink and R.M. Bink-Moenen leg.; RMNH. Democratic Republic of the Congo • 1♂; Kongo-Central, Boma; 16 Jun 1915; Lang and Chapin leg.; KMMA • 1♂; Haut-Lomami, Kitompo, Fungwi; 18 Jun 1911; Dr. Bequaert leg.; KMMA. Mozambique • 1♂; Lower Shire R.; 24 Jun 1916; R.C. Wood leg.; NHMUK.

##### Re-description male

**(Fig. 14).** Body length: 11.8–13.3 mm. Wing length: 8.0–9.8 mm.

***Head*** (Fig. 57). Eyes bare; slightly dichoptic, distance between eyes approx. the width of ocellus. Face white with dark medial vitta; white pilose; white pollinose. Vertical triangle black pilose; white pollinose in area before ocellar triangle. Distance between lateral ocellus and eye margin less than 1/2 width of ocellus. Occiput yellow; yellow pilose; yellow and white pollinose. Frontal triangle dark black pilose; strongly yellow pollinose on ventral half. Frontal prominence shiny black. Antenna black; antennal arista brown.

***Thorax.*** Scutum black with, dorsally, a pair of well-demarcated yellow vittae and a faint yellow medial line; vittae and line are connected anteriorly and posteriorly; with lateral, yellow vitta; yellow-white pilose. Scutellum uniformly yellow-brown; yellow pilose.

***Legs.*** Femora and entire metaleg brown; pro- and mesofemora and tarsi yellow-brown; small darkened medial patch in all tarsi, except for the basitarsus. Proleg: Femur without apical pile brush; yellow pilose on anterior and ventral side; with shorter, black pile on dorsal and posterior side. Mesoleg: Femur similar to profemur, but also with a row on longer black pile ventrally. Metaleg (Fig. 198): Femur with long, yellow pile on anterior side; with a basoventral patch of long, black pile and, somewhat more distally, a smaller patch of long, black pile on the posteroventral side; anterior to this black pile, a small swelling. Tibia unmodified; almost straight.

***Wing*** (Fig. 137). Entire wing uniformly dense microtrichose.

***Abdomen*** (Fig. 94). Tergite II with pair of very large yellow rounded maculae; black marking hourglass-shaped, with anterior black marking larger than posterior black marking, the latter not reaching the lateral sides of the tergite. Medial black vitta more or less parallel-sided. Black tergal marking with short stiff black pile which does not extend to the lateral sides. Tergite III with small triangular to rounded black marking. Tergite IV with very large rounded black marking occupying most of the tergite; narrowly white pollinose anteriorly. The yellow pile of the abdomen is very short, except on tergite V, where it is not appressed to the lateral sides of the tergite. Tergite V black; strongly white pollinose.

***Genitalia*** (Fig. 216). Epandrium: Dorsal lobe of surstylus short, nearly circular; with short black spines on almost entire surface; long yellow pilose dorsally. Ventral lobe of surstylus straight; bare. Hypandrium markedly downwardly curved.

##### Re-description female

**(Fig. 35).** Body length: 10.6–13.5 mm. Wing length: 7.5–9.4 mm.

***Head.*** Eyes bare; dichoptic. Face white with dark medial vitta; white pilose, white pollinose. Frons black on dorsal 2/5, yellow-white on ventral 3/5; black and white pilose on ocellar triangle and just ventrally of ocellar triangle, otherwise white pilose; strongly white pollinose on ventral 3/5, weak white pollinose on dorsal 2/5. Distance between lateral ocellus and eye margin slightly less than width of ocellus. Occiput yellow-white; yellow-white pilose; yellow-white pollinose. Frontal prominence shiny black. Antenna dark brown to black; antennal arista reddish-brown.

***Thorax.*** Scutum dark brown with a pair of dorsolateral yellow pollinose vittae which are connected posteriorly; with lateral, yellow pollinose vitta; sometimes with a fine medial white to yellow pollinose vita; yellow pilose. Scutellum yellow-orange; yellow pilose.

***Legs.*** Proleg (Fig. 173): Femur black, distal end orange-brown; yellow pilose, with short black pile interspersed. Tibia orange, darkened in distal 1/2; yellow pilose on dorsal proximal half, yellow and black pilose otherwise. Tarsi orange-brown with darkened medial area; black pilose dorsally, yellow pilose ventrally; especially the posterior side has very conspicuous thick black pile. Mesoleg: Femur black, distal end orange-brown; black and white pilose. Tibia orange-brown; orange-yellow pilose on dorsal side, black pilose on ventral side. Tarsi orange-brown with darkened dorsal medial area; black pilose. Metaleg: Femur black, distal end orange-brown; orange-yellow pilose, with short, black pile on dorsal distal end, with shorter and thicker black pile on ventral distal half; with marked ventral swelling in middle (Fig. 204). Tibia orange-brown; orange-yellow pilose with some black pile interspersed at distal end. Tarsi black dorsally, orange ventrally; black pilose dorsally; densely orange pilose ventrally.

***Wing.*** Entire wing uniformly microtrichose.

***Abdomen*** (Fig. 116). Tergite II with a pair of very large, rounded yellow-orange maculae; black pilose on triangular posteromedial section, yellow pilose otherwise; posterior black marking does not reach the lateral tergal sides; medial part of black marking narrow, approx. 1/10 of tergal width; posterior black marking white pollinose. Tergite III with yellow-orange fascia which occupies entire tergal length on lateral sides and approx. 1/3 of tergal length in medial section; with triangular posterior black marking that does not reach the lateral tergal sides; black pilose on triangular posteromedial section, yellow pilose otherwise; posterior black marking white pollinose. Tergite IV as tergite III but with much narrower yellow-orange fascia (approx. 1/10 of tergal length in medial section). Tergite V with narrow anterior black marking; with a pair of yellow-orange maculae in anterolateral corners, otherwise black; yellow pilose; black marking strongly white pollinose, especially in anterior half.

##### Distribution.

Benin, Cameroon, Chad, Democratic Republic of the Congo, Malawi and Mozambique.

##### Comments.

Two female syntypes at the NHMUK (NHMUK-0103428950 and NHMUK-0103428951) are not *M. minor* as both lack the ventral swelling on the metafemur, the black marking on tergite II is not of the typical hourglass shape and the short black spines on the ventral side of the metafemur are restricted to the proximal half. Syntype NHMUK-0103428950 corresponds to the female of *M. capensis*, while syntype NHMUK-0103428951 corresponds to the female of either *M. strigilatus* or *M. caffer*.

#### Mesembrius morio

Taxon classificationAnimaliaDipteraSyrphidae

(Bezzi, 1915)

8B3481DF-E273-5475-A417-BB3469539B9A

Figs 36 , 74 , 117 , 138

Helophilus (Mesembrius) morio Bezzi, 1915: 98.
Mesembrius morio – Smith and Vockeroth (1980): 504.

##### Differential diagnosis.

*Mesembrius morio* females are entirely black and cannot be confused with any other *Mesembrius* species. The male is unknown.

##### Examined material.

*Helophilus morio* Bezzi: Holotype, female, “Holo-//type” “Hel. (Mes.)//Type//morio//Bezzi” “Neguelo,//Usambara,//German E. Africa//Purchd. From//H.Rolle.//1904-117.” “Mesembrius//morio n.sp.//Type ♀”; “NHMUK 013428952” [NHMUK].

***Paratype*:** Tanzania • 1♀; Usambara Mountains, Neguelo; date unknown; H. Rolle leg.; NHMUK.

##### Other material

 Democratic Republic of the Congo • 1♀; Eala; 24 Aug 1935; J. Ghesquière leg.; KBIN. Malawi • 1♀; Mount Mulanje; 6 Nov 1913; S.A. Neave leg.; NHMUK. Tanzania • 2♀♀; Neguelo, Usambara Mountains; date unknown; H. Rolle leg.; NHMUK. Uganda • 2♀♀; Entebbe; 17 Jun 1972; H. Falke leg.; CNC.

##### Re-description female

**(Fig. 36).** Body length: 12.7–13.5 mm. Wing length: 11.6–12.5 mm.

***Head*** (Fig. 74). Eyes bare; dichoptic. Face black; black and white pilose; white pollinose. Frons black; black pilose; lower half white pollinose. Vertex black; black pilose; grey pollinose. Distance between lateral ocellus and eye margin approx. the width of ocellus. Occiput black; yellow and black pilose dorsally, yellow pilose more ventrally; grey pollinose. Frontal prominence shiny brown-black; black pilose. Antenna black; arista reddish-brown.

***Thorax.*** Scutum and scutellum black; without vitta; short white and black pilose.

***Legs.*** Dark reddish-brown to black; short black and white pilose.

***Wing*** (Fig. 138). Entire wing uniformly, very densely microtrichose; dark brown in anterior half.

***Abdomen*** (Fig. 117). Entirely black; short yellow-white and black pilose.

**Male.** Unknown.

##### Distribution.

Democratic Republic of the Congo, Malawi, Tanzania and Uganda.

##### Comments.

Previously only known from the holo- and paratype. Curran (1927) considers *M. morio* to be a dark morphotype of *M. cyanipennis*. As the male of *M. morio* is unknown, we could not compare the male genitalia. However, since the differentiation between the two species with DNA barcodes (*p*-distance: 6.4%) is of the same magnitude as the differentiation between other closely related species (range *p*-distances: 4.3–14.7%; see Discussion and Fig. 229), we consider *M. morio* and *M. cyanipennis* as two different morphospecies.

#### Mesembrius nigriceps

Taxon classificationAnimaliaDipteraSyrphidae

Curran, 1927

7691ECD7-2466-5122-8DCD-1D9BE08E00EC

Figs 15 , 58 , 95 , 139 , 192 , 217

Mesembrius nigriceps Curran, 1927: 63.
Mesembrius nigriceps – Smith and Vockeroth (1980): 504.

##### Differential diagnosis.

*Mesembrius nigriceps* males lack an apical pile brush on the profemur and have a metatibia which is curved, but less than in *M. strigilatus*. The face ground colour is black (white to yellow in *M. strigilatus*). The metafemur is curved, has a patch of conspicuous black pile at the base and, perpendicular to this, a stretch of dense black pile on the ventroposterior side. The male is distinguished from any other species (except from *M. strigilatus*) by the strongly curved metafemur and metatibia. It differs from *M. strigilatus* in the colour of the face (white to yellow in *M. strigilatus*; black in *M. nigriceps*), in the size and shape of the maculae on tergite II which are small and nearly triangular (large and rounded in *M. strigilatus*) and by the broader black medial marking on tergite II (narrow in *M strigilatus*). The female is unknown.

##### Examined material.

*Mesembrius nigriceps* Curran: Holotype, male, “Mesembrius // TYPE // nigriceps // Curran” “Taken from Bembex” “Stanleyville, Cgo. // 25°10’E, 0°30’N // III.1915” “Lang & Chapin // Collectors” [AMNH]. Type studied from picture on website.

##### Other material

 Ghana • 1♂; Eastern Region, N of Kibi, Atewa Range Forest Reserve; 21 Jun 2006; K.-D.B. Dijkstra leg.; MZH. Togo • 1♂; Kloto Forest; Mar 2004; G. Goergen leg.; IITA.

##### Re-description male

**(Fig. 15).** Body length: 11.0 mm. Wing length: 8.4 mm.

***Head*** (Fig. 58). Eyes bare; slightly dichoptic, distance between eyes approx. width of ocellus. Face white with dark medial vitta; white pilose; white pollinose. Vertical triangle black; black pilose; lower half weakly white pollinose. Distance between lateral ocellus and eye margin somewhat less than width of ocellus. Frontal triangle black; white pilose; grey pollinose at the sides. Frontal prominence shiny black; black pilose. Occiput black; yellow pilose with a stretch of black pile near the eye margin; grey-white pollinose. Antenna black; antennal arista brown.

***Thorax.*** Scutum black with, dorsally, a pair of faint yellow vittae which fade out posteriorly; lateral yellow vitta very faint; yellow-rufous pilose. Scutellum uniformly yellow-brown; yellow-rufous pilose, with some short black pile interspersed, especially in the posterior half.

***Legs.*** Femora and entire metaleg dark brown to black; pro- and mesofemora and tarsi dark brown; tarsi without a small darkened medial patch. Proleg: Femur without apical pile brush; short black pilose dorsally, long black pilose ventrally, long yellow pilose posteriorly. Tibia long yellow pilose and short black pilose, except for a row of long black pile posterodorsally. Tarsi black pilose dorsally, yellow-orange pilose ventrally. Mesoleg: Femur similar to profemur, but with long, black pile on posterior and posteroventral side and with black pile on anterodorsal side which is markedly longer in the proximal half. Tibia yellow pilose ventrally, except at extreme distal end, where it is also black; short black pilose dorsally; long black pilose anterordorsally, especially in proximal 1/2. Tarsi black pilose dorsally, yellow-orange pilose ventrally, with some thick black pile on ventral side. Metaleg (Fig. 192): Femur weakly curved; thickened in distal 1/3; with long yellow pile on anterior and anteroventral side; ventrally with dense, long black pile in proximal 1/3 and less thick and less dense black pile elsewhere; no swelling on the mid-section of the ventral side. Tibia strongly curved, especially from posterior view; flattened; with very long, black pile on dorsal and ventral side. Tarsi black pilose dorsally, yellow-orange pilose ventrally.

***Wing*** (Fig. 139). Entire wing uniformly dense microtrichose.

***Abdomen*** (Fig. 95). Tergite II with a pair of large, triangular, yellow maculae; anterior and posterior black markings equal in size and with broad medial black marking; black markings with short, stiff black setulae which do not extend to the lateral sides; strongly yellow-orange pilose. Tergite III with a pair of smaller, triangular to rounded yellow maculae in anterior half; strongly yellow-orange pilose. Tergite IV black; long yellow-orange pilose, especially on lateral sides.

***Genitalia*** (Fig. 217). Epandrium: Dorsal lobe of surstylus strongly bent, sickle-shaped, short yellow pilose on distal half; with long, thick black setulae at bend ventrally; distal half dorsally broadly convex; densely covered with long yellow pile and with some equally long, but thicker black pile interspersed. Ventral lobe of surstylus bare.

##### Female.

Unknown.

##### Distribution.

Democratic Republic of the Congo, Ghana and Togo.

##### Comments.

The species is very similar in morphology to *M. strigilatus* and they are sister species in the NJ phylogenetic analysis (but no support for such relationship in the ML analysis). Compared to *M. strigilatus*, *M. nigriceps* has a black face, a less curved metatibia, the yellow maculae on tergite II are smaller and more triangular and the yellow abdominal pile on abdominal tergite IV is not so strongly appressed on the sides. The male surstylus is morphologically also similar to that of *M. strigilatus*, but the thin apex is much longer in *M. nigriceps* and the dorsal surface of the distal half is more convex in *M. nigriceps*.

#### Mesembrius perforatus

Taxon classificationAnimaliaDipteraSyrphidae

(Speiser, 1913)

42E7056D-3668-51C9-86C5-B613BCE4D966

Figs 16 , 59 , 96 , 140 , 152 , 163 , 185 , 218

Prionotomyia perforata Speiser, 1913: 129.
Mesembrius perforatus – Smith and Vockeroth (1980): 504.

##### Differential diagnosis.

*Mesembrius perforatus* males have a black apical pile brush on the profemur, the protarsi are very broad, orange and the probasitarsus has a tuft of orange pile on the posterior side. The metafemur is long and slender with black pile ventrally which becomes longer towards the distal end. The metatibia has a long and deep posterior depression which is bordered with long black pile. The species resembles other species with a dark apical pile brush on the profemur, but the probasitarsus has a tuft of orange pile as in *M. rex* from which it differs in the metafemur which is entirely covered in short black pile ventrally (with a row of short spines in *M. rex*). Other species with a dense apical pile brush have either no tuft of pile on the probasitarsus (*M. regulus*) or a tuft of black pile (other species). It is the only species which has three depressions on the posterior side of the metatibia. The female is unknown.

##### Examined material.

***Holotype***, male: Tanzania• Niussi; 17 Dec 1905; Chr. Schröder leg. (type not found/studied).

##### Other material

 Benin • 1♂; Calavi; 11 Nov 1993; G. Goergen leg.; IITA • 1♂; Calavi; Oct 2001; G. Goergen leg.; IITA. Democratic Republic of the Congo • 1♂; Elisabethville [= Lubumbashi]; Apr 1930; M. Bequaert leg.; KMMA • 1♂; Elisabethville [= Lubumbashi]; Apr 1930; M. Bequaert leg.; RMNH • 1♂; Tshibinda; 21–27 Aug 1931; W.P. Cockerell leg.; NHMUK. Kenya • 1♂; Kakamega Forest, Isecheno Station; 24 Jan 1991; Earthwatch Team 2 leg.; NMK. Uganda • 1♂; Entebbe; 27 May 1912; C.C. Gowdey leg.; NHMUK • 4♂♂; Entebbe; 7–9 May 1912; C.C. Gowdey leg.; NHMUK • 1♂; Entebbe; 21 Aug 1911; C.C. Gowdey leg.; NHMUK • 1♂; Entebbe; 18–20 Nov 1912; C.C. Gowdey leg.; NHMUK • 3♂♂; Entebbe; 7 Oct 1971; H. Falke leg.; CNC • 1♂; Entebbe; 23–31 Jan 1973; H. Falke leg.; CNC • 1♂; Entebbe; 25–27 Mar 1973; H. Falke leg.; CNC.

##### Re-description male

**(Fig. 16).** Body length: 14.6–15.7 mm. Wing length: 10.5–11.5 mm.

***Head*** (Fig. 59). Eyes bare; slightly dichoptic, distance between the eyes approx. the width of ocellus. Face yellow with dark medial vitta; white pilose; white pollinose. Vertical triangle black; black pilose; yellow pollinose on lower half. Distance between lateral ocellus and eye margin less than 1/2 width of ocellus. Occiput black; yellow pilose with black pile in dorsal area; yellow and white pollinose. Frontal triangle short; black; with some long black pile; white pollinose. Frontal prominence shiny black with orange-brown apex. Antenna, scape and postpedicel black; pedicel dark orange-black; antennal arista reddish-brown.

***Thorax.*** Scutum black with, dorsally, a pair of faint grey pollinose vittae; lateral vitta faint, not well-demarcated; black pilose with long yellow pile on anterolateral part and postpronotum. Scutellum uniformly yellow-brown; with long yellow and black pile.

***Legs.*** Proleg (Figs 152, 163): Femur black; dorsoventrally flattened; with a black apical pile brush; proximoventral section with long, thick black setae; posterior side with long golden pile. Tibia dorsally brown, ventrally orange-brown; with, especially on the ventral side, long black pile. Basitarsus very broad; orange; with a tuft of orange pile on posterior side (Fig. 163). Other tarsi very broad; orange; becoming shorter distally; the most distal tarsal segment white; sparsely black pilose dorsally, but with denser short black pile in anterior half, short orange pilose ventrally. Mesoleg: Femur dark brown; with long yellow pile on ventroproximal side, scattered yellow pile posterodorsally, but black at distal end. Tibia dorsally brown, ventrally orange-brown; with a tuft of black curved pile on ventroproximal end. Tarsi orange; sparsely black pilose dorsally, orange pilose ventrally with some thick long black pile. Metaleg (Fig. 185): Femur long and slender; dark brown; black pilose ventrally, the pile gradually becomes longer towards distal end; pile otherwise yellow and less dense. Tibia dorsally dark brown, ventrally orange-brown; in anterior view with a strong carina on the ventral side in the middle; with three deep depressions on the posterior side of the proximal half which are bordered with long, black pile, especially dorsally. Tarsi dark brown; sparsely black pilose dorsally, short orange pilose ventrally.

***Wing*** (Fig. 140). Entire wing uniformly dense microtrichose.

***Abdomen*** (Fig. 96). Tergite II with pair of large, yellow triangular maculae; black marking hourglass-shaped, white pollinose on posterior end; yellow and black pilose, but black pile most conspicuous on posterior black marking of tergite. Tergite III with broad yellow fascia and a triangular black marking on posterior half; black marking strongly white pollinose, covered with short black spines; otherwise yellow pilose. Tergite IV with large triangular posterior black marking, otherwise yellow; strongly white pollinose.

***Genitalia*** (Fig. 218). Epandrium: Dorsal lobe of surstylus short, broadly rounded, with short, black spines on almost entire surface. Ventral lobe of surstylus straight; bare.

##### Female.

Unknown.

##### Distribution.

Benin, Democratic Republic of the Congo, Kenya, Tanzania and Uganda.

##### Comments.

We could not find the male holotype in any of the surveyed collections. The male has a set of unambiguous character states mentioned in the original description and cannot be confused with any other species of the genus. The specimens we have studied correspond with the original species description and are therefore considered to be conspecific.

#### Mesembrius regulus

Taxon classificationAnimaliaDipteraSyrphidae

(Hull, 1937)

48C3F554-CBCC-5493-984B-7E0CD53C6BB6

Figs 18 , 38 , 61 , 76 , 98 , 119 , 142 , 153 , 171 , 183 , 202 , 220

Tityusia regulus Hull, 1937: 119.
Mesembrius regulus – Smith and Vockeroth (1980): 504.

##### Differential diagnosis.

*Mesembrius regulus* males have a dark brown apical pile brush on the profemur, a strongly flattened protibia with long black pile in the proximal half and long yellow-orange pile in the distal half. The species resembles other species with a dark apical pile brush on the profemur, but the probasitarsus lacks a tuft of orange or black pile as in the other species. It is the only species with a strongly flattened protibia and with very long, thick black pile on the metabasitarsus. Females have a frons which is black pilose on its entire length, except laterally. The female can be distinguished from the female of *M. sulcus* sp. nov. and *M. tarsatus* by the colour of the tibiae (yellow-brown to chocolate-brown in *M. regulus*; black in *M. sulcus* sp. nov. and *M. tarsatus*). It differs from *M. chapini* by the black pile on the protibia which is restricted to the distal half (over the entire length in *M. chapini*). It differs from the female of *M. rex* by the presence of black pile on the ventral side of the pro- and mesotibia (absent in *M. rex*), the lighter protarsus compared to the distal part of the protibia (concolourous in *M. rex*) and wing cell r_1_ which is nearly closed (distinctly open in *M. rex*).

##### Examined material.

*Tityusia regulus* Hull: Holotype, male, “Efufup // Kamerun, // W. Africa // VIII.30.1919” “Carn. Mus. //Acc. 6552” “type” “Tityusia // regulus // type Hull” “Monstromyia rex // Hull Curr.” [MCZ] [type studied from pictures].

##### Other material

 Benin • 2♂♂ 1♀; Calavi; Apr 2014; G. Goergen leg.; IITA • 1♀; Calavi; Oct 2015; G. Goergen leg.; IITA • 1♀; Ifangni-range; 6 May 2016; G. Goergen leg.; KMMA • 1♂ 2♀♀; Ifangni-range; 19 Mar 2017; G. Goergen leg.; KMMA • 1♂ 1♀; Pobé; 27 Jan 2016; G. Goergen leg.; IITA • 1♀; Porto Novo; Mar 2003; G. Goergen leg.; IITA • 1♀; Porto Novo; Dec 2005; G. Goergen leg.; IITA • 1♀; Porto Novo; Jul 2005; G. Goergen leg.; IITA • 1♀; Porto Novo; Jan 2008; G. Goergen leg.; IITA • 1♂ 2♀♀; Porto Novo; Mar 2008; G. Goergen leg.; IITA • 2♂♂; Porto Novo; 31 Jan 2014; G. Goergen leg.; KMMA • 2♀♀; Porto Novo; 27 Jan 2016; G. Goergen leg.; KMMA • 3♀♀; Porto Novo; date unknown; K. Jordaens leg.; KMMA. Democratic Republic of the Congo • 1♀; Equateur, Eala; Oct 1935; J. Ghesquière leg.; KMMA • 1♀; Equateur, Eala; Sep 1935; J. Ghesquière leg.; RMNH • 1♂; Equateur, Eala; Aug 1935; J. Ghesquière leg.; KBIN • 1♀; Equateur, Eala; J. Ghesquière leg.; KBIN • Jan 1936; J. Ghesquière leg.; KBIN • 1♀; Equateur, Lopri River; May–Jun1927; J. Ghesquière leg.; KMMA • 1♀; Terr. de Banningville, Kwilu, Panga; Aug 1945; Fain leg.; KMMA • 1♀; Tshuapa, Flandria [= Boteka]; 18 Oct 1945; P. Hulstaert leg.; KMMA • 1♀; Ubangi, Nzali; 3–4 Mar 1932; H.J. Brédo leg.; KMMA • 1♂; Uelé, Tukpwo; Jul 1937; J. Vrijdagh leg.; KMMA. Nigeria • 1♀; Lagos; 22 Nov 1911; W.A. Lamborn leg.; OXUM • 1♀; Lagos; 20 Feb 1912; W.A. Lamborn leg.; OXUM • 2♂♂; Lagos; 21 Mar 1912; W.A. Lamborn leg.; OXUM. Togo; 1♂; Kloto Forest; Feb 2008; G. Goergen leg.; IITA.

##### Re-description male

**(Fig. 18).** Body length: 21.5–24.2 mm. Wing length: 13.2–15.0 mm.

***Head*** (Fig. 61). Eyes bare; holoptic, eye contiguity as long as length of ocellar triangle. Face white with dark medial vitta; white pilose; white pollinose. Vertical triangle black; black pilose; yellow pollinose on lower half. Ocellus and eye touching. Occiput black; yellow pilose; black pilose dorsally; yellow and white pollinose. Frontal triangle short; black; with some long black pile; strongly white pollinose. Frontal prominence shiny black with orange apex. Antenna black; postpedicel strongly white pollinose; antennal arista orange-brown.

***Thorax.*** Scutum dark brown to black with, dorsally, a pair of very faint, grey pollinose vittae which fade out posteriorly; pile short, dense, black and yellow-white. Scutellum yellow-brown with darker anterior border; with dense yellow and, on the posterior half and centre, shorter, black pile.

***Legs.*** Proleg (Figs 153, 171): Femur dark brown; dorsoventrally flattened; with a dark brown apical pile brush; remainder of posterior side with less dense, long brown pile. Tibia orange-brown in proximal 1/3, but darker in distal 2/3; very broad; with brown to black pile which is longer posteriorly. Basitarsus orange-brown, longer than wide. Other tarsi orange-brown; progressively becoming shorter, wider and lighter; most distal tarsal segment greyish. Mesoleg: Femur dark brown; with long yellow pile on ventroposterior 4/5, black on distal 1/5; pile otherwise short and black. Tibia orange-brown; with long black pile ventrally and shorter, strongly curved black pile dorsally. Tarsi orange; with short, black pile. Metaleg (Figs 183, 202): Femur long and slender; orange-brown; with long yellow pile on all but ventral sides, except for long black pile at extreme distal end; pile much shorter and black ventrally. Tibia orange-brown; with brown to black long pile; unmodified. Basitarsus orange-brown; with a very conspicuous thick tuft of very long and very dense brown pile on distal dorsal half; with long brown pile at extreme proximal end ventrally. Second tarsomere orange-brown; with long brown pile posteriorly. Other tarsi orange-brown; sparsely black pilose dorsally, short orange-brown pilose ventrally.

***Wing*** (Fig. 142). Entire wing uniformly dense microtrichose.

***Abdomen*** (Fig. 98). Tergite II with a pair of large yellow, rounded maculae; black marking hourglass-shaped; yellow pilose in anterior half and along tergite margins, black pilose in posterior half; black marking white pollinose posteriorly. Tergite III with yellow fascia and a triangular black marking on posterior half which is strongly white pollinose; yellow pilose on anterior 1/3 and along tergite margins, black pilose on posterior 2/3. Tergite IV dark brown to black; yellow-white pilose, but with shorter, black pile medially; strongly white pollinose on anterior 1/3 to 1/2.

**Figures 83–88.**
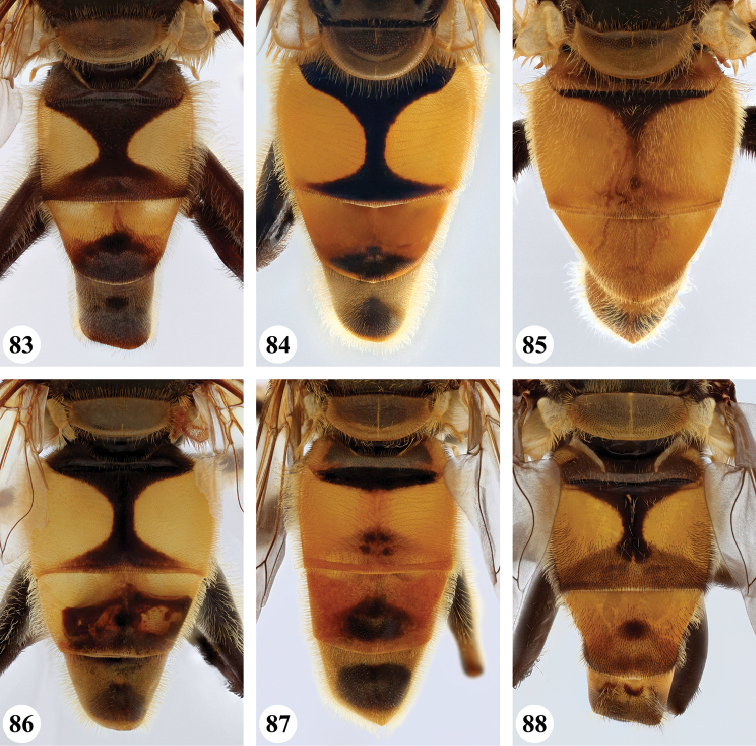
*Mesembrius* spp., abdomen, dorsal view **83***M. arcuatus* sp. nov. (♂) **84***M. caffer* (Loew) (nominal morph) (♂) **85***M. caffer* (Loew) (spined morph) (♂) **86***M. ctenifer* Hull syn. nov. (♂) **87***M. capensis* (Macquart) (♂) **88***M. chapini* Curran (♂).

***Genitalia*** (Fig. 220). Epandrium: Dorsal lobe of surstylus short, broadly rounded; with short, black spines on almost entire surface; long yellow pilose dorsally, especially at proximal end. Ventral lobe of surstylus straight; bare.

**Figures 89–94.**
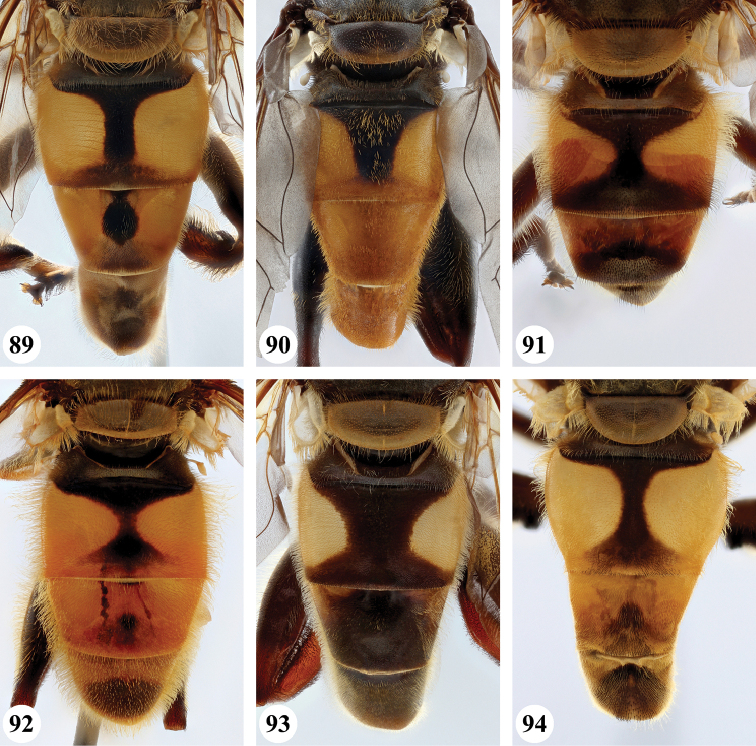
*Mesembrius* spp., abdomen, dorsal view **89***M. copelandi* sp. nov. (♂) **90***M. cyanipennis* (Bezzi) (♂) **91***M. ingratus* (Loew) (♂) **92***M. longipilosus* sp. nov. (♂) **93***M. madagascariensis* Keiser (♂) **94***M. minor* (Bezzi) (♂).

##### Description female

**(Fig. 38).** Body length: 11.8–16.7 mm. Wing length: 11.2–12.5 mm.

***Head*** (Fig. 76). Eyes bare; dichoptic. Face white with dark medial vitta; white pilose; white pollinose. Distance between lateral ocellus and eye margin approx. width of ocellus. Occiput black; yellow and black pilose; yellow pollinose. Frons black; black pilose; yellow pollinose on ventral half, sometimes pollinosity almost absent. Frontal prominence shiny black, orange-brown at distal end; scape and pedicel orange-brown to black; postpedicel black; postpedicel white pollinose; antennal arista reddish-brown.

**Figures 95–100.**
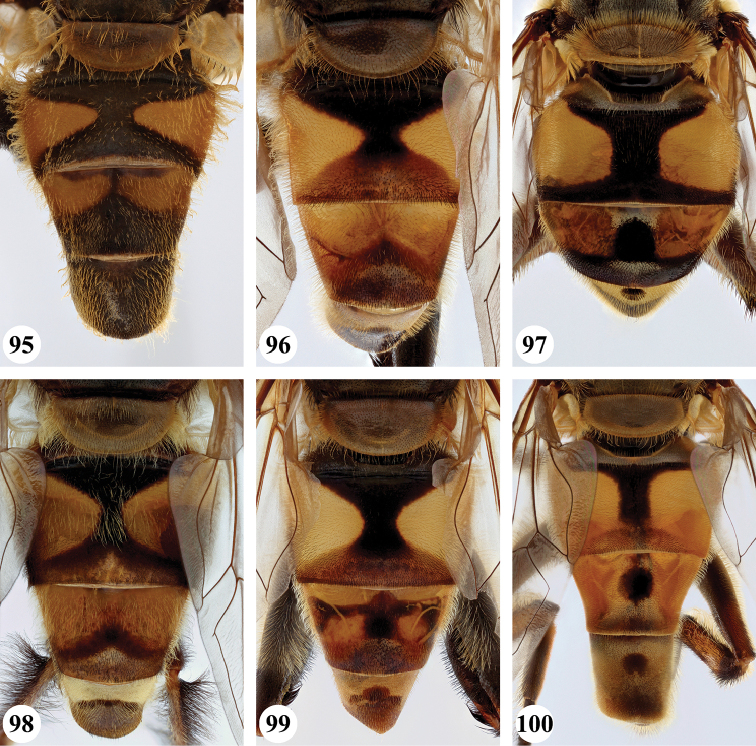
*Mesembrius* spp., abdomen, dorsal view **95***M. nigriceps* Curran (♂) **96***M. perforatus* (Speiser) (♂) **97***M. platytarsis* Curran syn. nov. (♂) **98***M. regulus* (Hull) (♂) **99***M. rex* Curran (♂) **100***M. senegalensis* (Macquart) (♂).

***Thorax.*** Scutum dark brown to black with, dorsally, a pair of very vague yellow pollinose vittae; short yellow and black pilose.

***Legs.*** All legs brown to black, protibia and protarsus lighter, yellow-brown; protarsus lighter than distal part of protibia; profemur predominantly black pilose, the pile is longer on the posterior and posterodorsal side than on the remainder of the profemur; pro- and mesotibia black pilose in distal 1/2–1/4, otherwise yellow and black pilose.

**Figures 101–106.**
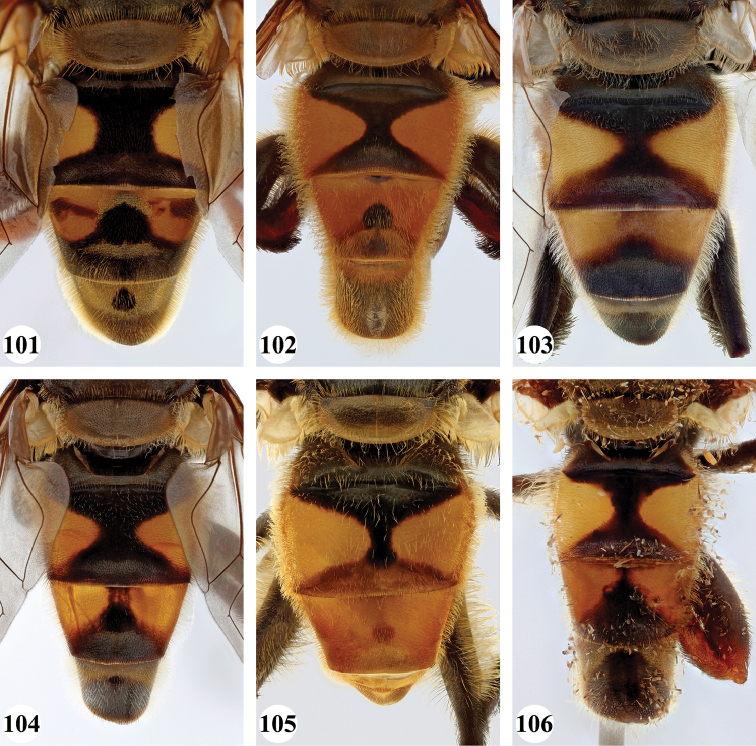
*Mesembrius* spp., abdomen, dorsal view **101***M. simplicipes* Curran (♂) **102***M. strigilatus* (Bezzi) (♂) **103***M. sulcus* sp. nov. (♂) **104***M. tarsatus* (Bigot) (♂) **105***M. tibialis* sp. nov. (♂) **106***M. vockerothi* sp. nov. (♂).

***Wing.*** Entire wing uniformly dense microtrichose. Wing cell r_1_ nearly closed.

***Abdomen*** (Fig. 119). Tergite II with a pair of large, orange maculae; black medial marking narrow, approximately 1/9 of tergal width; orange pilose on anterior half, black pilose on posterior half; posterior black marking strongly white pollinose. Tergite III with orange fascia (approx. half of tergite length on lateral sides; approx. 1/5 of tergite length in medial area); orange pilose on anterior end, otherwise black pilose; posterior half white pollinose, especially in medial area. Tergite IV as tergite III, but yellow pilose throughout with black pile interspersed on black marking. Tergite V black with or without a pair of vague orange maculae in anterolateral corner; yellow pilose; white pollinose on anterior half.

**Figures 107–112.**
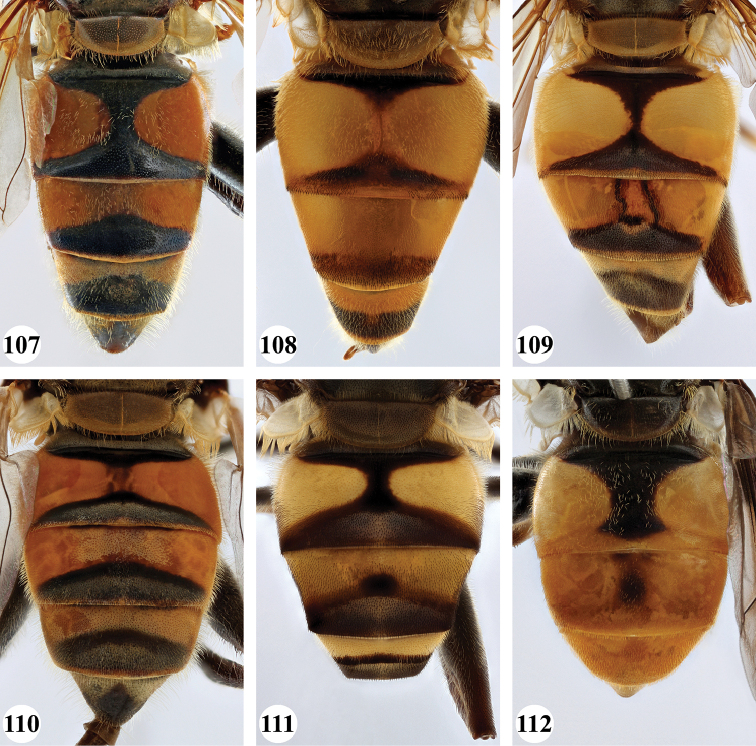
*Mesembrius* spp., abdomen, dorsal view **107***M. caffer* (Loew) (nominal morph) (♀) **108***M. caffer* (Loew) (spined morph) (♀) **109***M. ctenifer* syn. nov. Hull (♀) **110***M. capensis* (Macquart) (♀) **111***M. chapini* Curran (♀) **112***M. cyanipennis* (Bezzi) (♀).

##### Distribution.

Benin, Cameroon, Democratic Republic of the Congo, Nigeria and Togo.

##### Comments.

The male has a set of unambiguous character states mentioned in the original description and cannot be confused with any other species of the genus. The specimens we have studied correspond with the original species description and are, therefore, considered to be conspecific. Until now, the species was only known from the male holotype. We here report on the first females, which we matched with the males through DNA barcoding. The species seems locally common in west and central Africa.

**Figures 113–118.**
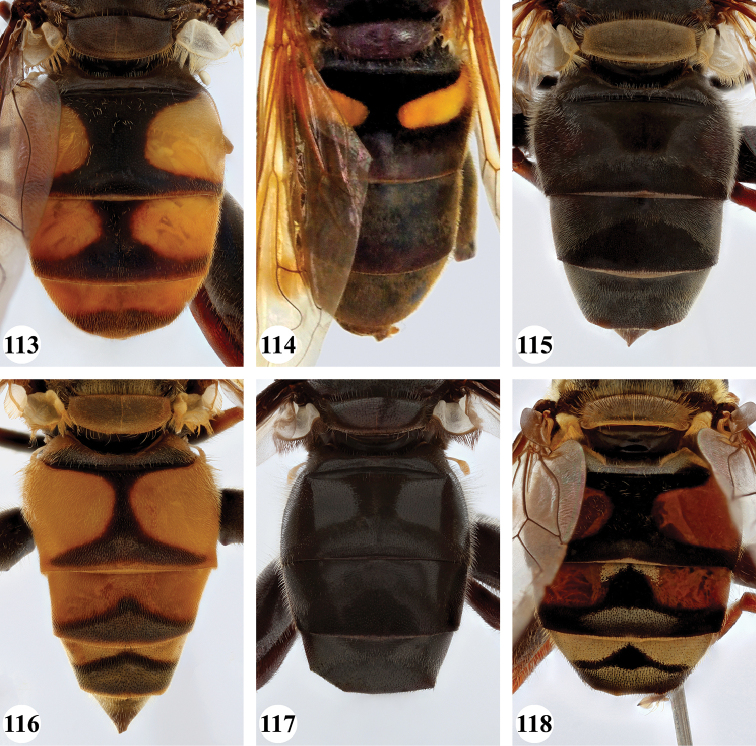
*Mesembrius* spp., abdomen, dorsal view **113***M. cyanipennis* (Bezzi) (♀) **114***M. maculifer* Hull (♀) **115***M. madagascariensis* Keiser (♀) **116***M. minor* (Bezzi) (♀) **117***M. morio* (Bezzi) (♀) **118***M. platytarsis* Curran syn. nov. (♀).

**Figures 119–123.**
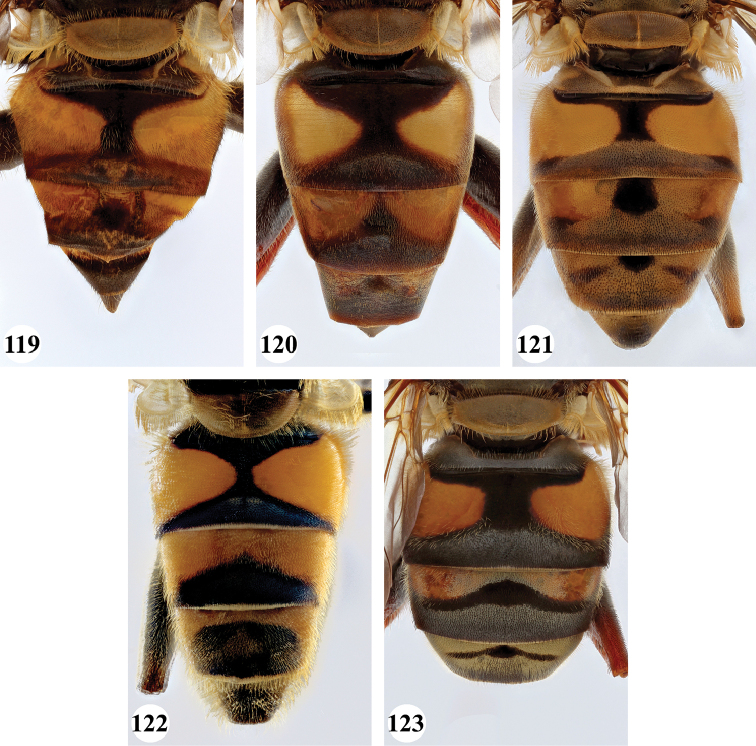
*Mesembrius* spp., abdomen, dorsal view **119***M. regulus* (Hull) (♀) **120***M. rex* Curran (♀) **121***M. senegalensis* (Macquart) (♀) **122***M. simplicipes* Curran (♀) **123***M. strigilatus* (Bezzi) (♀).

**Figures 124–126.**
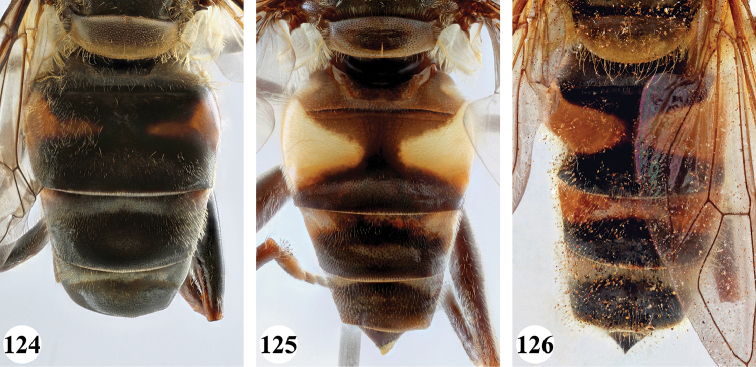
*Mesembrius* spp., abdomen, dorsal view **124***M. sulcus* sp. nov. (♀) **125***M. tarsatus* (Bigot) (♀) **126***M. vockerothi* sp. nov. (♀).

#### Mesembrius rex

Taxon classificationAnimaliaDipteraSyrphidae

Curran, 1927

251C351C-606C-5B46-884D-CE570E23B925

Figs 19 , 39 , 62 , 77 , 99 , 120 , 143 , 154 , 170 , 184 , 221

Mesembrius rex Curran, 1927: 61.
Mesembrius rex – Curran (1939): 10 – Smith and Vockeroth (1980): 504.

##### Differential diagnosis.

*Mesembrius rex* males have an entirely black apical pile brush on the profemur, a metatibia with a row of > 10 short, widely spaced black spines (without spines or with dense pile in other species). The metatibia has one deep depression on the posterior side (three in *M. perforatus*; none in *M. tibialis*) which is not markedly bordered with long black pile (bordered with long black pile in *M. chapini* and *M. sulcus* sp. nov.). Females have a frons which is black pilose on its entire length, except laterally. The female can be distinguished from the female of *M. sulcus* sp. nov. and *M. tarsatus* by the colour of the tibiae (yellow-brown to chocolate-brown in *M. rex*; black in *M. sulcus* sp. nov. and *M. tarsatus*), the absence of black pile on the ventral side of the pro- and mesotibia (present in *M. regulus* and *M. chapini*). It also differs from *M. regulus* by the concolourous protarsus and protibia (protarsus lighter than distal part of protibia in *M. regulus*) and wing cell r_1_ which is distinctly open (nearly closed in *M. regulus*).

##### Examined material.

*Mesembrius rex* Curran: Holotype, male, “Mesembrius // TYPE // rex Curran // No.” “Taken from Bembex” “Stanleyville, Cgo. // 25°10’E, 0°30’N // IV.7.1915” “Lang & Chapin // collectors” “Stanleyville // Congo // From Leg of // Type [♂]” “Mesembrius // rex // det. Curran // Det. C.H. Curran” [AMNH] [type studied from picture on website].

##### Other material

 Democratic Republic of the Congo • 1♀; Bolongo; 23 Jun 1936; J. Ghesquière leg.; KBIN • 1♀; Lulua, Kapanga; Nov 1928; Walker leg.; KMMA • 1♀; Basoko; Oct 1948; P.L.G. Benoit leg.; RMNH • 1♂; Eala; Oct 1935; J. Ghesquière leg.; RMNH • 1♀; locality and date unknown; J. Ghesquière leg.; KBIN. Malawi • 1♀; Mount Mulanje, Likhubula; 19 Nov 1912; S.A. Neave leg.; NHMUK • 1♂; Mount Mulanje; 25 Nov 1912; S.A. Neave leg.; NHMUK • 1♀; Mount Mulanje; 2 Dec 1912; S.A. Neave leg.; NHMUK • 1♀; Mount Mulanje; 16 Nov 1912; S.A. Neave leg.; NHMUK • 1♀; Mount Mulanje; 25 Nov 1912; S.A. Neave leg.; NHMUK. Togo • 1♀; Kloto Forest; Feb 2016; G. Goergen leg.; IITA. Uganda • 1♂; W. shores of Vic. Nyanza, Buddu; 19–25 Sep 1911; S.A. Neave leg.; NHMUK • 1♂; Barada; 16 Apr 1940; B. Lebied leg.; NHMUK • 1♂; Entebbe; 11 Nov 1912; C.C. Gowdey leg.; NHMUK • 2♂♂; Entebbe; 7 Oct 1971; H. Falke leg.; CNC • Country Unknown • 1♀; locality unknown; 1973; F.M. Hull leg.; CNC.

##### Re-description male

**(Fig. 19).** Body length: 14.3–17.0 mm. Wing length: 10.0–11.3 mm.

***Head*** (Fig. 62). Eyes bare; holoptic, eye contiguity approximately as long as the length of the ocellar triangle. Face yellow with dark medial vitta; white pilose; white pollinose. Frontal triangle short, black, with a few long, black pile; white pollinose on dorsal half; vertical triangle black, black pilose, yellow pollinose on lower half. Distance between lateral ocellus and eye margin less than 1/2 width of ocellus. Frontal prominence shiny black with orange-brown apex. Occiput black; yellow pilose, with black pile interspersed dorsally; yellow and white pollinose. Antenna, scape and pedicel black; postpedicel brown; antennal arista orange-brown.

***Thorax.*** Scutum black with, dorsally, one pair of grey pollinose vittae; lateral vitta faint, not well-demarcated; pile, especially on the anterior half yellow, but with some black pile interspersed; pile on posterior half very short. Scutellum black in anterior 1/3, brown in middle 1/3, white-yellow in posterior 1/3, with long yellow and shorter black pile. Metasternum with very long, strongly curved golden pile.

***Legs.*** Proleg (Fig. 154): Femur black; dorsoventrally flattened; with an apical pile brush of long, black pile dorsally and long, yellow pile ventrally. Tibia orange-brown; with very long, yellow pile on anteroventral side. Basitarsus orange; with a tuft of orange pile on posterior side. Other tarsi very broad; orange; becoming shorter distally; most distal tarsal segment white. Mesoleg: Femur dark brown; with long yellow pile dorsally, except for black pile on dorsal 1/5. Tibia orange-brown; proximal half strongly compressed. Basitarsus orange-brown. Other tarsi dark brown. Metaleg (Fig. 184): Coxa with long yellow pile on anteroventrally. Femur long and slender; chocolate-brown; ventrally with a row of > 10 short, widely spaced black spines in the proximal 2/3; with denser, black spines on posterior 1/3; pile otherwise yellow and loose. Tibia orange-brown; with a deep invagination in the proximal 1/3 posteriorly which is bordered with long, black pile ventrally; remainder of ventral side with dense, black pile. Tarsi orange-brown.

***Wing*** (Fig. 143). Entire wing uniformly dense microtrichose; brown infuscated in dorsal half.

***Abdomen*** (Fig. 99). Tergite II with a pair of large, yellow rounded maculae; black markings hourglass-shaped, white pollinose on posterior end; yellow and black pilose, but black pile more conspicuous in posterior part of black marking. Tergite III with broad yellow fascia and a triangular black marking on posterior half which is strongly white pollinose and covered with short, black spines; yellow pilose otherwise. Tergite IV with large triangular posterior black marking; otherwise yellow with strong white pollinosity.

***Genitalia*** (Fig. 221). Epandrium: Dorsal lobe of surstylus short, broadly rounded, with short, black spines on almost entire surface; long brown pilose dorsally. Ventral lobe of surstylus straight; bare.

##### Description female

**(Fig. 39).** Body length: 16.0–16.2 mm. Wing length: 11.2–12.5 mm. As *M. regulus*, but with the following differences: Pro- and mesotibia yellow pilose, with only short black pile on posterior side of mesotibia; protarsus chocolate-brown, concolourous with protibia (Fig. 170); wing cell r_1_ distinctly open. Abdomen (Fig. 120) with orange pilosity somewhat more prominent. Head as in Fig. 77.

##### Distribution.

Democratic Republic of the Congo, Malawi, Togo and Uganda.

##### Comments.

The male has a set of unambiguous character states mentioned in the original description and cannot be confused with any other species of the genus. The specimens we have studied correspond with the photographs of the type and are, therefore, considered to be conspecific. Until now, the species was only known from the male holotype. We here report on the first females, which we matched with the males through DNA barcoding. The species seems rare throughout a large part of the Afrotropical Region and seems absent from southern Africa.

#### Mesembrius senegalensis

Taxon classificationAnimaliaDipteraSyrphidae

(Macquart, 1842)

DF44BA57-885C-50E8-951B-8C915B0E3D6B

Figs 20 , 40 , 63 , 78 , 100 , 121 , 144 , 161 , 174 , 181 , 200 , 222

Helophilus senegalensis Macquart, 1842: 121.
Tubifera senegalensis – Kertész (1910): 260.
Mesembrius senegalensis – Curran (1927): 65 – Curran (1939): 10 – Smith and Vockeroth (1980): 504.
Helophilus africanus Verrall, 1898: 416. syn. nov.
Tubifera africana – Kertész (1910): 249.
Helophilus (Mesembrius) africanus – Bezzi (1915): 97.
Mesembrius africanus – Smith and Vockeroth (1980): 504.

##### Differential diagnosis.

*Mesembrius senegalensis* males lack an apical pile brush on the profemur and have an unmodified metatibia. The proximal ventral part of the profemur lacks black pile and the metafemur is covered with long, thin yellow pile and has a band of very short, thicker, black pile on the posteroventral side and some scattered short black pile ventrally. The yellow-orange maculae on tergite II are very large and rectangular and the anterior and posterior black markings are narrow and perpendicular to the narrow medial black vitta. The male is distinguished from morphologically similar species in the shape of the maculae on tergite II which are rectangular (rounded to triangular in other species) and the band of short thick black pile on the posteroventral side of the metafemur (fewer in *M. longipilosus* sp. nov.; much denser and longer in *M. copelandi* sp. nov., *M. minor* and *M. strigilatus*). Apart from the shape of the maculae, it also differs from *M. longipilosus* sp. nov. with the absence of equally long black pile amongst the long yellow pile on the proximal ventral end of the metafemur (several in *M. longipilosus* sp. nov.). Females have a frons which is pale pilose on the ventral half. Tergite II has a pair of yellow maculae (fascia in *M. capensis* and spined morph of *M. caffer*). The black markings on the abdomen are strongly reduced because of the strong white pollinosity (clearly visible in all other species). The pro- and metafemur, as well as the pro- and metatibia are yellow-brown (largely dark brown to black in other species) and the metafemur has no ventral swelling in the middle (swelling present in *M. minor*).

##### Examined material.

*Helophilus senegalensis* Macquart: Lectotype (hereby designated), male, “SYNTYPE” “MNHN, Paris // ED6788” “1 ♂ Helophilus // senegalensis Macq // C.F. Kassebeer 1999” [MNHN]. Paralectotype, female, “SYNTYPE” “MNHN, Paris // ED6789” [MNHN] [the female is indicated as male on MNHN website] [a paralectotype is present at the MNHN, but could only be studied from the pictures on the website; see comments].

*Helophilus africanus* Verrall: Lectotype (hereby designated), male, “S.W. ARABIA // 19 m. fr. Aden, // Haithalhim. // Capt. Mar. 23.95 // & press. 1899 by // J.W. Yerbury. // Trans. Ent. Soc., // 1898, page 413.” “S.W. ARABIA // 19 m. fr. Aden, // Haithalhim. // Capt. Mar. 23.95 // & press. 1899 by // J.W. Yerbury. // Trans. Ent. Soc., // 1898, page 416.” “TYPE. // G.H. VERRALL // Trans. Ent. Soc., // 1898, page 413.” “TYPE. // G.H. VERRALL // Trans. Ent. Soc., // 1898, page 416.” “TYPE Dipt: 105 1/4 // Helophilus // africanus // Verrall // HOPE DEPT. OXFORD” “1899 // 7645” “RMCA PIC // 00012” “LECTOTYPUS” [OXUM]. Paralectotype, male, “S.W. ARABIA // 19 m. fr. Aden, // Haithalhim. // Capt. Mar. 23.95 // & press. 1899 by // J.W. Yerbury. // Trans. Ent. Soc., // 1898, page 413.” “S.W. ARABIA // 19 m. fr. Aden, // Haithalhim. // Capt. Mar. 23.95 // & press. 1899 by // J.W. Yerbury. // Trans. Ent. Soc., // 1898, page 416.” “TYPE. // G.H. VERRALL // Trans. Ent. Soc., // 1898, page 413.” “TYPE. // G.H. VERRALL // Trans. Ent. Soc., // 1898, page 416.” “TYPE Dipt: 105 3/4 // Helophilus // africanus // Verrall // HOPE DEPT. OXFORD” “1899 // 7646” “RMCA PIC // 00013” “PARA- // LECTOTYPUS” [OXUM]. Paralectotype, female, “S.W. ARABIA // 19 m. fr. Aden, // Haithalhim. // Capt. Mar. 24.95 // & press. 1899 by // J.W. Yerbury. // Trans. Ent. Soc., // 1898, page 413.” “S.W. ARABIA // 19 m. fr. Aden, // Haithalhim. // Capt. Mar. 24.95 // & press. 1899 by // J.W. Yerbury. // Trans. Ent. Soc., // 1898, page 416.” “TYPE. // G.H. VERRALL // Trans. Ent. Soc., // 1898, page 413.” “TYPE. // G.H. VERRALL // Trans. Ent. Soc., // 1898, page 416.” “TYPE Dipt: 105 4/4 // Helophilus // africanus // Verrall // HOPE DEPT. OXFORD” “1899 // 7650” “RMCA PIC // 00015” “PARA- // LECTOTYPUS” [OXUM]. Paralectotype, female, “S.W. ARABIA // 19 m. fr. Aden, // Haithalhim. // Capt. Mar. 23.95 // & press. 1899 by // J.W. Yerbury. // Trans. Ent. Soc., // 1898, page 413.” “S.W. ARABIA // 19 m. fr. Aden, // Haithalhim. // Capt. Mar. 23.95 // & press. 1899 by // J.W. Yerbury. // Trans. Ent. Soc., // 1898, page 416.” “TYPE. // G.H. VERRALL // Trans. Ent. Soc., // 1898, page 413.” “TYPE. // G.H. VERRALL // Trans. Ent. Soc., // 1898, page 416.” “TYPE Dipt: 105 5/4 // Helophilus // africanus // Verrall // HOPE DEPT. OXFORD” “1899 // 7648” “RMCA PIC // 00016” “PARA- // LECTOTYPUS” [OXUM]. Paralectotype, female, “Haithalhim // 23.3.95 // Col. Yerb.” “VC-TYPE 33 // Helophilus ♀ // africanus // Verrall” “Haithalhim” “PARA- // LECTOTYPUS” [OXUM].

##### Other material

 Benin • 1♂; Cotonou; Feb 2003; G. Goergen leg.; IITA • 2♀♀; Cotonou; 14 Jan 2013; K. Jordaens and G. Goergen leg.; KMMA • 1♂ 1♀; Cotonou; 28 Jan 2016; K. Jordaens and G. Goergen leg.; KMMA • 1♂; Togbin; Dec 2005; G. Goergen leg.; IITA. Chad • 1♀; Bebedja; date unknown; F.A. Brink and R.M. Brink-Moenen leg.; RMNH. Kenya • 2♂♂ 7♀♀; Jipe, Taita-Taveta; 27 Jan 2017; M. Reemer leg.; RMNH • 2♂♂ 2♀♀; Jipe; Taita-Taveta; 27 Jan 2017; X. Mengual leg.; ZFMK • 1♀; Nairobi, ICIPE campus; 6 May 2014; R. Copeland leg.; ICIPE • 1♂; Taita Hills; 2017; A. Ssymank leg.; ASPC • 1♀; Makindu; 5–7 Apr 1911; S.A. Neave leg.; NHMUK. Oman • 1♂; Dhofar, Ayun pools; 8 Oct 1977; K.M. Guichard leg.; NHMUK.

##### Re-description male

**(Fig. 20).** Body length: 12.0–13.8 mm. Wing length: 9.2–10.2 mm.

***Head*** (Fig. 63). Eyes bare; slightly dichoptic, distance between eyes approx. 1/2 width of ocellus. Face yellow with dark medial vitta; yellow pilose; yellow pollinose. Vertical triangle with yellow pile and yellow pollinosity in lower half, black in upper half; distance between lateral ocellus and eye margin less than 1/2 width of ocellus. Occiput yellow; yellow pilose with interspersed short, black setulae; yellow and white pollinose. Frontal triangle yellow; yellow pilose; yellow pollinose. Frontal prominence shiny black; yellow pilose. Antenna black; antennal arista reddish-brown.

***Thorax.*** Scutum black with three dorsal, well-demarcated yellow vittae which are connected anteriorly and posteriorly; with lateral, yellow vitta; pile rufous. Scutellum yellow-brown; yellow pilose.

***Legs.*** All legs light- to dark brown. Proleg (Fig. 161) and mesoleg (Fig. 181): profemur without apical pile brush; yellow pilose, pile ventrally long in proximal half, shorter in distal half; with shorter black pile on distal half. Tibia yellow and black pilose. Tarsi black pilose dorsally, yellow pilose ventrally; with some thick black pile posterodorsally. Metaleg: Femur anteriorly and dorsally with long and posteriorly with shorter, yellow pile; ventral yellow pile scarce, except for a row of long, thin pale pile; with band of short black pile posteroventrally. Tibia unmodified; long yellow pilose; ventrally with much shorter and thicker black pile. Tarsi black pilose dorsally, yellow pilose ventrally.

***Wing*** (Fig. 144). Entire wing uniformly dense microtrichose.

***Abdomen*** (Fig. 100). Tergite II with a pair of very large, yellow to orange rectangular maculae; medial black markings very narrow and perpendicular to anterior and posterior narrow, black marking; posterior black marking with short, stiff black setulae which do not extend to the lateral tergite sides; white pollinose. Tergite III and IV with very large, yellow fascia; yellow pilose; with large medial black marking; with short black stiff setulae posterior to medial dark spot, these setulae not reaching the lateral tergite sides. Tergite III white pollinose in medial part. Tergite IV entirely white pollinose.

***Genitalia*** (Fig. 222). Epandrium: Dorsal lobe of surstylus distally broadly rounded, with characteristic upwardly pointed projection; long pilose dorsally; with shorter, dense black pile ventrally and laterally. Ventral lobe of surstylus with a row of approx. 10 long black setulae.

##### Re-description female

**(Fig. 40).** Body length: 11.5–14.4 mm. Wing length: 8.3–10.4 mm. As male, except for the following character states: Eyes dichoptic (Fig. 78). Frons yellow pilose in ventral 2/3, black and yellow pilose on dorsal 1/3 (ocellar triangle and surrounding area); strongly yellow pollinose. Pile on legs shorter (Figs 174, 200). Abdomen as in Fig. 121.

##### Distribution.

Benin, Chad, Kenya, Oman and Yemen.

##### Comments.

Verrall (1898) already suggests that *M. africanus* could be conspecific to *M. senegalensis* (Macquart, 1842), but he did not study the type of the latter. We have studied the syntypes of both species and confirm Verrall’s suggestion that both species are conspecific and, therefore, we consider *M. africanus* (Verrall, 1898) a junior synonym of *M. senegalensis* (Macquart, 1842). A paralectotype of *M. senegalensis* (at the MNHN) is on loan and several requests to the borrower to return the specimen were left unanswered.

#### Mesembrius simplicipes

Taxon classificationAnimaliaDipteraSyrphidae

Curran, 1929

2B80D8D0-9198-5DA3-82C8-F97213F40741

Figs 17 , 21 , 37 , 41 , 60 , 64 , 75 , 79 , 97 , 101 , 118 , 122 , 141 , 145 , 166–167 , 193 , 194 , 219 , 223

Mesembrius simplicipes Curran, 1929: 500.
Mesembrius simplicipes – Keiser (1971): 266 – Smith and Vockeroth (1980): 504.
Mesembrius platytarsis Curran, 1929: 501. syn. nov.
Mesembrius platytarsis – Hull (1941): 330 – Keiser (1971): 265 – Smith and Vockeroth (1980): 504.

##### Differential diagnosis.

*Mesembrius simplicipes* males lack an apical pile brush on the profemur which is dorsoventrally flattened. The pro- and mesolegs are orange and with a darker area on the dorsal side of the femur. The probasitarsus is laterally expanded and has some long orange pile. The large yellow maculae on the abdomen lack short, black spines on the posterior edges. Scutum and scutellum are entirely yellow pilose. The male of this species cannot be confused with any other species by the lateral lobe on the probasitarsus. Females have a nearly black abdomen with a pair of vague lateral maculae on tergites II and III. The female of *M. simplicipes* can be distinguished from any other species (except from *M. madagascariensis*) by the nearly black abdomen (clearly yellow to orange and black in other species). It can be distinguished from *M. madagascariensis* by the pro- and mesolegs which are reddish-brown (extensively brown and black in *M. madagascariensis*).

##### Examined material.

*Mesembrius simplicipes* Curran: Holotype, male: “Mesembrius // TYPE // simplicipes // Curran. // No.” “Mesembrius // simplicipes // Curran” “Madagascar // Great Oriental // Forest” “California Academy // of Sciences // Type No. 11230” [CAS] [date and collector unknown; type studied from pictures].

*Mesembrius platytarsis* Curran: Holotype, male: “Mesembrius // TYPE // platytarsis // Curran. // No.” “Madagascar // Great Oriental // Forest” “California Academy // of Sciences // Type No. 11229” [CAS] [date and collector unknown; type studied from pictures].

##### Other material

 Madagascar • 2♀♀; Alaotra, Station Agric.; 24 Dec 1957; B.R. Stuckenberg leg.; NMSA • 1♀; Analvony; 30 Mar 1958; F. Keiser leg.; NMB • 1♂; Antananarivo; Nov 1952; E.S. Brown leg.; NHMUK • 1♂; Antananarivo; 18 Oct 1957; F. Keiser leg.; NMB • 1♀; Antananarivo; 13 Dec 1957; F. Keiser leg.; NMB • 2♀♀; Antananarivo; 14 Dec 1957; F. Keiser leg.; NMB • 1♂; Antananarivo; 6 Sep 1958; F. Keiser leg.; NMB •1♀; Antananarivo; 8 Feb 1970; L. and R. Blommers leg.; RMNH • 1♂; Antananarivo, Ampefy, Lake Kavitaha; 25 Mar 1959; F. Keiser leg.; NMB • 1♂; Antananarivo, Ampefy, Lake Kavitaha; 29 Mar 1959; F. Keiser leg.; NMB • 1♂; Antananarivo, Parc Tsimbazaza; 2 Feb 1968; J.W. Boyes leg.; CNC • 1♀; Antananarivo, Park Tsimbazaza; 14 Dec 1957; F. Keiser; NMB • 1♀; Antananarivo, Park Tsimbazaza; 15–22 Oct 1993; C. Kassebeer leg.; NHMUK • 1♂ 1♀; Antananarivo, Park Tsimbazaza; 16–22 Oct 1993; C. Kassebeer leg.; CNC • 2♂♂; Antananarivo, Park Tsimbazaza; 16–22 Oct 1993; C. Kassebeer leg.; CAS • 1♂ 1♀; Antananarivo, Park Tsimbazaza; 16–22 Oct 1993; C. Kassebeer leg.; NMK • 1♀; Antananarivo, Park Tsimbazaza; 26 Oct 1993; C. Kassebeer leg.; CNC • 1♀; Antananarivo, Park Tsimbazaza; 26 Oct 1993; C. Kassebeer leg.; CAS • 1♂; Antananarivo, Park Tsimbazaza; 6 Nov 1993; C. Kassebeer leg.; CNC • 1♂; Antananarivo, Park Tsimbazaza; 6 Nov 1993; C. Kassebeer leg.; NHMUK • 1♂; Antananarivo, Perinet; 30 Sep 1957; F. Keiser leg.; NMB • 1♀; Nosivola; date and collector unknown; RMNH.

##### Re-description male

**(Figs 17, 21).** Body length: 12.7–13.4 mm. Wing length: 8.8–10.4 mm.

***Head*** (Figs 60, 64). Eyes bare; holoptic, eye contiguity as long as length of ocellar triangle. Face white with dark medial vitta; white pilose; white pollinose. Vertical triangle black; black pilose; strongly yellow pollinose before anterior ocellus. Lateral ocelli nearly touching eye margin. Occiput black, yellow pilose with a few very short, stiff black setulae near dorsal eye margin; strongly white pollinose. Frontal triangle black; black pilose; strongly yellow pollinose. Frontal prominence shiny reddish-black. Antenna, scape and pedicel very dark reddish-black; postpedicel black; antennal arista reddish-brown.

***Thorax.*** Scutum black with dorsally a pair of yellow pollinose vittae which are less well demarcated posteriorly; with a lateral yellow pollinose vitta; yellow pilose. Scutellum uniformly yellow-brown; yellow pilose.

***Legs.*** Proleg (Figs 166, 167): Femur dorsoventrally flattened; dorsally brown, except proximal 1/5 and distal end; otherwise orange; yellow pile on posteroventral side longer than black pile on posterodorsal side; with a patch of very short black stiff spines on proximal ventral 1/5; pile entirely yellow anteriorly. Tibia orange; with long orange pile, but pile black on ventral distal end. Basitarsus whitish; laterally expanded; with a tuft of orange pile on the expansion; ventrally with some thick stiff pile on the expansion. Other tarsi orange; with orange and black short pile. Mesoleg: Femur as proleg, but without the posterodorsal black pile. Tibia orange; yellow pilose, with some short stiff black pile on distal end ventrally. Tarsi orange; with short black pile and some short stiff black pile ventrally. Metaleg (Figs 193, 194): Femur chocolate-brown, distal end orange; with loose yellow pile on proximal 1/2 to 2/3 and mostly black pilose on distal 1/3 to 1/2. Tibia orange; with short black pile. Metabasitarsus either deeply excavated anteriorly at proximal end and with a lobe (Fig. 193) or unmodified (Fig. 194); orange; black pilose. Other tarsi orange; dorsally black pilose, ventrally orange pilose.

***Wing*** (Figs 141, 145). Entire wing uniformly very dense microtrichose.

***Abdomen*** (Figs 97, 101). Tergite II with a pair of very large, yellow almost square maculae which expand into the anterolateral corners; black markings hourglass-shaped, posterior part reaching the lateral sides of the tergite; posterior part of black marking with some black pile in the centre which do not extend to the lateral tergite sides; otherwise yellow pilose. Tergite III with a yellow fascia which is almost interrupted by the medial broad black marking; the latter strongly white pollinose anteriorly; with a medial area of white pollinosity; posterior part of black marking with sparse black pile in the centre; yellow pilose otherwise. Tergite IV strongly white pollinose, except for a black medial area and a darker posterior border; yellow pilose.

***Genitalia*** (Figs 219, 223). Epandrium: Dorsal lobe of surstylus short, bent (as a boomerang); irregularly covered with short black spines which are denser at distal and proximal end and at dorsal bend. Ventral lobe of surstylus straight; bare.

##### Re-description female

**(Figs 37, 41).** Body length: 14.2–14.9 mm. Wing length: 10.0–10.6 mm.

***Head*** (Figs 75, 79). Eyes bare; dichoptic. Face yellow-white with dark medial vitta; white pilose, white pollinose. Frons black in dorsal half and medial part of ventral half, yellow-white on lateral parts of ventral half; black pilose on black parts, white pilose on yellow-white parts. Distance between lateral ocellus and eye margin approx. width of ocellus. Occiput yellow-white; yellow-white pilose; yellow-white pollinose. Frontal prominence shiny black, orange-brown at distal end; scape black; pedicel orange-brown; postpedicel black; antennal arista reddish-brown.

***Thorax.*** Scutum dark brown with one pair of dorsolateral yellow-white pollinose vittae which are vaguely connected posteriorly; with lateral, yellow-white pollinose vitta; short yellow pilose on anterior half, short yellow and black pilose on posterior half. Scutellum yellow-orange; yellow pilose with shorter, black pile interspersed in posterior half.

***Legs.*** Proleg: Femur orange brown with darker, central area on dorsal side; yellow-white pilose with black pile on posterodorsal side and posteroventral distal half. Tibia orange, slightly darkened on ventral distal end; yellow-white pilose. Tarsi orange-brown; basitarsus and second tarsomere yellow-white pilose, other tarsi yellow-white and black pilose. Femur orange brown with darker, central area on dorsal side; yellow-white pilose with black pile on ventral distal half. Tibia orange-brown; yellow-white pilose, with some short, thick black pile at ventral distal end. Basitarsus orange; yellow-white pilose, with short, thick black pile on ventral side. Other tarsi orange-brown; yellow-white and black pilose; with short, thick black pile ventrally, except on most distal tarsomere. Metaleg: Femur dark brown to black, reddish-brown at distal end; yellow-white pilose with shorter and thicker black pile on anteroventral and ventral distal 1/2. Tibia orange-brown; yellow-white and black pilose. Tarsi orange-brown; black and yellow-white pilose dorsally, yellow-orange pilose ventrally.

***Wing.*** Entire wing uniformly microtrichose.

***Abdomen*** (Figs 118, 122). Tergite II with a pair of large orange maculae; with an anterior and posterior black marking which are connected with a parallel-sided central black marking which is 1/5 the tergite width; yellow pilose on maculae and on anterior and central black marking, black pilose on posterior black marking; white pollinose on posterior black marking. Tergite III with orange fascia (approx. 1/2 of tergal length laterally; approx. 1/8 in medially); with posterior large black marking; yellow pilose on most of the orange fascia, black pilose on black marking and central area of orange fascia; strongly white pollinose on posterior half and medial anterior part. Tergite IV similar, but without orange fascia. Tergite V orange-brown with darker lateral sides; yellow-white pilose; white pollinose in anterolateral corners.

##### Distribution.

Madagascar.

##### Comments.

Morphologically, the species is similar to *M. platytarsis* syn. nov. Males of both species differ in the presence (*M. platytarsis* syn. nov.) or absence (*M. simplicipes*) of a large lobe on the anterior side of the metabasitarsus. Male genitalia are also very similar (compare Fig. 219 with Fig. 223). Females are morphologically similar as well and the supposed difference in the extent of black pile amongst the yellow pile on the frons is unreliable. The mean interspecific *p*-distance between both species is very low (0.02%) and of what is usually as observed within species. Both species have been described from the same locality, i.e. the Eastern Forest of Madagascar. Keiser (1971) also noted that both taxa often co-occur. We, therefore, consider the presence or absence of the lobe on the anterior side of the metabasitarsus in the male as a polymorphism and consider *M. platytarsis* Curran, 1929 a junior synonym of *M. simplicipes* Curran, 1929.

#### Mesembrius strigilatus

Taxon classificationAnimaliaDipteraSyrphidae

(Bezzi, 1912)

904BC299-CC2E-5B9E-A5B5-B35FABC01813

Figs 22 , 42 , 65 , 102 , 123 , 146 , 172 , 197 , 203 , 224

Tubifera (Mesembrius) strigilata Bezzi, 1912: 436.
Helophilus (Mesembrius) strigilatus – Bezzi (1915): 96 – Curran and Bryan (1926): 82.
Mesembrius strigilatus – Hervé-Bazin (1914a): 481 – Curran (1927): 62 – Curran (1939): 10 – Szilády (1942): 92 – Smith and Vockeroth (1980): 504.

##### Differential diagnosis.

*Mesembrius strigilatus* males lack an apical pile brush on the profemur and have a metatibia which is strongly curved. The metafemur is curved, has a patch of conspicuous black pile at the base and, perpendicular to this, a stretch of dense black pile on the ventroposterior side. Tergite II has a pair of very large and rounded maculae and a narrow black medial marking. The male is distinguished from any other species by the strongly curved metafemur and metatibia, except from *M. nigriceps* though the metafemur and metatibia are less curved in the latter. It differs further from *M. nigriceps* in the the colour of the face (white to yellow in *M. strigilatus*; black in *M. nigriceps*), in the size and shape of the maculae on tergite II which are large and rounded (small and nearly triangular in *M. nigriceps*) and by the narrow black medial marking on tergite II (broad in *M nigriceps*). Females have a frons which is pale pilose on the ventral half. Females of *M. strigilatus* have a pair of yellow maculae on tergite II (fascia in the spined morph of *M. caffer* and in *M. capensis*). Pro- and mesofemur are dark brown to black (yellow-brown in *M. senegalensis*), the metafemur lacks a ventral medial swelling (present in *M. minor*), the face is not markedly produced downwards (produced downwards in *M. vockerothi* sp. nov.) and the mesofemur has long black pile ventrally, especially on the distal half (very few and short black pile on distal end in the nominal morph of *M. caffer*).

**Figures 127–130.**
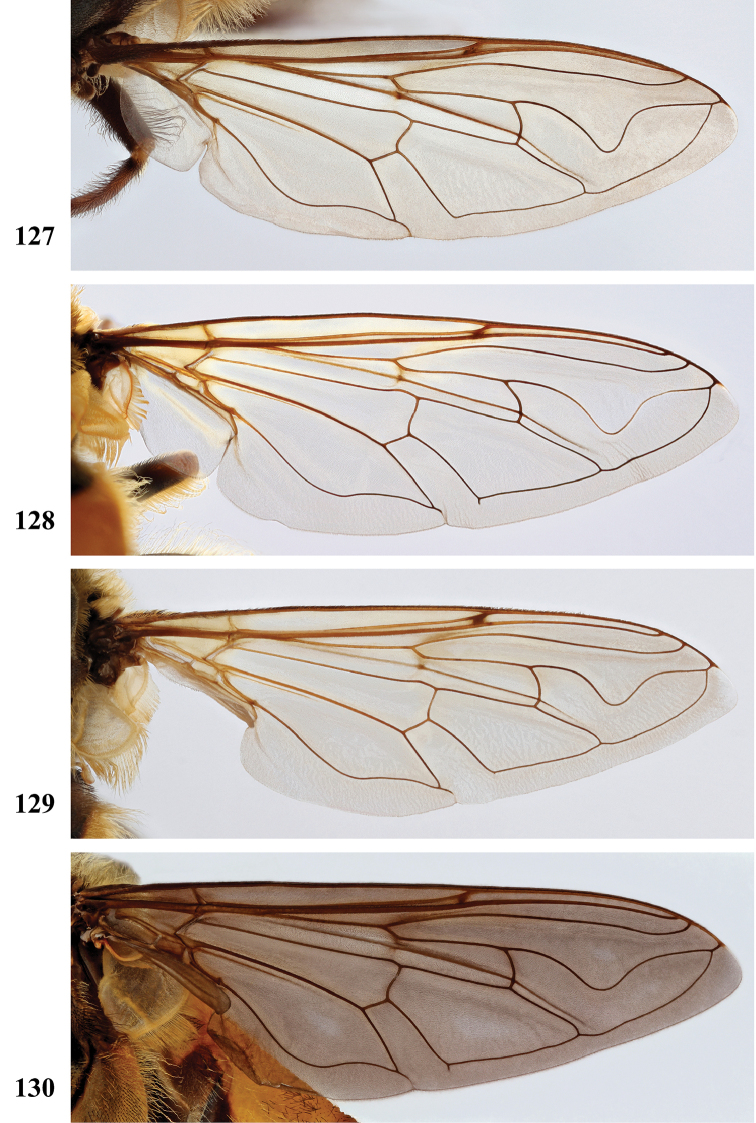
*Mesembrius* spp., right wing **127***M. arcuatus* sp. nov. (♂) **128***M. caffer* (Loew) (nominal morph) (♂) **129***M. capensis* (Macquart) (♂) **130***M. chapini* Curran (♂).

##### Examined material.

*Tubifera strigilata* Bezzi: Lectotype (hereby designated), male, “LECTOTYPUS” “SYNTYPUS ♂ // Tubifera (Mesemb.) // strigilata // Bezzi, 1912” “Congo Francese // Fernand-Vaz // IX-X.1902. L. fea” “RMCA PIC // 00033” “Museo Civico // di Genova” “LECTOTYPUS”[MSNG]. Paralectotype, 4 males, “SYNTYPUS ♂ // Tubifera (Mesemb.) // strigilata // Bezzi, 1912” “Congo Francese” // Fernand-Vaz // IX-X.1902. L. fea” “Museo Civico // di Genova” “PARA- // LECTOTYPUS” [MSNG]. Paralectotype, female, “SYNTYPUS ♀” “Tubifera (Mesemb.) // strigilata // Bezzi, 1912” “Museo Civico // di Genova” “PARA- // LECTOTYPUS” [MSNG].

**Figures 131–134.**
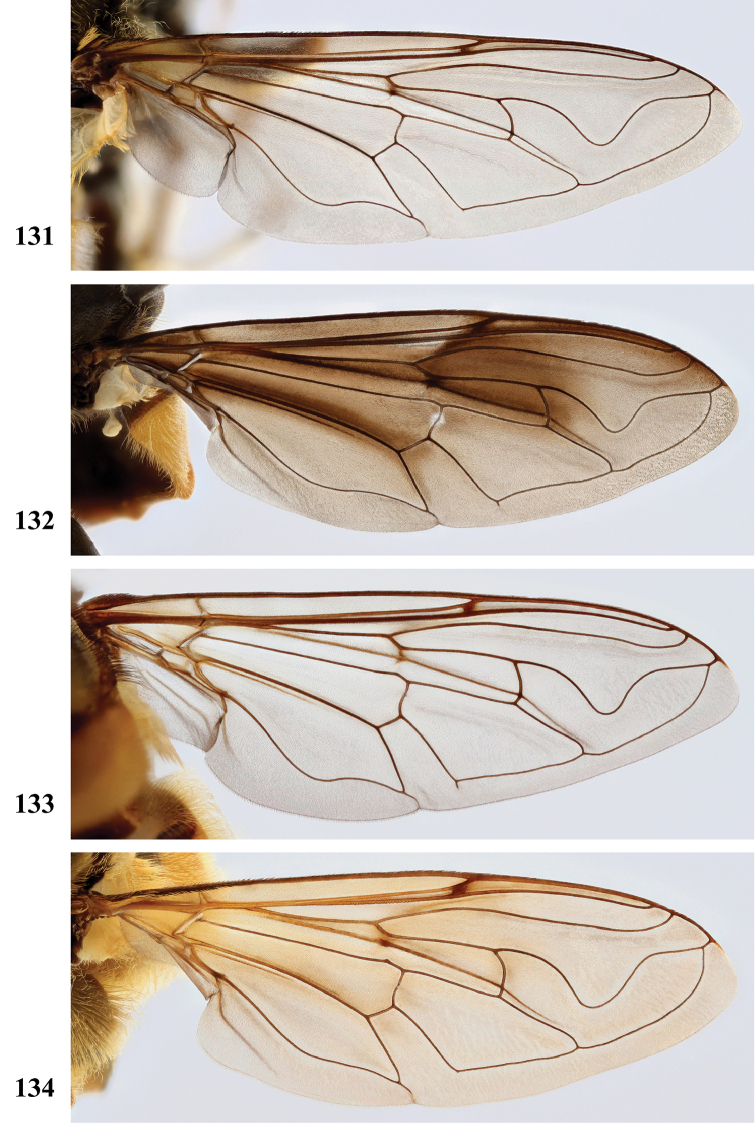
*Mesembrius* spp., right wing **131***M. copelandi* sp. nov. (♂) **132***M. cyanipennis* (Bezzi) (♂) **133***M. ingratus* (Loew) (♂) **134***M. longipilosus* sp. nov. (♂).

##### Other material

 Benin • 1♂ 1♀; Azaourissé; 7 Mar 2018; K. Jordaens leg.; KMMA • 1♂; Calavi; 27 Jan 2017; G. Goergen leg.; IITA • 1♀; Cotonou; 14 Dec 2013; G. Goergen and K. Jordaens leg.; KMMA • 1♀; Cotonou; Feb 2003; G. Goergen leg.; IITA • 1♀; Cotonou; Dec 2003; G. Goergen leg.; IITA • 1♂; Lokossa; Jun 2006; G. Goergen leg.; IITA • 1♂; Pahou; 11 Jan 2014; G. Goergen leg.; IITA • 1♂ 1♀; Pobé; 27 Jan 2016; G. Goergen leg.; IITA • 2♂♂; Porto Novo; 20 Jan 2018; G. Goergen leg.; IITA • 1♂ 1♀; Gblo Gblo; 11 Sep 2014; G. Goergen leg.; KMMA • 1♀; Pahou; 11 Jan 2014; G. Goergen leg.; KMMA • 1♂ 1♀; Porto Novo; 27 Jan 2016; K. Jordaens leg.; KMMA • 7♂♂; Porto Novo; 7 Mar 2018; K. Jordaens leg.; KMMA • 1♀; Sérou; Nov 2016; G. Goergen leg.; KMMA • 1♂ 3♀♀; Pobé; 12 Dec 2013; G. Goergen and K. Jordaens leg.; KMMA • 4♂♂ 4♀♀; Pobé; 28 Jan 2016; G. Goergen and K. Jordaens leg.; KMMA. Burundi • 2♂♂; Bujumbura; 21 Feb 2017; G. Goergen leg.; KMMA • 1♂; Nyanza-Lac; 14 Oct 2013; L. Ndayikeza leg.; OBPE • 1♀; Rusizi River; 10 Nov 2010; L. Ndayikeza leg.; OBPE. Cameroon • 1♂; Batanga; collection date unknown; A. I. Good leg.; CNC • 5♂♂ 2♀♀; Douala; 9 Jul 1974; J.A.W. Lucas leg.; RMNH. Democratic Republic of the Congo • 2♀♀; Haut-Katanga; 4 Sep 1930; G.F. de Witte leg.; KMMA • 1♀; Equateur, Bamania; 21 Jul 1924; J. Bequaert leg.; KMMA • 1♀; Equateur, Bamania; 24 Jul 1924; J. Bequaert leg.; KMMA • 1♂; Basoko; Oct 1948; P.L.G. Benoit leg.; KMMA • 1♂; Bokuma; Jul 1962; R.P. Lootens leg.; KMMA • 1♀; Mai-Ndombe, Bololo, Makamendulu; 1938; H. Schouteden leg.; KMMA • 2♂♂; Boma; Jul 1915; Lang and Chapin leg.; KMMA • 1♀; Boma; 16 Jun 1915; Lang and Chapin leg.; KMMA • 1♀; Boma; 17 Jun 1915; Lang and Chapin leg.; KMMA • 1♀; Boma; 18 Jun 1915; Lang and Chapin leg.; KMMA • 4♀♀; Boma; 4 Dec 1920; H. Schouteden leg.; KMMA • 1♀; Boma; 11 Jul 1920; H. Schouteden leg.; KMMA • 1♂ 1♀; Boma; 12 Jul 1920; H. Schouteden leg.; KMMA • 1♀; Haut-Lomami, Bukama; 8 Jun 1911; J. Bequaert leg.; KMMA • 1♀; South-Kivu, Bukavu; May 1949; H. Bomans leg.; KMMA • 1♂; Equateur, Eala; Jul 1931; H.J. Brédo leg.; KMMA • 1♀; Equateur, Eala; Nov 1931; H.J. Brédo leg.; KMMA • 1♀; Equateur, Eala; 7 Oct 1931; H.J. Brédo leg.; KMMA • 1♂; Equateur, Eala; Jul 1932; A. Corbissier leg.; KMMA • 1♀; Equateur, Eala; Apr 1933; A. Corbissier leg.; KMMA • 1♂; Equateur, Eala; 1933; A. Corbissier leg.; KMMA • 1♀; South-Kivu, Kabare; 1♀; 31 Jul 1914; J. Bequaert leg.; KMMA • 1♀; Lomami, Kabinda; date unknown; Schwetz leg.; KMMA • 1♂; Kachichwe; 17 Jan 1912; Dr. Bequaert leg.; KMMA • 1♀; Kalemie; Dec 1918; R. Mayné; leg.; KMMA • 1♂; Lualaba; Kabombo; 29 Jun 1947; M. Poll leg.; KMMA • 1♀; Léopoldville [= Kinshasa]; 28 Oct 1951; mevr. Bequaert leg.; KMMA • 1♂; Tshopo, Stanleyville [= Kisangani]; Apr 1915; Lang and Chapin leg.; KMMA • 1♂; Equateur, Lukolela; 17 Jul 1926; J. Bequaert leg.; KMMA • 2♂♂; Lomami, Luputa; Mar 1935; Bouvier leg.; KMMA • 1♀; Natl. Parc Albert, Rwindi, St. Edouard; 17 Apr 1936; L. Lippens leg.; KMMA • 1♀; Tumbalunga, Dibaya; 8 Nov 1930; G.F. de Witte leg.; KMMA • 1♀; Ubani, Bosobolo; 8–11 Jan 1932; H.J. Brédo leg.; KMMA • 1♀; Ubangi, Tungu; 4 Mar 1932; H.J. Brédo leg.; KMMA • 1♀; Uele, Garamba; Jul 1912; Lang and Chapin leg.; KMMA • 1♂ 1♀; Mai-Ndombe, Wimbali; Jul 1913; P. Vanderijst leg.; KMMA • 1♀; 30 Sep 1913; P. Vanderijst leg.; KMMA. Gabon • 1♀; Lolo River; 22 May 1925; J. Rodhain leg.; KMMA. Ghana • 1♀; Nsakjam; 13 Sep 2016; G. Goergen leg.; KMMA • 1♂; Tema; 19 Dec 2004; G. Goergen leg.; IITA • 1♂; Kumasi; 6 Nov 1946; J. Bowden leg.; NMSA. Kenya • 1♂; Kabete; 12 Jun 1916; T.J. Anderson leg.; NHMUK • 1♂; Merifano; Nov 1932; McArthur leg.; NMK • 1♀; Mugura Forest; 2 May 1981; R.H. Markham leg.; NMK • 1♀; Zwani; date unknown; van Someren leg.; CNC. Madagascar • 1♂; Antananarivo; 28 Feb 2016; G. Goergen leg.; IITA. Malawi • 4♂♂ 2♀♀; Chiromo; date unknown; J.E.S. Old. leg.; NHMUK • 1♂; Mount Mulanje; 4 Oct 1913; S.A. Neave leg.; NHMUK • 1♂; Monkey Bay; 12 Dec 1980; J.H.G. Londt leg.; NMSA • 3♂♂; Senga Hills; 1 Dec 1980; B.R. Stuckenberg leg.; NMSA. Mozambique • 1♀; Lourenço-Marques [= Maputo]; date unknown; H.A. Junod leg.; NHMUK • 2♂♂; Sofala, Gorongosa National Park, Chitengo; 20–30 Apr 2004; M. Hauser leg. and H. Rung leg.; CAS • 2♂♂ 1♀; Sofala, Gorongosa National Park, Chitengo; 16–30 Apr 2015; M. Hauser and H. Rung leg.; CAS • 6♂♂; Luabo, Lower Zambezi River; 1 Jun 1957; B.R. Stuckenberg leg.; NMSA • 2♂♂; Luabo, Lower Zambezi River; 1 Jun 1957; P. Usher leg.; NMSA • 1♂; Luabo, Lower Zambezi River; 1 Aug 1957; P. Usher leg.; NMSA • 1♂; Siluwe Hills, W. of Beira; 3 Jun 1964; D. Cookson leg.; NMSA. Nigeria • 1♂; Ibadan; 14 Jun 1957; G.H. Caswell leg.; NHMUK • 1♂; Ibadan; Dec 1988; G. Goergen leg.; IITA. Senegal • 1♀; Dassilamé, Sérére; 30 Nov 2016; S. Cavaillès leg.; SCPC. South Africa • 1♂; Durban; Jul 1903; G. Burn leg.; NMSA • 1♂; KwaZulu-Natal, Durban, Blue Lagoon; 25 May 1991; J.A.W. Lucas leg.; RMNH • 1♂; KwaZulu-Natal, Durban; 14 May 1903; G.F. Leigh leg.; NMSA • 2♂♂; KwaZulu-Natal, Nseleni Nature Reserve; 10 Jan 1994; Natal Museum Staff leg.; NMSA • 2♂♂; KwaZulu-Natal, St. Lucia Park Reserve; 2 Feb 1988; J.H.G. Londt leg.; NMSA • 5♂♂; KwaZulu-Natal, Dukuduku Forest; 18 Jul 1981; B.R. Stuckenberg leg.; NMSA • 1♂; KwaZulu-Natal, Dukuduku Forest, 4♂♂ W of St. Lucia; 26 Nov 1971; M.E. Irwin leg.; NMSA • 1♂; KwaZulu-Natal, Ndumu Game Reserve; 26 Oct 1972; M.E. Irwin leg.; NMSA. Tanzania • 1♂; Kahe, Usambara Mountains; 2 Jun 1916; T.J. Anderson leg.; NHMUK. Togo • 1♀; Kloto Forest; Mar 2004; G. Goergen leg.; IITA • 1♂; Kloto Forest; May 2016; G. Goergen leg.; KMMA. Uganda • 1♀; Busoga; Mar 1906; A. Hodges leg.; CNC • 1♂; Tero Forest, S.E. Buddu; 26–30 Sep 1911; S.A. Neave leg.; NHMUK. Zambia • 1♂ 1♀; Lusaka Province, 8.5 km NW Katondwe; 20 Apr 2016; M. Hauser leg.; CSCA.

**Figures 135–138.**
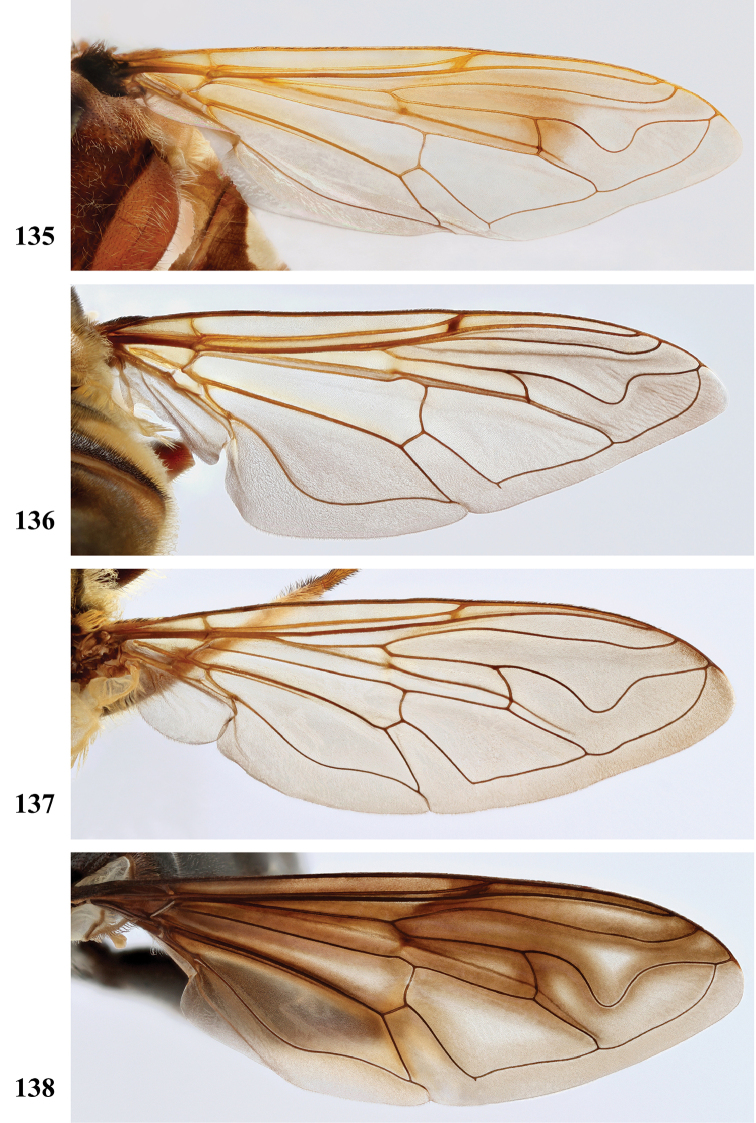
*Mesembrius* spp., right wing **135***M. maculifer* Hull (♀) **136***M. madagascariensis* Keiser (♂) **137***M. minor* (Bezzi) (♂) **138***M. morio* (Bezzi) (♀).

##### Re-description male

**(Fig. 22).** Body length: 11.2–12.5 mm. Wing length: 8.1–8.8 mm.

**Figures 139–142.**
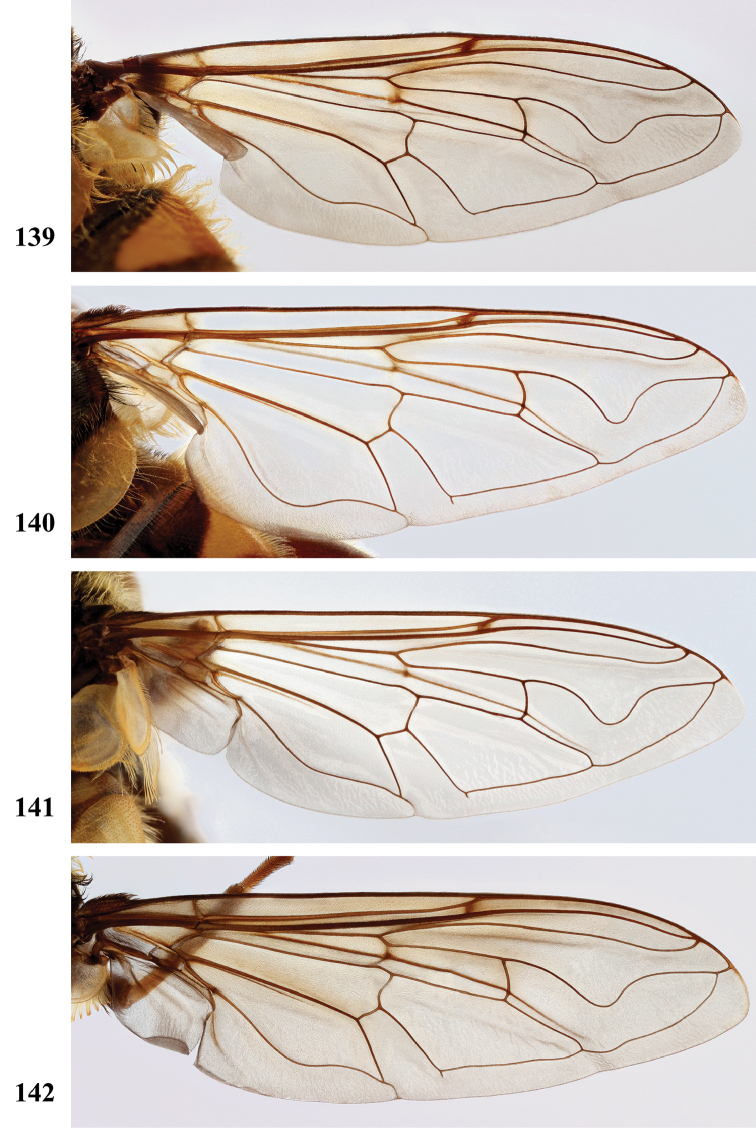
*Mesembrius* spp., right wing **139***M. nigriceps* Curran (♂) **140***M. perforatus* (Speiser) (♂) **141***M. platytarsis* Curran syn. nov. (♂) **142***M. regulus* (Hull) (♂).

***Head*** (Fig. 65). Eyes bare; slightly dichoptic, distance between eyes approx. the width of ocellus. Face white with dark medial vitta; white pilose; white pollinose. Distance between lateral ocellus and eye margin 1/2 width of ocellus. Occiput yellow; yellow pilose; yellow and white pollinose. Frontal triangle black; black pilose; ventral half weakly white pollinose. Frontal prominence shiny black. Antenna black; antennal arista brown.

**Figures 143–146.**
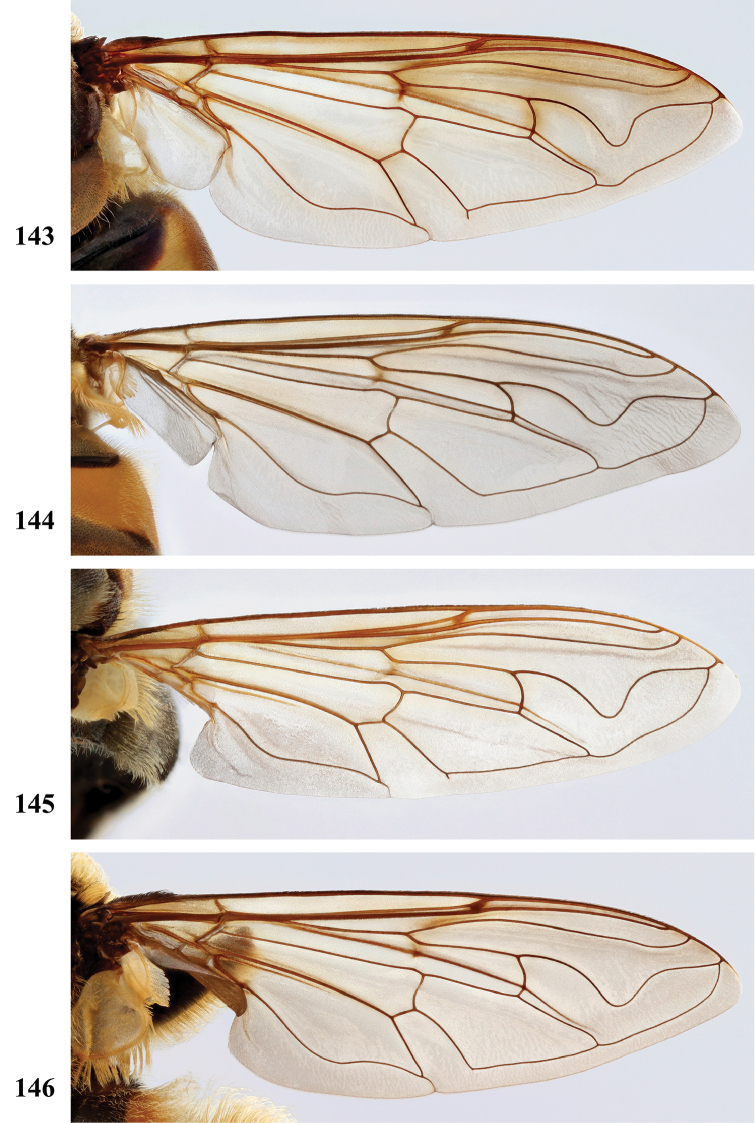
*Mesembrius* spp., right wing **143***M. rex* Curran (♂) **144***M. senegalensis* (Macquart) (♂) **145***M. simplicipes* Curran (♂) **146***M. strigilatus* (Bezzi) (♂).

***Thorax.*** Scutum black with dorsally a pair of weak yellow vittae which fade out posteriorly; with very faint lateral yellow vitta; yellow-rufous pilose. Scutellum uniformly yellow-brown; yellow-rufous pilose, with some short black pile interspersed, especially in the posterior half.

**Figures 147–150.**
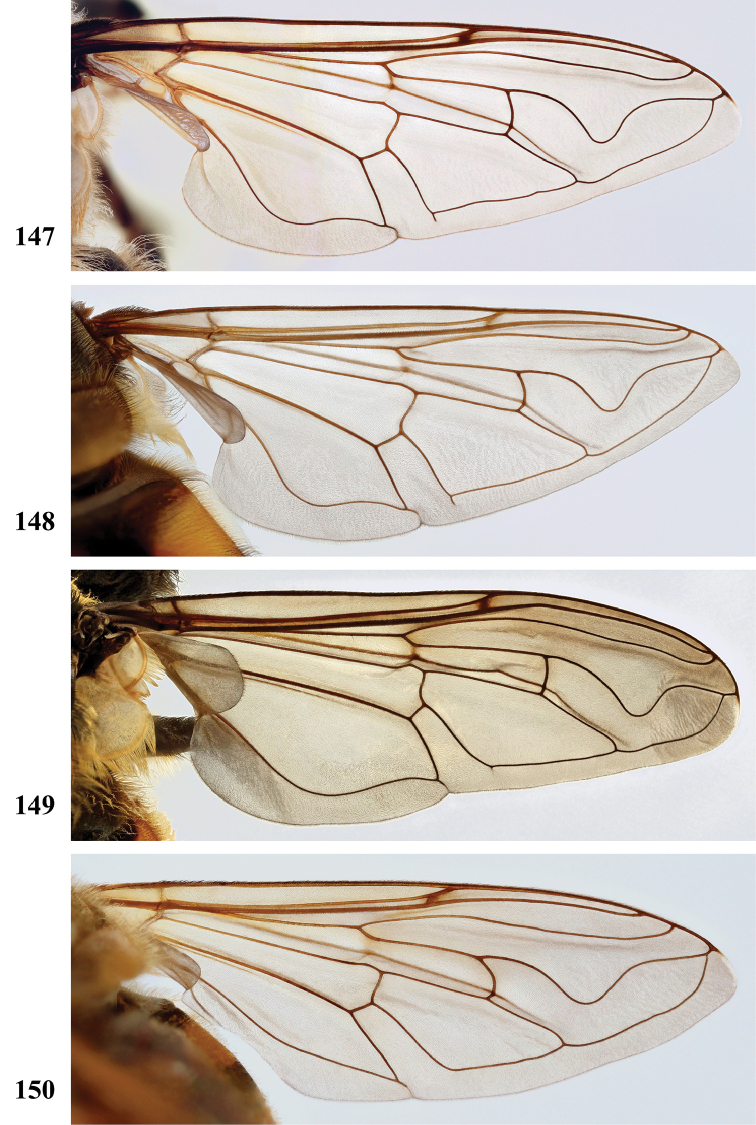
*Mesembrius* spp., right wing **147***M. sulcus* sp. nov. (♂) **148***M. tarsatus* (Bigot) (♂) **149***M. tibialis* sp. nov. (♂) **150***M. vockerothi* sp. nov. (♂).

***Legs.*** Femora and entire metaleg dark brown to black; pro- and mesofemora and tarsi yellow-brown; tarsi without a small darkened medial patch. Proleg: Femur without apical pile brush; yellow pilose ventrally; with long black pile on anterodorsally; with shorter, black pile dorsally. Mesoleg: Femur similar as profemur, but with long, black pile posteriorly and posteroventrally; with black pile anterodorsally which is markedly longer in the proximal half. Metaleg (Fig. 197): Femur with long, yellow pile anteriorly and anteroventrally; with long black pile on the posteroventral distal 1/3; thickened on distal 1/3; no swelling on the mid-section of the ventral side. Tibia strongly curved, especially from posterior view, flattened; with very long black pile dorsally and ventrally.

***Wing*** (Fig. 146). Entire wing uniformly dense microtrichose.

***Abdomen*** (Fig. 102). Tergite II with a pair of very large yellow, rounded maculae; anterior black marking larger in size than posterior black marking, which mostly reach to the lateral tergite sides; medial black marking more or less parallel-sided; long yellow pilose; posterior black marking with short, stiff black pile which extend to the lateral sides. Tergite III with small, triangular to rounded black marking; long yellow pilose with some short thick black pile in medial posterior area; black marking usually strongly white pollinose. Tergite IV entirely dark brown to black, except for anterior 1/5 which is strongly white pollinose; long yellow pilose, the pile is strongly appressed on the lateral sides. Tergite V dark brown.

***Genitalia*** (Fig. 224). Epandrium: Dorsal lobe of surstylus strongly bent, sickle-shaped, short yellow pilose on distal half; with long, thick black setulae at bend ventrally; distal half dorsally convex; densely covered with long yellow pile and with some equally long, but thicker black pile interspersed. Ventral lobe of surstylus bare.

##### Re-description female

**(Fig. 42).** Body length: 11.3–15.0 mm. Wing length: 8.1–9.4 mm.

***Head.*** Eyes bare; dichoptic. Face white with dark medial vitta; white pilose, white pollinose. Frons black on dorsal 2/5, yellow-white on ventral 3/5; black and white pilose on ocellar triangle and just ventrally of ocellar triangle, otherwise white pilose; strongly white pollinose on ventral 3/5, weak white pollinose on dorsal 2/5. Distance between lateral ocellus and eye margin slightly less than width of ocellus. Occiput yellow-white; yellow-white pilose; yellow-white pollinose. Frontal prominence shiny black. Antenna dark brown to black; antennal arista reddish-brown.

***Thorax.*** Scutum dark brown with a pair of dorsolateral yellow pollinose vittae which are connected posteriorly; with lateral, yellow pollinose vitta; sometimes with a fine medial white to yellow pollinose vitta; yellow pilose. Scutellum yellow-orange; yellow pilose.

***Legs.*** Proleg (Fig. 172): Femur black, distal end orange-brown; yellow pilose, with short black pile interspersed. Tibia orange, darkened in distal 1/2; yellow pilose on dorsal proximal half, yellow and black pilose otherwise. Tarsi uniformly dark brown; black pilose dorsally, yellow pilose ventrally; especially the posterior side has very conspicuous thick black pile. Mesoleg: Femur black, distal end orange-brown; black and white pilose. Tibia orange-brown; orange-yellow pilose on dorsal side, black pilose on ventral side. Tarsi orange-brown with darkened dorsal medial area; black pilose. Metaleg: Femur black, distal end orange-brown; orange-yellow pilose, with short, black pile on dorsal distal end, with shorter and thicker black pile on ventral distal half; without ventral swelling in middle (Fig. 203). Tibia orange-brown; orange-yellow pilose with some black pile interspersed at distal end. Tarsi black dorsally, orange ventrally; black pilose dorsally; densely orange pilose ventrally.

***Wing.*** Entire wing uniformly microtrichose.

***Abdomen*** (Fig. 123). Tergite II with a pair of very large, rounded yellow-orange maculae; black pilose on triangular posteromedial section, yellow pilose otherwise; posterior black marking extends to lateral tergite sides; medial part of black marking narrow, approx. 1/10 of tergal width; posterior black marking white pollinose. Tergite III with yellow-orange fascia which occupies entire tergal length on lateral sides and approx. 1/3 of tergal length in medial section; with triangular posterior black marking that extends to the lateral tergite sides; black pilose on triangular posteromedial section, yellow pilose otherwise; posterior black marking white pollinose. Tergite IV as tergite III, but with much narrower yellow-orange fascia (approx. 1/10 of tergal length in medial section). Tergite V with narrow anterior black board; with a pair of yellow-orange maculae in anterolateral corners, otherwise black; yellow pilose; black marking strongly white pollinose, especially in anterior half.

##### Distribution.

Benin, Burundi, Cameroon, Democratic Republic of the Congo, Gabon, Ghana, Guinea-Bissau, Kenya, Madagascar, Malawi, Mozambique, Nigeria, Senegal, South Africa, Tanzania, Togo, Uganda and Zambia.

##### Comments.

See *M. nigriceps*.

#### Mesembrius sulcus

Taxon classificationAnimaliaDipteraSyrphidae

Jordaens, Goergen & De Meyer sp. nov.

762E8C6B-A67D-5D0E-831D-FC9035491346

http://zoobank.org/AD9517F7-E4CA-479A-BC44-B7B9401C1313

Figs 23 , 43 , 66 , 80 , 103 , 124 , 147 , 155 , 164 , 176 , 187 , 225

##### Differential diagnosis.

*Mesembrius sulcus* sp. nov. males have an apical pile brush on the profemur of thick and dense black pile dorsally and yellow pile ventrally. The metafemur is very long and slender and has long yellow pile throughout and shorter, yellow and black pile on the ventral side. The metatibia has a deep groove on the posterior proximal half which is bordered by long black pile. The probasitarsus has a tuft of long black pile on the posterior side. The male differs from any other species in the colour of the apical pile brush (except from *M. tibialis* sp. nov.) which is black dorsally and golden-yellow ventrally (yellow-orange in *M. chapini*; dark-brown to black in other species). It differs from *M. tibialis* sp. nov. in the entirely orange probasitarsus (orange and black in *M tibialis* sp. nov.), in the presence of a deep groove in the posterior proximal half of the metatibia (absent in *M. tibialis* sp. nov.) and in the unmodified mesotibia (proximal half strongly compressed in *M. tibialis* sp. nov.). Females have a frons which is black pilose on its entire length, except laterally. It can be distinguished from other such females (except from *M. tarsatus*) by the black legs, including the tibiae (tibiae yellow-brown in other species). It differs from the female of *M. tarsatus* in tergite II, which has a pair of small yellow-orange maculae that laterally reach to halfway of the tergite length (almost to posterior end in *M. tarsatus*) and in tergite III which has a pair of vague anterolateral yellow-orange maculae (clear pair of maculae in *M. tarsatus*).

##### Examined material.

*Mesembrius sulcus* Jordaens, Goergen & De Meyer: Holotype, male, “HOLOTYPUS” “MUSÉE DU CONGO // Ituri: Nioka // -VII-1934 // J. Leroy” “van Doesburg det., 1956 // Mesembrius // spec.?nov. ♂” “RMCA ENT // 000030186” [KMMA].

***Paratypes*:** Malawi • 1♂; Mount Mulanje; 17 Oct 1913; S.A. Neave leg.; NHMUK 013428977 • 1♂; Zomba; Feb 1911; J.E.G. Old. Leg.; NHMUK • 2♂♂; Mount Mulanje; 17 Oct 1913; S.A. Neave leg.; NHMUK. Kenya • 3♂♂ 3♀♀; Nairobi, Karura Forest; 2 Dec 2017; K. Jordaens leg.; ICIPE. South Africa • 1♂; Port St. Johns; 1–31 Oct 1969; E. and W. Gess leg.; AMGS.

##### Description male

**(Fig. 23).** Body length: 14.0–15.6 mm. Wing length: 10.2–11.5 mm

***Head*** (Fig. 66). Eyes bare; holoptic, eye contiguity approx. as long as length of ocellar triangle. Face yellow to orange with dark medial vitta; white pilose; white pollinose. Vertical triangle black; black pilose; yellow pollinose on medium third. Distance between lateral ocellus and eye margin slightly less than width of ocellus. Occiput yellow; yellow pilose with some shorter and thicker black pile near eye margin; yellow and white pollinose. Frontal triangle short; yellow-white; with some long, yellow and black pile; white pollinose. Frontal prominence shiny black with orange-brown apex. Antenna, scape and pedicel reddish-brown; postpedicel black, white pollinose; antennal arista reddish-brown.

***Thorax.*** Scutum black with dorsally a pair of very faint white pollinose vittae; lateral white pollinose vitta very faint; yellow pilose. Scutellum uniformly yellow-brown; long yellow pilose with some very short black pile on posterior half.

***Legs.*** All legs chocolate-brown to black, but protarsus orange. Proleg (Figs 155, 164): Femur dorsoventrally flattened; with long yellow pile posterodorsally; with apical pile brush of thick black pile dorsally and thick yellow pile ventrally; with short and black thick pile at proximal end. Basitarsus orange; with a tuft of black pile posteriorly. Other tarsi orange; with sparse short black pile. Mesoleg (Fig. 176): Femur, ventrally with long yellow pile on proximal 2/3 and shorter black pile on distal 1/3, with some long black pile interspersed in middle section. Metaleg (Fig. 187): Femur long and thin, slightly curved; with yellow pile anterodorsally; with some denser shorter and black pile at distal ventral end. Tibia with long black pile; posteriorly with deep and broad excavation in proximal half which is bordered with long black pile.

***Wing*** (Fig. 147). Entire wing uniformly dense microtrichose.

***Abdomen*** (Fig. 103). Tergite II with a pair of very large, yellow, more or less triangular maculae; black marking hourglass-shaped; posterior and anterior black marking slightly connected in the middle; posterior marking with strong white pollinosity; yellow pilose, except for black pile at posterior border of posterior black marking. Tergite III and IV with orange fascia; black marking on posterior half strong white pollinose; black pilose in medial part of black marking, yellow pilose on orange fascia and lateral parts of black marking.

***Genitalia*** (Fig. 225). Epandrium: Dorsal lobe of surstylus broadly rounded; with short black spines on almost entire surface; dorsally long yellow pilose. Ventral lobe of surstylus straight; bare.

##### Description female

**(Fig. 43).** Body length: 11.2–13.4 mm. Wing length: 12.7–16.6 mm.

***Head*** (Fig. 80). Eyes bare; dichoptic. Face white with dark medial vitta; white pilose; white pollinose. Frons black; black pilose in dorsal half and medial ventral half, yellow pilose on lateral sides of ventral half; yellow-white pollinose, especially in ventral half. Distance between lateral ocellus and eye margin 1½× width of ocellus. Occiput black; yellow pilose, with some black pile near eye margin; yellow-white pollinose. Frontal prominence shiny black. Antenna, scape and pedicel orange-brown; postpedicel black; antennal arista reddish-brown.

***Thorax.*** Scutum dark brown to black with dorsally a pair of vague brown pollinose vittae which are connected posteriorly; yellow pilose with some black pile interspersed. Scutellum dark brown with lighter posterior border; yellow pilose.

***Legs.*** All femora, tibiae and metatarsi dark brown to black, except for extreme distal ends which are reddish-brown (as in *M. tarsatus*; Fig. 168); yellow pilose; metatibia with short black spines on posteroventral 1/3. Pro- and mesotarsi orange; most distal tarsomere darkened distally, sometimes all tarsi darkened; dorsally short black pilose, ventrally short yellow pilose.

***Wing.*** Entire wing uniformly dense microtrichose.

***Abdomen*** (Fig. 124). Tergite II black; yellow pilose with some very short thick black pile on black markings; with a pair of L-shaped, small orange maculae; white pollinose, especially in the central area of the posterior black marking. Tergite III black; yellow pilose with short thick black pile interspersed, especially in the posterior half; with a pair of small orange maculae in the anterolateral corners. Tergite IV as tergite III, but without orange maculae. Tergite V black; yellow pilose.

##### Distribution.

Democratic Republic of the Congo, Kenya, Malawi and South Africa.

##### Comments.

This is a new species to the Afrotropical Region with a relatively wide distribution. The species morphologically resembles *M. tarsatus*, which appears to be its sister species (see Fig. 230), but the males differ markedly in the morphology of the metafemur. Females are very similar to females of *M. tarsatus*. The mean *p*-distance for the DNA barcoding is relatively low (1.6 %), but differences are consistent (i.e. no barcodes are shared between the species) (Fig. 229).

##### Etymology.

The specific epithet *sulcus* (Latin) means groove (noun in apposition) and was chosen with reference to the deep groove on the metatibia. It is to be treated as an adjective (nominative singular masculine).

#### Mesembrius tarsatus

Taxon classificationAnimaliaDipteraSyrphidae

(Bigot, 1883)

92B41985-235A-5D4C-8A99-00262C4BF2F1

Figs 2 , 24 , 44 , 67 , 81 , 104 , 125 , 148 , 159 , 168 , 189 , 226

Prionotomyia tarsata Bigot, 1883: CXXI.
Prionotomyia tarsata – Kertész (1910): 266 – Speiser (1913): 128.
Mesembrius tarsata – Curran (1939): 9.
Mesembrius tarsatus – Smith and Vockeroth (1980): 504.

##### Differential diagnosis.

*Mesembrius tarsatus* males are holoptic, have a loose black apical pile brush on the profemur, a black scutum with, dorsally, a pair of weakly-demarcated yellow pollinose vittae, an orange probasitarsus with a tuft of black pile on the posterior side and two black spots on the most distal tarsomere and a slender metatibia with a swelling in the posterior medial half. It can be distinguished from any other species by the apical pile brush of the profemur which is loose and entirely black (black dorsally, yellow ventrally in *M. arcuatus* sp. nov.; yellowish with some black pile interspersed in *M. ingratus*) and by the rounded swelling on the metatibia (strongly compressed in *M. arcuatus* sp. nov.; with a deep groove in *M. ingratus*). Females have a frons which is black pilose on its entire length, except laterally. It can be distinguished from other such females (except from *M. sulcus* sp. nov.) by the black legs, including the tibiae (tibiae yellow-brown in other species). It differs from the female of *M. sulcus* sp. nov. in tergite II which has a pair of large yellow-orange maculae which, laterally, reach to almost the posterior end (small pair of yellow-orange maculae which, latteraly, reach to halfway of the tergite length *M. sulcus* sp. nov.) and in tergite III which has a pair of clear anterolateral yellow-orange maculae (vague pair of maculae in *M. sulcus* sp. nov.).

##### Examined material.

*Prionotomyia tarsata* Bigot: Lectotype (hereby designated), male, “LECTOTYPUS” “SYN- // TYPE” “Prionotomyia // tarsata Big.” “Prionotomyia ♂ // tarsata Bigot // Senegal” “ex. coll. Bigot, // Press. by // G.H. Verrall. // B.M. 1894234” “BMNH(E) # // 230741” “NHMUK 010369820” [NHMUK]. Paralectotype, male, “SYN- // TYPE” “Prionotomyia // tarsata Big.” “Prionotomyia ♂ // tarsata Bigot // Senegal” “ex. coll. Bigot, // Press. by // G.H. Verrall. // B.M. 1894234” “BMNH(E) # // 230742” “NHMUK 010369821” “PARA- // LECTOTYPUS” [NHMUK].

##### Other material

 Democratic Republic of the Congo • 1♀; Banningville [= Bandundu], Kwilu River, Panga; Aug 1945; Fain leg.; RMNH • 1♀; Haut-Katanga, Kasenga; 5 Mar 1912; J. Bequaert leg.; KMMA • 1♂; South-Kivu, Musingiro; 8 Oct 1922; Ch. Seydel leg.; KMMA • 1♀; Tshibinda; 21–27 Aug 1931; T.D.A. Cockerell leg.; NHMUK • 1♀; Eala; 5 Oct 1935; J. Ghesquière leg.; RMNH • 1♀; Eala; Aug 1936; J. Ghesquière leg.; RMNH • 1♀; Eala; Mar 1935; J. Ghesquière leg.; RMNH • 1♀; Eala; Oct 1936; J. Ghesquière leg.; RMNH • 1♀; Lulua, Kapanga; Nov 1928; Walker leg.; RMNH • 1♂; Lulua, Kapanga; Aug 1932; F.G. Overlaet leg.; RMNH. Kenya • 1♂; Naivasha, Fisherman’s Camp; 14 Mar 1993; M. De Meyer leg.; NMK • 4♂♂ 2♀♀; Nairobi; Mar 1928; van Someren leg.; KMMA • 1♂; Nairobi; 20 Mar 1921; A.F.J. Gedye leg.; NMK • 7♂♂ 2♀♀; Nairobi, Karura Forest; 23 Nov 2017; PINDIP course leg.; KMMA. Malawi • 1♂; Mulanje Mountain; 26 Nov 1912; S.A. Neave leg.; NHMUK • 1♂ 1♀; Zomba Plateau; 1 Dec 1911; collector unknown; NHMUK • 3♂♂; Zomba Plateau; 24 Nov 1980; B.R. Stuckenberg leg.; NMSA • 2♂♂; Zomba Plateau; date unknown; H.S. Stannus leg.; NHMUK • 1♂; Zomba Plateau; 24–27 Nov 1980; J.G.H. Londt and B. Stuckenberg leg.; NMSA • 7♂♂ 6♀♀; Zomba, Kuchawe Trout Farm; 8–11 Nov 2016; K. Jordaens leg.; KMMA. Senegal • 2♂♂; locality and date unknown; Bigot leg.; NHMUK • 1♂; Nema Ba; 10 Nov 2016; S. Cavaillès leg.; SCPC. South Africa • 2♂♂; Barber Nature Reserve; 7 Oct 2015; A. Vujić et al. leg.; UNS • 1♂; KwaZulu-Natal, Dukuduku Forest Reserve; 18–19 Jul 1981; J.G.H. Londt and B. Stuckenberg leg.; NMSA • 1♂; KwaZulu-Natal, Umlalazi Nature Reserve; 8 Nov 1997; J.G.H. Londt and A. Londt leg.; NMSA • 1♂; KwaZulu-Natal, Bluff Nature Reserve; 3 Sep 2018; J. Midgley leg.; NMSA • 1♂; KwaZulu-Natal, Durban; 23 Apr 1920; C.N. Barker leg.; DMSA • 1♂; KwaZulu-Natal, Durban; 8 Feb 1919; C.N. Barker leg.; DMSA • 1♂; KwaZulu-Natal, Durban; Nov–Dec1945; H.W. Bell Marley leg.; DMSA • 1♂; KwaZulu-Natal, Pietermaritzburg, Botanical Gardens; 15 Nov 2018; J. Midgley leg.; NMSA • 1♂; KwaZulu-Natal, Ngoye Forest Reserve; 29 Jan 1968; J.G.H. Londt leg.; NMSA • 1♂; KwaZulu-Natal, Ubombo Mountain Reserve; 11 Oct 2019; D. Brothers leg.; NMSA • 1♂; KwaZulu-Natal, Umlalazi Nature Reserve; 8 Nov 1997; J.G.H. Londt leg.; NMSA • 1♂; KwaZulu-Natal, Dukuduku Forest, 4♂♂ W of St. Lucia; 26 Nov 1971; M.E. Irwin leg.; NMSA. Uganda • 1♂; between Jinja and Bussia; 28 Jul–1 Aug 1911; S.A. Neave leg.; NHMUK • 1♀; Jinja; Oct 1930; van Someren leg.; NHMUK • 2♂♂ 1♀; between Sewiza and Kampala; 27–31 Aug 1911; S.A. Neave leg.; NHMUK • 1♂; Entebbe; 17 Aug 1911; C.C. Gowdey leg.; NHMUK • 1♂ 2♀♀; Entebbe; 21 Aug 1911; C.C. Gowdey leg.; NHMUK • 1♀; Entebbe; 31 Aug 1911; C.C. Gowdey leg.; NHMUK • 1♂; Entebbe; 18–20 Nov 1911; C.C. Gowdey leg.; NHMUK • 1♀; Entebbe; 11 Aug 1912; C.C. Gowdey leg.; NHMUK • 1♂; Entebbe; 16 Aug 1912; C.C. Gowdey leg.; NHMUK • 2♂♂; Entebbe; 17 Aug 1912; C.C. Gowdey leg.; NHMUK • 2♂♂; Entebbe; 27 May 1912; C.C. Gowdey leg.; NHMUK • 3♂♂ 1♀; Entebbe; 7–9 May 1912; C.C. Gowdey leg.; NHMUK • 2♂♂ 2♀♀; Entebbe; 3 Nov 1912; C.C. Gowdey leg.; NHMUK • 1♂ 1♀; Entebbe; 14 Nov 1912; C.C. Gowdey leg.; NHMUK • 6♂♂ 1♀; Entebbe; 18–20 Nov 1912; C.C. Gowdey leg.; NHMUK • 1♂; Entebbe; 13 Oct 1912; C.C. Gowdey leg.; NHMUK • 1♂ 1♀; Entebbe; 16 Oct 1912; C.C. Gowdey leg.; NHMUK • 1♂; Entebbe; 3 Sep 1912; C.C. Gowdey leg.; NHMUK • 1♂; Entebbe; 18–20 Nov 1911; C.C. Gowdey leg.; NHMUK • 1♂; Entebbe; 1–14 Sep 1912; C.A. Wiggins leg.; NHMUK • 1♂; Entebbe; 1–11 Sep 1911; S.A. Neave leg.; NHMUK • 1♂; South of Maseka, Katera Forest; May 1972; E.B. Babyetagara leg.; CNC • 5♂♂ 2♀♀; N.W. shores of Vic. Nyanza; 12–15 Sep 1911; S.A. Neave leg.; NHMUK • 5♂♂ 3♀♀; Northern Buddu; 16–18 Sep 1911; S.A. Neave leg.; NHMUK • 1♂; Nsoje River; 2 Mar 1911; van Someren leg.; NHMUK • 1♂; N. Ankole, Nyrbthozi; 21 Jan 1975; M.K. Paulus leg.; CNC • 1♀; South of Lake George; 17–19 Oct 1911; S.A. Neave leg.; NHMUK • 1♂; S.E. Ankole; 4–8 Oct 1911; S.A. Neave leg.; NHMUK • 1♂; Tero Forest; 8 Jul 1912; C.C. Gowdey leg.; NHMUK • 2♂♂; S.E. Buddu, Tero Forest; 26–30 Sep 1911; S.A. Neave leg.; NHMUK • 1♀; Toro, Duro River; 6 Mar 1911; van Someren leg.; NHMUK • 1♂ 1♀; Toro, Duro River; 12 Mar 1911; van Someren leg.; NHMUK • 1♂; Tororo; 25 Jan 1967; F.K. Masasai leg.; NHMUK • 1♂; District West Uganda, W. Ankole; 19–24 Apr 1973; H. Falke leg.; CNC • 1♂; District West Uganda, W. Ankole; 30 Dec 1975; M.K. Paulus leg.; CNC • 1♀; locality and date unknown; R.C. Bradley leg.; NHMUK. Zambia • 1♂; Lake Bangweulu, Kapola, N. of Kapata; 27 Oct 1946; collector unknown; NHMUK. Zimbabwe • 1♂; Mporokoso; 2 Aug 1909; S.A. Neave leg.; OXUM.

##### Re-description male

**(Fig. 24).** Body length: 13.1–15.4 mm. Wing length: 9.7–10.9 mm.

***Head*** (Fig. 67). Eyes bare; holoptic, eye contiguity somewhat shorter than length of ocellar triangle. Face white with dark medial vitta; white pilose; white pollinose. Vertical triangle black; black pilose; yellow pollinose on ventral half. Distance between lateral ocellus and eye margin 1/2 width of ocellus. Occiput yellow; yellow pilose, with some shorter and thicker black pile near eye margin; yellow and white pollinose. Frontal triangle short; yellow-white; with some long, black pile; yellow pollinose. Frontal prominence shiny black with orange-brown apex. Antenna black; postpedicel white pollinose; antennal arista reddish-brown.

**Figures 151–154.**
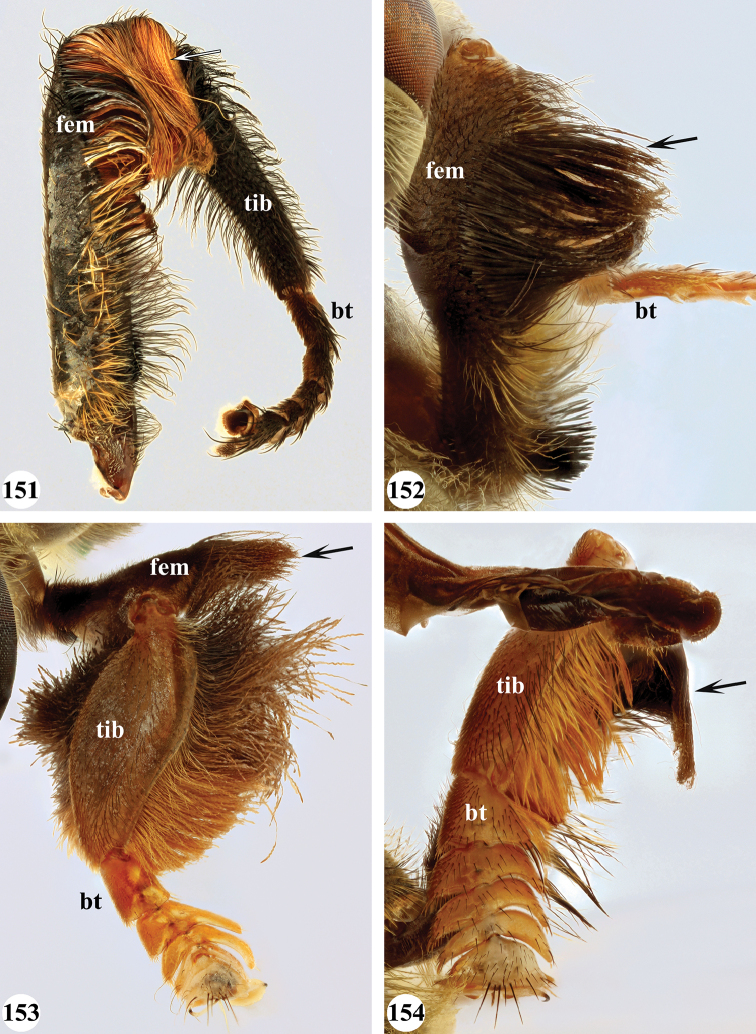
*Mesembrius*, proleg, dorsal view **151***M. chapini* Curran (♂) **152***M. perforatus* (Speiser) (♂) **153***M. regulus* (Hull) (♂) **154***M. rex* Curran (♂). Abbreviations: bt-basitarsus, fem-femur, tib-tibia.

***Thorax.*** Scutum black with, dorsally, a pair of weakly-demarcated yellow pollinose vittae which fade out posteriorly; lateral yellow pollinose vitta very faint to absent; yellow and black pilose. Scutellum dark brown to black with a lighter posterior border; with long yellow pile and shorter black pile, the latter most prominent in the posterior half.

**Figures 155–158.** *Mesembrius*, proleg, dorsal view **155***M. sulcus* sp. nov. (♂) **156***M. tibialis* sp. nov. (♂) **157***M. arcuatus* sp. nov. (♂) **158***M. ingratus* (Loew) (♂). Abbreviations: bt-basitarsus, fem-femur, tib-tibia.

**Figures 159–162.**
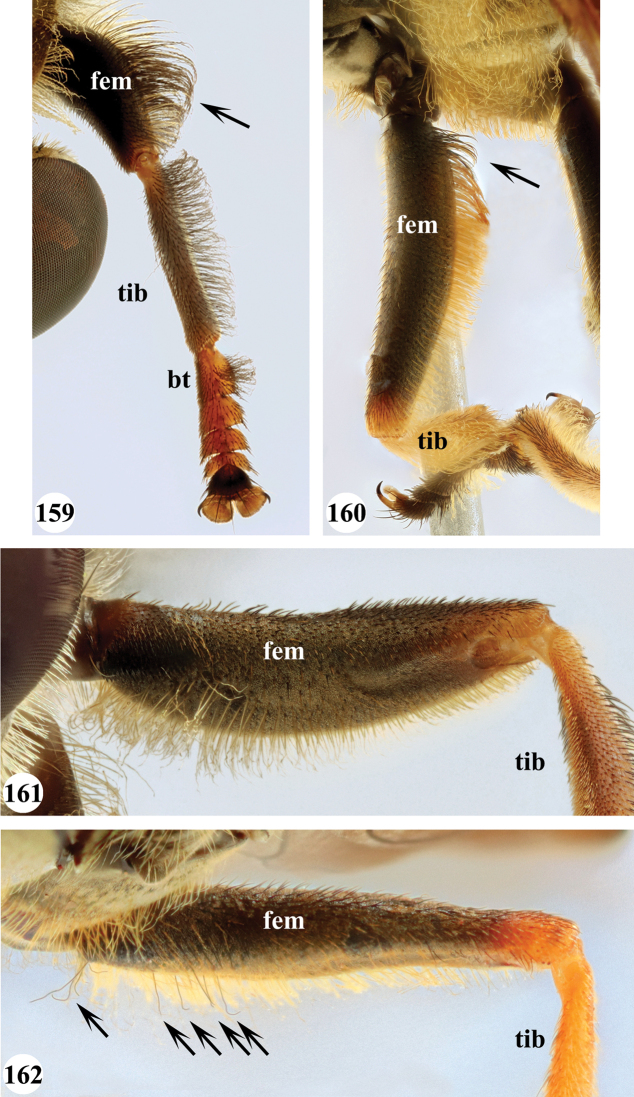
*Mesembrius* spp., proleg, dorsal view **159***M. tarsatus* (Bigot) (♂) **160***M. capensis* (Macquart) (♂) **161***M. senegalensis* (♂) **162***M. longipilosus* sp. nov. (♂). Abbreviations: bt-basitarsus, fem-femur, tib-tibia.

***Legs*** (Fig. 168). All legs black, but protarsus reddish-brown; black pilose. Proleg (Fig. 159): Femur dorsoventrally flattened; posterodorsal side with yellow, long pile and some long black pile in proximal half; with apical pile brush of long, relatively loose and curved black pile; with long black pile anteroventrally. Basitarsus orange; with a tuft of black pile on posterior side and two black spots on the most distal tarsomere. Mesoleg: Femur with long yellow and black pile. Metaleg (Fig. 189): Femur with long and thin yellow pile; with some black pile towards distal end; with a patch of shorter and thicker black pile ventroproximally. Tibia with long black pile, especially in distal 2/3; ventrally with a swelling on distal 1/3.

***Wing*** (Fig. 148). Entire wing uniformly dense microtrichose.

***Abdomen*** (Fig. 104). Tergite II with a pair of very large yellow-orange rounded maculae; black marking hourglass-shaped; posterior black marking equal in size to anterior black marking and with a medial white pollinose area; yellow pilose, but black pilose on posterior black marking and on posterolateral corners. Tergite III and IV with broad yellow-orange fascia, with large black, white pollinose marking on posterior 2/3; black pilose on black marking and adjacent parts of yellow-orange fascia, yellow pilose on remainder of yellow fascia and on lateral sides.

**Figures 163–165.**
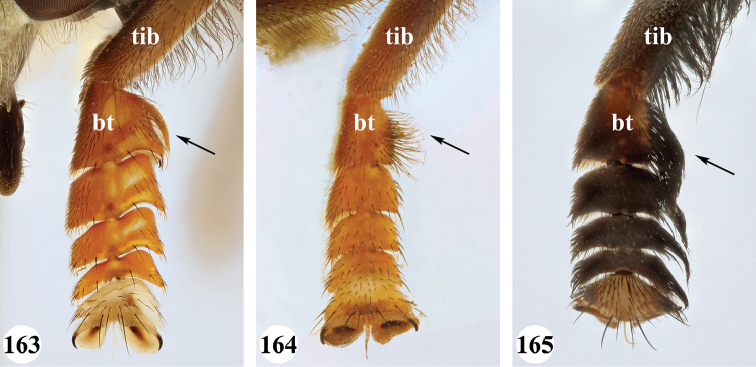
*Mesembrius* spp., proleg, dorsal view **163***M. perforatus* (Speiser) (♂) **164***M. sulcus* sp. nov. (♂) **165***M. tibialis* sp. nov. (♂). Abbreviations: bt-basitarsus, tib-tibia.

***Genitalia*** (Fig. 226). Epandrium: Dorsal lobe of surstylus broadly rounded; with short black spines on almost entire surface; dorsally long yellow pilose. Ventral lobe of surstylus straight; bare.

##### Description female

**(Fig. 44).** Body length: 11.0–13.2 mm. Wing length: 12.7–15.6 mm. Similar to the female of *M. sulcus* sp. nov., but the maculae on abdominal tergite II are larger and triangular (Fig. 125). Head as in Fig. 81.

##### Distribution.

Democratic Republic of the Congo, Kenya, Malawi, Senegal, South Africa, Uganda, Zambia and Zimbabwe.

**Figures 166–171.**
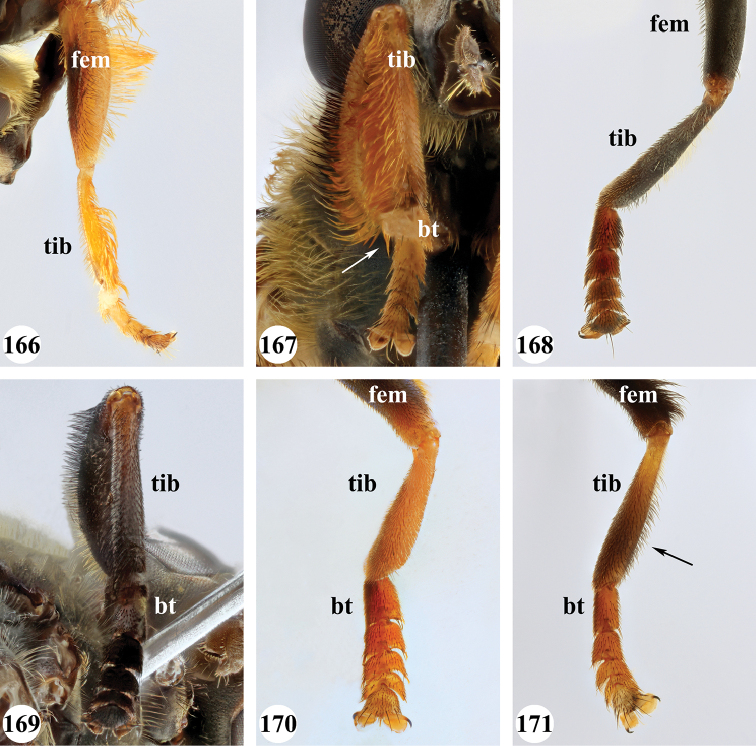
*Mesembrius* spp., proleg, dorsal view **166***M. simplicipes* Curran (♂) **167***M. platytarsis* Curran syn. nov. (♂) **168***M. tarsatus* (Bigot) (♀) **169***M. chapini* Curran (♀) **170***M. rex* Curran (♀) **171***M. regulus* (Hull) (♀). Abbreviations: bt-basitarsus, fem-femur, tib-tibia.

##### Comments.

See *M. sulcus* sp. nov.

**Figures 172–176.**
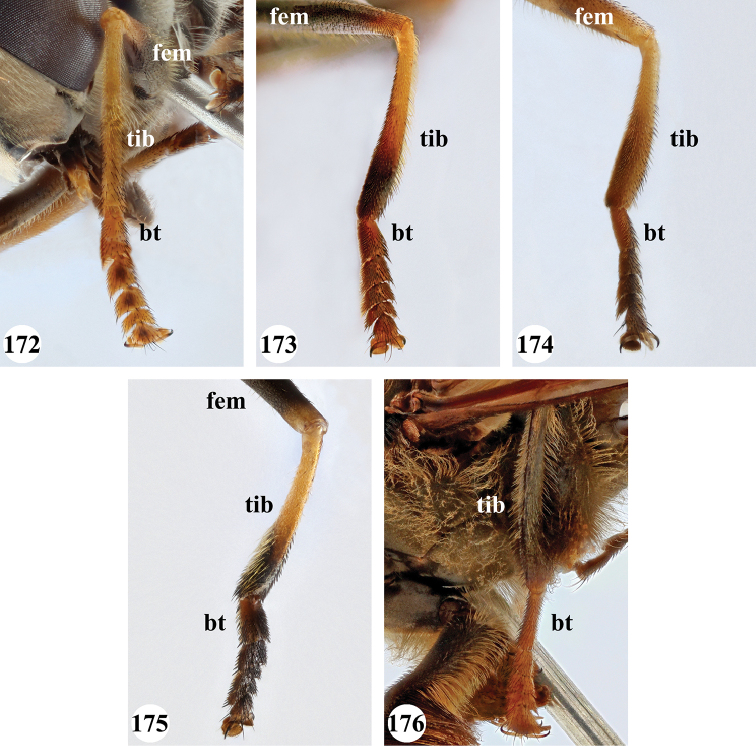
*Mesembrius* spp., proleg, dorsal view **172***M. strigilatus* (Bezzi) (♀) **173***M. minor* (Bezzi) (♀) **174***M. senegalensis* (Macquart) (♀) **175***M. caffer* (Loew) (♀). Mesoleg, posterior view **176***M. sulcus* sp. nov. (♂). Abbreviations: bt-basitarsus, fem-femur, tib-tibia.

#### Mesembrius tibialis

Taxon classificationAnimaliaDipteraSyrphidae

Jordaens, Goergen & De Meyer sp. nov.

6A80FA85-551F-5011-BCC3-E82CBBEEFAD3

http://zoobank.org/EB6378B6-C7E0-490B-B491-EC947BF4436A

Figs 25 , 68 , 105 , 149 , 156 , 165 , 177 , 188 , 227

##### Differential diagnosis.

*Mesembrius tibialis* sp. nov. males have an apical pile brush on the profemur of thick, dense black pile dorsally and yellow pile ventrally. The metafemur is very long and slender and has long yellow pile and shorter yellow and black pile on the ventral side. The metatibia has no groove on the posterior side. The probasitarsus has a tuft of long black pile. The mesotibia is curved and the proximal half is compressed. The male differs from any other species in the colour of the apical pile brush (except from *M. sulcus* sp. nov.) which is black dorsally and golden-yellow ventrally (yellow-orange in *M. chapini*; dark-brown to black in other species). It differs from *M. sulcus* sp. nov. in the orange and black probasitarsus (orange in *M. sulcus* sp. nov.), in the absence of a deep groove in the posterior proximal half of the metatibia and in the strongly compressed mesotibia (unmodified in *M. sulcus* sp. nov.). The female is unknown.

**Figures 177–180.**
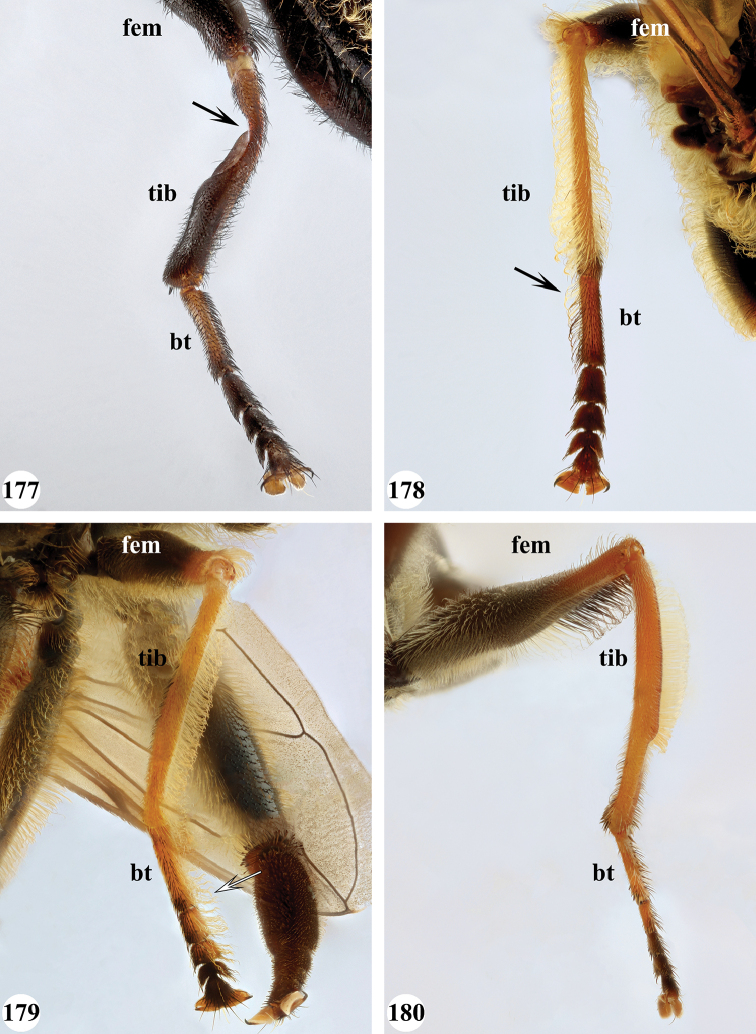
*Mesembrius* spp., mesoleg, dorsal view **177***M. tibialis* sp. nov. (♂) **178***M. caffer* (Loew) (♂) **179***M. capensis* (Macquart) (♂) **180***M. madagascariensis* Keiser (♂). Abbreviations: bt-basitarsus, fem-femur, tib-tibia.

##### Examined material.

*Mesembrius tibialis* Jordaens, Goergen & De Meyer: Holotype, male “HOLOTYPUS” “Togo, Kloto Forest // II.2017 // leg. G. Goergen” “Mesembrius tibialis // Det. K. Jordaens” “DNA 1149A04 // K. Jordaens // RMCA 2019” [KMMA].

**Figures 181–184.**
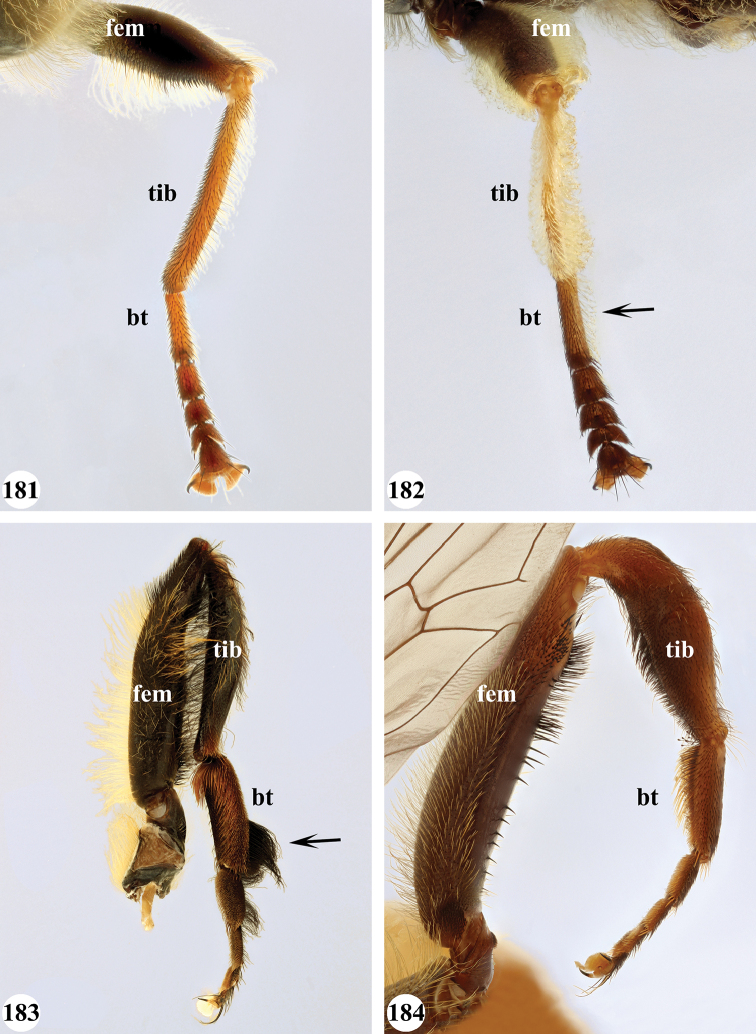
*Mesembrius* spp., mesoleg, dorsal view **181***M. senegalensis* (Macquart) (♂) **182***M. caffer* (Loew) (♂). Metaleg, posterior view **183***M. regulus* (Hull) (♀) **184***M. rex* Curran (♀). Abbreviations: bt-basitarsus, fem-femur, tib-tibia.

***Paratypes*:** Togo • 2♂♂; Kloto Forest; Dec 2017; G. Goergen leg.; IITA.

**Figures 185–188.**
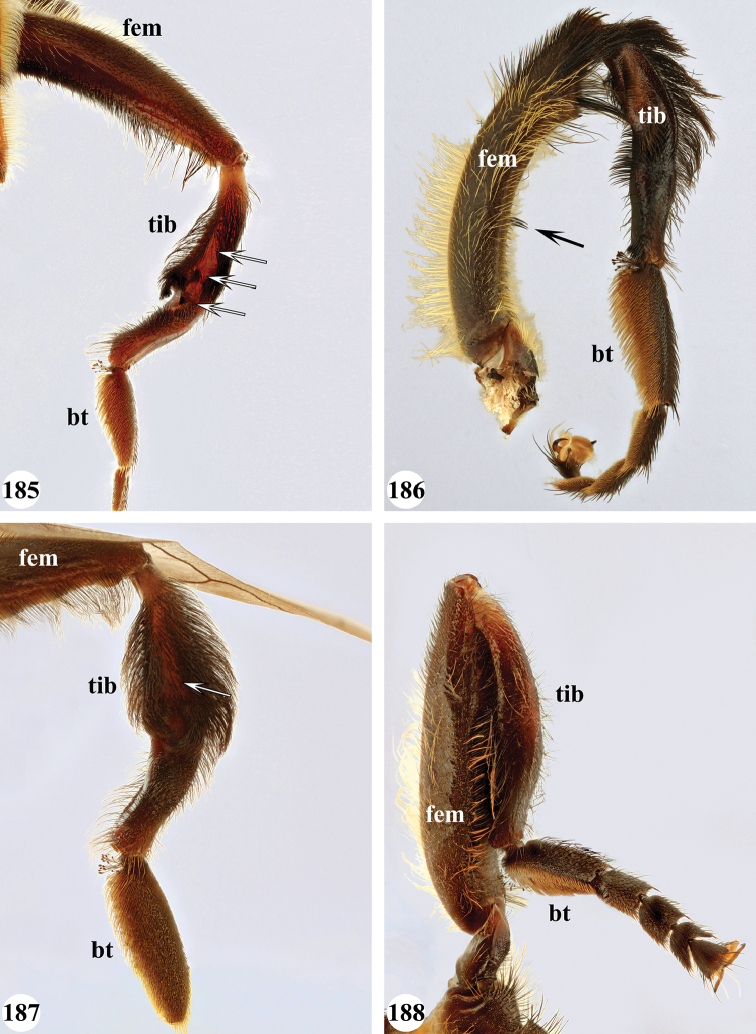
*Mesembrius* spp., mesoleg, dorsal view **185***M. perforatus* (Speiser) (♀). Metaleg, posterior view **186***M. chapini* Curran (♂) **187***M. sulcus* sp. nov. (♂). Metaleg, ventral view **188***M. tibialis* sp. nov. (♂). Abbreviations: bt-basitarsus, fem-femur, tib-tibia.

##### Description male

**(Fig. 25).** Body length: 14. mm. Wing length: 12.7 mm.

***Head*** (Fig. 68). Eyes bare; holoptic, eye contiguity approx. as long as length of ocellar triangle. Face yellow to orange with dark medial vitta; white pollinose; yellow-white pilose. Vertical triangle black; black pilose; yellow pollinose on medium third. Distance between lateral ocellus and eye margin slightly less than width of ocellus. Occiput black; yellow pilose with some shorter and thicker black pile near eye margin; white pollinose. Frontal triangle short; yellow-white; with some long black pile; white pollinose. Frontal prominence shiny black. Antenna black; postpedicel white pollinose; antennal arista orange-brown.

**Figures 189–192.**
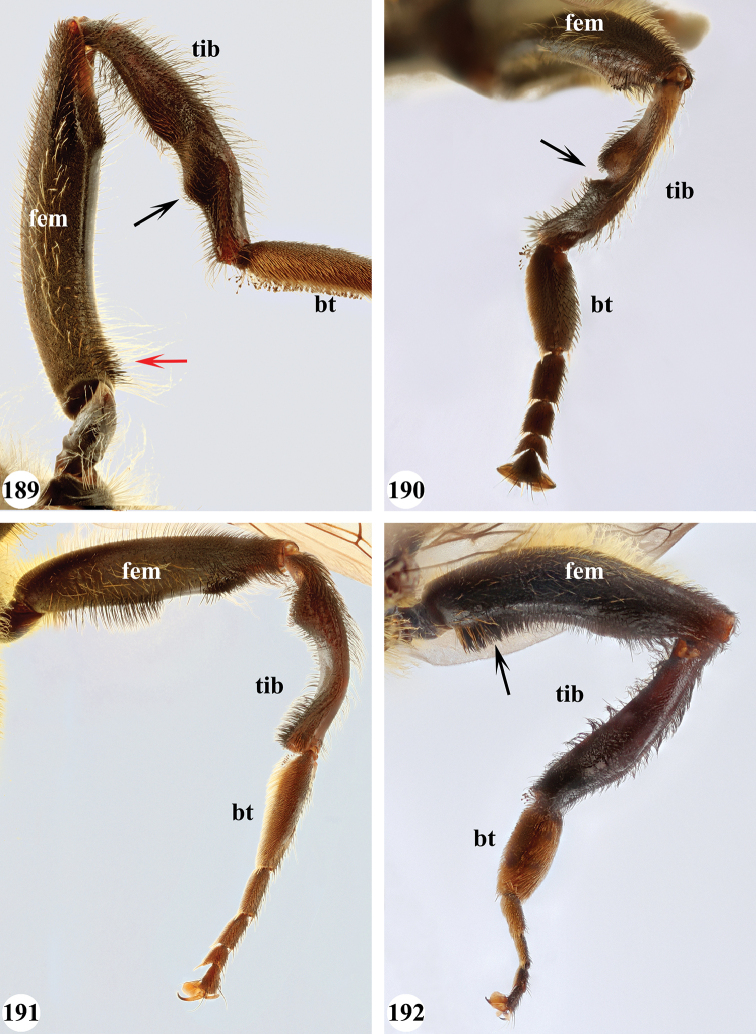
*Mesembrius* spp., metaleg, posterior view **189***M. tarsatus* (Bigot) (♂) **190***M. ingratus* (Loew) (♂) **191***M. arcuatus* sp. nov. (♂) **192***M. nigriceps* Curran (♂). Abbreviations: bt-basitarsus, fem-femur, tib-tibia.

***Thorax.*** Scutum black with dorsally, in the anterior half, a pair of very faint yellow pollinose vittae. Scutellum black in anterior half, yellow-brown in posterior half; yellow pilose with, in the posterior half, some shorter black pile interspersed.

**Figures 193–196.**
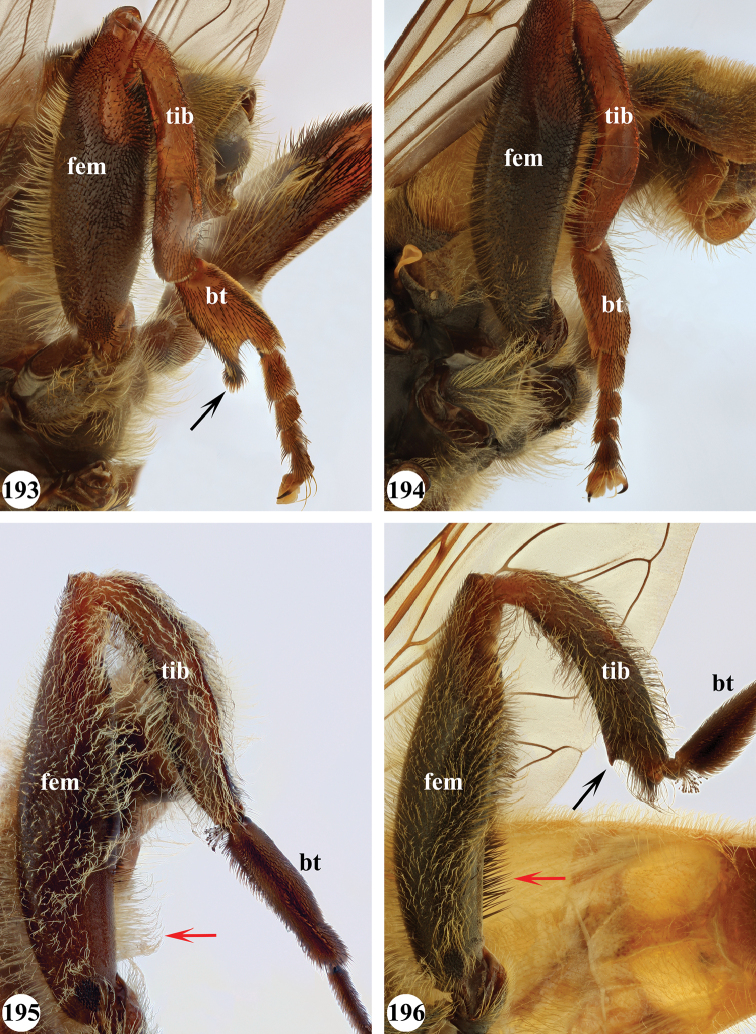
*Mesembrius* spp., metaleg, dorsal view **193***M. platytarsis* Curran syn. nov. (♂) **194***M. simplicipes* Curran (♂). Metaleg, anterior view **195***M. caffer* (Loew) (nominal morph) (♂) **196***M. caffer* (Loew) (spined morph) (♂). Abbreviations: bt-basitarsus, fem-femur, tib-tibia.

***Legs.*** All legs chocolate-brown to black; distal ends black; other tarsi black. Proleg (Figs 156, 165): Femur dorsoventrally flattened; with apical pile brush of long dense and curved thick black pile dorsally and thick yellow pile ventrally; ventrally with long black and shorter yellow pile. Tibia black; with long black pile. Basitarsus black and orange; with tuft of black pile on posterior side. Tarsi 2–4 black, tarsomere 5 white. Mesoleg (Fig. 177): Femur with long yellow pile posterodorsally; short black pile ventroproximally. Tibia curved; proximal half compressed. Metaleg (Fig. 188): Femur with very long and thin yellow pile, especially on anterior and posterior side; with shorter black pile ventrally and posteriorly. Tibia with long black pile; without groove on posterior side.

**Figures 197–200.**
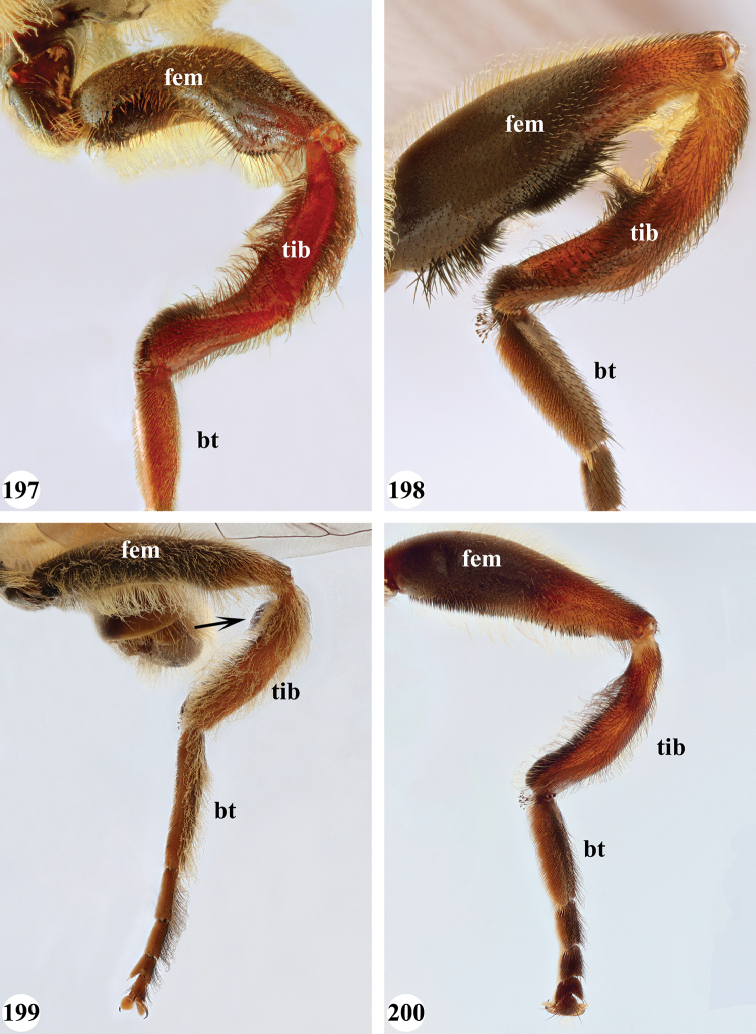
*Mesembrius* spp., metaleg, ventral view **197***M. strigilatus* (Bezzi) (♂). Metaleg, ventral view **198***M. minor* (Bezzi) (♂). Metaleg, frontal view **199***M. copelandi* sp. nov. (♂). Metaleg, posterior view **200***M. senegalensis* (Macquart) (♀). Abbreviations: bt-basitarsus, fem-femur, tib-tibia.

***Wing*** (Fig. 149). Entire wing uniformly dense microtrichose.

**Figures 201–204.**
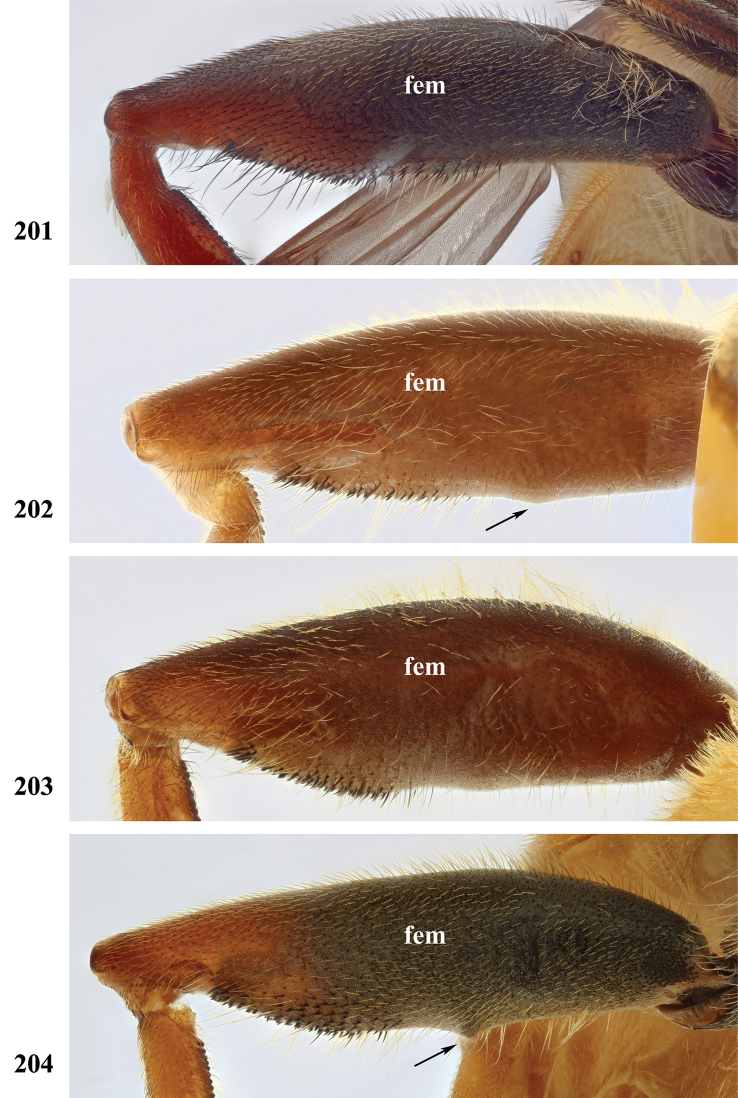
*Mesembrius* spp., metaleg, anterior view **201***M. chapini* Curran (♀). Metaleg, posterior view **202***M. regulus* (Hull) (♀) **203***M. strigilatus* (Bezzi) (♀). Metaleg, anterior view **204***M. minor* (Bezzi) (♀). Abbreviations: fem-femur.

***Abdomen*** (Fig. 105). Tergite II with a pair of very large yellow, rounded maculae; black marking hourglass-shaped; yellow pilose except for short, black pile on the posterior black marking; posterior marking white pollinose. Tergite III and IV with orange fascia; short orange pile in medial part of tergites; long yellow-orange pilose on lateral sides; with white pollinose triangular posterior area.

**Figures 205–216.**
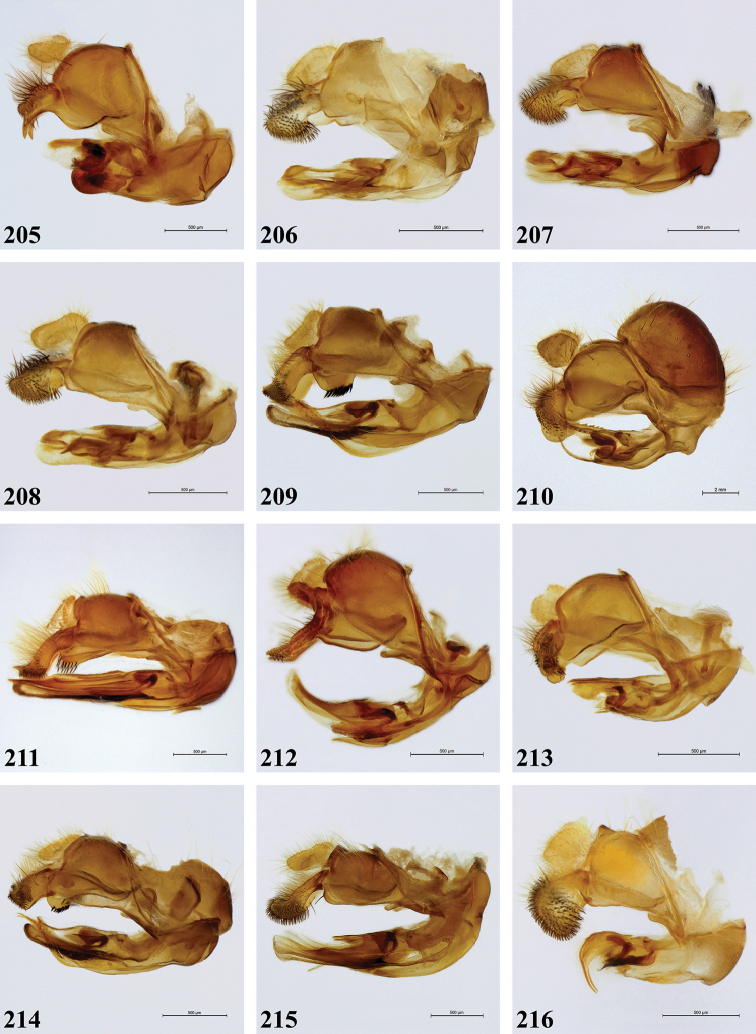
*Mesembrius* spp., male genitalia, lateral view **205***M. arcuatus* sp. nov. **206***M. caffer* (Loew) (nominal morph) **207***M. caffer* (Loew) (spined morph) **208***M. ctenifer* Hull syn. nov. **209***M. capensis* (Macquart) **210***M. chapini* Curran **211***M. copelandi* sp. nov. **212***M. cyanipennis* (Bezzi) **213***M. ingratus* (Loew) **214***M. longipilosus* sp. nov. **215***M. madagascariensis* Keiser **216***M. minor* (Bezzi).

***Genitalia*** (Fig. 227). Epandrium: Dorsal lobe of surstylus short, broadly rounded; covered in short black spines and some longer pile. Ventral lobe of surstylus straight; bare.

**Figures 217–228.**
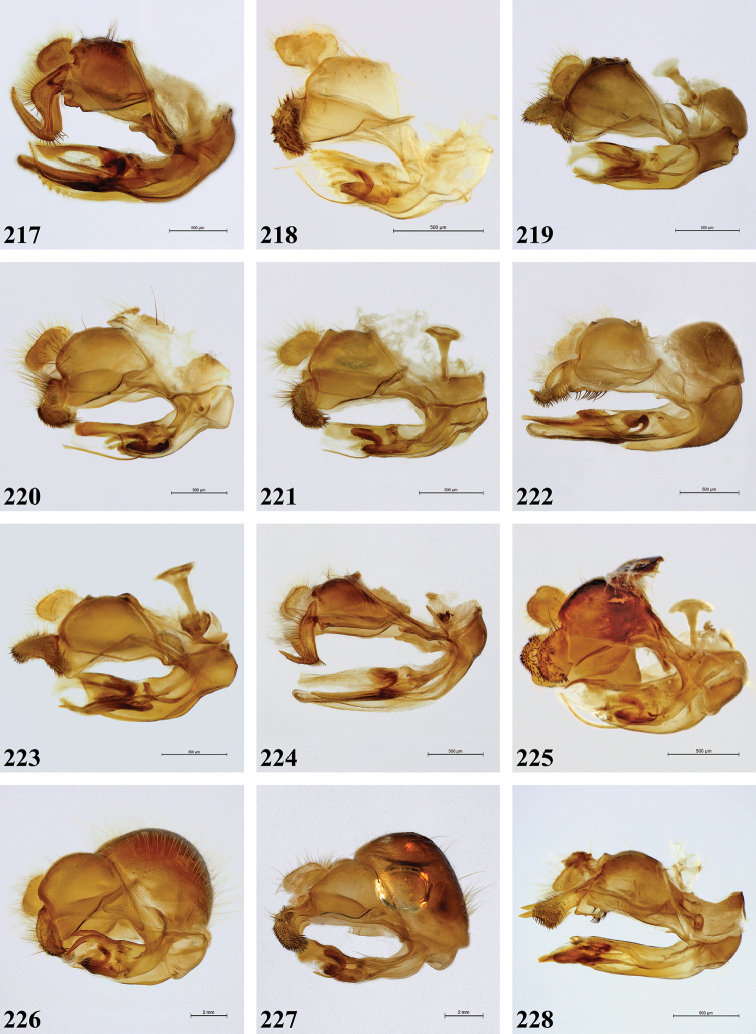
*Mesembrius* spp., male genitalia, lateral view **217***M. nigriceps* Curran **218***M. perforatus* (Speiser) **219***M. platytarsis* Curran syn. nov. **220***M. regulus* (Hull) **221***M. rex* Curran **222***M. senegalensis* (Macquart) **223***M. simplicipes* Curran **224***M. strigilatus* (Bezzi) **225***M. sulcus* sp. nov. **226***M. tarsatus* (Bigot) **227***M. tibialis* sp. nov**. 228***M. vockerothi* sp. nov.

##### Female.

Unknown.

##### Distribution.

Togo.

##### Comments.

This is a new species that is only known from three males from Kloto Forest, Togo.

##### Etymology.

The specific epithet *tibialis* is derived from the Latin word *tibia* (pertaining to the tibia) and was chosen in reference to the mesotibia, which is curved and proximally compressed. It is to be treated as an adjective (nominative singular masculine).

#### Mesembrius vockerothi

Taxon classificationAnimaliaDipteraSyrphidae

Jordaens, Goergen & De Meyer sp. nov.

2D27B621-B82A-5446-BA6D-65C5ACCF7564

http://zoobank.org/7EE04C53-0B7E-4D02-AB1D-0C71EABC9F7C

Figs 26 , 45 , 69 , 82 , 106 , 126 , 150 , 228

##### Differential diagnosis.

*Mesembrius vockerothi* sp. nov. is the smallest of the *Mesembrius* species. Males lack an apical pile brush on the profemur, have an unmodified metatibia and are dichoptic and the face is markedly conical. It can be distinguished from the male of other species by its smaller size and the conical face. The yellow pile on the mesotarsomeres is inconspicuous (very prominent on all tarsomeres in *M. capensis*) and the scutellum is yellow pilose with short black pile interspersed on its entire surface (yellow pilose only in both morphotypes of *M. caffer*, *M. capensis*, *M. minor* and *M. senegalensis*; yellow pilose with black pile in posterior half in *M. strigilatus*). Females have a frons which is pale pilose on the ventral half. It can be distinguished from the female of other species by its smaller size and the conical face. The pro- and metafemur are dark brown to black (yellow-brown in *M. senegalensis*). Tergite II has a pair of yellow maculae (fascia in *M. capensis* and spined morph of *M. caffer*) and the black posterior marking extends to the lateral margins (not so in *M. minor*). The metafemur has no ventral swelling in the middle (present in *M. minor*). The pro- and mesotarsi are uniformly dark brown (brown with a darker medial part in *M. minor*). The posteroventral side of the metafemur has short black setae at distal 1/2 to 1/3 (only at distal 1/6 in the nominal morph of *M. caffer* and in *M. strigilatus*).

##### Examined material.

*Mesembrius vockerothi* Jordaens, Goergen & De Meyer: Holotype, male,” “UGANDA: // Kampala, // 12.xii.1934, // F.W. Edwards. //B.M. 1935-203.” “HOLOTYPUS” “Mesembrius vockerothi // Jordaens & De Meyer 2019” “NHMUK 010369964” [NHMUK].

***Paratypes*:** Democratic Republic of the Congo • 1♀; Kalembelembe, Baraka; Jul 1918; R. Mayné leg.; RMNH • 1♀; North-Kivu, Beni à Lesse; Jul 1911; Murtula leg.; KMMA. Kenya • 1♂; Jinja; Oct 1930; van Someren leg.; NHMUK • 1♀; Nyeri; Oct 1948; van Someren leg.; NHMUK. Uganda • 1♂ 1♀; Entebbe; 17 Aug 1911; C.C. Gowdey leg.; NHMUK • 2♂♂; Entebbe; 9 Nov 1971; H. Falke leg.; CNC • 1♂; Entebbe; 5 Jan 1972; H. Falke leg.; CNC • 1♀; Kampala; 12–20 Mar 1918; C.C. Gowdey leg.; NHMUK • 3♂♂; Kampala; 12 Dec 1934; F.W. Edwards leg.; NHMUK • 1♂ 1♀; Namanue; 13 Dec 1934; J. Ford leg.; NHMUK • 1♀; Tero Forest; 26–30 Sep 1911; S.A. Neave leg.; NHMUK • 1♀; Unyoro District; C.H. Marshall leg.; NMSA • 1♀; Central Region, Wakiso District, Mabamba Swamp; 16 Dec 2018; G. Ståhls leg.; MZH.

##### Other material

 1♀ with locality and date unknown, D. Bruce leg. (NHMUK).

##### Description male

**(Fig. 26).** Body length: 11.0–13.2 mm. Wing length: 9.6–10.5 mm.

***Head*** (Fig. 69). Eyes dichoptic; distance between eyes approx. the width of anterior ocellus. Face conical; white with dark medial vitta; white pilose. Vertical triangle black; black pilose; yellow pollinose on ventral half. Distance between lateral ocellus and eye margin 1/2 width of ocellus. Occiput yellow; yellow pilose; yellow and white pollinose. Frontal triangle short; yellow-white; with long, black pile medially, yellow pilose on gena; yellow pollinose. Frontal prominence shiny dark brown to black. Antenna dark brown, antennal arista reddish-brown.

***Thorax.*** Scutum black with, dorsally, a pair of well-demarcated white pollinose vittae which are connected posteriorly; lateral white pollinose vitta clear; yellow pilose. Scutellum yellow-brown; yellow pilose with shorter black pile interspersed on its entire surface.

***Legs.*** All femora dark brown to black, except for extreme distal ends which are orange-brown; femora yellow to orange. Pro- and mesoleg: Femur with black pile on anterior and dorsal side and with longer yellow pile on posterior and posterodorsal sides. Tarsi yellow to orange. Metaleg: Femur with long and thin yellow pile; with black pile ventrally on distal half. Tibia with yellow and black pile, of which the yellow pile is longer on posterodorsal side. Metatibia unmodified. Metatarsi dark brown.

***Wing*** (Fig. 150). Entire wing uniformly very dense microtrichose.

***Abdomen*** (Fig. 106). Tergite II with a pair of very large yellow-orange, rounded maculae; black marking hourglass-shaped; posterior black marking equal in size to anterior black marking and with a medial white pollinose area; yellow pilose, but black pilose on posterior half of black marking. Tergite III with a pair of large yellow-orange maculae; with large black marking on posterior 2/3; yellow pilose on maculae, black pilose on black marking. Tergite IV black, with a pair of small yellow maculae in anterolateral corners; white pilose and strongly white pollinose on anterior and lateral parts; predominantly black pilose on black marking.

***Genitalia*** (Fig. 228). Epandrium: Dorsal lobe of surstylus distally broadly rounded, with characteristic large tooth-like projection; entirely pilose, except on tooth and at basis (stalk). Ventral lobe of surstylus with one large black setula in middle section and a row of 4–5 long black setulae.

##### Description female

**(Fig. 45).** Body length: 14.0–15.1 mm. Wing length: 9.7–10.3 mm.

As male, except for the following character states: Eyes dichoptic (Fig. 82). Frons white pilose, brown pilose on ocellar triangle and surrounding area; strongly white pollinose to just before ocellar triangle. Pile on legs shorter. Abdomen as in Fig. 126.

##### Distribution.

Democratic Republic of the Congo, Kenya and Uganda.

##### Comments.

This is a new species and the smallest in size of all Afrotropical *Mesembrius* hitherto known. It is the only Afrotropical *Mesembrius* species with a conical face.

##### Etymology.

Named in honour of the Dipterist Dick Vockeroth (1928–2012), who already indicated on the labels that some specimens from Uganda probably belonged to a new species. The specific epithet should be treated as a noun in the genitive case.

## Discussion

In total, we recognise 23 valid *Mesembrius* s.s. species in the Afrotropical Region. Six of these are new to science: *Mesembrius arcuatus* sp. nov., *M. copelandi* sp. nov., *M. longipilosus* sp. nov., *M. sulcus* sp. nov., *M. tibialis* sp. nov. and *M. vockerothi* sp. nov. The males of two very rare species, *M. maculifer* and *M. morio*, are unknown, while the female is unknown for *M. arcuatus* sp. nov., *M. ingratus*, *M. longipilosus* sp. nov., *M. nigriceps*, *M. perforatus* and *M. tibialis* sp. nov.

**Figure 229.**
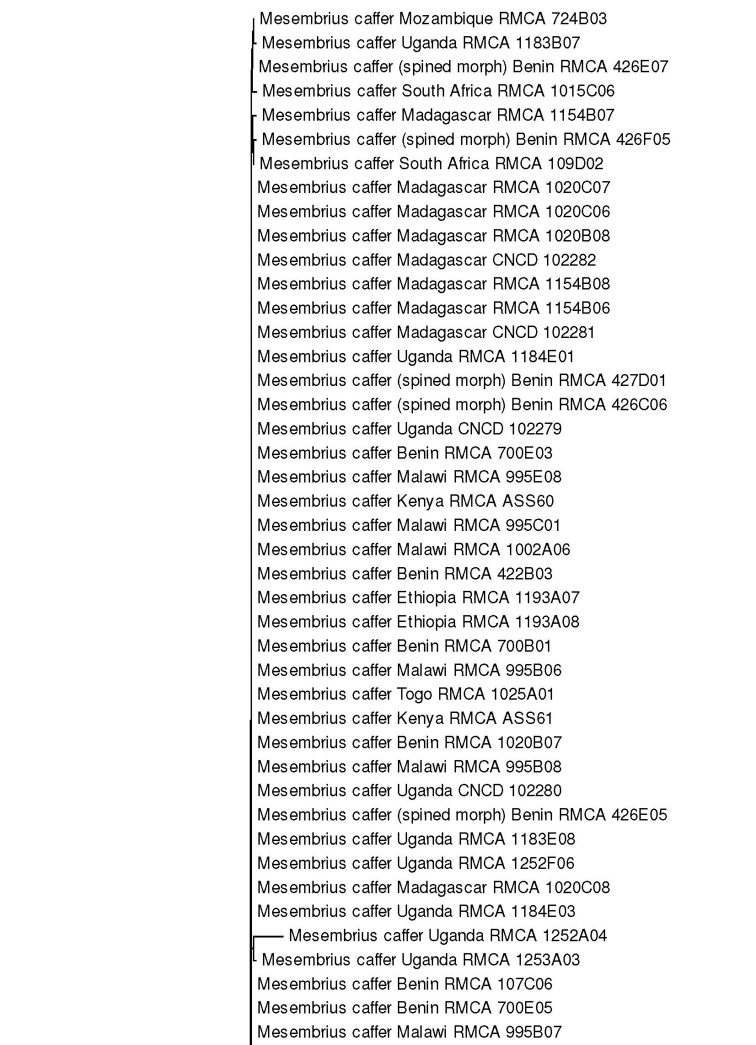
Neighbour-Joining tree (K2P distances) of 236 DNA barcodes of 18 Afrotropical *Mesembrius* species. *Eristalis tenax* was used as outgroup. (Part 1).

**Figure 229.**
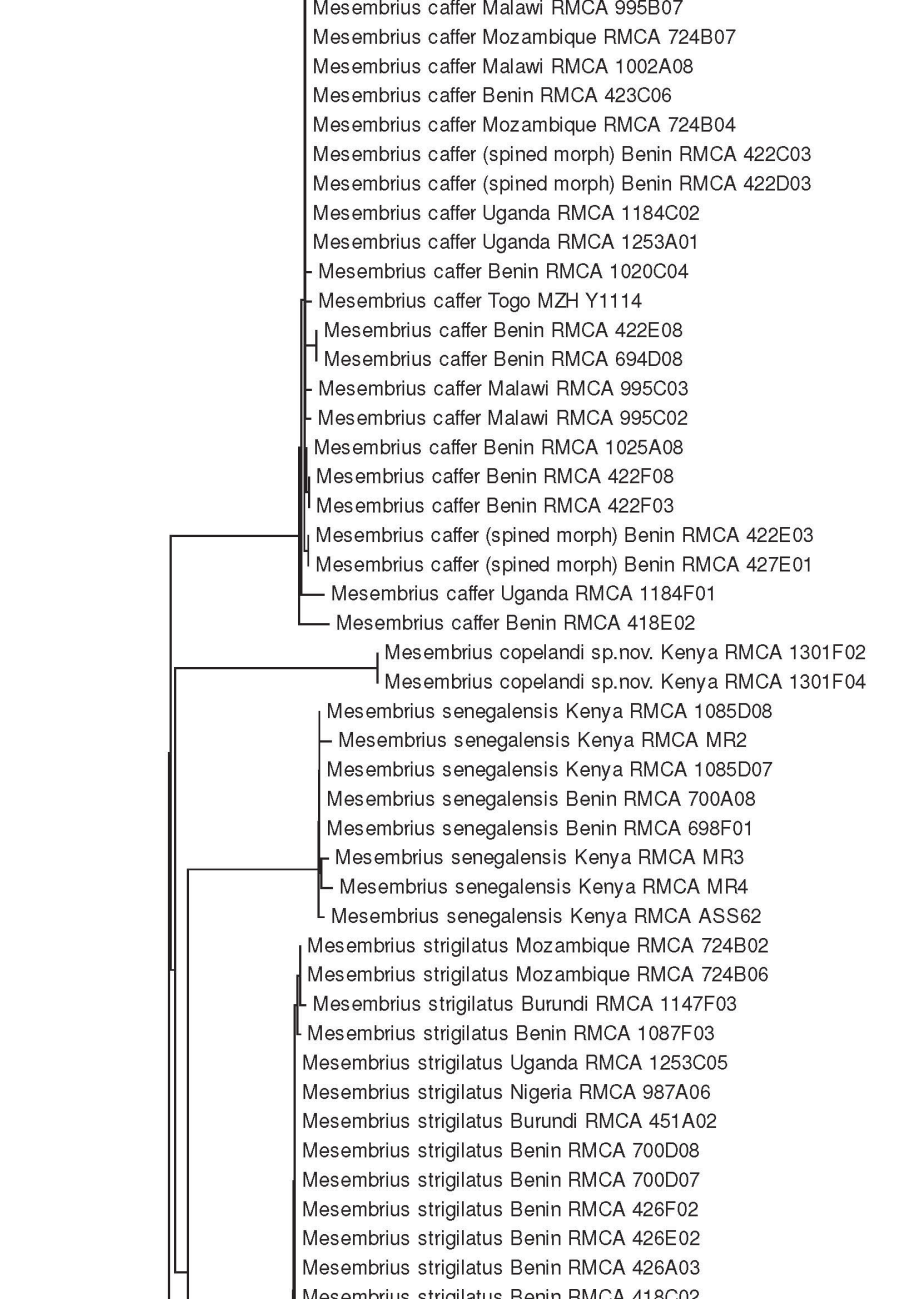
Continued. (Part 2).

**Figure 229.**
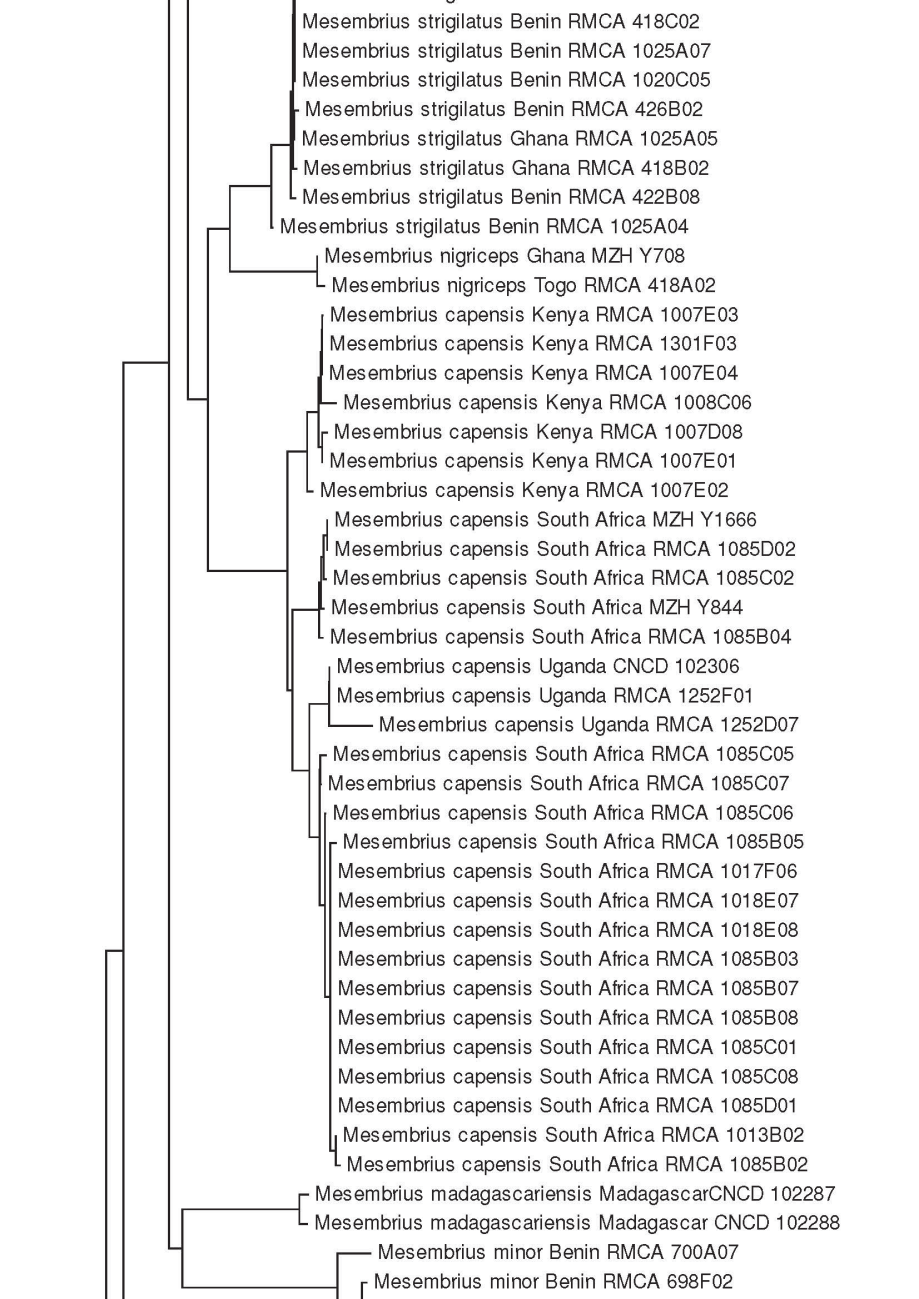
Continued. (Part 3).

**Figure 229.**
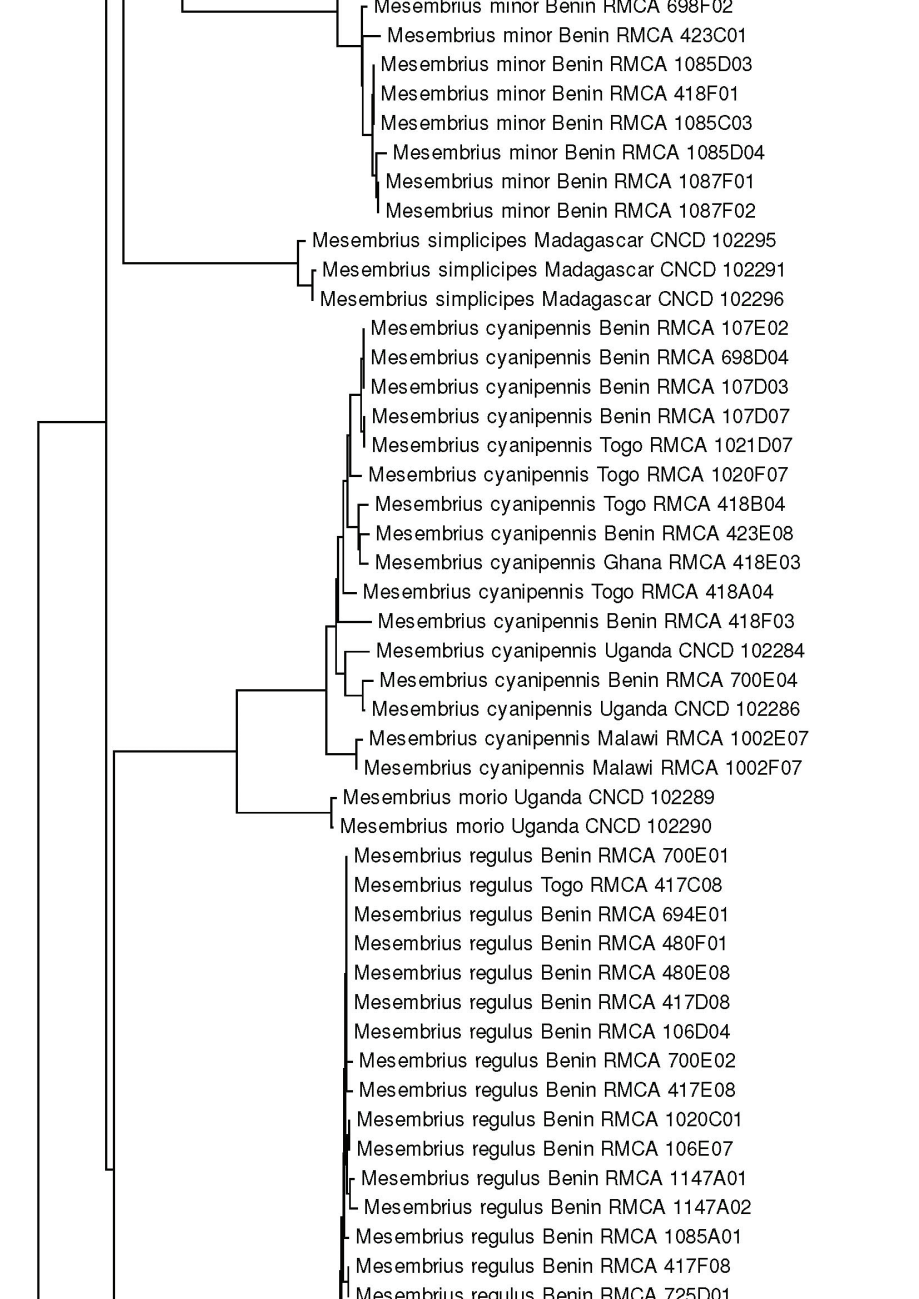
Continued. (Part 4).

**Figure 229.**
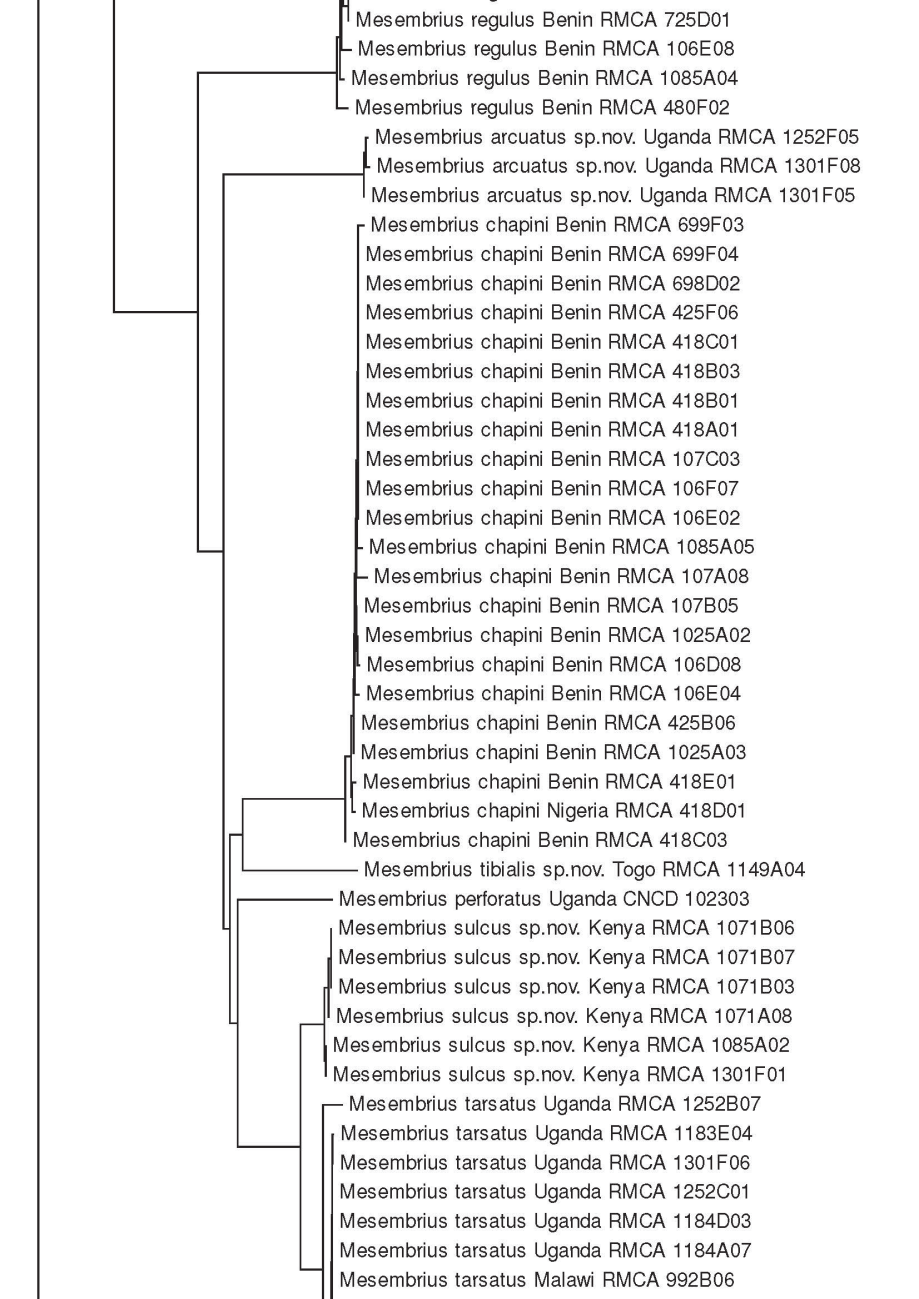
Continued. (Part 5).

**Figure 229.**
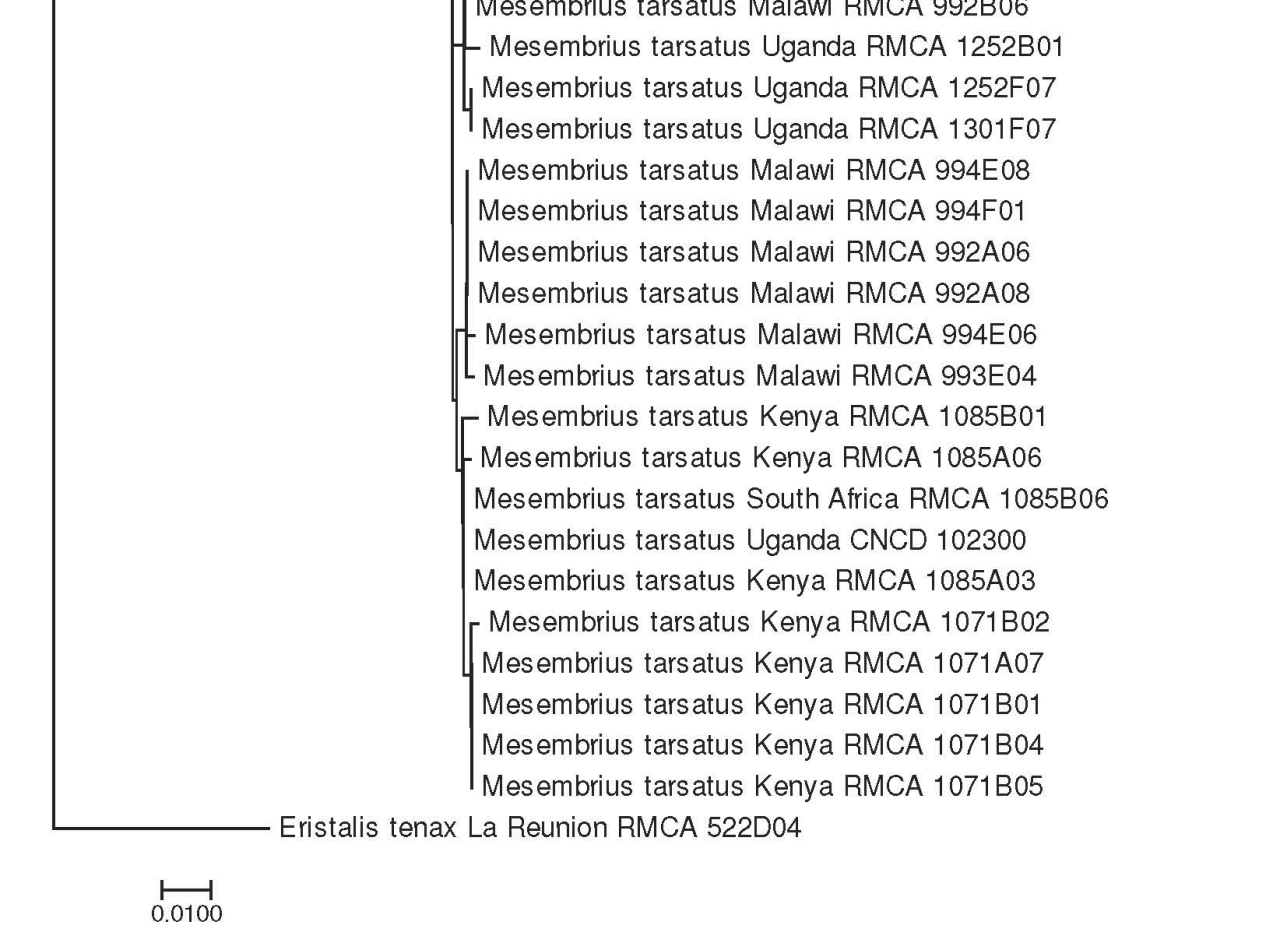
Continued. (Part 6).

Interestingly, the species can be divided into a group of species with a strong sexual dimorphism and a group of species where the sexual dimorphism is very weak. Whereas the former group of species shows strong support in the NJ- and ML-analyses, the latter group of species does not. In the clade of species with a strong sexual dimorphism, the males are characterised by the extensive strong pilosity on the pro- (and for some taxa also on the meta-) legs and the grooves, swellings and/or depressions in the metatibia. In the species group with a weak sexual dimorphism, the males are devoid of conspicuous pilosity on the prolegs and the metatibia are unmodified.

In general and especially for the species where males have an apical pile brush on the profemur, males are more commonly observed than females. A potential explanation could be that males of these species are often found in forests where they seem to defend small sunny patches and that females have a less conspicuous lifestyle. Species, in which the males do not have an apical pile brush on the profemur, occur in higher densities in more open habitat and are, therefore, more easily collected (Jordaens and Goergen pers. obs.). It would be worthwhile to compare the mating behaviour of the two male morphs. In some leaf cutter bees (genus *Megachile* Latreille, 1802; Hymenoptera: Megachilidae), males and females mate on the ground. Thereby, the male grasps the female under the abdomen with its metalegs, prevents the female from flying by restraining her with his mesolegs and covers the female’s eyes with a flattened pad on the protarsi (Wittmann and Blochtein 1995). Other *Megachile* species have no such male adaptations and mate in flight. As for leaf cutter bees, we speculate that the apical pile brush of males in some species of *Mesembrius* is a secondary sexual character and plays an active role in their mating behaviour, although we have no mating observations on Afrotropical *Mesembrius*.

**Figure 230.**
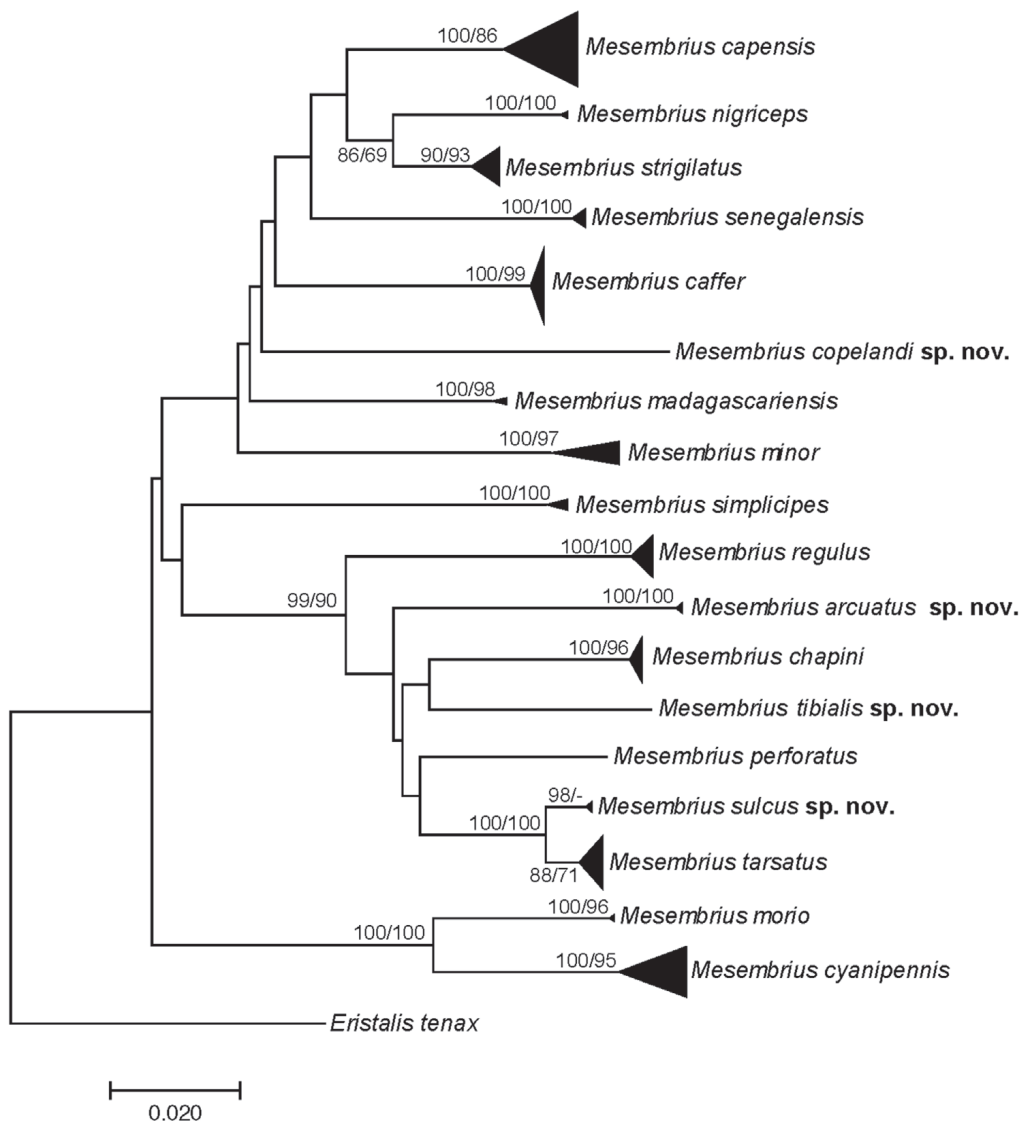
Phylogenetic tree of 18 Afrotropical *Mesembrius* species and with *Eristalis tenax* as outgroup. Bootstrap values ≥ 70% are presented at the nodes as (NJ/ML).

In species with a strong sexual dimorphism, male surstyli are simple (i.e. short, broadly rounded and covered with short, black spines) and male genitalia are morphologically very similar amongst species (Figs 206–208, 210, 213, 218–221, 225–227). The only exception is the male of *M. arcuatus* sp. nov., which has a long, sharp extension on the distal end of the dorsal surstylar lobe (Fig. 205). In contrast, in the species group with weak sexual dimorphism, the male genitalia are strongly differentiated in the size and shape of the surstyli: often elongated, curved, flattened or broadened and with a variety of short or long, thin or thick pile, setulae and spines, for some also on the ventral side of the ventral lobe (Figs 209, 211, 212, 214, 215, 217, 222–224, 228). The only exception is *M. minor* which has simple surstyli (Fig. 216), i.e. similar to those seen in the species group with strong sexual selection.

In summary, Afrotropical *Mesembrius* seems composed of species with weak sexual dimorphism, where males show species-specific surstyli morphology and adults occur in open habitats and of species with strong sexual dimorphism, where males have simple surstyli and adults occur in forests. It remains to be investigated whether these marked differences translate into marked differences in mating behaviour and/or strategies, as has been observed in leaf cutter bees of the genus *Megachile*.

The DNA barcode analysis shows very low intraspecific variation in all species (Suppl. material 2: Table S2 and Suppl. material 1: Table S1) (range *p*-distances: 0–1.4%). Except for the low mean interspecific distance of 1.6% between *M. sulcus* sp. nov. and *M. tarsatus*, all other mean interspecific *p*-distances are high (mean: 9.4%; range: 4.3–14.7%) (Suppl. material 2: Table S2). Hence, all Afrotropical *Mesembrius* species, for which DNA barcodes could be sequenced, can be identified using DNA barcodes (Fig. 229).

The NJ- and ML-analyses of the COI barcode region (Fig. 230) show strong support for a clade of species with strong sexual dimorphism. Other deeper nodes are not supported (e.g. there is no support for a clade of species with weak sexual dimorphism) and thus, the phylogenetic relationships amongst the species requires further study. Indeed, our preliminary analyses only suggest three sister-species relationships. First, *M. nigriceps* and *M. strigilatus* are sister-species in the NJ-analysis, but not in the ML-analysis and show relatively similar male genital morphology. Moreover, *M. nigriceps* looks like a very dark *M. strigilatus*. Secondly, *M. morio* and *M. cyanipennis* seem sister-species. Curran (1927) considered *M. morio* to be a dark morphotype of *M. cyanipennis*, but the strong DNA barcode differentiation (5.7%), which is within the range what is observed between other *Mesembrius* species (4.2–14.6%), suggests that both warrant species status. The male of *M. morio* is unknown so we could not compare the male copulatory organs of both species. Thirdly, *M. sulcus* sp. nov. and *M. tarsatus* show low differentiation with DNA barcoding (mean *p*-distance: 1.6%), but male and female external morphology are substantially different. Probably, the latter two species have recently diverged. The male genitalia of both species are morphologically very similar, as is the case for all Afrotropical *Mesembrius* species that show sexual dimorphism (except *M. arcuatus* sp. nov.). A phylogenetic study of the full mtDNA of *Mesembrius* is currently ongoing to shed light on the evolutionary relationships of Afrotropical *Mesembrius* and on the evolution of strong sexual dimorphism in the genus.

## Supplementary Material

XML Treatment for Mesembrius

XML Treatment for Mesembrius arcuatus

XML Treatment for Mesembrius caffer

XML Treatment for Mesembrius capensis

XML Treatment for Mesembrius chapini

XML Treatment for Mesembrius copelandi

XML Treatment for Mesembrius cyanipennis

XML Treatment for Mesembrius ingratus

XML Treatment for Mesembrius longipilosus

XML Treatment for Mesembrius maculifer

XML Treatment for Mesembrius madagascariensis

XML Treatment for Mesembrius minor

XML Treatment for Mesembrius morio

XML Treatment for Mesembrius nigriceps

XML Treatment for Mesembrius perforatus

XML Treatment for Mesembrius regulus

XML Treatment for Mesembrius rex

XML Treatment for Mesembrius senegalensis

XML Treatment for Mesembrius simplicipes

XML Treatment for Mesembrius strigilatus

XML Treatment for Mesembrius sulcus

XML Treatment for Mesembrius tarsatus

XML Treatment for Mesembrius tibialis

XML Treatment for Mesembrius vockerothi

## Supplementary materials

Supplementary material 1 Table S1. List of specimens, collection information, DNA voucher codes and GenBank/Barcode of Life Datasystems (BOLD) accession numbers used for the DNA barcode and phylogenetic analyses Data type species data https://binary.pensoft.net/file/557527This dataset is made available under the Open Database License (http://opendatacommons.org/licenses/odbl/1.0/). The Open Database License (ODbL) is a license agreement intended to allow users to freely share, modify, and use this Dataset while maintaining this same freedom for others, provided that the original source and author(s) are credited.Kurt Jordaens, Georg Goergen, Jeffrey H. Skevington, Scott Kelso, Marc De Meyer

Supplementary material 2 Table S2 Data type molecular data Explanation note Intra- (diagonal and in bold) and interspecific (below diagonal) mean uncorrected *p*-distances, based on the barcode region of the mitochondrial cytochrome *c* oxidase subunit I gene in Afrotropical *Mesembrius* s.s. https://binary.pensoft.net/file/557528This dataset is made available under the Open Database License (http://opendatacommons.org/licenses/odbl/1.0/). The Open Database License (ODbL) is a license agreement intended to allow users to freely share, modify, and use this Dataset while maintaining this same freedom for others, provided that the original source and author(s) are credited.Kurt Jordaens, Georg Goergen, Jeffrey H. Skevington, Scott Kelso, Marc De Meyer
